# Computational Bacterial Genome-Wide Analysis of Phylogenetic Profiles Reveals Potential Virulence Genes of *Streptococcus agalactiae*

**DOI:** 10.1371/journal.pone.0017964

**Published:** 2011-04-04

**Authors:** Frank Po-Yen Lin, Ruiting Lan, Vitali Sintchenko, Gwendolyn L. Gilbert, Fanrong Kong, Enrico Coiera

**Affiliations:** 1 Centre for Health Informatics, University of New South Wales, Sydney, Australia; 2 School of Biotechnology and Biomolecular Sciences, University of New South Wales, Sydney, Australia; 3 Centre for Infectious Diseases and Microbiology-Public Health, Westmead Hospital and Sydney Medical School, The University of Sydney, Sydney, Australia; The University of Hong Kong, Hong Kong

## Abstract

The phylogenetic profile of a gene is a reflection of its evolutionary history and can be defined as the differential presence or absence of a gene in a set of reference genomes. It has been employed to facilitate the prediction of gene functions. However, the hypothesis that the application of this concept can also facilitate the discovery of bacterial virulence factors has not been fully examined. In this paper, we test this hypothesis and report a computational pipeline designed to identify previously unknown bacterial virulence genes using group B streptococcus (GBS) as an example. Phylogenetic profiles of all GBS genes across 467 bacterial reference genomes were determined by candidate-against-all BLAST searches,which were then used to identify candidate virulence genes by machine learning models. Evaluation experiments with known GBS virulence genes suggested good functional and model consistency in cross-validation analyses (areas under ROC curve, 0.80 and 0.98 respectively). Inspection of the top-10 genes in each of the 15 virulence functional groups revealed at least 15 (of 119) homologous genes implicated in virulence in other human pathogens but previously unrecognized as potential virulence genes in GBS. Among these highly-ranked genes, many encode hypothetical proteins with possible roles in GBS virulence. Thus, our approach has led to the identification of a set of genes potentially affecting the virulence potential of GBS, which are potential candidates for further *in vitro* and *in vivo* investigations. This computational pipeline can also be extended to *in silico* analysis of virulence determinants of other bacterial pathogens.

## Introduction

Virulence - the ability of a pathogen to damage a host and evade host immune defenses - arises from a range of complex host-pathogen interactions and can be expressed as the pathogen's toxicity, invasiveness, colonization, and ability to be transmitted to another host [1], [2]. Contemporary methods of searching for the genetic determinants of virulence exploit the differential presence of virulence genes in invasive pathogens compared to their less invasive counterparts. Several criteria have been suggested to help formalize this process including molecular Koch's postulates or adoption of Hill's criteria [3], [4]. In practice, the discovery process usually involves iterative gene screening via labor-intensive laboratory experiments. Given the relentless growth in bacterial genomic data, alternative approaches capable of handling large datasets would facilitate the selection of potential genes of interest and thus accelerate the discovery of new virulence genes.

The search for virulence genes in pathogenic bacteria has been revolutionized over the last decade by comparative genomics [5] with rapid advances in DNA microarrays [6]–[8] and whole-genome sequencing [9]. Purely *in silico* approaches have been suggested as an alternative to costly collections of experimental data. For example, genes that were positively selected in a uropathogenic *E. coli* (UPEC) genome were identified using phylogenetic analysis by maximum likelihood (PAML) of several *E. coli* genomes and verified in a sample of UPEC clinical isolates [10]. While these high-throughput methods are powerful, there are practical limitations: DNA microarrays are limited to detecting genes for which allelic variants have already been characterized and may miss emerging mutations; the PAML-based approach requires multiple genomes of phenotypic variants of the same species, which are not always available.

**Figure 1:**
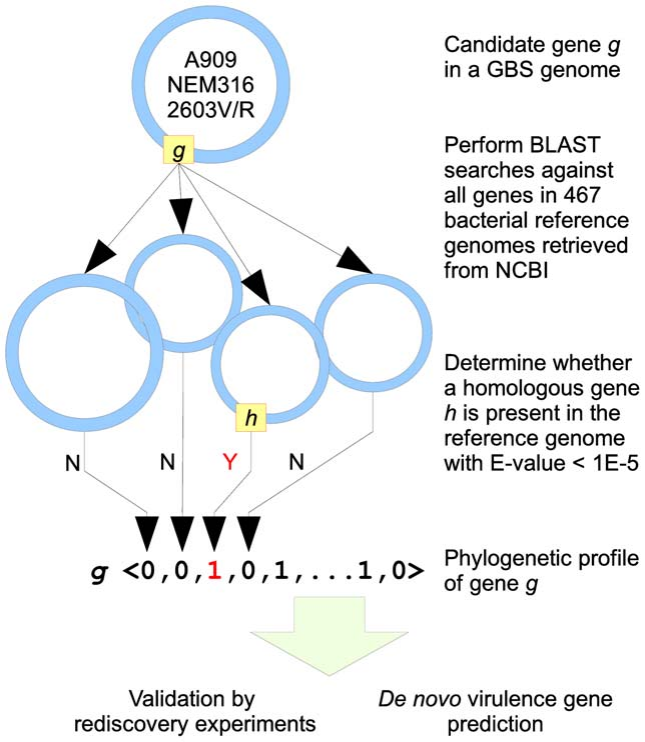
Determination of phylogenetic profiles. For each gene, a candidate-against-all BLAST was performed to determine whether at least one homolog of a candidate gene is present in a given reference genome. The binary values of presence (1) or absence (0) were stored in a vector which were used for subsequent rediscovery analyses and virulence gene predictions.

This study utilized an alternate approach that identifies genes with similar *phylogenetic profiles* (PPs). A PP is defined as a binary vector indicating the presence or absence of homologs to the gene in the reference genomes (Figure 1) and represents the evolutionary history of the gene among phylogeny of life. Functionally similar genes are assumed to have distinct yet conserved evolutionary “footprints” in different strains, species, and genera. While patterns of PP have been utilized to predict gene functions in other setting [11]–[16], they have not been systematically applied to the discovery of bacterial virulence factors. We have developed and validated a computational method of *inductive candidate gene prioritization* (ICGP) to predict bacterial gene functions through the recognition of specific PP signatures [17]. We expect ICGP to be applicable to the discovery of bacterial virulence factors, in the same way that various forms of host-pathogen interaction, such as epithelial adhesion or mucosal invasion, may also possess specific fingerprints that allow their discovery through an *in silico*, cross-genomic analysis.

We hypothesized that the ICGP method can also facilitate the discovery of previously unrecognized bacterial virulence genes and tested this hypothesis using an important bacterial pathogen, *Streptococcus agalactiae*, or group B streptococcus (GBS), as an example. GBS is the leading cause of neonatal sepsis in developed countries [18] and GBS infection remains a significant burden despite implementation of screening programs and antibiotic chemoprophylaxis [19]–[21]. While experimental studies have identified many GBS virulence genes [22], [23], it is likely that many others and/or specific allelic variants of known factors, contribute to pathogenesis and should be taken into account in studies of GBS pathogenesis and drug target selection. Discovery of new GBS virulence factors could also contribute to more targeted prenatal screening and facilitate vaccine design [23]. This paper describes the application of ICGP to published bacterial whole genome sequences with a goal of identifying GBS genes with putative roles in virulence that may act synergistically with known genes contributing to pathogenesis of GBS disease.

## Results

### GBS genes contributing to virulence through molecular mechanisms similar to those of genes of other bacterial species can be identified using a PP-based model

We tested the hypothesis that PP can predict whether a GBS gene is associated with virulence. We first determined the PPs by examining which GBS genes from all fully sequenced *S. agalactiae* genomes are also present in 467 reference genomes of other bacterial species. Evaluation experiments were subsequently performed to determine whether virulence genes can be rediscovered by using ICGP trained with functionally-related virulence genes with corresponding PPs. Two rediscovery experiments were performed to evaluate the ICGP models on a “gold standard” dataset comprised of all known GBS virulence genes. Virulence genes were assigned to three major categories, namely, adhesins, invasins, and immune evasins, and 15 functional gene categories (*fbsA*, *fbsB*, *lmb*, *pavA*, *scpB*, minor pilin cluster, *cyl* cluster, *cfb*, *spb1*, *hylB*, *bca*/*bac*, *cps* and *neu* clusters, *cspA*, and *pbp1A*, Table 1). The first experiment sought to determine whether ICGP could rediscover currently known virulence genes within a genome of *S. agalactiae* serotype III (NEM316, GenBank accession: AL732656). Among the four algorithms used in ICGP evaluations, support vector machines (SVM) with radial basis (RBF) and linear kernel algorithms were the most successful in rediscovering these genes (Table 2) with area under receiver operating characteristic (ROC) curve (AUC) of approximately 0.8 evaluated using 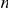-fold cross-validation. In particular, the gene clusters encoding GBS pilus and sialic acid synthases (*neu* cluster) achieved almost perfect AUC (>0.98) in the rediscovery task, indicating that ICGP is able to distinguish functional groups of genes responsible for specific bacterial virulence mechanisms.

**Table 1:** List of known GBS virulence genes with systematic gene names in three published reference genomes.

			Systematic name/loci in reference genomes			
Category	Gene	Function/annotation	NEM316 (III)	A909 (Ia)	2603 (V)	Ref.
Adhesins	*fbsA*	fibrinogen-binding protein FbsA	GBS1087	SAK1142	SAG1052	[S1-4]
	*fbsB*	fibrinogen-binding protein FbsB	GBS0850	SAK0955	SAG0832	[S4,5]
	*pavA*	fibronectin-binding protein	GBS1263	SAK1277	SAG1190	[S6]
	*scpB*	C5a peptidase	GBS1308	SAK1320	SAG1236^a^	[S7,8]
	*lmb*	laminin-binding protein	GBS1307	SAK1319	SAG1234	[S9-11]
	GBS pilus cluster	streptococcal pilus cluster	GBS0628-32	SAK0776-80	SAG0645-49	[S12-14]
Invasins	*cyl* b	*β*-hemolysin/cytolysin	GBS0644-55	SAK0790-0801	SAG0662-73	[S20-26]
	*cfb*	CAMP factor	GBS2000	SAK1983	SAG2043	[S27]
	*spb1*	hemolysin III	GBS1477	SAK1440	SAG1407	[S27,S31]
	*hylB*	hyaluronate lyase	GBS1270	SAK1284	SAG1197	[S28-30]
	*rib* c	surface protein rib	GBS0470		SAG0433	[S15-19]
	*bca* c	C-*α* protein		SAK0517		[S15-19]
Immune evasins	*bac*	C-*β* protein	-	SAK0186		[S32-34]
	*cps*	*cps* gene cluster	GBS1237-47	SAK1251-62	SAG1162-75	[S35-37]
	*neu*	*neu* gene cluster	GBS1233-36	SAK1247-50	SAG1158-61	[S38-41]
	*scpB* d	C5a peptidase	(see above)			[S7,8]
	*cspA* c	serine protease cspA	GBS2008	SAK1991	SAG2053	[S42]
	*pbp1A*/*ponA*	penicillin-binding protein 1A	GBS0288	SAK0370	SAG0298	[S43-45]

a. IS*1548* is embedded upstream of *scpB* gene in 2603 V/R.

b. although primarily an invasin, *cyl* is capable of damaging phagocytes and hence also have a role in immune system evasion.

c. dual roles of both an invasin and an immune system evading gene.

d. dual roles of both an adhesin and an immune system evading gene.

Please refer to Text S3 for the reference entries.

**Table 2:** Performance of algorithms (area under ROC curve, AUC) in the rediscovery experiment using only NEM316 genome.

		Algorithms (AUC)			
Virulence gene category	*n*	ADTree	IBk	RBF	Poly
All virulence genes	43	0.721	0.722	**0.804**	0.791
Adhesins	10	0.716	0.776	**0.780**	0.767
minor pilin cluster	5	0.970	0.763	**0.980**	0.881
Invasins	17	0.864	0.679	0.857	**0.880**
*cyl* cluster	12	0.824	0.648^*^	**0.825**	0.820
Immune evasins	17	0.825	0.770	**0.876**	0.860
*cps* cluster	11	0.808	0.797	**0.919**	0.849
*neu* cluster	4	**1.000**	0.836	**1.000**	**1.000**
*cps*/*neu* cluster	15	0.864	0.773	**0.925**	0.914

This analysis evaluated the relative performance of each algorithm to rediscover virulence genes by applying stratified *n*-fold cross-validations with 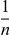 of the entire set of *S. agalactiae* NEM316 genes serving as test-set in each fold. Each fold of training set comprised 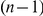 positive and 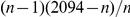 negative examples.*n*: number of virulence genes in the category. Singleton virulence gene categories were excluded from this analysis, as it is not possible to perform cross-validations on training sets with *n* = 1. All but one (labeled^*^) AUCs reached the statistical significance level at *α* = 0.05 (two-tailed Mann-Whitley U-test). At least 3 out of 4 algorithms were still significant after adjustment for multiple testing (across the family of 4 algorithms) by the Bonferroni method. Abbreviations: ADTree: alternating decision tree; IBk: nearest neighbor classifier; SVM: support vector machine; RBF: SVM with radial basis function; Poly: SVM with polynomial kernel. Refer to the methods section for the parameters used to train the machine learning algorithms. The numbers in bold face indicate the best performing algorithm for a given category.

We further examined whether ICGP can rediscover genes with identical PPs. All genes in the published GBS reference genomes NEM316, A909/Ia (GenBank accession: CP000114), and 2603V/R (GenBank accession: AE009948) were selected as candidate genes and *n*-fold cross-validation analyses were performed. The gene categories for cross-validation were identical to the previous experiment. As expected, most categories with exactly one orthologous gene led to a perfect AUC. Overall, the currently known GBS virulence genes were rediscovered with AUCs as high as 0.98 by the nearest-neighbor classifier IBk with all orthologous genes included in the cross-validation set (Table 3). AUCs of better than 0.96, 0.89, and 0.95 were achieved for all genes encoding adhesins, invasins, and immune evasins, respectively (Table 3).

**Table 3:** The performance of inductive CGP algorithms in the rediscovery of known virulence genes in all 3 GBS reference genomes.

		Algorithms (AUC)			
Virulence gene category	*n*	ADTree	IBk	SVM/RBF	SVM/Poly
**All virulence genes**	134	0.848	**0.980**	0.951	0.960
**Adhesins**	30	**0.968**	0.961	0.960	0.965
*fbsA*	3	0.888	0.677	0.754	**0.961**
*fbsB*	3	0.874	**0.971**	0.959	0.957
*lmb*	3	**1**	**1**	**1**	**1**
*pavA*	3	**1**	**1**	**1**	**1**
*scpB* a	3	**1**	**1**	**1**	**1**
minor pilin cluster	15	**1**	**1**	**1**	**1**
**Invasins**	51	0.929	0.974	0.954	**0.982**
*cyl* cluster	36	0.950	**0.988**	0.962	0.980
*cfb*	3	**1**	**1**	**1**	**1**
*spb1*	3	**1**	**1**	**1**	**1**
*hylB*	3	**1**	**1**	**1**	**1**
C-*α* genes b	3	0.933	0.967	**0.979**	0.978
**Immune evasins**	60	0.929	0.974	0.954	**0.982**
*bac* c	1	-	-	-	-
*cps* cluster	37	0.960	0.966	0.948	**0.967**
*neu* cluster	12	**1**	**1**	**1**	**1**
*cps/neu* cluster^d^	49	0.970	0.974	0.960	**0.979**
*cspA ^e^*	3	**1**	**1**	**1**	**1**
*pbp1A*/*ponA*	3	**1**	**1**	**1**	**1**

This rediscovery analysis applied all known GBS virulence genes by applying stratified *n*-fold cross-validations with 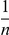 of the entire set of *S. agalactiae* genes in A909, NEM316, and 2603V/R genomes serving as test-set in each fold.*n*: number of genes in the category.

a. *scpB* was also included as immune evasion genes.

b. Including both *bca* and *rib*; also included as immune evasion genes.

c. *bac* was represented by less than two genes in the three reference genomes studied. No rediscovery experiment was performed.

d. Including all genes from the *cps-neu* operon. e. *cspA* was also included as an invasin.

### *De novo* discovery of *S. agalactiae* virulence genes

**Figure 2:**
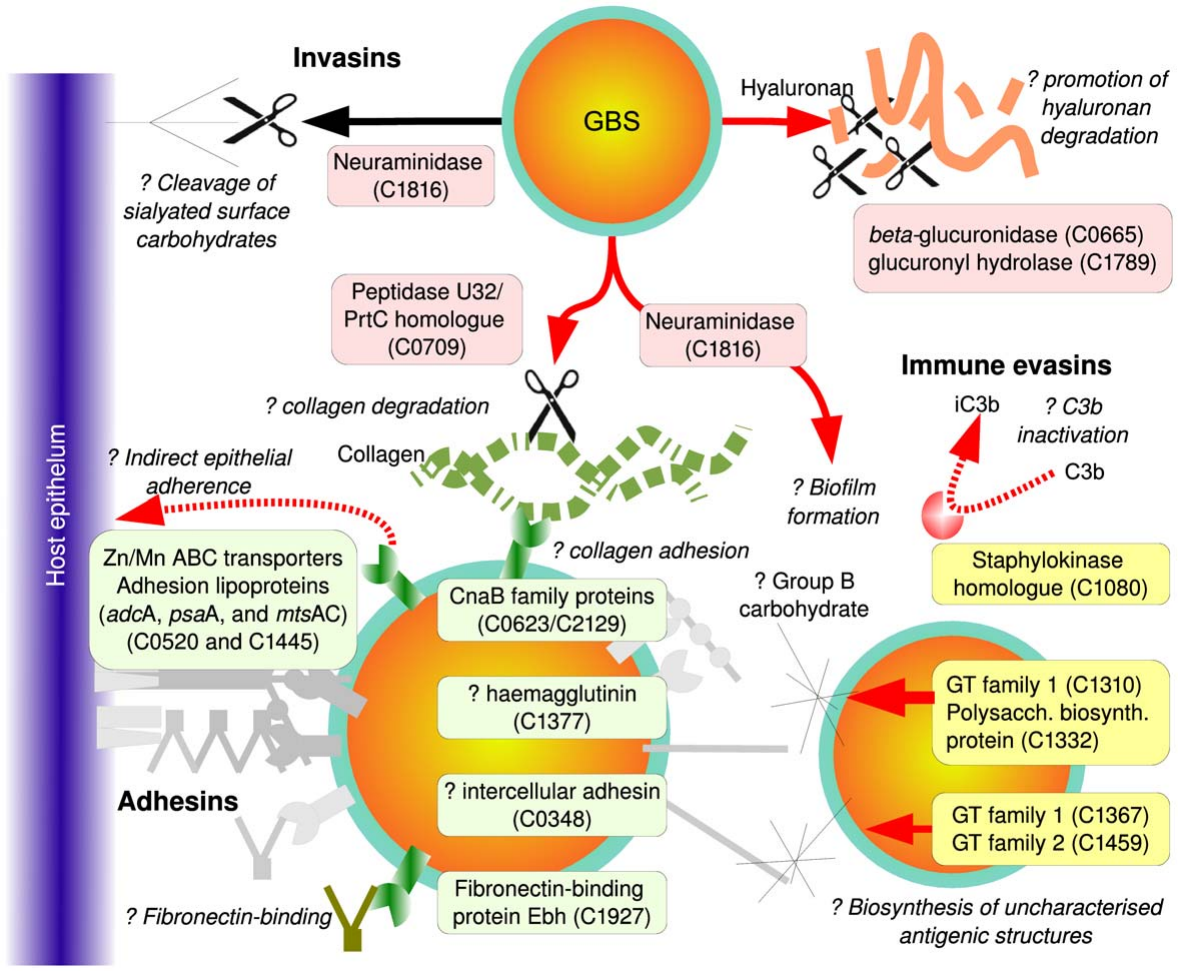
Proposed candidate GBS virulence genes. The figure illustrates the putative *S. agalactiae* virulence genes identified in this paper, of which the biological function have been known in other pathogens or inferred by sequence similarity with known protein motifs. The cluster IDs (C*number*) identify the homolog clusters defined in Table S2.

We prioritized all genes in three GBS reference genomes to find potential virulence genes that are yet to be recognized. To generate the gene ranks, we trained the ICGP models with known virulence factors alongside the corresponding PPs (see methods section) for each of the 15 virulence gene categories (Table 1). The top-10 genes from each category (of less than 0.5% of total open reading frames in a GBS genome) are shown in Figure 2 and listed in Table S1. A total of 119 unique homologous genes (416 genes in three genomes) occupied 150 possible ranks. ICGP rediscovered 11 known GBS virulence genes from 119 homologous genes, equivalent to 48 of 416 genes in all three genomes (11.5%). We estimated that our prioritization method had an overall enrichment of >5.4 folds (compared with baseline 134/6,214 genes used for model training, 2.2%). Sixteen of 119 genes were ranked in more than one category. The highly ranked genes of unknown function encoding hypothetical proteins are listed in Table 4.

**Table 4:** List of genes encoding hypothetical proteins and their putative biological significance.

Cluster	Gene*	In rank(s)	Have orthologs in other genomes with annotations; Contains Pfam Motifs† (E-value)	Predicted function
C0036	GBS0036	*spb1*	DUF386 ( 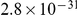 )	
C0255	GBS0253	*fbsA*	quinone-reactive Ni/Fe hydrogenase, cytochrome b subunit	
C0257	GBS0255	*cyl*	lipoprotein	
C0348	GBS0344	*fbsA*	intercellular adhesion protein C	? adhesin
C0429	GBS0488	*cfb*	superfamily II helicase	
C0442	GBS0502	minor pilin	ATP-dependent endopeptidase	
C0560	(absent)	*cfb*	phage protein; DUF1642 ( 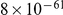 )	
C0613	GBS0616	C-*α*/*β*	DUF1706 ( 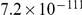 )	
C0753	GBS0806	*cspA*, *fbsA*	Methyltransferase; (Methyltransf_11 domain, 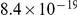 )	? methyltransferase
C1080	GBS1195	*fbsB*	[*skc*] streptokinase plasminogen activator	? staphylokinase analog
C1172	GBS1295	*neu* cluster	DUF208 ( 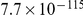 )	
C1271	GBS1415	*fbsA*	DUF2127 ( 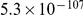 )	
C1332	GBS1482	*cspA*	putative O-antigen transporter;	? synthesis of unknown antigens
			Polysaccharide biosynthesis protein (Polysacc_synt, 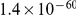 )	
C1377	GBS1529	*fbsB*	streptococcal hemagglutinin; fibrinogen-binding adhesin (SdrG_C_C, 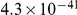 )	? adhesin
C1412	GBS1559	*fbsB*	[*blpX*] bacteriocin self-immunity protein	
C1716	GBS1861	*cfb*	putative DNA-binding protein; YheO-like PAS domain (PAS_6, 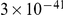 )	
C1856	GBS1961	*fbsA*	RNA-binding protein	
C1860	GBS1992	*cyl*	ABC-type transport system, permease	
C1977	(absent)	*neu*, *fbsA*	filamentation induced by cAMP protein Fic; (Fic family domain, 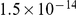 )	
C2042	GBS0486	*scpB*, *lmb*	Methyltransferase (Methyltransf_11 domain, 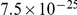 )	? methyltransferase

This table lists the genes encoding hypothetical proteins from the top-10 genes of all 15 functional category listed in Table S1. Cluster refers to the homolog clusters listed in Table S2. In ranks(s): within top-10 of functional categories (ranks). Each hypothetical protein was searched against KEGG [47] and Pfam database [48] to identify potential homologous sequence motifs. Note: *) Systematic gene names in the NEM316 (serotype III) genome. †) Pfam motifs with E-value 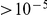 are not presented in the table.

### Many highly ranked genes have known virulence roles in other bacterial pathogens

In addition to the 11 known GBS virulence genes rediscovered, 15 of 119 homologous genes (13%) contributing to mechanisms of virulence in other pathogens were also recognized. This is equivalent to a 10-fold enrichment (91 potential virulence genes identified from the list of 416 genes in 3 GBS genomes, 22%, including genes from GBS and other human pathogens identified in the literature) when compared to current knowledge (2.2%). Several genes encoding putative adhesins were identified; genes encoding metallo-binding adhesion lipoproteins (C0520 and C1445) and permease proteins (C1443 and C0154) were highly ranked. The homologs of these genes in *S. pneumoniae* (*adcAB*, *psaA*, and *mtsAC* genes) promote indirect adherence to epithelial cells and have contributed to virulence in other Gram-positive pathogens [24]. The gene product of C1927 is distantly similar to a large 1.1-MDa surface protein Ebh in *Staphylococcus aureus*, in which fibronectin-binding activities have been demonstrated *in vitro* [25]. Both genes C0623 and C2129 contain a collagen-binding cna-B protein domain; Cna protein is a virulence determinant of staphylococcal septic arthritis in mouse model, and has been implicated in causing keratitis in human [26], [27].

Genes encoding potential invasins have also been recognized within the top-10 of the ranks. For instance, two glycosidic hydrolase genes, the unsaturated glucuronyl hydrolase (*ugl*, C1789) and *β*-glucuronidase genes (C0665), may have putative roles in facilitating the degradation of hyaluronan in synergy with streptococcal hyaluronidase (encoded by *hylB*). The peptidase U32 (C0709) is similar to a metalloprotease gene *prtC* in *Prophyromas gingivalis*, an anaerobe causing periodontitis. PrtC is a known factor in contributing to the degradation of type I collagen in gingivial infection [28].

Genes that encode mechanisms facilitating the evasion of host immune system were also found. For example, a gene encoding neuraminidase homologue (C1816) was highly ranked. In *S. pneumoniae*, the neuraminidase is known to cleave the terminal sialic acids of host polysaccharides [29] and promotes the formation of biofilm [30]. It was also interesting to locate several family 1 and 2 glycosyltransferase genes (GT1: C1330, C1367, GT2: C1459) within the top rankings of several immune evasins training sets (*cspA*, *cps* and *neu* clusters); as many of the *cps* genes encode glycosyltransferase enzymes [31], these highly prioritized genes may play a role in the biosynthesis of unrecognized carbohydrate structures contributing to the antigenic diversity of GBS. This finding is in concordance with a study which suggested that C1330 (SAG1410 in 2603V/R) encodes an *α*-galactosyltransferase participating in group B carbohydrate synthesis [32]. In addition, a gene encoding putative staphylokinase homolog C1080 (SAG1127 and GBS1195) was found. Staphylokinase is known to cleave the Fc portion of human IgG and complement C3b [33] and to inactivate *α*-defensin produced by neutrophils during *S. aureus* infection [34].

### Corroborated discovery of virulence genes using functionally unrelated virulence genes as a training set

**Figure 3:**
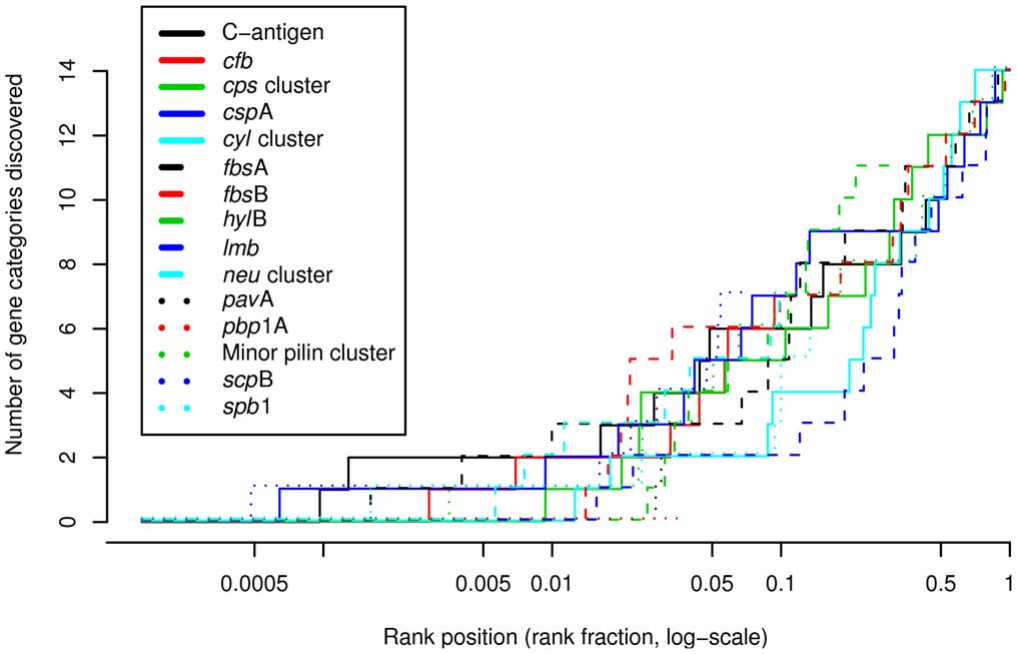
Number of other gene categories discoverable at a certain rank position. This analysis evaluated how many virulence gene categories are discoverable at a given position of a prioritized rank. A category is considered discoverable by another if at least one virulence gene is present above a given position in the rank is being analyzed. The gene positions were measured by rank fraction (between 0 and 1) with 0 being the top of the rank and 1 at the bottom. Candidate genes were ranked by SVM/RBF algorithm (the best algorithm evaluated in Table 2).

Because bacterial pathogenesis is mediated by a variety of distinct molecular mechanisms, a good gene prioritization model would be expected to identify different classes of virulence genes from which the predictive model can be built. To estimate the predictive power of such “cross-group” discoveries, we examined the rankings of known GBS virulence genes in each the 15 lists produced by ICGP. It was noted that, on average, at least one gene from a functional category can be discovered in the top 1% of a gene rank produced by the SVM/RBF algorithm (the best performing algorithm in the first rediscovery experiment, Table 2) that is trained on genes of another virulence category. A median of 4 other categories (out of total of 15) was discoverable within the top 5% of a given rank. The cumulative gain plot depicting this phenomenon is shown in Figure 3.

**Figure 4:**
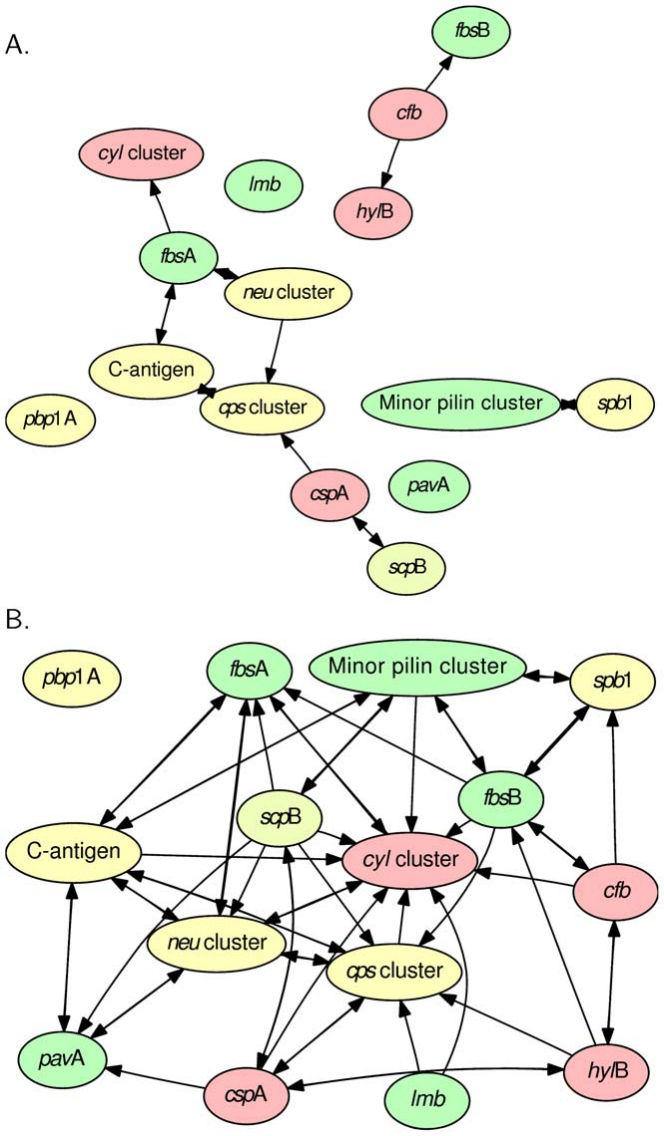
Inter-discovery between virulence gene categories. These figures provide two cross-sectional views of Figure 3 at the positions of top-1% (A) and -5% (B) respectively. The arrowheads indicates which other categories of virulence genes were discoverable by the category at the tail of arrow.

Several qualitative observations were made during the cross-group analysis which supported the plausibility of the prioritized gene lists. For example, at least one gene from the categories of GBS surface C-antigens (including all *bca*, *rib* and *bac* genes), *cps*, and *neu* clusters were discoverable within the top-1% of the ranks of the other two functional categories (approximately 21 genes including training set genes, Figure 4A). At top 5% (approximately 104 genes including the training set) of the ranks, all but one (*pbp1A*) gene category can either be used to discover through, or at least have one gene being discovered by, another category (Figure 4B). While we did not identify apparent directions of discovery between the major virulence function classes (adhesins, invasins, and evasins), these qualitative observations (of the majority of known GBS virulence genes placed on the top of the prioritized lists of other functional categories) reconfirmed the capacity of our method to identify genes with potential impact on virulence within the set of remaining highly-ranked but functionally unrecognized genes.

### Highly-ranked genes are not linked with the genes in training sets

**Figure 5:**
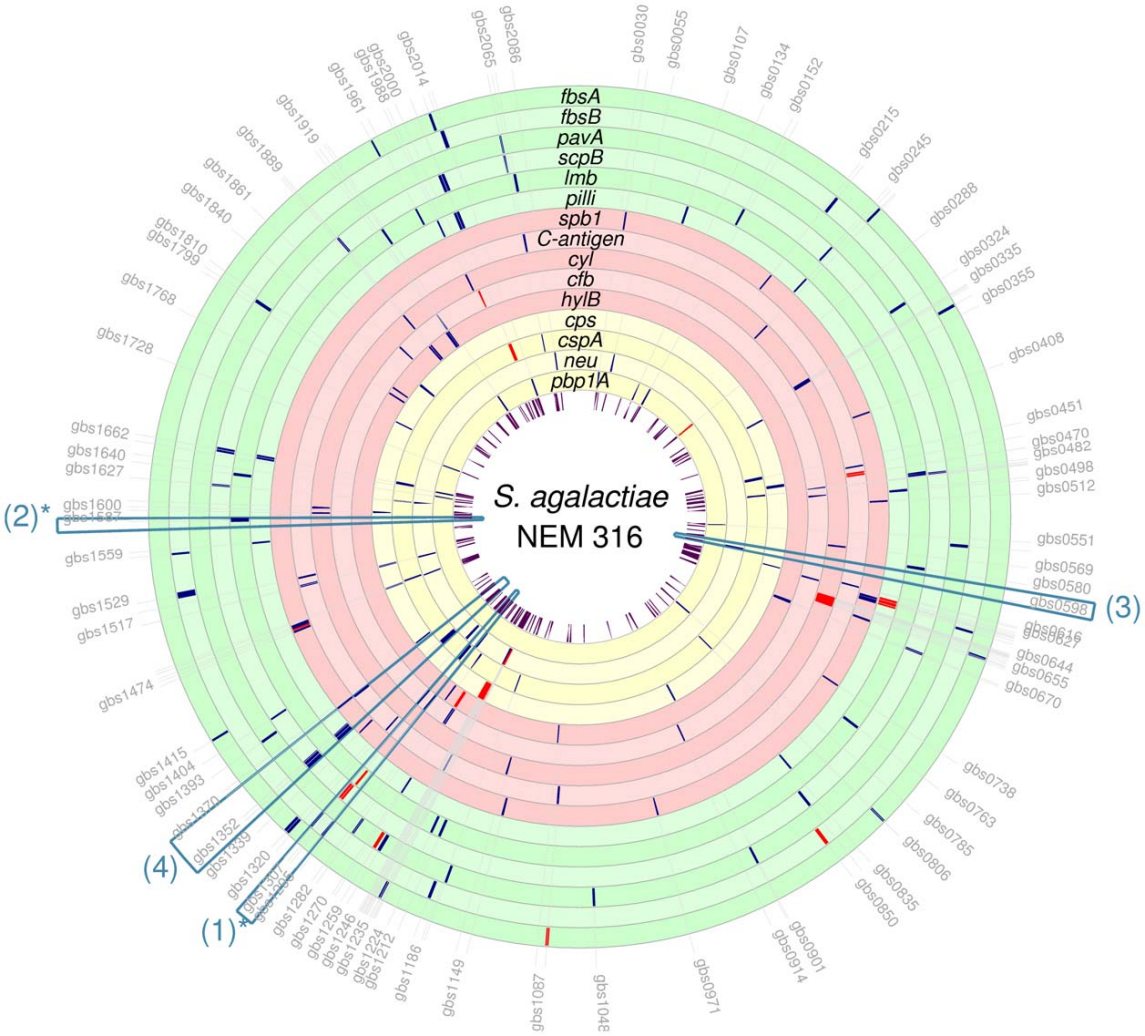
Positions of the training set (in red) and top-10 genes (in blue) in each of the 15 virulence gene categories in *S. agalactiae* NEM316 genome (serotype III). The highly-ranked genes is shown to be scattered across the entire GBS genome and not aggregated in close physical proximity. Physical linkages between the known and the prioritized genes are therefore unlikely. This illustration demonstrated the novelty of the PP approach for virulence gene discovery compared with the traditional paradigm of physical linkage and gene clusters. The blue boxes refer to the known genomic islands and are discussed in the results section. (*) Predicted by homology to other reference genomes, as islands (1) and (2) were not listed in PAI-DB or IslandViewer for NEM316.

To demonstrate that the virulence genes predicted by ICGP do not merely discover neighboring genes, highly-ranked genes in the NEM316 genome were plotted on the chromosome map (Figure 5). It is evident that the newly discovered genes were scattered across the genome. Comparing the average distance between start codons of neighboring genes (mean: 1,056 bp, 95% confidence interval: 1,018–1,093 bp), the average distance between the highly-ranked genes and the closest gene of the corresponding training set was 544,441 bp with a wide range (95% CI: 495,802–593,080 bp), which indicates a clear difference in placement of predicted virulence genes discovered by the ICGP method (two-sample unpaired *t*-test, t = 22.1, df = 157, 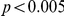).

### Highly-ranked genes can reside within known or predicted genomic islands

Several top-ranked genes are located within known genomic islands in two or more reference genomes: (1) The gene IS*Sag2* (C1177), encoding a transposase, was placed within the top-10 on the *fbsB* rank; IS*Sag2* transposase flanks a 17-kbp composite transposon found in virtually all GBS strains [35] and characterizes a pathogenicity island (PI) containing virulence genes *scpB* and *lmb* (2603V/R: SAG1228-44, A909: SAK1314-23). (2) Genes *mtsA/C* (C1443 and C1445) were discovered in another known PI (2603V/R: SAG1527-33, A909: SAK1550-6). (3) Genes *vex1-3* and *vncR/S* were located within the genomic island located at GBS0587-600 (2603V/R: SAG0608-20, A909: SAK0692-705). While the exact functions of the *vex*-*vnc* clusters remain to be elucidated, it has been demonstrated that mutants lacking of *vex3* are associated with altered resistance of *S. pneumoniae* to vancomycin [36], [37]. (4) Prioritized in the C-*α*/*β* rank, a putative type II DNA modification methyltransferase (C1224) was found on the predicted genomic island containing the *bac* gene, the gene encoding surface protein C-*β* antigen, which was bound by GBS1350 and GBS1371 in NEM316 (2603V/R: SAG1287-97; A909: SAK0720-64).

### The rediscovery of virulence genes in *Streptococcus pneumoniae*

To demonstrate that our approach is generalizable to other species, the rediscovery experiments were replicated on 6,355 genes in 3 published *S. pneumoniae* genomes (D39, R6, and TIGR4). Forty-seven known pneumococcal virulence genes were arranged into 21 virulence gene groups through the review of literature [38]: choline-binding protein genes (*cpbC-G*), capsular polysaccharide gene cluster (*cps*), serine protease gene *htrA*, hyaluronidase gene *hysA*, IgA protease gene *iga1*, autolysin genes (*lytABC*), neuraminidase genes (*nanAB*), adhesin and ABC transporter genes (*pavA*, *piaA*, and *piuA*), pneumolysin gene *ply*, a manganese-binding ABC transporter gene (*psaA*), peptidylprolyl isomerase genes (*ppmA* and *slrA*), and a zinc-metalloprotease gene *zmpB*. It was found that: (1) Within the top-0.5% of the *cbpA-G*, *lytABC*, *nanAB*, *iga1*, *zmpB*, and *hysA* ranks, at least one other gene from the other virulence gene groups was able to be identified. (2) The *de novo* gene lists (of top-0.5% of the prioritized genes) have also revealed genes suggestive of virulence functions in *S. pneumoniae*: putative helicase genes spr0500-3 (in the *hysA*, *iga1* and *nanAB* ranks), ferric-iron permease *fatC* (in *piaA*, *piuA*, *lytB*, and *cbpB* ranks), *murM* and exfoliative toxin *shetA* genes (in *cbpD* and *hysA* ranks), pyrorolidone-carboxylate peptidase gene (*pcp*, in *lytC* and *hysA* ranks), alpha-galactosidase gene *aga* (with *nanAB*), a Hes/MoeB/ThiF family gene (in the *cbpE* and *lytB* ranks), as well as surface proteins spr0583 and pcpA, galactose-1-phosphate uridylyltransferase genes *gatT*, and hypothetical protein spr0217 (in *pspC*, *cbpEG*, and *lytAC* ranks) were revealed. ICGP has also suggested a hemolysin-related protein gene (spr0737) which was found to be closely associated with *ply*. The unsaturated glucuronyl hydrolase gene (*ugl*) was ranked highly with *hysA*, and *psaA* was associated with laminin-binding protein gene *lmb* and ABC transporter genes *adcA*, *psaC*, *adbC*, and *appA*. These encouraging results have thus supported the use of ICGP in virulence gene prediction in other pathogens.

## Discussion

This paper demonstrates a new approach to discover potential virulence genes in bacterial genomes. It describes a computational pipeline using phylogenetic profiles to identify new virulence genes in *S. agalactiae*. Fifteen genes, for which there is evidence of either confirmed or plausible associations with virulence in other bacterial pathogens have been identified. Many of these genes are involved in epithelial adhesion, damaging to host cells, or evasion of the host immune system (Figure 2). While most of these genes are considered “general” virulence factors, it is likely that some of them may play an unique role in the pathogenesis of GBS infection in susceptible newborns. Determining the optimal cut-off of the gene rank was, however, challenging because it was not possible to estimate the number of virulence genes in a genome in advance. While other criteria for determining the significance level may be imposed, for example, inverse of the number of genes in the target given genome [10], obtaining an objective score for a generative classification model is less trivial (discussed below). We adopted a more practical approach by reviewing the top-10 genes (approximately 0.5% of the GBS genome) of the ranks from each functional category to examine their potential biological roles. Although the selection of this significance level seemed arbitrary and true virulence genes may have lower ranks, our results have demonstrated that, by using this threshold (top-10 genes), the probability of finding a true virulence gene could be improved by up to 10 times compared with random selection of candidate genes. Thus, our objective of postulating new GBS candidate virulence genes has been fulfilled; this is also evident through qualitative analysis of evidence retrieved from the published literature and databases.

Our *in silico* gene ranking approach offers a new opportunity to perform a genome-wide identification of virulence genes in bacterial pathogens. The functional validity of this approach was also strengthened by, for instance, the ability to recover 6 out of 10 known peptidoglycan genes with the PP of penicillin binding protein gene, *pbp1A*. These results support our original hypothesis that a group of virulence genes with closely-related mechanisms can be widely distributed across bacterial genomes. Thus, the concept of virulence gene-infectious disease relationship may be modified from one that involves a simple association between a gene and a pathogen trait, where virulence is related to the presence or absence of incriminated genes, to a complex repertoire of widely distributed genes that confer specific survival advantage on the pathogen. The good prediction results from our rediscovery experiments imply that there are specific combination patterns of virulence genes in bacterial pathogens. The existence of such patterns is conceivable, because the co-occurrence of virulence genes is a fundamental requirement for pathogen function and interaction with the host at the cellular level [39]. However, the interpretation and comprehension of these implicit patterns is challenging. Bowers et al. (2004), for example, analyzed gene co-occurrence patterns to find higher-order inter-relationships between genes [40]. The integration of PP-based gene prioritization methods with other data sources should be explored. For example, mapping PP signatures to gene ontology and annotation databases, to decipher the underlying meaning of these highly-conserved profiles, can be of value.

There are several points to note in the selection of training data and algorithms. First, we based our *de novo* predictions on the individual categories of virulence function as opposed to a training set consisting of all known virulence factors. Although novel genes may be revealed by training the ICGP models with the aggregated training set, the categorized approach can be justified because results are likely to be skewed towards gene functions presented with higher proportion in the training set (see Text S1). It is also evident that training sets with higher functional consistency at molecular level have better cross-validation results. For example, the category of *neu* cluster is more consistent over the broader category of immune system evasins. Second, we selected ICGP algorithms based on the results of our previous work, which showed that the discriminative classifiers outperformed the generative model of naïve Bayes in a set of standard prioritization tasks [17]. One disadvantage of using a discriminative model is that the classifier outputs do not generally correspond to a true probability distribution of gene-function relationships. Although attempts were made to rectify the probability estimates for models such as SVM (i.e., fitting logistic models to output and aggregating individual rankings by voting), the distribution of scores still depends on individual algorithms. This may also explain the disparity of good rediscovery performances achieved by most algorithms (Table 2) and poor agreements between individual gene rankings (Text S2). Thirdly, our approach only aims to recover the genes having similar phylogenetic profile to the known virulence factors. In cases where no virulence genes are known, alternative methods would need to be sought for the gene prioritization task.

In conclusion, we have performed a computational genome-wide prioritization for discovering potential virulence genes in *S. agalactiae* through a cross-genomic analysis of PPs. Our comparative genomic approach requires fewer genomes of the target virulence species for hypothesizing potential virulence genes. A number of plausible molecular mechanisms have been revealed, some of which have been documented in other bacterial pathogens. Furthermore, we have significantly extended the number of potential bacterial gene targets for drug and vaccine design by identifying highly-ranked yet uncharacterized candidate genes which may have roles in GBS virulence. This approach can also be applicable to the discovery of virulence genes in other bacterial pathogens.

## Materials and Methods

### Data sources

The phylogenetic profiles of the whole genome of three strains of *S. agalactiae* A909 [41], NEM316 [42], and 2603V/R [9] were determined by searching the occurrence of 6,214 genes in 467 annotated bacterial genomes retrieved from National Center for Biotechnology Information database (NCBI, ftp://ftp.ncbi.nlm.nih.gov/genomes/Bacteria/; downloaded in April 2007) by using Basic Local Alignment and Search Tool (BLAST) algorithm (blastp program). The presence of a potential homologous gene was determined at the critical E-value of 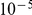 (Dataset S1). For each known GBS virulence factor, a further literature search was performed and the location of associated genes identified and labeled in the reference genomes (see Text S3 for more details). The criteria for grouping of the known virulence factors into 15 functional categories were: discriminable by BLAST and a distinguishable biological mechanism in GBS pathogenesis.

Descriptive analysis of the PPs revealed that 527 of 6,124 genes (8.5%) were specific to GBS (present in at least 1 of the 3 genomes), including 4.2% of genes specific to individual GBS reference strains. Four hundred and seventy seven genes (7.7%) were present in >95% of reference genomes. Overall, the 467-genome panel was able to characterize GBS genes into large numbers of genotypes in 2603V/R (1,712 types), NEM316(1,675 types), and A909 (1,689 types) genomes respectively. This is equivalent to approximately 80–85% of unique genotypes when compared to the number of genes per GBS genome, indicating that our PP panel can be used to characterize individual genes with satisfactory discriminatory power. The inclusion of multiple genomes per species may have introduced redundancy, as all NCBI genomes were used as the reference panel. However, it has been previously shown that redundancy did not result in performance penalties in machine learning-based gene prioritization methods [17] and hence a more inclusive approach was adopted in the selection of reference genomes.

### Machine learning algorithms

Four machine learning algorithms were applied to each of the functional categories of known GBS genes. Algorithm selection was based on performance in our previous work [17]. The algorithms include: support vector machine with linear kernel (SVM/Poly, trained by sequential minimization optimization algorithm), SVM with RBF kernels (SVM/RBF), alternating decision tree (ADTree with number of boosting iterations set to 10), and *k*-nearest neighbor classifier (IBk with inverse distance weighing where *k* was determined by leave-one-out cross-validation). The output of each classifier was used for the basis for gene ranking. Logistic models were fitted to estimate the posterior probabilities of both SVM algorithms. Algorithms were implemented using Waikato Environment for Knowledge Analysis (WEKA) version 3.5.6 [43].

### Rediscovery of the training genes

For each functional GBS gene category containing *n* virulence genes, a *n*-fold cross-validation was performed, with the remaining candidate genes assigned a negative class. Rediscovery performance was measured by AUC for each combination of algorithm and gene category. All genes in NEM316 genome were used for cross-validation in the first rediscovery experiment, and all genes from the 3 reference genomes were applied in the second experiment.

### Sub-sampling of negative examples in the *de novo* discovery of GBS virulence genes

For each functional category, all of known virulence genes were labeled as positive gene examples in the training set. To reduce the oversampling of negative classes, only a subset of the remaining unlabeled genes were labeled as negative examples in the training set. The remaining 3/4 of candidate genes were randomly sampled without replacement and were assigned a negative class. Predictions were made on the remaining one-quarter of the unknown genes and scores from each run were obtained for each gene to be predicted. The above procedure was repeated for 1000 runs to improve coverage. Scores from each run were averaged by arithmetic means which formed the basis of ranking. This procedure is detailed in Text S3.

### Combining the ranks from multiple models

To increase the likelihood of identifying true virulence genes, we aggregated ranks produced by 4 machine learning algorithms into a final rank by using the following voting function:
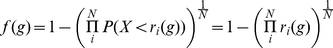
where *g* is a candidate gene, 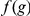 is the final aggregated score of gene 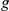, 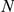 is number of ranks (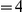), X is an uniform random variable, and 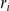 is the rank fraction (position of the rank, starting from 1, divided by the total number of genes in the entire list) of rank 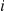.

### Clustering of homologous genes

Because homologous (including both orthologs and closely-related paralogs) genes would appear multiple times in close proximity in a prioritized rank due to high degrees of similarities in PPs, the genes from each resultant rank were collated into homolog clusters to ease the interpretation of results. The reciprocal best BLAST hit method described by Hirsh et al. was employed [44]. The complete list of homolog clusters is shown in Table S2.

### Identification of genomic islands

The participation of genes in genomic islands was examined by search against the IslandViewer database [45] and PAthogenicity Island DataBase (PAI-DB) [46].

## Supporting Information

Dataset S1 The phylogenetic profiles of all 6,214 genes of 3 GBS genomes (NEM316, A909/Ia, and 2603V/R) used in this paper.
(TXT)

Table S1 Top-10 genes of each virulence function category prioritized by inductive CGP.
(DOC)

Table S2 List of homolog clusters in the three *S. agalactiae* genomes defined in this paper.
(PDF)

Text S1 Prioritization of candidate virulence genes in the GBS genomes by using all known virulence factors as training set.
(DOC)

Text S2 Correlations between prioritized gene lists produced by different machine learning algorithms.
(DOC)

Text S3 Additional materials and methods.
(DOC)
