# Supplementary material for: Computational Bacterial Genome-Wide Analysis of Phylogenetic Profiles Reveals Potential Virulence Genes of Streptococcus agalactiae
Source: PLoS One. 2011 Apr 4;6(4):e17964. doi: 10.1371/journal.pone.0017964 (PMC3070697; doi:10.1371/journal.pone.0017964)
Supplement: Table S1 — Top-10 genes of each virulence function category prioritized by inductive CGP. (DOC) [file pone.0017964.s002.doc]

**Supporting Information - Table S1. Top-10 genes of each virulence function category prioritized by inductive CGP**

| **Rank** | **Cluster** | **Score** | **Gene product / function** | **Systematic names in ref. genomes** |
| --- | --- | --- | --- | --- |
| **Adhesins** | | | | |
| **a) *fbs*A rank** | | | | |
| 1 | C0348/S | 0.9932 | hypothetical protein | SAG0357, SAK0431, GBS0344 |
| 2 | C1924/R | 0.9914 | patatin-like phospholipase family protein | SAG2059, SAK1997, GBS2014 |
| 3 | C1856/S | 0.9910 | hypothetical protein | SAG1975, SAK1935, GBS1961 |
| 4* | C0642/GC | 0.9897 | [*cyl*J] cylJ protein | SAG0672, SAK0800, GBS0654 |
| 5† | C1977/S | 0.9896 | hypothetical protein | SAG2119 |
| 6* | C1115 | 0.9881 | [*cps*K] capsular polysaccharide synth. protein CpsK | SAG1163, SAK1252, GBS1237 |
| 7 | C0753/QR | 0.9867 | hypothetical protein | SAG0786, SAK0911, GBS0806 |
| 8 | C0255 | 0.9867 | hypothetical protein | SAG0263, SAK0335, GBS0253 |
| 9 | C1271/S | 0.9864 | hypothetical protein | SAG1345, SAK1376, GBS1415 |
| 10† | C2124 | 0.9863 | type IIG restriction enzyme and methyltransferase | SAK1333, GBS1324 |
| **b) *fbs*B rank** | | | | |
| 1 | C1927 | 0.9991 | pathogenicity protein, putative | SAG2063, SAK2002, GBS2018 |
| 2 | C0222/D | 0.9982 | [*pre*] plasmid recombination enzyme | SAG0226, SAK0286, GBS0219 |
| 3 | C1080 | 0.9980 | hypothetical protein | SAG1127, GBS1195 |
| 4 | C2178 | 0.9979 | hypothetical protein | SAK2093 |
| 5 | C1377 | 0.9965 | cell wall surface anchor family protein | SAG1462, SAK1493, GBS1529 |
| 6 | C2043/G | 0.9956 | PTS system, galactitol-specific IIB component, putative | SAK0525 |
| 7 | C0623/M | 0.9955 | Cna protein B-type domain | SAG0651, SAK0782, GBS0636 |
| 8 | C1412 | 0.9952 | hypothetical protein | SAG1498, SAK1524, GBS1559 |
| 9 | C2045/G | 0.9944 | PTS system, galactitol-specific IIB component, putative | SAK0530 |
| 10† | C1177/L | 0.9944 | IS*Sdy*1/IS*Sag2*, transposase OrfA | SAG1228/1243, SAK1314/1322, GBS1300/1310 |
| **c) *pav*A gene rank** | | | | |
| 1 | C0498/R | 0.9985 | HD domain protein | SAG0512, SAK0662, GBS0558 |
| 2 | C1161/R | 0.9978 | [*rnz*] ribonuclease Z | SAG1210, SAK1296, GBS1282 |
| 3 | C1137/R | 0.9940 | metallo-β-lactamase superfamily protein | SAG1186, SAK1273, GBS1259 |
| 4 | C1072/E | 0.9939 | [*thr*B] homoserine kinase | SAG1119, SAK1204, GBS1186 |
| 5 | C1659/R | 0.9934 | metallo-β-lactamase superfamily protein | SAG1761, SAK1783, GBS1804 |
| 6 | C0850 | 0.9933 | conserved hypothetical protein TIGR00159 | SAG0885, SAK1008, GBS0902 |
| 7 | C0972/R | 0.9932 | GTP-binding protein | SAG1013, SAK1108, GBS1048 |
| 8 | C1249/C | 0.9931 | [*fni*] isopentenyl pyrophosphate isomerase | SAG1323, SAK1354, GBS1393 |
| 9 | C0327/L | 0.9925 | [*com*FA] competence protein ComFA, putative | SAG0336, SAK0406, GBS0324 |
| 10† | C1194/K | 0.9923 | [*cad*C] cadmium resistance accessory protein CadX | SAG1258, SAK2052, GBS2065 |
| **d) *scp*B gene rank** | | | | |
| 1*† | C0646/O | 0.9995 | [*csp*A] cell surface serine endopeptidase CspA | SAG0676/2053, SAK0804/1991, GBS2008 |
| 2† | C1483/R | 0.9952 | AcuB family protein | SAG1577, SAK1593, GBS1627 |
| 3† | C0664/G | 0.9903 | carbohydrate kinase, PfkB family | SAG0697/1906, SAK0823,GBS0670/1893 |
| 4† | C2042/QR | 0.9897 | hypothetical protein | SAK0522, GBS0486 |
| 5† | C1194/K | 0.9887 | [*cad*C] cadmium resistance accessory protein CadX | SAG1258, SAK2052, GBS2065 |
| 6† | C1211/KL | 0.9887 | SNF2 family protein | SAG1280/1618, SAK1633, GBS1352/1353/1666 |
| 7 | C1260 | 0.9873 | [*def*] peptide deformylase | SAG1334, SAK1365, GBS1404 |
| 8 | C0790 | 0.9873 | polysaccharide deacetylase family protein | SAG0824, SAK0948, GBS0842 |
| 9 | C0245/J | 0.9871 | acetyltransferase, GNAT family | SAG0252, SAK0327, GBS0245 |
| 10 | C2136/R | 0.9869 | phenazine biosynthesis protein, PhzF family | SAK1767 |
| **e) *lmb* gene rank** | | | | |
| 1 | C0520/RP | 0.9991 | [*adc*A] ABC transporter, Zn-binding adhesion lipoprotein | SAG0535, SAK0685, GBS0580 |
| 2 | C1443/P | 0.9982 | [*mts*C] manganese ABC transporter, permease protein | SAG1531, SAK1554, GBS1587 |
| 3 | C1821/P | 0.9982 | laminin-binding surface protein | SAG1938, SAK1898, GBS1926 |
| 4 | C0154/P | 0.9978 | [*adc*B] zinc ABC transporter, permease protein | SAG0156, SAK0219, GBS0152 |
| 5 | C1445/P | 0.9970 | [*mts*A] manganese ABC transporter, manganese-binding adhesion liprotein | SAG1533, SAK1556, GBS1589 |
| 6 | C1497/P | 0.9968 | K+ transporter (Trk) family protein TrkH, putative | SAG1591, SAK1606, GBS1640 |
| 7 | C0709 | 0.9956 | peptidase, U32 (collagenase) family | SAG0742, SAK0868, GBS0763 |
| 8† | C2042/QR | 0.9930 | hypothetical protein | SAK0522, GBS0486 |
| 9 | C0880/L | 0.9916 | prophage LambdaSa2, site-specific recombinase, phage integrase family | SAG0915/1885/1986/1993, SAK1943/2059/2094, GBS0482/1224/1969/2073 |
| 10 | C1097/E | 0.9907 | sodium:alanine symporter family protein | SAG1145, SAK1231, GBS1212 |
| **f) GBS pilus gene cluster rank** | | | | |
| 1*† | C0646/O | 0.9961 | [*csp*A] cell surface serine endopeptidase CspA | SAG0676/2053, SAK0804/1991, GBS2008 |
| 2 | C1419/LR | 0.9943 | MutT/nudix family protein | SAG1505, SAK1529, GBS1564 |
| 3 | C2103 | 0.9939 | prophage LambdaSa04, holin | SAK0760 |
| 4 | C0106/R | 0.9935 | hypothetical protein | SAG0108, SAK0158, GBS0107 |
| 5† | C1211/KL | 0.9932 | SNF2 family protein | SAG1280/1618, SAK1633, GBS1352/1353/1666 |
| 6 | C0249/K | 0.9925 | RNA polymerase sigma factor, ECF subfamily | SAG0256, SAK0331, GBS0249 |
| 7 | C1146/LR | 0.9917 | MutT/nudix family protein | SAG1195, SAK1282, GBS1268 |
| 8 | C1901 | 0.9909 | hypothetical protein | SAG2030, SAK1968, GBS1988 |
| 9 | C0442/T | 0.9906 | hypothetical protein | SAG0455, SAK0556, GBS0502 |
| 10 | C0784/S | 0.9897 | probable proton-coupled thiamine transporter YuaJ | SAG0817, SAK0940, GBS0835 |
| **Invasins** | | | | |
| **g) *spb*1 gene rank** | | | | |
| 1 | C2129/M | 0.9994 | cna B-type domain protein | SAK1441 |
| 2† | C2046/G | 0.9971 | α-galactosidase, putative | SAK0535 |
| 3 | C0651/V | 0.9967 | [*vex*3] ABC transporter, permease protein Vexp3 | SAG0615/0682, SAK0700/0810, GBS0657 |
| 4* | C0619/M | 0.9961 | GBS52 (minor pilin cluster) | SAG0646/1404, SAK0777, GBS0629/1474 |
| 5 | C1191 | 0.9953 | Tn5252, Orf 9 protein | SAG1251, GBS1339 |
| 6 | C0036 | 0.9950 | hypothetical protein | SAG0037, SAK0070, GBS0036 |
| 7 | C0218 | 0.9948 | replication initiation factor, RepA family | SAG0222/1299, SAK0282, GBS0215/0408/0738/0971/1149/1372 |
| 8* | C0622/M | 0.9942 | GBS104 (minor pilin cluster) | SAG0649/1408, SAK0780, GBS0632/1478 |
| 9 | C0451 | 0.9932 | hypothetical protein | SAG0465, SAK0567, GBS0512 |
| 10 | C2044/G | 0.9923 | [*rha*D] rhamnulose-1-phosphate aldolase | SAK0527 |
| **h) C-α family/C-β protein gene rank** | | | | |
| 1* | C1007 | 0.9974 | [*fbs*A] fibrinogen-binding protein | SAG1052, SAK1142, GBS1087 |
| 2 | C0613/S | 0.9945 | hypothetical protein | SAG0636/2111,SAK0719/0769, GBS0616 |
| 3 | C0435/L | 0.9939 | transposase, IS*256* family | SAG0448 |
| 4† | C2061/KL | 0.9937 | prophage LambdaSa03, helicase, putative | SAK0617 |
| 5 | C1224/L | 0.9937 | prophage LambdaSa2, type II DNA modification methyltransferase, putative | SAG1297/1869, SAK0739, GBS1370 |
| 6 | C0413/R | 0.9919 | pyridoxamine 5'-phosphate oxidase family | SAG0422, SAK0503, GBS0457 |
| 7 | C0897/D | 0.9913 | Tn916, FtsK/SpoIIIE family protein | SAG0933, SAK2056,GBS1320/2069 |
| 8* | C1117/M | 0.9901 | [*cps*O] capsular polysaccharide biosythesis protein CpsI | SAG1165/1166/1455, SAK1254/1488, GBS1239/1524 |
| 9 | C0585/M | 0.9888 | prophage LambdaSa03, peptidoglycan endolysin | SAG0604, SAK0653 |
| 10† | C1367/M | 0.9874 | glycosyltransferase, group 1 family protein | SAG1448, SAK1481, GBS1517 |
| **i) *cyl* gene cluster rank** | | | | |
| 1 | C0342/I | 0.9960 | [*fab*Z] (3R)-hydroxymyristoyl ACP dehydratase | SAG0351, SAK0425, GBS0338 |
| 2 | C0340/IQ | 0.9955 | [*fab*F] 3-oxoacyl-(acyl carrier protein) synthase | SAG0349, SAK0423, GBS0336 |
| 3 | C0508/IQR | 0.9945 | acetoin reductase | SAG0523, SAK0674, GBS0569 |
| 4 | C0339/IQR | 0.9945 | [*fab*G] 3-ketoacyl-(acyl-carrier-protein) reductase | SAG0348/1904, SAK0422, GBS0335/1891 |
| 5 | C0338/I | 0.9908 | [*fab*D] acyl-carrier-protein *S*-malonyltransferase | SAG0347, SAK0421, GBS0334 |
| 6 | C1044/R | 0.9864 | oxidoreductase, short chain dehydrogenase family | SAG1091, SAK1176, GBS1158 |
| 7 | C1462/S | 0.9845 | hypothetical protein | SAG1554, SAK1573, GBS1608 |
| 8 | C1455/IQR | 0.9818 | [*fab*G] 3-ketoacyl-(acyl-carrier-protein) reductase | SAG1544, SAK1566, GBS1600 |
| 9 | C1860 | 0.9811 | hypothetical protein | SAG1979/2034, SAK1974, GBS1992 |
| 10 | C0257 | 0.9811 | hypothetical protein | SAG0265, SAK0337, GBS0255 |
| **j) *cfb* gene rank** | | | | |
| 1 | C0538/K | 0.9988 | prophage LambdaSa1, antirepressor, putative | SAG0555 |
| 2 | C0560 | 0.9983 | hypothetical protein | SAG0577, SAK0625 |
| 3 | C1716/S | 0.9982 | hypothetical protein | SAG1820, SAK1840, GBS1861 |
| 4 | C0429 | 0.9978 | hypothetical protein | SAG0441, SAK0544, GBS0488 |
| 5 | C0617 | 0.9970 | transcriptional regulator, AraC family | SAG0644, SAK0775, GBS0627 |
| 6 | C0862 | 0.9959 | CRISPR-associated SAG0897 family protein | SAG0897, SAK1020, GBS0914 |
| 7 | C0438 | 0.9958 | bacteriocin transport accessory protein, putative | SAG0451, SAK0553, GBS0498 |
| 8* | C1148 | 0.9954 | [*hyl*B] hyaluronate lyase | SAG1197, SAK1284, GBS1270 |
| 9† | C1819/GT | 0.9949 | PTS system, galactitol-specific IIA component, putative | SAG1935, SAK0524/0528/1895, GBS1922 |
| 10† | C1177/L | 0.9939 | IS*Sdy*1, transposase OrfA | SAG1228/1243, SAK1314/1322, GBS1300/1310 |
| **k) *hyl*B gene rank** | | | | |
| 1 | C1789/R | 0.9992 | [*ugl*] glucuronyl hydrolase | SAG1901, GBS1889 |
| 2 | C1665/R | 0.9958 | 5'-nucleotidase, lipoprotein e(P4) family | SAG1767, SAK1789, GBS1810 |
| 3 | C1816/G | 0.9951 | neuraminidase-related protein | SAG1932, SAK1891/1892, GBS1919 |
| 4† | C1819/GT | 0.9922 | PTS system, galactitol-specific IIA component, putative | SAG1935, SAK0524/0528/1895, GBS1922 |
| 5† | C2046/G | 0.9897 | α-galactosidase, putative | SAK0535 |
| 6 | C0665/G | 0.9893 | β-glucuronidase | SAG0698, SAK0824, GBS0671 |
| 7 | C1166/P | 0.9888 | exfoliative toxin A, putative | SAG1215, SAK1301, GBS1287 |
| 8 | C1014/E | 0.9882 | glycine cleavage system H protein, putative | SAG1059, SAK1148, GBS1093 |
| 9† | C0664/G | 0.9846 | carbohydrate kinase, PfkB family | SAG0697/1906, SAK0823, GBS0670/1893 |
| 10 | C1654/E | 0.9819 | [*lta*E] low specificity L-threonine aldolase | SAG1756, SAK1778, GBS1799 |
| **Immune evasins** | | | | |
| **l) *cps* gene cluster rank** | | | | |
| 1† | C1330/M | 0.9951 | glycosyl transferase, group 1 family protein | SAG1410, SAK1445, GBS1480 |
| 2 | C1622/U | 0.9930 | [*lep*B] signal peptidase I | SAG1723, SAK1443/1731, GBS1768 |
| 3† | C1367/M | 0.9928 | glycosyl transferase, group 1 family protein | SAG1448, SAK1481, GBS1517 |
| 4 | C0359/K | 0.9926 | transcriptional regulator, putative | SAG0368, SAK0442, GBS0355 |
| 5 | C0849/M | 0.9924 | *mur* ligase family protein | SAG0884, SAK1007, GBS0901 |
| 6* | C0424/D | 0.9910 | [*bac*] cell wall surface anchor family protein, truncation | SAG0433, SAK0186/0517/0722/0771, GBS0470/0619 |
| 7 | C1695/G | 0.9884 | [*xfp*] putative phosphoketolase | SAG1799, SAK1819, GBS1840 |
| 8 | C1231/E | 0.9876 | [*mmu*M] homocysteine methyltransferase | SAG1305, SAK1337, GBS1377 |
| 9 | C1459/M | 0.9876 | glycosyl transferase, group 2 family protein | SAG1548/1551, SAK1570, GBS1605 |
| 10 | C1193/L | 0.9874 | transposase, IS*L*3 family | SAG1253 |
| **m) *neu* gene cluster rank** | | | | |
| 1† | C1977/S | 0.9986 | hypothetical protein | SAG2119 |
| 2 | C1985/T | 0.9952 | sensor histidine kinase, putative | SAG2127, SAK2066, GBS2086 |
| 3† | C1483/R | 0.9943 | AcuB family protein | SAG1577, SAK1593, GBS1627 |
| 4 | C0596/T | 0.9938 | [*vnc*S] sensor histidine kinase VncS, putative | SAG0617, SAK0188/0702, GBS0598 |
| 5† | C1367/M | 0.9920 | glycosyl transferase, group 1 family protein | SAG1448, SAK1481, GBS1517 |
| 6 | C0054/E | 0.9908 | [*thr*C] threonine synthase | SAG0055, SAK0088, GBS0055 |
| 7 | C1172/S | 0.9889 | hypothetical protein | SAG1223, SAK1309, GBS1295 |
| 8† | C1330/M | 0.9887 | glycosyl transferase, group 1 family protein | SAG1410, SAK1445, GBS1480 |
| 9* | C1114/R | 0.9875 | [*cps*L] capsular polysaccharide repeat unit transporter | SAG1162, SAK1251, GBS1237 |
| 10† | C2124 | 0.9855 | type IIG restriction enzyme and methyltransferase | SAK1333, GBS1324 |
| **n) *csp*A gene rank** | | | | |
| 1* | C0407/O | 0.9995 | [*scp*B] streptococcal C5a peptidase | SAG0416, SAK1320, GBS0451/1308 |
| 2† | C1194/K | 0.9983 | [*cad*C] cadmium resistance accessory protein CadX | SAG1258, SAK2052, GBS2065 |
| 3† | C1483/R | 0.9914 | AcuB family protein | SAG1577, SAK1593, GBS1627 |
| 4† | C1211/KL | 0.9909 | SNF2 family protein | SAG1280/1618, SAK1633, GBS1352/1353/1666 |
| 5† | C2061/KL | 0.9905 | prophage LambdaSa03, helicase, putative | SAK0617 |
| 6 | C0753/QR | 0.9900 | hypothetical protein | SAG0786, SAK0911, GBS0806 |
| 7 | C0430/J | 0.9886 | acetyltransferase, GNAT family | SAG0442/0443, SAK0545, GBS0489/0490 |
| 8 | C1332/R | 0.9840 | polysaccharide biosynthesis protein | SAG1412, SAK1447, GBS1482 |
| 9 | C1161/R | 0.9829 | [*rnz*] ribonuclease Z | SAG1210, SAK1296, GBS1282 |
| 10 | C1074 | 0.9828 | polysaccharide deacetylase family protein | SAG1121, SAK1206, GBS1188 |
| **o) *pbp*1A (*pon*A) gene rank** | | | | |
| 1 | C0156/M | 0.9993 | penicillin-binding protein 1B, putative | SAG0159, SAK0222, GBS0155 |
| 2 | C1930/M | 0.9992 | [*pbp*2A] penicillin-binding protein 2A | SAG2066, SAK2005, GBS2020 |
| 3 | C0030/M | 0.9979 | [*zoo*A] peptidase, M23/M37 family | SAG0031, SAK0064, GBS0030 |
| 4 | C0136 | 0.9959 | [*upp*P] undecaprenyl pyrophosphate phosphatase | SAG0138, SAK0196, GBS0134 |
| 5 | C1519 | 0.9958 | hypothetical protein | SAG1613, SAK1628, GBS1662 |
| 6 | C1586/M | 0.9958 | [*alr*] alanine racemase | SAG1684, SAK1696, GBS1728 |
| 7 | C1823/TK | 0.9930 | [*rel*A] GTP pyrophosphokinase family protein | SAG1940, SAK1900, GBS1928 |
| 8 | C0491 | 0.9911 | [*hup*] HU like DNA-binding protein | SAG0505, SAK0606, GBS0551 |
| 9 | C1506/M | 0.9898 | [*mur*I] glutamate racemase | SAG1600, SAK1615, GBS1649 |
| 10 | C0732/M | 0.9895 | penicillin-binding protein 2b | SAG0765, SAK0890, GBS0785 |

This table shows the final ranks of 15 individual GBS virulence gene categories. The rank positions of individual ranks from four machine learning algorithms were combined by using a multiplicative model as described in the methods section. Notes: *) gene is a known virulence gene (see Table 1). †) these genes appeared more than once within the top-10 of other ranks.
