# Supplementary material for: Computational Bacterial Genome-Wide Analysis of Phylogenetic Profiles Reveals Potential Virulence Genes of Streptococcus agalactiae
Source: PLoS One. 2011 Apr 4;6(4):e17964. doi: 10.1371/journal.pone.0017964 (PMC3070697; doi:10.1371/journal.pone.0017964)
Supplement: Table S2 — List of homolog clusters in the three S. agalactiae genomes defined in this paper. (PDF) [file pone.0017964.s003.pdf]

Supporting Information - Table S2: List of homolog clusters of the three *S. agalactiae* genomes defined in this paper

List of homolog clusters in the 3 GBS reference genomes

ID	Locus tag	Gene	COG	Annotation
C0000	SAG0001	*dna*A	L	chromosomal replication initiation protein
	SAK0001	*dna*A	L	chromosomal replication initiation protein
	GBS0001	*dna*A	L	chromosomal replication initiation protein
C0001	SAG0002	*dna*N	L	DNA polymerase III subunit beta
	SAK0002	*dna*N	L	DNA polymerase III subunit beta
	GBS0002	*dna*N	L	DNA polymerase III subunit beta
C0002	SAG0003	-	IR	diacylglycerol kinase catalytic domain protein, putative
	SAK0003	-	IR	diacylglycerol kinase domain protein
	GBS0003	-	IR	hypothetical protein
C0003	SAG0004	-		hypothetical protein
	SAK0004	-		hypothetical protein
	GBS0004	-		hypothetical protein
C0004	SAG0005	-		hypothetical protein
	SAK0005	-		hypothetical protein
	GBS0005	-		hypothetical protein
C0005	SAG0006	*ych*F	J	GTP-binding protein YchF
	SAK0006	*ych*F	J	GTP-binding protein YchF
	GBS0006	-	J	hypothetical protein
C0006	SAG0007	*pth*		peptidyl-tRNA hydrolase
	SAK0007	*pth*		peptidyl-tRNA hydrolase
	GBS0007	-		peptidyl-tRNA hydrolase
C0007	SAG0008	*mfd*	LK	transcription-repair coupling factor
	SAK0008	*mfd*	LK	transcription-repair coupling factor
	GBS0008	-	LK	hypothetical protein
C0008	SAG0009	-		hypothetical protein
C0009	SAG0010	-	J	S4 domain protein
	SAK0009	-	J	S4 domain protein
	GBS0009	-	J	hypothetical protein
C0010	SAG0011	-	D	cell division protein DivIC, putative
	SAK0010	-	D	hypothetical protein
	GBS0010	-	D	hypothetical protein
C0011	SAG0012	-		hypothetical protein
	SAK0011	-		hypothetical protein
	GBS0011	-		hypothetical protein
C0012	SAG0013	-	V	hypothetical protein
	SAK0012	-	V	hypothetical protein
	GBS0012	-	V	hypothetical protein
C0013	SAG0014	-	D	MesJ/Ycf62 family protein
	SAK0013	*til*S	D	tRNA(Ile)-lysidine synthetase
	GBS0013	-	D	hypothetical protein
C0014	SAG0015	*hpt*	F	hypoxanthine-guanine phosphoribosyltransferase
	SAK0014	*hpt*	F	hypoxanthine-guanine phosphoribosyltransferase
	GBS0014	-	F	hypoxanthine-guanine phosphoribosyltransferase
C0015	SAG0016	*fts*H	O	cell division protein FtsH
	SAK0015	*fts*H	O	cell division protein FtsH
	GBS0015	*fts*H	O	cell division protein FtsH
C0016	SAG0017	*psc*B	S	pcsB protein
	SAK0050	*pcs*B	R	PcsB protein
	GBS0016	*pcs*B	R	PcsB protein
C0017	SAG0018	*prs*A-1	FE	ribose-phosphate pyrophosphokinase
	SAK0051	*prs*	FE	ribose-phosphate pyrophosphokinase
	GBS0017	-	FE	ribose-phosphate pyrophosphokinase
C0018	SAG0019	-	E	hypothetical protein
	SAK0052	-	E	hypothetical protein
	GBS0018	-	E	hypothetical protein
C0019	SAG0020	*rec*O	L	DNA repair protein RecO
	SAK0053	*rec*O	L	DNA repair protein RecO
	GBS0019	*rec*O	L	DNA repair protein RecO
C0020	SAG0021	-		protease, putative
	SAK0054	-		CAAX amino terminal protease family protein
	GBS0020	-		hypothetical protein
C0021	SAG0022	*pls*X	I	fatty acid/phospholipid synthesis protein
	SAK0055	*pls*X	I	fatty acid/phospholipid synthesis protein
	GBS0021	-	I	fatty acid/phospholipid synthesis protein
C0022	SAG0023	*acp*P-1	IQ	acyl carrier protein
	SAK0056	-	IQ	acyl carrier protein
	GBS0022	-	IQ	acyl carrier protein

(Continue on next page)

List of homolog clusters in the 3 GBS reference genomes (Cont'd)

ID	Locus tag	Gene	COG	Annotation
C0023	SAG0024	*pur*C		phosphoribosylaminoimidazole-succinocarboxamide synthase
	SAK0057	*pur*C		phosphoribosylaminoimidazole-succinocarboxamide synthase
	GBS0023	-		phosphoribosylaminoimidazole-succinocarboxamide synthase
C0024	SAG0025	-	F	phosphoribosylformylglycinamidine synthase, putative
	SAK0058	-	F	phosphoribosylformylglycinamidine synthase
	GBS0024	-	F	hypothetical protein
C0025	SAG0026	*pur*F	F	amidophosphoribosyltransferase
	SAK0059	*pur*F	F	amidophosphoribosyltransferase
	GBS0025	-	F	amidophosphoribosyltransferase
C0026	SAG0027	*pur*M	F	phosphoribosylaminoimidazole synthetase
	SAK0060	*pur*M	F	phosphoribosylaminoimidazole synthetase
	GBS0026	-	F	phosphoribosylaminoimidazole synthetase
C0027	SAG0028	*pur*N		phosphoribosylglycinamide formyltransferase
	SAK0061	*pur*N		phosphoribosylglycinamide formyltransferase
	GBS0027	-		phosphoribosylglycinamide formyltransferase
C0028	SAG0029	-		acetyltransferase, GNAT family
	SAK0062	-		acetyltransferase, GNAT family
	GBS0028	-		hypothetical protein
C0029	SAG0030	*pur*H		bifunctional phosphoribosylaminoimidazolecarboxamide formyltransferase/IMP cyclohydrolase
	SAK0063	*pur*H		bifunctional phosphoribosylaminoimidazolecarboxamide formyltransferase/IMP cyclohydrolase
	GBS0029	*pur*H		bifunctional phosphoribosylaminoimidazolecarboxamide formyltransferase/IMP cyclohydrolase
C0030	SAG0031	-	M	peptidase, M23/M37 family
	SAK0064	*zoo*A	M	zoocin A
	GBS0030	-	M	hypothetical protein
C0031	SAG0032	-		group B streptococcal surface immunogenic protein
	SAK0065	*sip*		group B streptococcal surface immunogenic protein
	GBS0031	-		hypothetical protein
C0032	SAG0033	-	G	N-acetylmannosamine-6-phosphate 2-epimerase
	SAK0066	-	G	N-acetylmannosamine-6-phosphate 2-epimerase
	GBS0032	-	G	N-acetylmannosamine-6-phosphate 2-epimerase
C0033	SAG0034	-	G	sugar ABC transporter, sugar-binding protein
	SAK0067	-	G	carbohydrate uptake 1 (CUT1) family ABC transporter, carbohydrate-binding protein
	SAK0532	-	G	sugar ABC transporter, sugar-binding protein, putative
	GBS0033	-	G	hypothetical protein
C0034	SAG0035	-	G	sugar ABC transporter, permease protein
	SAK0068	-	G	carbohydrate uptake 1 (CUT1) family ABC transporter, permease protein
	SAK0533	-	G	sugar ABC transporter, permease protein, putative
	GBS0034	-	G	hypothetical protein
C0035	SAG0036	-	G	sugar ABC transporter, permease protein
	SAK0069	-	G	carbohydrate uptake 1 (CUT1) family ABC transporter, permease protein
	SAK0534	-	G	ABC transporter, permease protein
	GBS0035	-	G	hypothetical protein
C0036	SAG0037	-		hypothetical protein
	SAK0070	-		hypothetical protein
	GBS0036	-		hypothetical protein
C0037	SAG0038	-		hypothetical protein
	SAK0071	-		hypothetical protein
	GBS0037	-		hypothetical protein
C0038	SAG0039	-	EM	N-acetylneuraminate lyase, putative
	SAK0072	-	EM	N-acetylneuraminate lyase, putative
	GBS0038	-	EM	hypothetical protein
C0039	SAG0040	-	KG	ROK family protein
	SAK0073	-	KG	ROK family protein
	GBS0039	-	KG	hypothetical protein
C0040	SAG0041	-	Q	acetyl xylan esterase, putative
	SAK0074	-	Q	acetyl xylan esterase, putative
	GBS0040	-	Q	hypothetical protein
C0041	SAG0042	-	K	phosphosugar-binding transcriptional regulator, RpiR family
	SAK0075	-	K	phosphosugar-binding transcriptional regulator, RpiR family
	GBS0041	-	K	hypothetical protein
C0042	SAG0043	*pur*D	F	phosphoribosylamine–glycine ligase
	SAK0076	*pur*D	F	phosphoribosylamine–glycine ligase
	GBS0042	-	F	phosphoribosylamine–glycine ligase
C0043	SAG0044	*pur*E	F	phosphoribosylaminoimidazole carboxylase catalytic subunit
	SAK0077	*pur*E	F	phosphoribosylaminoimidazole carboxylase catalytic subunit
	GBS0043	-	F	phosphoribosylaminoimidazole carboxylase catalytic subunit

(Continue on next page)

List of homolog clusters in the 3 GBS reference genomes (Cont'd)

ID	Locus tag	Gene	COG	Annotation
C0044	SAG0045	*pur*K	F	phosphoribosylaminoimidazole carboxylase
	SAK0078	*pur*K	F	phosphoribosylaminoimidazole carboxylase
	GBS0044	-	F	phosphoribosylaminoimidazole carboxylase
C0045	SAG0046	-		hypothetical protein
	SAK0079	-		hypothetical protein
C0046	SAG0047	*pur*B	F	adenylosuccinate lyase
	SAK0080	*pur*B	F	adenylosuccinate lyase
	GBS0047	-	F	adenylosuccinate lyase
C0047	SAG0048	-	K	transcriptional regulator, Cro/CI family
	SAK0081	-		DNA-binding protein
	GBS0048	-		hypothetical protein
C0048	SAG0049	*ruv*B	L	Holliday junction DNA helicase RuvB
	SAK0082	*ruv*B	L	Holliday junction DNA helicase RuvB
	GBS0049	*ruv*B	L	Holliday junction DNA helicase RuvB
C0049	SAG0050	-	T	phosphotyrosine protein phosphatase, low molecular weight
	SAK0083	-	T	low molecular weight phosphotyrosine protein phosphatase
	GBS0050	-	T	hypothetical protein
C0050	SAG0051	-	S	MORN motif family protein
	SAK0084	-	S	MORN repeat family protein
	GBS0051	-	S	hypothetical protein
C0051	SAG0052	-	I	hypothetical protein
	SAK0085	-	I	acyltransferase, putative
	GBS0052	-	I	hypothetical protein
C0052	SAG0053	*adh*E	C	aldehyde-alcohol dehydrogenase
	SAK0086	-	C	aldehyde-alcohol dehydrogenase
	GBS0053	-	C	hypothetical protein
C0053	SAG0054	*adh*P	R	alcohol dehydrogenase
	SAK0087	-	R	alcohol dehydrogenase
	GBS0054	-	R	alcohol dehydrogenase
C0054	SAG0055	*thr*C	E	threonine synthase
	SAK0088	*thr*C	E	threonine synthase
	GBS0055	*thr*C	E	threonine synthase
C0055	SAG0056	-	V	MATE efflux family protein
	SAK0089	-	V	MATE efflux family protein
	GBS0056	-	V	hypothetical protein
C0056	SAG0057	*rps*J	J	30S ribosomal protein S10
	SAK0090	*rps*J	J	30S ribosomal protein S10
	GBS0057	*rps*J	J	30S ribosomal protein S10
C0057	SAG0058	*rpl*C		50S ribosomal protein L3
	SAK0091	*rpl*C		50S ribosomal protein L3
	GBS0058	*rpl*C		50S ribosomal protein L3
C0058	SAG0059	*rpl*D		50S ribosomal protein L4
	SAK0092	*rpl*D		50S ribosomal protein L4
	GBS0059	*rpl*D		50S ribosomal protein L4
C0059	SAG0060	*rpl*W	J	50S ribosomal protein L23
	SAK0093	*rpl*W	J	50S ribosomal protein L23
	GBS0060	*rpl*W	J	50S ribosomal protein L23
C0060	SAG0061	*rpl*B	J	50S ribosomal protein L2
	SAK0094	*rpl*B	J	50S ribosomal protein L2
	GBS0061	*rpl*B	J	50S ribosomal protein L2
C0061	SAG0062	*rps*S	J	30S ribosomal protein S19
	SAK0095	*rps*S	J	30S ribosomal protein S19
	GBS0062	*rps*S	J	30S ribosomal protein S19
C0062	SAG0063	*rpl*V		50S ribosomal protein L22
	SAK0096	*rpl*V		50S ribosomal protein L22
	GBS0063	*rpl*V		50S ribosomal protein L22
C0063	SAG0064	*rps*C	J	30S ribosomal protein S3
	SAK0097	*rps*C	J	30S ribosomal protein S3
	GBS0064	*rps*C	J	30S ribosomal protein S3
C0064	SAG0065	*rpl*P	J	50S ribosomal protein L16
	SAK0098	*rpl*P	J	50S ribosomal protein L16
	GBS0065	*rpl*P	J	50S ribosomal protein L16
C0065	SAG0066	*rpm*C		50S ribosomal protein L29
	SAK0099	*rpm*C		50S ribosomal protein L29
	GBS0066	*rpm*C		50S ribosomal protein L29
C0066	SAG0067	*rps*Q	J	30S ribosomal protein S17
	SAK0100	*rps*Q	J	30S ribosomal protein S17
	GBS0067	*rps*Q	J	30S ribosomal protein S17
C0067	SAG0068	*rpl*N		50S ribosomal protein L14
	SAK0101	*rpl*N		50S ribosomal protein L14
	GBS0068	*rpl*N		50S ribosomal protein L14

(Continue on next page)

List of homolog clusters in the 3 GBS reference genomes (Cont'd)

ID	Locus tag	Gene	COG	Annotation
C0068	SAG0069	*rpl*X	J	50S ribosomal protein L24
	SAK0102	*rpl*X	J	50S ribosomal protein L24
	GBS0069	*rpl*X	J	50S ribosomal protein L24
C0069	SAG0070	*rpl*E	J	50S ribosomal protein L5
	SAK0103	*rpl*E	J	50S ribosomal protein L5
	GBS0070	*rpl*E	J	50S ribosomal protein L5
C0070	SAG0071	-	J	30S ribosomal protein S14
	SAK0104	*rps*NA	J	30S ribosomal protein S14
	GBS0071	-	J	30S ribosomal protein S14
C0071	SAG0072	*rps*H		30S ribosomal protein S8
	SAK0105	*rps*H		30S ribosomal protein S8
	GBS0072	*rps*H		30S ribosomal protein S8
C0072	SAG0073	*rpl*F	J	50S ribosomal protein L6
	SAK0106	*rpl*F	J	50S ribosomal protein L6
	GBS0073	*rpl*F	J	50S ribosomal protein L6
C0073	SAG0074	*rpl*R	J	50S ribosomal protein L18
	SAK0107	*rpl*R	J	50S ribosomal protein L18
	GBS0074	*rpl*R	J	50S ribosomal protein L18
C0074	SAG0075	*rps*E	J	30S ribosomal protein S5
	SAK0108	*rps*E	J	30S ribosomal protein S5
	GBS0075	*rps*E	J	30S ribosomal protein S5
C0075	SAG0076	*rpm*D	J	50S ribosomal protein L30
	SAK0109	*rpm*D	J	50S ribosomal protein L30
	GBS0076	*rpm*D	J	50S ribosomal protein L30
C0076	SAG0077	*rpl*O	J	50S ribosomal protein L15
	SAK0110	*rpl*O	J	50S ribosomal protein L15
	GBS0077	*rpl*O	J	50S ribosomal protein L15
C0077	SAG0078	-	U	preprotein translocase SecY
	SAK0111	*sec*Y	U	preprotein translocase SecY
	GBS0078	*sec*Y	U	preprotein translocase SecY
C0078	SAG0079	*adk*	F	adenylate kinase
	SAK0112	*adk*	F	adenylate kinase
	GBS0079	*adk*	F	adenylate kinase
C0079	SAG0080	*inf*A	J	translation initiation factor IF-1
	SAK0113	*inf*A	J	translation initiation factor IF-1
	GBS0080	*inf*A	J	translation initiation factor IF-1
C0080	SAG0081	*rpm*J	J	50S ribosomal protein L36
	GBS0081	-	J	50S ribosomal protein L36
C0081	SAG0082	*rps*M	J	30S ribosomal protein S13
	SAK0114	*rps*M	J	30S ribosomal protein S13
	GBS0082	*rps*M	J	30S ribosomal protein S13
C0082	SAG0083	*rps*K	J	30S ribosomal protein S11
	SAK0115	*rps*K	J	30S ribosomal protein S11
	GBS0083	*rps*K	J	30S ribosomal protein S11
C0083	SAG0084	*rpo*A	K	DNA-directed RNA polymerase alpha subunit
	SAK0116	*rpo*A	K	DNA-directed RNA polymerase alpha subunit
	GBS0084	*rpo*A	K	DNA-directed RNA polymerase alpha subunit
C0084	SAG0085	*rpl*Q	J	50S ribosomal protein L17
	SAK0117	*rpl*Q	J	50S ribosomal protein L17
	GBS0085	*rpl*Q	J	50S ribosomal protein L17
C0085	SAG0086	-		lipoprotein, putative
	SAG0087	-		hypothetical protein
	SAG0245	-		protein of unknown function/lipoprotein, putative
	SAG0257	-		lipoprotein, putative
	SAK0137	-		lipoprotein, putative
	SAK0321	-		lipoprotein, putative
	GBS0086	-		hypothetical protein
C0086	SAG0088	-		hypothetical protein
	SAK0138	-		hypothetical protein
C0087	SAG0089	-		hypothetical protein
	SAK0139	-		hypothetical protein
	GBS0088	-		hypothetical protein
C0088	SAG0090	-		hypothetical protein
	SAK0140	-		hypothetical protein
	GBS0089	-		hypothetical protein
C0089	SAG0091	-		transcriptional regulator ComX1, putative
	SAK0141	*com*X		competence-specific sigma factor ComX
	GBS0090	-		hypothetical protein
C0090	SAG0092	-	G	phosphoglycerate mutase family protein
	SAK0142	-	G	phosphoglycerate mutase family protein
	GBS0091	-	G	hypothetical protein

(Continue on next page)

List of homolog clusters in the 3 GBS reference genomes (Cont'd)

ID	Locus tag	Gene	COG	Annotation
C0091	SAG0093	-		D-alanyl-D-alanine carboxypeptidase family protein
	SAK0143	-		D-alanyl-D-alanine carboxypeptidase
	GBS0092	-		hypothetical protein
C0092	SAG0094	-	NU	N-acetylmuramoyl-L-alanine amidase, family 4 protein
	SAK0144	-	NU	mannosyl-glycoprotein endo-beta-N-acetylglucosamidase family protein
	GBS0093	-	NU	hypothetical protein
C0093	SAG0095	*hrc*A	K	heat-inducible transcription repressor
	SAK0145	*hrc*A	K	heat-inducible transcription repressor
	GBS0094	-	K	heat-inducible transcription repressor
C0094	SAG0096	*grp*E	O	heat shock protein GrpE
	SAK0146	*grp*E	O	co-chaperone protein GrpE
	GBS0095	-	O	hypothetical protein
C0095	SAG0097	*dna*K	O	molecular chaperone DnaK
	SAK0147	*dna*K	O	molecular chaperone DnaK
	GBS0096	*dna*K	O	molecular chaperone DnaK
C0096	SAG0098	*dna*J	O	dnaJ protein
	SAK0148	*dna*J	O	co-chaperone protein DnaJ
	GBS0097	*dna*J	O	Chaperone protein DnaJ
C0097	SAG0099	-	KE	transcriptional regulator, GntR family
	SAK0149	-	KE	transcriptional regulator, GntR family
	GBS0098	-	KE	hypothetical protein
C0098	SAG0100	*tru*A	J	tRNA pseudouridine synthase A
	SAK0150	*tru*A	J	tRNA pseudouridine synthase A
	GBS0099	*tru*A	J	tRNA pseudouridine synthase A
C0099	SAG0101	-	H	phosphomethylpyrimidine kinase
	SAK0151	-	H	phosphomethylpyrimidine kinase
	GBS0100	-	H	phosphomethylpyrimidine kinase
C0100	SAG0102	-	S	hypothetical protein
	SAK0152	-	S	hypothetical protein
	GBS0101	-	S	hypothetical protein
C0101	SAG0103	-		conserved hypothetical protein TIGR01440
	SAK0153	-		conserved hypothetical protein TIGR01440
	GBS0102	-		hypothetical protein
C0102	SAG0104	-	M	hypothetical protein
	SAK0154	-	M	mechanosensitive ion channel family protein
	GBS0103	-	M	hypothetical protein
C0103	SAG0105	*tig*	O	trigger factor
	SAK0155	*tig*	O	trigger factor
	GBS0104	*tig*	O	trigger factor
C0104	SAG0106	-	K	DNA-directed RNA polymerase subunit delta
	SAK0156	*rpo*E	K	DNA-directed RNA polymerase subunit delta
	GBS0105	-	K	DNA-directed RNA polymerase subunit delta
C0105	SAG0107	*pyr*G	F	CTP synthetase
	SAK0157	*pyr*G	F	CTP synthetase
	GBS0106	-	F	CTP synthetase
C0106	SAG0108	-	R	hypothetical protein
	SAK0158	-	R	hypothetical protein
	GBS0107	-	R	hypothetical protein
C0107	SAG0109	*dut*		deoxyuridine 5'-triphosphate nucleotidohydrolase
	SAK0160	*dut*		deoxyuridine 5'-triphosphate nucleotidohydrolase
	GBS0108	-		deoxyuridine 5'-triphosphate nucleotidohydrolase
C0108	SAG0110	*rad*A	O	DNA repair protein RadA
	SAK0161	*rad*A	O	DNA repair protein RadA
	GBS0109	-	O	hypothetical protein
C0109	SAG0111	-		carbonic anhydrase-related protein
	SAK0162	-		carbonic anhydrase, putative
	GBS0110	-		hypothetical protein
C0110	SAG0112	-	C	pyridine nucleotide-disulfide oxidoreductase
	SAG1254	*mer*A-1	C	mercuric reductase
	SAG2023	*mer*A-2	C	mercuric reductase
	SAK0163	-	C	pyridine nucleotide-disulfide oxidoreductase
	GBS0111	-	C	pyridine nucleotide-disulfide oxidoreductase
C0111	SAG0113	*glt*X	J	glutamyl-tRNA synthetase
	SAK0165	*glt*X	J	glutamyl-tRNA synthetase
	GBS0112	*glt*X	J	glutamyl-tRNA synthetase
C0112	SAG0114	*rbs*B	G	ribose ABC transporter, periplasmic D-ribose-binding protein
	SAK0166	*rbs*B	G	ribose ABC transporter, ribose-binding protein
	GBS0113	-	G	hypothetical protein
C0113	SAG0115	*rbs*C	G	ribose ABC transporter, permease protein
	SAK0167	*rbs*C	G	ribose ABC transporter, permease protein
	GBS0114	-	G	hypothetical protein

(Continue on next page)

List of homolog clusters in the 3 GBS reference genomes (Cont'd)

ID	Locus tag	Gene	COG	Annotation
C0114	SAG0116	*rbs*A	G	ribose ABC transporter, ATP-binding protein
	SAK0168	*rbs*A	G	ribose ABC transporter, ATP-binding protein
	GBS0115	-	G	hypothetical protein
C0115	SAG0117	*rbs*D	G	ribose ABC transporter protein RbsD
	SAK0169	*rbs*D	G	ribose ABC transporter protein RbsD
	GBS0116	-	G	hypothetical protein
C0116	SAG0118	*rbs*K	G	ribokinase
	SAK0170	*rbs*K	G	ribokinase
	GBS0117	-	G	hypothetical protein
C0117	SAG0119	*rbs*R	K	ribose operon repressor RbsR
	SAK0171	*rbs*R	K	ribose operon repressor
	GBS0118	-	K	hypothetical protein
C0118	SAG0120	-		hypothetical protein
C0119	SAG0121	-	V	permease, putative
	SAK0172	-	V	permease, putative
	GBS0119	-	V	hypothetical protein
C0120	SAG0122	-	V	ABC transporter, ATP-binding protein
	SAK0173	-	V	ABC transporter, ATP-binding protein
	GBS0120	-	V	hypothetical protein
C0121	SAG0123	-	TK	DNA-binding response regulator
	SAK0174	-	TK	DNA-binding response regulator
	GBS0121	-	TK	hypothetical protein
C0122	SAG0124	-	T	sensor histidine kinase
	SAK0175	-	T	sensor histidine kinase
	GBS0122	-	T	hypothetical protein
C0123	SAG0125	*arg*G	E	argininosuccinate synthase
	SAK0176	*arg*G	E	argininosuccinate synthase
	GBS0123	-	E	argininosuccinate synthase
C0124	SAG0126	*arg*H	E	argininosuccinate lyase
	SAK0177	*arg*H	E	argininosuccinate lyase
	GBS0124	-	E	argininosuccinate lyase
C0125	SAG0127	*fba*	G	fructose-bisphosphate aldolase
	SAK0178	*fba*	G	fructose-bisphosphate aldolase
	GBS0125	-	G	fructose-bisphosphate aldolase
C0126	SAG0128	-	C	L-2-hydroxyisocaproate dehydrogenase
	SAK0179	-	C	L-2-hydroxyisocaproate dehydrogenase
	GBS0126	-	C	hypothetical protein
C0127	SAG0129	*rpm*B	J	50S ribosomal protein L28
	SAK0180	*rpm*B	J	50S ribosomal protein L28
	GBS0127	*rpm*V	J	50S ribosomal protein L28
C0128	SAG0130	-		hypothetical protein
	SAK0181	-		hypothetical protein
	GBS0128	-		hypothetical protein
C0129	SAG0131	-	R	DAK2 domain protein
	SAK0182	-	R	DAK2 domain protein
	GBS0129	-	R	hypothetical protein
C0130	SAG0132	-	O	SPFH domain/Band 7 family protein
	SAK0183	-	O	SPFH domain/band 7 family protein
	GBS0130	-	O	hypothetical protein
C0131	SAG0133	-		hypothetical protein
C0132	SAG0134	-		hypothetical protein
	SAK0192	-		hypothetical protein
C0133	SAG0135	-	E	amino acid ABC transporter, ATP-binding protein
	SAK0193	-	E	amino acid ABC transporter, ATP-binding protein, putative
	GBS0131	-	E	hypothetical protein
C0134	SAG0136	-	E	amino acid ABC transporter, amino acid-binding protein/permease protein
	SAK0194	-	E	amino acid ABC transporter, amino acid-binding/permease protein, His/Glu/Gln/Arg/opine family
	GBS0132	-	ET	hypothetical protein
C0135	SAG0137	-	S	hypothetical protein
	SAK0195	-	S	hypothetical protein
	GBS0133	-	S	hypothetical protein
C0136	SAG0138	*upp*P		undecaprenyl pyrophosphate phosphatase
	SAK0196	*upp*P		undecaprenyl pyrophosphate phosphatase
	GBS0134	*upp*P		undecaprenyl pyrophosphate phosphatase
C0137	SAG0139	-		adaptor protein
	SAK0197	-		adaptor protein
	GBS0135	-		adaptor protein
C0138	SAG0140	-	M	glycosyl transferase, group 4 family protein
	SAK0198	-	M	glycosyl transferase family protein
	GBS0136	-	M	hypothetical protein

(Continue on next page)

List of homolog clusters in the 3 GBS reference genomes (Cont'd)

ID	Locus tag	Gene	COG	Annotation
C0139	SAG0141	-	O	ABC transporter, ATP-binding protein
	SAK0199	*suf*C	O	FeS assembly ATPase SufC
	GBS0137	-	O	hypothetical protein
C0140	SAG0142	-		hypothetical protein
	SAK0200	*suf*D		FeS assembly protein SufD
	GBS0138	-		hypothetical protein
C0141	SAG0143	*csd*B	E	selenocysteine lyase
	SAK0201	*suf*S	E	cysteine desulfurase SufS
	GBS0139	-	E	hypothetical protein
C0142	SAG0144	-	C	NifU family protein
	SAK0202	-	C	SUF system FeS assembly protein, NifU family
	GBS0140	-	C	hypothetical protein
C0143	SAG0145	-		hypothetical protein
	SAK0203	*suf*B		FeS assembly protein SufB
	GBS0141	-		hypothetical protein
C0144	SAG0146	-	M	penicillin-binding protein 4, putative
	SAK0204	-	M	D-alanyl-D-alanine carboxypeptidase
	GBS0142	-	M	hypothetical protein
C0145	SAG0147	-	M	D-alanyl-D-alanine carboxypeptidase family protein
	SAK0205	-	M	D-alanyl-D-alanine carboxypeptidase
	GBS0143	-	M	hypothetical protein
C0146	SAG0148	-	E	oligopeptide ABC transporter, substrate-binding protein, putative
	SAG0979	-	E	ABC transporter, substrate-binding protein
	SAK0206	-	E	oligopeptide ABC transporter, oligopeptide-binding protein, putative
	SAK1074	-	E	ABC transporter, substrate-binding protein
	GBS0144	-	E	hypothetical protein
	GBS0966	-	E	hypothetical protein
C0147	SAG0149	-	EP	oligopeptide ABC transporter, permease protein
	SAK0207	*opp*D	EP	oligopeptide ABC transporter, permease protein
	GBS0145	-	EP	hypothetical protein
C0148	SAG0150	-	EP	oligopeptide ABC transporter, permease protein
	SAK0208	-	EP	oligopeptide ABC transporter, permease protein OppC, putative
	GBS0146	-	EP	hypothetical protein
C0149	SAG0151	-	EP	oligopeptide ABC transporter, ATP-binding protein
	SAK0209	*opp*D	EP	oligopeptide ABC transporter, ATP-binding protein
	GBS0147	-	EP	hypothetical protein
C0150	SAG0152	-	E	oligopeptide ABC transporter, ATP-binding protein
	SAK0210	*opp*F	E	oligopeptide ABC transporter, ATP-binding protein
	GBS0148	-	E	hypothetical protein
C0151	SAG0153	*isp*E	I	4-diphosphocytidyl-2-C-methyl-D-erythritol kinase
	SAK0216	*isp*E	I	4-diphosphocytidyl-2-C-methyl-D-erythritol kinase
	GBS0149	-	I	4-diphosphocytidyl-2-C-methyl-D-erythritol kinase
C0152	SAG0154	*adc*R	K	adc operon repressor AdcR
	SAK0217	*adc*R	K	adc operon repressor AdcR
	GBS0150	-	K	hypothetical protein
C0153	SAG0155	-	P	zinc ABC transporter, ATP-binding protein
	SAK0218	*adc*C	P	zinc ABC transporter, ATP-binding protein
	GBS0151	-	P	hypothetical protein
C0154	SAG0156	-	P	zinc ABC transporter, permease protein
	SAK0219	*adc*B	P	zinc ABC transporter, permease protein
	GBS0152	-	P	hypothetical protein
C0155	SAG0158	*tyr*S	J	tyrosyl-tRNA synthetase
	SAK0221	*tyr*S	J	tyrosyl-tRNA synthetase
	GBS0154	*tyr*S	J	tyrosyl-tRNA synthetase
C0156	SAG0159	-	M	penicillin-binding protein 1B, putative
	SAK0222	-	M	penicillin-binding protein 1B
	GBS0155	-	M	hypothetical protein
C0157	SAG0160	*rpo*B	K	DNA-directed RNA polymerase beta subunit
	SAK0223	*rpo*B	K	DNA-directed RNA polymerase beta subunit
	GBS0156	*rpo*B	K	DNA-directed RNA polymerase beta subunit
C0158	SAG0161	*rpo*C	K	DNA-directed RNA polymerase beta' subunit
	SAK0224	*rpo*C	K	DNA-directed RNA polymerase beta' subunit
	GBS0157	*rpo*C	K	DNA-directed RNA polymerase beta' subunit
C0159	SAG0162	-		hypothetical protein
	SAK0225	-		hypothetical protein
	GBS0158	-		hypothetical protein
C0160	SAG0163	*cgl*A	NU	competence protein CglA
	SAK0226	*com*GA	NU	competence protein ComGA
	GBS0159	-	NU	hypothetical protein

(Continue on next page)

List of homolog clusters in the 3 GBS reference genomes (Cont'd)

ID	Locus tag	Gene	COG	Annotation
C0161	SAG0164	*cgl*B	NU	competence protein CglB
	SAK0227	-	NU	ComG operon protein 2, putative
	GBS0160	-	NU	hypothetical protein
C0162	SAG0165	-	U	hypothetical protein
	SAK0231	-	U	hypothetical protein
	GBS0164	-	U	hypothetical protein
C0163	SAG0166	-		hypothetical protein
	SAK0232	-		hypothetical protein
	GBS0165	-		hypothetical protein
C0164	SAG0167	-	L	hypothetical protein
	SAK0233	-	L	hypothetical protein
	GBS0166	-	L	hypothetical protein
C0165	SAG0168	*ack*A	C	acetate kinase
	SAK0234	*ack*A	C	acetate kinase
	GBS0167	*ack*A	C	acetate kinase
C0166	SAG0169	-	K	transcriptional regulator, Cro/CI family
	SAG0251	-	K	transcriptional regulator, Cro/CI family
	SAK0235	-	K	DNA-binding protein
	SAK0326	-	K	DNA-binding protein
	GBS0168	-	K	hypothetical protein
C0167	SAG0170	-		hypothetical protein
	SAK0236	-		hypothetical protein
C0168	SAG0171	-		hypothetical protein
	SAK0237	-		hypothetical protein
	GBS0169	-		hypothetical protein
C0169	SAG0172	-		protease, putative
	SAG1293	-		protease, putative
	SAK0238	-		CAAX amino terminal protease family protein
	GBS0170	-		hypothetical protein
	GBS1366	-		hypothetical protein
C0170	SAG0173	*pro*C	E	pyrroline-5-carboxylate reductase
	SAK0239	*pro*C	E	pyrroline-5-carboxylate reductase
	GBS0171	-	E	pyrroline-5-carboxylate reductase
C0171	SAG0174	*pep*A	G	glutamyl-aminopeptidase
	SAK0240	*pep*A	G	glutamyl aminopeptidase
	GBS0172	-	G	hypothetical protein
C0172	SAG0175	-		hypothetical protein
	SAK0241	-		hypothetical protein
	GBS0173	-		hypothetical protein
C0173	SAG0176	-		hypothetical protein
	SAK0242	-		hypothetical protein
	GBS0174	-		hypothetical protein
C0174	SAG0177	-	OC	thioredoxin family protein
	SAK0243	-	OC	thioredoxin family protein
	GBS0175	-	OC	hypothetical protein
C0175	SAG0178	-	R	tRNA binding domain protein
	SAK0244	-	R	tRNA binding domain protein
	GBS0176	-	R	hypothetical protein
C0176	SAG0179	-	QR	hypothetical protein
	SAK0245	-	QR	hypothetical protein
	GBS0177	-	QR	hypothetical protein
C0177	SAG0180	*ssb*	L	single-strand DNA-binding protein
	SAK0246	*ssb*2	L	single-strand DNA-binding protein
	GBS0178	-	L	single-strand DNA-binding protein
C0178	SAG0181	-	R	hydrolase, haloacid dehalogenase-like family
	SAK0247	-	R	HAD-superfamily hydrolase, subfamily IA, variant 3 family protein
	GBS0179	-	R	hypothetical protein
C0179	SAG0182	-	T	sensor histidine kinase, putative
	SAK0248	-	T	sensor histidine kinase
	GBS0180	-	T	hypothetical protein
C0180	SAG0183	-	KT	response regulator
	SAK0249	-	KT	DNA-binding response regulator
	GBS0181	-	KT	hypothetical protein
C0181	SAG0184	-	R	hypothetical protein
	SAK0250	-	R	LrgA family protein
	GBS0182	-	R	hypothetical protein
C0182	SAG0185	-		hypothetical protein
	SAK0251	-		lrgB-like family protien
	GBS0183	-		hypothetical protein
C0183	SAG0186	-		hypothetical protein

(Continue on next page)

List of homolog clusters in the 3 GBS reference genomes (Cont'd)

ID	Locus tag	Gene	COG	Annotation
	SAG0187	-	E	oligopeptide ABC transporter, oligopeptide-binding protein
C0184	SAK0252	-	E	peptide/opine/nickel uptake (PepT) family ABC transporter, substrate-binding protein
	GBS0184	-	E	hypothetical protein
	SAG0188	-	EP	oligopeptide ABC transporter, permease protein
C0185	SAK0253	-	EP	peptide/opine/nickel uptake (PepT) family ABC transporter, permease protein
	GBS0185	-	EP	hypothetical protein
	SAG0189	-	EP	oligopeptide ABC transporter, permease protein
C0186	SAK0254	-	EP	peptide/opine/nickel uptake (PepT) family ABC transporter, permease protein
	GBS0186	-	EP	hypothetical protein
	SAG0190	-	EP	peptide ABC transporter, ATP-binding protein
C0187	SAK0255	-	EP	peptide/opine/nickel uptake (PepT) family ABC transporter, ATP-binding protein
	GBS0187	-	EP	hypothetical protein
	SAG0191	-	EP	peptide ABC transporter, ATP-binding protein
C0188	SAK0256	-	EP	peptide/opine/nickel uptake (PepT) family ABC transporter, ATP-binding protein
	GBS0188	-	EP	hypothetical protein
	SAG0192	-	G	PTS system, IIABC components
C0189	SAK0257	-	G	PTS system, trehalose-specific IIBCA component
	GBS0189	-	G	hypothetical protein
	SAG0193	-		alpha amylase family protein
C0190	SAK0258	-		alpha amylase family protein
	GBS0190	-		hypothetical protein
	SAG0194	-	K	transcriptional antiterminator, BglG family
	SAG0196	-	GT	hypothetical protein
C0191	SAK0259	-	GT	PRD domain/PTS system IIA domain protein
	SAK0523	-	K	PTS system IIA domain protein
	GBS0191	-	K	hypothetical protein
C0192	SAG0195	-	L	IS1548 transposase
	SAG0197	-	G	PTS system, IIB component, putative
C0193	SAK0260	-	G	PTS system, IIB component, lactose/cellobiose family
	GBS0192	-	G	hypothetical protein
	SAG0198	*ula*A		ascorbate-specific PTS system enzyme IIC
C0194	SAK0261	*ula*A		ascorbate-specific PTS system enzyme IIC
	GBS0193	*ula*A		ascorbate-specific PTS system enzyme IIC
	SAG0199	-	G	transketolase, N-terminal subunit
C0195	SAK0262	-	G	transketolase, N-terminal subunit, putative
	GBS0194	-	G	hypothetical protein
	SAG0200	-	G	transketolase, C-terminal subunit
C0196	SAK0263	-	G	transketolase, C-terminal subunit, putative
	GBS0195	-	G	hypothetical protein
	SAG0201	-	P	oxidoreductase, putative
C0197	SAK0264	-	P	oxidoreductase, NAD-binding
	GBS0196	-	P	hypothetical protein
	SAG0202	*rps*O		30S ribosomal protein S15
C0198	SAK0265	*rps*O		30S ribosomal protein S15
	GBS0197	*rps*O		30S ribosomal protein S15
	SAG0203	*pnp*	J	polyribonucleotide nucleotidyltransferase
C0199	SAK0266	*pnp*	J	polyribonucleotide nucleotidyltransferase
	GBS0198	*pnp*A	J	polynucleotide phosphorylase, alpha chain
	SAG0204	-		hypothetical protein
C0200	SAK0267	-		hypothetical protein
	GBS0199	-		hypothetical protein
	SAG0205	*cys*E	E	serine O-acetyltransferase
C0201	SAK0268	*cys*E	E	serine O-acetyltransferase
	GBS0200	*cys*E	E	hypothetical protein
	SAG0206	-		lipoprotein, putative
C0202	SAK0269	-		lipoprotein, putative
	GBS0201	-		hypothetical protein
	SAG0207	*cys*S	J	cysteinyl-tRNA synthetase
C0203	SAK0270	*cys*S	J	cysteinyl-tRNA synthetase
	GBS0202	*cys*S	J	cysteinyl-tRNA synthetase
	SAG0208	-	S	hypothetical protein
C0204	SAK0271	-	S	hypothetical protein
	GBS0203	-	S	hypothetical protein
	SAG0209	-	J	RNA methyltransferase, TrmH family, group 3
C0205	SAK0272	-	J	RNA methyltransferase, TrmH family
	GBS0204	-	J	hypothetical protein

(Continue on next page)

List of homolog clusters in the 3 GBS reference genomes (Cont'd)

ID	Locus tag	Gene	COG	Annotation
C0206	SAG0210	-		hypothetical protein
	SAK0273	-		hypothetical protein
	GBS0205	-		hypothetical protein
C0207	SAG0211	-	S	DegV family protein
	SAK0274	-	S	DegV family protein
	GBS0206	-	S	hypothetical protein
C0208	SAG0212	-		hypothetical protein
C0209	SAG0213	-		hypothetical protein
C0210	SAG0214	*rpl*M		50S ribosomal protein L13
	SAK0276	*rpl*M		50S ribosomal protein L13
	GBS0209	*rpl*M		50S ribosomal protein L13
C0211	SAG0215	*rps*I		30S ribosomal protein S9
	SAK0277	*rps*I		30S ribosomal protein S9
	GBS0210	*rps*I		30S ribosomal protein S9
C0212	SAG0216	-		hypothetical protein
C0214	SAG0218	-	K	transcriptional regulator, Cro/CI family
	GBS0475	-	K	hypothetical protein
C0215	SAG0219	-		hypothetical protein
	SAK0280	-		hypothetical protein
	GBS0213	-		hypothetical protein
C0216	SAG0220	-		hypothetical protein
	SAK0281	-		hypothetical protein
	GBS0214	-		hypothetical protein
C0217	SAG0221	-		hypothetical protein
C0218	SAG0222	-		hypothetical protein
	SAG1299	-		hypothetical protein
	SAK0282	-		replication initiation factor, RepA family
	GBS0215	-		hypothetical protein
	GBS0408	-		hypothetical protein
	GBS0738	-		hypothetical protein
	GBS0971	-		hypothetical protein
	GBS1149	-		hypothetical protein
	GBS1372	-		hypothetical protein
C0219	SAG0223	-	P	conserved hypothetical protein, fusion
	SAG1295	-	P	hypothetical protein
	SAK0283	-	P	hypothetical protein
	GBS0216	-	P	hypothetical protein
	GBS1368	-	P	hypothetical protein
C0220	SAG0224	-		replication initiation protein, putative
	SAK0284	-		replication initiation factor family protein
	GBS0217	-		hypothetical protein
C0221	SAG0225	-		hypothetical protein
	SAK0285	-		hypothetical protein
	GBS0218	-		hypothetical protein
C0222	SAG0226	-	D	recombination protein
	SAK0286	*pre*	D	plasmid recombination enzyme
	GBS0219	-	D	hypothetical protein
C0223	SAG0227	-		hypothetical protein
	SAK0287	-		hypothetical protein
	GBS0220	-		hypothetical protein
C0224	SAG0228	-		hypothetical protein
	SAK0288	-		RelE/ParE family protein
	GBS0221	-		hypothetical protein
C0225	SAG0229	-		hypothetical protein
	SAK0289	-		hypothetical protein
	GBS0222	-		hypothetical protein
C0226	SAG0230	-	S	hypothetical protein
	SAK0290	-	S	hypothetical protein
	SAK1956	-	S	hypothetical protein
	GBS0223	-	S	hypothetical protein
	GBS1979	-	S	hypothetical protein
C0227	SAG0231	-		hypothetical protein
	SAK0291	-		hypothetical protein
	GBS0224	-		hypothetical protein
	GBS0225	-		hypothetical protein
C0228	SAG0232	-		hypothetical protein
	SAK0292	-		hypothetical protein
	GBS0226	-		hypothetical protein

(Continue on next page)

List of homolog clusters in the 3 GBS reference genomes (Cont'd)

ID	Locus tag	Gene	COG	Annotation
C0229	SAG0233	-		hypothetical protein
	SAK0293	-		conserved hypothetical protein, truncation
	SAK1953	-		hypothetical protein
	GBS1967	-		hypothetical protein
C0230	SAG0234	-		hypothetical protein
	SAK0294	-		hypothetical protein
	GBS0228	-		hypothetical protein
	GBS1966	-		hypothetical protein
C0231	SAG0235	-		hypothetical protein
	SAK0295	-		hypothetical protein
	GBS0229	-		hypothetical protein
C0232	SAG0236	-		hypothetical protein
	SAK0296	-		hypothetical protein
C0233	SAG0237	-		hypothetical protein
	SAK0297	-		hypothetical protein
C0234	SAG0238	-		hypothetical protein
C0235	SAG0239	-		transcriptional regulator, MutR family
	SAK0298	-		transcriptional activator, Rgg/GadR/MutR family
	GBS0230	-		hypothetical protein
C0236	SAG0240	-		transporter, putative
	SAK0299	-		major facilitator family transporter
	GBS0231	-		hypothetical protein
C0237	SAG0241	-	E	amino acid ABC transporter, permease protein
	SAK0300	-	E	quaternary amine uptake (QAT) family ABC transporter, permease protein
	GBS0232	-	E	hypothetical protein
C0238	SAG0242	-	M	amino acid ABC transporter, amino acid-binding protein
	SAK0301	-	M	quaternary amine uptake (QAT) family ABC transporter, amino acid-binding protein
	GBS0233	-	M	hypothetical protein
C0239	SAG0243	-	E	amino acid ABC transporter, permease protein
	SAK0302	-	E	quaternary amine uptake (QAT) family ABC transporter, permease protein
	GBS0234	-	E	hypothetical protein
C0240	SAG0244	-	E	amino acid ABC transporter, ATP-binding protein
	SAK0303	-	E	quaternary amine uptake (QAT) family ABC transporter, ATP-binding protein
	GBS0235	-	E	hypothetical protein
C0241	SAG0246	-		hypothetical protein
	SAG0247	-		hypothetical protein
	SAK0322	-		hypothetical protein
	GBS0087	-		hypothetical protein
C0242	SAG0248	-		hypothetical protein
	SAK0323	-		hypothetical protein
C0243	SAG0249	-		hypothetical protein
	SAK0324	-		hypothetical protein
C0244	SAG0250	-		hypothetical protein
	SAK0325	-		hypothetical protein
C0245	SAG0252	-	J	acetyltransferase, GNAT family
	SAK0327	-	J	acetyltransferase, GNAT family
	GBS0245	-	J	hypothetical protein
C0246	SAG0253	-	J	acetyltransferase, GNAT family
	SAK0328	-	J	acetyltransferase, GNAT family
	GBS0246	-	J	hypothetical protein
C0247	SAG0254	-	J	acetyltransferase, GNAT family
	SAK0329	-	J	acetyltransferase, GNAT family
	GBS0247	-	J	hypothetical protein
C0248	SAG0255	-		hypothetical protein
	SAK0330	-		hypothetical protein
	GBS0248	-		hypothetical protein
C0249	SAG0256	-	K	RNA polymerase sigma factor, ECF subfamily
	SAK0331	-	K	RNA polymerase sigma-70 factor, ECF family
	GBS0249	-	K	hypothetical protein
C0250	SAG0258	-	K	transcriptional regulator, TetR family
	SAK0332	-	K	transcriptional regulator, TetR family
	GBS0250	-	K	hypothetical protein
C0251	SAG0259	-	V	ABC transporter efflux protein, DrrB family, putative
	SAK0333	-	V	ABC transporter, permease protein
	GBS0251	-	V	hypothetical protein
C0252	SAG0260	-	V	ABC transporter, ATP-binding protein
	SAK0334	-	V	ABC transporter, ATP-binding protein
	GBS0252	-	V	hypothetical protein

(Continue on next page)

List of homolog clusters in the 3 GBS reference genomes (Cont'd)

ID	Locus tag	Gene	COG	Annotation
C0253	SAG0261	-	L	IS1381 transposase protein B
	SAG0543	-	L	IS1381 transposase protein B
	SAG0966	-	L	IS1381 transposase protein B
	SAG1457	-	L	IS1381 transposase protein B
	SAG1550	-	L	IS1381 transposase protein B
	SAG2002	-	L	IS1381 transposase protein B
	SAK0190	-	L	IS1381 transposase protein B
	SAK0541	-	L	IS1381 transposase protein B
	SAK1061	-	L	IS1381 transposase protein B
	SAK1208	-	L	IS1381 transposase protein B
	SAK1969	-	L	IS1381 transposase protein B
	SAK2001	-	L	IS1381 transposase protein B
C0254	SAG0262	-		IS1381 transposase protein A
	SAG0542	-		IS1381 transposase protein A
	SAG0965	-		IS1381 transposase protein A
	SAG1458	-		IS1381 transposase protein A
	SAG1549	-		IS1381 transposase protein A
	SAG2003	-		IS1381 transposase protein A
	SAK0191	-		IS1381 transposase protein A
	SAK0540	-		IS1381 transposase protein A
	SAK1060	-		IS1381 transposase protein A
	SAK1209	-		IS1381 transposase protein A
	SAK1970	-		IS1381 transposase protein A
	SAK2000	-		IS1381 transposase protein A
C0255	SAG0263	-		hypothetical protein
	SAK0335	-		hypothetical protein
	GBS0253	-		hypothetical protein
C0256	SAG0264	-	K	hypothetical protein
	SAK0336	-	K	transcriptional regulator, PadR family
	GBS0254	-	K	hypothetical protein
C0257	SAG0265	-		hypothetical protein
	SAK0337	-		hypothetical protein
	GBS0255	-		hypothetical protein
C0258	SAG0266	*nag*A	G	N-acetylglucosamine-6-phosphate deacetylase
	SAK0338	*nag*A	G	N-acetylglucosamine-6-phosphate deacetylase
	GBS0256	-	G	hypothetical protein
C0259	SAG0267	-		hypothetical protein
	SAK0187	-		isoprenylcysteine carboxyl methyltransferase (ICMT) family protein
	SAK0339	-		isoprenylcysteine carboxyl methyltransferase (ICMT) family protein
	GBS0257	-		hypothetical protein
C0260	SAG0268	*gly*Q		glycyl-tRNA synthetase alpha subunit
	SAK0340	*gly*Q		glycyl-tRNA synthetase alpha subunit
	GBS0258	*gly*Q		glycyl-tRNA synthetase alpha subunit
C0261	SAG0269	-	I	acyl carrier protein phosphodiesterase, putative
	SAK0341	-	I	acyl carrier protein phosphodiesterase, putative
	GBS0259	-	I	hypothetical protein
C0262	SAG0270	*gly*S	J	glycyl-tRNA synthetase beta subunit
	SAK0342	*gly*S	J	glycyl-tRNA synthetase beta subunit
	GBS0260	*gly*S	J	glycyl-tRNA synthetase beta subunit
C0263	SAG0271	-		hypothetical protein
	SAK0343	-		hypothetical protein
	GBS0261	-		hypothetical protein
C0264	SAG0272	-		hypothetical protein
	SAK0344	-		hypothetical protein
	GBS0262	-		hypothetical protein
C0265	SAG0273	*glp*K	C	glycerol kinase
	SAK0345	*glp*K	C	glycerol kinase
	GBS0263	*glp*K	C	glycerol kinase
C0266	SAG0274	-	C	alpha-glycerophosphate oxidase
	SAK0346	*glp*O	C	glycerol-3-phosphate oxidase
	GBS0264	*glp*D	C	glycerol-3-phosphate dehydrogenase
C0267	SAG0275	*glp*F-1		glycerol uptake facilitator protein
	SAK0347	*glp*F		glycerol uptake facilitator protein
	GBS0265	*glp*F		hypothetical protein
C0268	SAG0276	-	R	NADH oxidase, putative
	SAK0348	-	R	pyridine nucleotide-disulfide oxidoreductase
	GBS0266	-	R	hypothetical protein
C0269	SAG0277	-		hypothetical protein
	SAK0349	-		hypothetical protein
	GBS0267	-		hypothetical protein

(Continue on next page)

List of homolog clusters in the 3 GBS reference genomes (Cont'd)

ID	Locus tag	Gene	COG	Annotation
C0270	SAG0278	*tkt*	G	transketolase
	SAK0350	*tkt*	G	transketolase
	SAK1756	*tkt*	G	transketolase
	GBS0268	-	G	transketolase
C0271	SAG0279	-		hypothetical protein
	SAK0351	-		hypothetical protein
	GBS0269	-		hypothetical protein
C0272	SAG0280	-	V	ABC transporter, ATP-binding protein
	SAK0352	-	V	ABC transporter, ATP-binding protein
	GBS0270	-	V	hypothetical protein
C0273	SAG0281	-		hypothetical protein
	SAK0353	-		hypothetical protein
	GBS0271	-		hypothetical protein
C0274	SAG0282	-	G	PTS system, IIBC components
	SAK0354	-	G	PTS system, IIBC component
	GBS0272	-	G	hypothetical protein
C0275	SAG0283	*pro*B	E	gamma-glutamyl kinase
	SAK0355	*pro*B	E	gamma-glutamyl kinase
	GBS0273	*pro*B	E	gamma-glutamyl kinase
C0276	SAG0284	*pro*A	E	gamma-glutamyl phosphate reductase
	SAK0356	*pro*A	E	gamma-glutamyl phosphate reductase
	GBS0274	*pro*A	E	gamma-glutamyl phosphate reductase
C0277	SAG0285	*mra*W		S-adenosyl-methyltransferase MraW
	SAK0357	*mra*W		S-adenosyl-methyltransferase MraW
	GBS0275	*mra*W		S-adenosyl-methyltransferase MraW
C0278	SAG0286	-		cell division protein FtsL, putative
	SAK0358	-		cell division protein FtsL, putative
	GBS0276	*fts*L		hypothetical protein
C0279	SAG0287	*pbp*X	M	penicillin-binding protein 2X
	SAK0359	*pbp*X	M	penicillin-binding protein 2X
	GBS0277	-	M	hypothetical protein
C0280	SAG0288	*mra*Y	M	phospho-N-acetylmuramoyl-pentapeptide-transferase
	SAK0360	*mra*Y	M	phospho-N-acetylmuramoyl-pentapeptide-transferase
	GBS0278	*mra*Y	M	phospho-N-acetylmuramoyl-pentapeptide-transferase
C0281	SAG0289	-	LKJ	ATP-dependent RNA helicase, DEAD/DEAH box family
	SAK0361	-	LKJ	ATP-dependent RNA helicase, DEAD/DEAH box family
	GBS0279	-	LKJ	hypothetical protein
C0282	SAG0290	-	ET	ABC transporter, substrate-binding protein
	SAK0362	-	ET	polar amino acid uptake (PAAT) family ABC transporter, amino acid-binding protein
	GBS0280	-	ET	hypothetical protein
C0283	SAG0291	-	E	amino acid ABC transporter, permease protein
	SAK0363	-	E	polar amino acid uptake (PAAT) family ABC transporter, permease protein
	GBS0281	-	E	hypothetical protein
C0284	SAG0292	-	E	amino acid ABC transporter, ATP-binding protein
	SAK0364	-	E	polar amino acid uptake (PAAT) family ABC transporter, ATP-binding protein
	GBS0282	-	E	hypothetical protein
C0285	SAG0293	-		hypothetical protein
	SAK0365	-		hypothetical protein
	GBS0283	-		hypothetical protein
C0286	SAG0294	*trx*B	O	thioredoxin reductase
	SAK0366	*trx*B	O	thioredoxin-disulfide reductase
	GBS0284	-	O	hypothetical protein
C0287	SAG0295	-	H	nicotinate phosphoribosyltransferase
	SAK0367	-	H	nicotinate phosphoribosyltransferase
	GBS0285	-	H	nicotinate phosphoribosyltransferase
C0288	SAG0296	*nad*E	H	NAD(+) synthetase
	SAK0368	*nad*E	H	NAD(+) synthetase
	GBS0286	*nad*E	H	NAD(+) synthetase
C0289	SAG0297	*pep*C	E	aminopeptidase C
	SAK0369	*pep*C	E	aminopeptidase C
	GBS0287	*pep*C	E	hypothetical protein
C0290	SAG0298	*pbp*1A	M	penicillin-binding protein 1A
	SAK0370	-	M	pencillin-binding protein, 1A family
	GBS0288	-	M	hypothetical protein
C0291	SAG0299	*rec*U	R	hypothetical protein
	SAK0371	*rec*U	R	hypothetical protein
	GBS0289	-	R	hypothetical protein
C0292	SAG0300	-		hypothetical protein
	SAK0372	-		hypothetical protein
	GBS0290	-		hypothetical protein

(Continue on next page)

List of homolog clusters in the 3 GBS reference genomes (Cont'd)

ID	Locus tag	Gene	COG	Annotation
C0293	SAG0301	-		hypothetical protein
C0294	SAG0302	-	D	hypothetical protein
	SAK0373	-	D	DivIVA domain protein
	GBS0291	-	D	hypothetical protein
C0295	SAG0303	-	L	hypothetical protein
	SAK0374	-	L	hypothetical protein
	GBS0292	-	L	hypothetical protein
C0296	SAG0304	-		hypothetical protein
	SAK0375	-		hypothetical protein
	GBS0293	-		hypothetical protein
C0297	SAG0305	*lux*S		S-ribosylhomocysteinase
	SAK0376	*lux*S		S-ribosylhomocysteinase
	GBS0294	-		S-ribosylhomocysteinase
C0298	SAG0306	-	R	hypothetical protein
	SAK0377	-	R	hypothetical protein
	GBS0295	-	R	hypothetical protein
C0299	SAG0307	-		hypothetical protein
C0300	SAG0308	-	V	ABC transporter, ATP-binding protein
	SAK0378	-	V	ABC transporter, ATP-binding protein
	GBS0296	-	V	hypothetical protein
C0301	SAG0309	-	V	ABC transporter, permease protein, putative
	SAK0379	-	V	hypothetical protein
	GBS0297	-	V	hypothetical protein
C0302	SAG0310	-	T	hypothetical protein
	SAK0380	-	T	sensor histidine kinase
	GBS0298	-	T	hypothetical protein
C0303	SAG0312	-		hypothetical protein
	SAK0382	-		hypothetical protein
	GBS0300	-		hypothetical protein
C0304	SAG0313	*gmk*	F	guanylate kinase
	SAK0383	*gmk*	F	guanylate kinase
	GBS0301	-	F	guanylate kinase
C0305	SAG0314	-	K	DNA-directed RNA polymerase omega subunit
	SAK0384	*rpo*Z	K	DNA-directed RNA polymerase omega subunit
	GBS0302	-	K	DNA-directed RNA polymerase omega subunit
C0306	SAG0315	*pri*A	L	primosome assembly protein PriA
	SAK0385	*pri*A	L	primosome assembly protein PriA
	GBS0303	*pri*A	L	primosome assembly protein PriA
C0307	SAG0316	*fmt*	J	methionyl-tRNA formyltransferase
	SAK0386	*fmt*	J	methionyl-tRNA formyltransferase
	GBS0304	*fmt*	J	methionyl-tRNA formyltransferase
C0308	SAG0317	*sun*	J	sun protein
	SAK0387	*sun*	J	sun protein
	GBS0305	-	J	hypothetical protein
C0309	SAG0318	-	T	serine/threonine phosphatase, putative
	SAK0388	*stp*1	T	serine/threonine protein phosphatase Stp1
	GBS0306	-	T	hypothetical protein
C0310	SAG0319	-	S	serine/threonine protein kinase
	SAK0389	*stk*1	S	serine/threonine protein kinase Stk1
	SAK0766	-	RTKL	serine/threonine protein kinase, putative
	GBS0307	-	RTKL	hypothetical protein
C0311	SAG0320	-	S	hypothetical protein
	SAK0390	-	S	hypothetical protein
	GBS0308	-	S	hypothetical protein
C0312	SAG0321	-	T	sensor histidine kinase, putative
	SAK0391	-	T	sensor histidine kinase
	GBS0309	-	T	hypothetical protein
C0313	SAG0322	-	TK	DNA-binding response regulator
	SAK0381	-	TK	DNA-binding response regulator, LuxR family
	SAK0392	-	TK	DNA-binding response regulator, LuxR family
	GBS0299	-	TK	hypothetical protein
	GBS0310	-	TK	hypothetical protein
C0314	SAG0323	-	O	hydrolase, haloacid dehalogenase family/peptidyl-prolyl cis-trans isomerase, cyclophilin type
	SAK0393	-	R	Cof-like hydrolase/peptidyl-prolyl cis-trans isomerase domain protein
	GBS0311	-	O	hypothetical protein
C0315	SAG0324	-	J	hypothetical protein
	SAK0394	-	J	hypothetical protein
	GBS0312	-	J	hypothetical protein

(Continue on next page)

List of homolog clusters in the 3 GBS reference genomes (Cont'd)

ID	Locus tag	Gene	COG	Annotation
C0316	SAG0325	*pfl*A-1	O	pyruvate formate-lyase-activating enzyme
	SAK0395	-	O	pyruvate formate-lyase-activating enzyme, putative
	GBS0313	-	O	hypothetical protein
C0317	SAG0326	-	KG	transcriptional regulator, DeoR family
	SAK0396	-	KG	transcriptional regulator, DeoR family
	GBS0314	-	KG	hypothetical protein
C0318	SAG0327	-	K	transcriptional regulator, putative
	SAK0397	-	K	transcriptional regulator, SorC family
	GBS0315	-	K	hypothetical protein
C0319	SAG0328	*cel*C		PTS system, cellobiose-specific IIA component
	SAK0398	-		PTS system, IIA component, lactose/cellobiose family
	GBS0316	-		hypothetical protein
	GBS1331	-		hypothetical protein
C0320	SAG0329	-	G	PTS system, cellobiose-specific IIB component
	SAK0399	-	G	PTS system, IIB component, lactose/cellobiose family
	GBS0317	-	G	hypothetical protein
C0321	SAG0330	*cel*B	G	PTS system, cellobiose-specific IIC component
	SAK0400	-	G	PTS system, IIC component, lactose/cellobiose family
	GBS0318	-	G	hypothetical protein
	GBS1330	-	G	hypothetical protein
C0322	SAG0331	*pfl*D-1	C	formate acetyltransferase
	SAK0401	-	C	formate acetyltransferase 2
	GBS0319	-	C	hypothetical protein
C0323	SAG0332	-		fructose-6-phosphate aldolase
	SAK0402	-		fructose-6-phosphate aldolase
	GBS0320	-		fructose-6-phosphate aldolase
C0324	SAG0333	*gld*A	C	glycerol dehydrogenase
	SAK0403	*gld*A	C	glycerol dehydrogenase
	GBS0321	*gld*A	C	glycerol dehydrogenase
C0325	SAG0334	*cys*K	E	cysteine synthase A
	SAK0404	-	E	cysteine synthase/cystathionine beta-synthase family protein
	GBS0322	*cys*K	E	hypothetical protein
C0326	SAG0335	-	S	conserved hypothetical protein TIGR00257
	SAK0405	-	S	conserved hypothetical protein TIGR00257
	GBS0323	-	S	hypothetical protein
C0327	SAG0336	-	L	helicase, putative
	SAK0406	-	L	competence protein ComFA, putative
	GBS0324	*com*FA	L	competence protein ComFA
C0328	SAG0337	-	R	competence protein F, putative
	SAK0407	-	R	competence protein ComFC, putative
	GBS0325	*com*FC	R	hypothetical protein
C0329	SAG0338	*yfi*A	J	ribosomal subunit interface protein
	SAK0408	*yfi*A	J	ribosomal subunit interface protein
	GBS0326	-	J	hypothetical protein
C0330	SAG0339	-	E	aspartate kinase
	SAK0414	-	E	aspartate kinase
	GBS0327	-	E	aspartate kinase
C0331	SAG0340	-	R	hydrolase, haloacid dehalogenase-like family
	SAK0415	-	R	HAD-superfamily hydrolase, subfamily IA, variant 3 family protein
	GBS0328	-	R	hypothetical protein
C0332	SAG0341	-		hypothetical protein
C0333	SAG0342	-	I	enoyl-CoA hydratase
	SAK0416	-	I	enoyl-CoA hydratase
	GBS0329	-	I	enoyl-CoA hydratase
C0334	SAG0343	-	K	transcriptional regulator, MarR family
	SAK0417	-	K	transcriptional regulator, MarR family
	GBS0330	-	K	hypothetical protein
C0335	SAG0344	*fab*H	I	3-oxoacyl-(acyl carrier protein) synthase
	SAK0418	*fab*H	I	3-oxoacyl-(acyl carrier protein) synthase
	GBS0331	-	I	3-oxoacyl-(acyl carrier protein) synthase
C0336	SAG0345	*acp*P	IQ	acyl carrier protein
	SAK0419	*acp*P	IQ	acyl carrier protein
	GBS0332	-	IQ	acyl carrier protein
C0337	SAG0346	*fab*K	R	enoyl-(acyl-carrier-protein) reductase II
	SAK0420	*fab*K	R	enoyl-(acyl-carrier-protein) reductase II
	GBS0333	-	R	hypothetical protein
C0338	SAG0347	*fab*D	I	acyl-carrier-protein S-malonyltransferase
	SAK0421	*fab*D	I	acyl-carrier-protein S-malonyltransferase
	GBS0334	-	I	acyl-carrier-protein S-malonyltransferase

(Continue on next page)

List of homolog clusters in the 3 GBS reference genomes (Cont'd)

ID	Locus tag	Gene	COG	Annotation
	SAG0348	*fab*G	IQR	3-ketoacyl-(acyl-carrier-protein) reductase
	SAG1904	-	IQR	gluconate 5-dehydrogenase
C0339	SAK0422	*fab*G	IQR	3-ketoacyl-(acyl-carrier-protein) reductase
	GBS0335	*fab*G	IQR	3-ketoacyl-(acyl-carrier-protein) reductase
	GBS1891	-	IQR	gluconate 5-dehydrogenase
	SAG0349	*fab*F	IQ	3-oxoacyl-(acyl carrier protein) synthase
C0340	SAK0423	*fab*F	IQ	3-oxoacyl-(acyl carrier protein) synthase
	GBS0336	-	IQ	3-oxoacyl-(acyl carrier protein) synthase
	SAG0350	*acc*B	I	acetyl-CoA carboxylase
C0341	SAK0424	*acc*B	I	acetyl-CoA carboxylase
	GBS0337	-	I	acetyl-CoA carboxylase
	SAG0351	*fab*Z	I	(3R)-hydroxymyristoyl ACP dehydratase
C0342	SAK0425	*fab*Z	I	(3R)-hydroxymyristoyl ACP dehydratase
	GBS0338	*fab*Z	I	(3R)-hydroxymyristoyl ACP dehydratase
	SAG0352	*acc*C	I	acetyl-CoA carboxylase
C0343	SAK0426	*acc*C	I	acetyl-CoA carboxylase
	GBS0339	-	I	acetyl-CoA carboxylase
	SAG0353	*acc*D	I	acetyl-CoA carboxylase beta subunit
C0344	SAK0427	*acc*D	I	acetyl-CoA carboxylase beta subunit
	GBS0340	-	I	acetyl-CoA carboxylase beta subunit
	SAG0354	*acc*A	I	acetyl-CoA carboxylase alpha subunit
C0345	SAK0428	*acc*A	I	acetyl-CoA carboxylase alpha subunit
	GBS0341	-	I	acetyl-CoA carboxylase alpha subunit
	SAG0355	-		hypothetical protein
C0346	SAK0429	-		hypothetical protein
	GBS0342	-		hypothetical protein
	SAG0356	*ser*S	J	seryl-tRNA synthetase
C0347	SAK0430	*ser*S	J	seryl-tRNA synthetase
	GBS0343	*ser*S	J	seryl-tRNA synthetase
	SAG0357	-	S	hypothetical protein
C0348	SAK0431	-	S	hypothetical protein
	GBS0344	-	S	hypothetical protein
	SAG0358	-		hypothetical protein
C0349	SAK0432	-		hypothetical protein
	GBS0345	-		hypothetical protein
	SAG0359	-		PTS system, mannose-specific IID component
	SAG1898	-		PTS system, IID component
C0350	SAK0433	-		PTS system, IID component, mannose/fructose/sorbose family
	GBS0346	-		hypothetical protein
	GBS1886	-		hypothetical protein
	SAG0360	*man*M		PTS system, mannose-specific IIC component
C0351	SAK0434	-		PTS system, IIC component, mannose/fructose/sorbose family
	GBS0347	-		hypothetical protein
	SAG0361	*man*L	G	PTS system, mannose-specific IIAB components
	SAG1900	-		PTS system, IIB component
	SAG1902	-	G	PTS system, IIA component
C0352	SAK0435	-	G	PTS system, IIAB component, mannose/fructose/sorbose family
	GBS0348	-	G	hypothetical protein
	GBS1888	-		hypothetical protein
	GBS1890	-	G	hypothetical protein
	SAG0362	-	R	hydrolase, haloacid dehalogenase-like family
C0353	SAK0436	-	R	Cof-like hydrolase
	GBS0349	-	R	hypothetical protein
	SAG0363	-		hypothetical protein
C0354	SAK0437	-		hypothetical protein
	GBS0350	-		hypothetical protein
	SAG0364	-		hypothetical protein
C0355	SAK0438	-		hypothetical protein
	GBS0351	-		hypothetical protein
	SAG0365	-	R	xanthine/uracil permease family protein
C0356	SAK0439	-	R	purine transporter, AzgA family
	GBS0352	-	R	hypothetical protein
	SAG0366	-	R	conserved hypothetical protein TIGR00150
C0357	SAK0440	-	R	conserved hypothetical protein TIGR00150
	GBS0353	-	R	hypothetical protein
	SAG0367	-		acetyltransferase, GNAT family
C0358	SAK0441	-		acetyltransferase, GNAT family
	GBS0354	-		hypothetical protein
	SAG0368	-	K	hypothetical protein
C0359	SAK0442	-	K	transcriptional regulator, putative
	GBS0355	-	K	hypothetical protein

(Continue on next page)

List of homolog clusters in the 3 GBS reference genomes (Cont'd)

ID	Locus tag	Gene	COG	Annotation
C0360	SAG0369	-		hypothetical protein
	SAK0443	-		hypothetical protein
	GBS0356	-		hypothetical protein
C0361	SAG0370	*hit*	FGR	HIT family protein
	SAK0444	-	FGR	HIT family protein
	GBS0357	-	FGR	hypothetical protein
C0362	SAG0371	-		hypothetical protein
	SAK0445	-		hypothetical protein
	GBS0358	-		hypothetical protein
C0363	SAG0372	-		hypothetical protein
C0364	SAG0373	-	V	ABC transporter, ATP-binding protein
	SAK0446	*ecs*A	V	ABC transporter, ATP-binding protein
	GBS0359	-	V	hypothetical protein
C0365	SAG0374	-		ABC transporter, permease protein
	SAK0447	-		ABC transporter, permease protein EcsB, putative
	GBS0360	-		hypothetical protein
C0366	SAG0375	-	M	hypothetical protein
	SAK0448	-	M	hypothetical protein
	GBS0411	-	M	hypothetical protein
C0367	SAG0376	-	R	tRNA (guanine-N(7)-)-methyltransferase
	SAK0449	*trm*B	R	tRNA (guanine-N(7)-)-methyltransferase
	GBS0412	-	R	tRNA (guanine-N(7)-)-methyltransferase
C0368	SAG0377	-	S	hypothetical protein
	SAK0451	-	S	hypothetical protein
	GBS0413	-	S	hypothetical protein
C0369	SAG0378	*nus*A	K	transcription elongation factor NusA
	SAK0452	*nus*A	K	transcription elongation factor NusA
	GBS0414	*nus*A	K	transcription elongation factor NusA
C0370	SAG0379	-	K	hypothetical protein
	SAK0453	*ylx*R	K	cytosolic protein YlxR
	GBS0415	-	K	hypothetical protein
C0371	SAG0380	-	J	hypothetical protein
	SAK0454	-	J	hypothetical protein
	GBS0416	-	J	hypothetical protein
C0372	SAG0381	*inf*B	J	translation initiation factor IF-2
	SAK0455	*inf*B	J	translation initiation factor IF-2
	GBS0417	*inf*B	J	translation initiation factor IF-2
C0373	SAG0382	*rbf*A	J	ribosome-binding factor A
	SAK0456	*rbf*A	J	ribosome-binding factor A
	GBS0418	*rbf*A	J	ribosome-binding factor A
C0374	SAG0383	-	I	protein of unknown function/lipoprotein, putative
	SAK0457	-	I	GDXG lipolytic enzyme family protein
	GBS0419	-	I	hypothetical protein
C0375	SAG0384	-	K	transcriptional repressor CopY
	SAG1264	-	K	transcriptional repressor CopY, putative
	SAK0458	*cop*Y	K	transcriptional repressor CopY
	GBS0420	-	K	hypothetical protein
C0376	SAG0385	-	P	copper-transporter ATPase CopA
	SAG1257	-	P	cation-transporting ATPase, E1-E2 family
	SAG1262	-	P	cation-transporting ATPase, E1-E2 family
	SAK0459	*cop*A	P	copper-translocating P-type ATPase
	GBS0421	-	P	hypothetical protein
C0377	SAG0386	-	P	copper-transporter protein CopZ
	SAK0460	-	P	copper transporter, copper-binding protein, putative
	GBS0422	-	P	hypothetical protein
C0378	SAG0387	-	S	hypothetical protein
	SAK0461	-	S	hypothetical protein
	GBS0423	-	S	hypothetical protein
C0379	SAG0388	-	R	hydrolase, haloacid dehalogenase-like family
	SAK0462	-	R	Cof-like hydrolase
	GBS0424	-	R	hypothetical protein
C0380	SAG0389	*pol*A	L	DNA polymerase I
	SAK0463	*pol*A	L	DNA polymerase I
	GBS0425	*pol*A	L	DNA polymerase I
C0381	SAG0390	-	R	CoA-binding domain protein
	SAK0464	-	R	CoA binding domain protein
	GBS0426	-	R	hypothetical protein
C0382	SAG0391	-	P	transcriptional regulator, Fur family
	SAK0465	-	P	transcriptional regulator, Fur family
	GBS0427	-	P	hypothetical protein

(Continue on next page)

List of homolog clusters in the 3 GBS reference genomes (Cont'd)

ID	Locus tag	Gene	COG	Annotation
C0383	SAG0392	-		cell wall surface anchor family protein
	SAK0466	-		cell wall surface anchor family protein
	GBS0428	-		hypothetical protein
C0384	SAG0393	-	TK	DNA-binding response regulator
	SAK0467	-	TK	DNA-binding response regulator
	GBS0429	-	TK	similar to two-component response regulator
C0385	SAG0394	-	T	sensor histidine kinase
	SAK0468	-	T	sensor histidine kinase
	GBS0430	-	T	hypothetical protein
C0386	SAG0395	-	S	hypothetical protein
	SAK0469	-	S	hypothetical protein
	GBS0431	-	S	hypothetical protein
C0387	SAG0396	*tgt*	J	queuine tRNA-ribosyltransferase
	SAK0470	*tgt*	J	queuine tRNA-ribosyltransferase
	GBS0432	*tgt*	J	queuine tRNA-ribosyltransferase
C0388	SAG0397	-	S	hypothetical protein
	SAK0471	-	S	CHY zinc finger family protein
	GBS0433	-	S	hypothetical protein
C0389	SAG0398	-	R	BioY family protein
	SAK0472	-	R	BioY family protein
	GBS0434	-	R	hypothetical protein
C0390	SAG0399	-	R	AtsA/ElaC family protein
	SAK0473	-	R	metallo-beta-lactamase family protein
	GBS0435	-	R	hypothetical protein
C0391	SAG0400	-	FJ	cytidine/deoxycytidylate deaminase family protein
	SAK0474	-	FJ	cytidine/deoxycytidylate deaminase family protein
	GBS0436	-	FJ	hypothetical protein
C0392	SAG0401	-		hypothetical protein
C0393	SAG0402	*pgi*	G	glucose-6-phosphate isomerase
	SAK0475	*pgi*	G	glucose-6-phosphate isomerase
	GBS0437	*pgi*	G	glucose-6-phosphate isomerase
C0394	SAG0403	-		5-formyltetrahydrofolate cyclo-ligase family protein
	SAK0476	-		5-formyltetrahydrofolate cyclo-ligase family protein
	GBS0438	-		hypothetical protein
C0395	SAG0404	-	R	rhomboid family protein
	SAK0477	-	R	rhomboid family protein
	GBS0439	-	R	hypothetical protein
C0396	SAG0405	-	R	protein of unknown function/lipoprotein, putative
	SAK0478	-	R	bmp family protein
	GBS0440	-	R	hypothetical protein
C0397	SAG0406	*gal*U	M	UTP-glucose-1-phosphate uridylyltransferase
	SAK0479	*gal*U	M	UTP-glucose-1-phosphate uridylyltransferase
	GBS0441	-	M	hypothetical protein
C0398	SAG0407	*gps*A	C	NAD(P)H-dependent glycerol-3-phosphate dehydrogenase
	SAK0480	*gps*A	C	NAD(P)H-dependent glycerol-3-phosphate dehydrogenase
	GBS0442	*gps*A	C	NAD(P)H-dependent glycerol-3-phosphate dehydrogenase
C0399	SAG0408	*rnp*A	J	ribonuclease P
	SAK0481	*rnp*A	J	ribonuclease P
	GBS0443	*rnp*A	J	ribonuclease P
C0400	SAG0409	-	U	SpoIIIJ family protein
	SAK0482	-	U	membrane protein oxaA, putative
	GBS0444	-	U	hypothetical protein
C0401	SAG0410	-	R	R3H domain protein
	SAK0483	-	R	R3H domain protein
	GBS0445	-	R	hypothetical protein
C0402	SAG0411	-	J	hypothetical protein
	SAK0492	-	J	hypothetical protein
	GBS0446	-	J	hypothetical protein
C0403	SAG0412	*rec*X	R	RecA regulator RecX
	SAK0493	*rec*X	R	RecA regulator RecX
	GBS0447	*rec*X	R	RecA regulator RecX
C0404	SAG0413	-	J	RNA methyltransferase, TrmA family
	SAK0494	*rum*A	J	23S rRNA (uracil-5-)-methyltransferase RumA
	GBS0448	-	J	hypothetical protein
C0405	SAG0414	-	F	hypothetical protein
	SAK0495	-	F	hypothetical protein
	GBS0449	-	F	hypothetical protein
C0406	SAG0415	-	R	acetyltransferase, GNAT family
	SAK0496	-	R	acetyltransferase, GNAT family
	GBS0450	-	R	hypothetical protein

(Continue on next page)

List of homolog clusters in the 3 GBS reference genomes (Cont'd)

ID	Locus tag	Gene	COG	Annotation
C0407	SAG0416	-	O	protease, putative
	SAK1320	*scp*B	O	C5a peptidase ScpB
	GBS0451	-	O	hypothetical protein
	GBS1308	*scp*B	O	streptococcal C5a peptidase
C0408	SAG0417	-	M	glycosyl transferase, group 2 family protein
	SAK0498	-	M	glycosyl transferase, group 2 family protein
	GBS0452	-	M	hypothetical protein
C0409	SAG0418	*nrd*F-1	F	ribonucleotide-diphosphate reductase beta subunit
	SAK0499	*nrd*F	F	ribonucleotide-diphosphate reductase beta subunit
	GBS0453	-	F	ribonucleotide-diphosphate reductase beta subunit
C0410	SAG0419	*nrd*I-1	F	hypothetical protein
	SAK0500	*nrd*I	F	hypothetical protein
	GBS0454	-	F	hypothetical protein
C0411	SAG0420	*nrd*E-1	F	ribonucleotide-diphosphate reductase alpha subunit
	SAG0819	*nrd*E-2	F	ribonucleotide-diphosphate reductase alpha subunit
	SAK0501	-	F	ribonucleotide-diphosphate reductase alpha subunit
	SAK0943	-	F	ribonucleotide-diphosphate reductase alpha subunit
	GBS0455	-	F	ribonucleotide-diphosphate reductase alpha subunit
	GBS0837	-	F	ribonucleotide-diphosphate reductase alpha subunit
C0412	SAG0421	-		cell wall surface anchor family protein
	SAK0502	-		hypothetical protein
	GBS0456	-		hypothetical protein
C0413	SAG0422	-	R	hypothetical protein
	SAK0503	-	R	pyridoxamine 5'-phosphate oxidase family
	GBS0457	-	R	hypothetical protein
C0414	SAG0423	-	S	hypothetical protein
	SAK0506	-	S	hypothetical protein
	GBS0458	-	S	hypothetical protein
C0415	SAG0424	-	S	hypothetical protein
	SAK0507	-		hypothetical protein
	SAK0508	-		hypothetical protein
	GBS0459	-	S	hypothetical protein
C0416	SAG0425	-	S	carboxymuconolactone decarboxylase family protein
	SAK0509	-	S	4-carboxymuconolactone decarboxylase, putative
	GBS0460	-	S	hypothetical protein
C0417	SAG0426	-	S	hypothetical protein
	SAK0510	-	S	cupin domain protein
	GBS0461	-	S	hypothetical protein
C0418	SAG0427	-	K	transcriptional regulator, MerR family
	SAG1255	*mer*R-1	K	mercuric resistance operon regulatory protein MerR
	SAG2024	*mer*R-2	K	mercuric resistance operon regulatory protein MerR
	GBS0462	-	K	hypothetical protein
C0419	SAG0428	-	ER	alcohol dehydrogenase, zinc-containing
	SAG1637	*adh*	ER	alcohol dehydrogenase, zinc-containing
	SAK1651	-	ER	alcohol dehydrogenase, zinc-containing
	GBS0464	-	ER	hypothetical protein
	GBS1684	-	ER	hypothetical protein
C0420	SAG0429	-	R	oxidoreductase, aldo/keto reductase family
	SAK0513	-	R	oxidoreductase, aldo/keto reductase family
	GBS0466	-	R	hypothetical protein
C0421	SAG0430	-	P	cation efflux system protein
	SAK0514	-	P	cation efflux transporter, cation diffusion facilitator (CDF) family
	GBS0467	-	P	hypothetical protein
C0422	SAG0431	-	K	transcriptional regulator, TetR family
	SAK0515	-	K	transcriptional regulator, TetR family
	SAK0721	-	K	transcriptional regulator, TetR family
	GBS0468	-	K	hypothetical protein
	GBS0618	-	K	hypothetical protein
C0423	SAG0432	-	T	transcriptional regulator, AraC family
	SAK0516	-	T	transcriptional regulator, AraC family
	GBS0469	-	T	hypothetical protein
C0424	SAG0433	-		surface protein Rib
	SAK0186	*bag*	D	IgA-binding beta antigen
	SAK0517	-		C protein alpha-antigen
	SAK0722	-		collagen-like surface protein, putative
	SAK0771	-		cell wall surface anchor family protein, truncation
	GBS0470	-		hypothetical protein
	GBS0619	-		hypothetical protein

(Continue on next page)

List of homolog clusters in the 3 GBS reference genomes (Cont'd)

ID	Locus tag	Gene	COG	Annotation
C0425	SAG0435	-	L	DNA-damage-inducible protein J, putative
	SAK0743	-	L	prophage LambdaSa04, RelB antitoxin family protein
	GBS0471	-	L	hypothetical protein
	GBS1982	-	L	hypothetical protein
C0426	SAG0436	-		hypothetical protein
	GBS0472	-		hypothetical protein
C0427	SAG0437	-		lipoprotein, putative
	GBS0473	-		hypothetical protein
C0428	SAG0440	-		hypothetical protein
	SAK0543	-		hypothetical protein
	GBS0487	-		hypothetical protein
C0429	SAG0441	-		hypothetical protein
	SAK0544	-		hypothetical protein
	GBS0488	-		hypothetical protein
C0430	SAG0442	-	J	acetyltransferase, GNAT family
	SAG0443	-	J	acetyltransferase, GNAT family
	SAK0545	-	J	acetyltransferase, GNAT family
	GBS0489	-	J	hypothetical protein
	GBS0490	-	J	hypothetical protein
C0431	SAG0444	-	E	hypothetical protein
	GBS0491	-	E	hypothetical protein
C0432	SAG0445	*val*S	J	valyl-tRNA synthetase
	SAK0547	*val*S	J	valyl-tRNA synthetase
	GBS0492	-	J	valyl-tRNA synthetase
C0433	SAG0446	-	R	oxidoreductase, Gfo/Idh/MocA family
	SAK0549	-	R	oxidoreductase, Gfo/Idh/MocA family
	GBS0494	-	R	hypothetical protein
C0434	SAG0447	-	P	magnesium transporter, CorA family
	SAK0550	-	P	metal ion transporter, CorA family
	GBS0495	-	P	hypothetical protein
C0435	SAG0448	-	L	transposase, IS256 family
C0436	SAG0449	-	S	hypothetical protein
	SAK0551	-	S	hypothetical protein
	GBS0496	-	S	hypothetical protein
C0437	SAG0450	*asn*A	E	asparagine synthetase AsnA
	SAK0552	*asn*A	E	asparagine synthetase AsnA
	GBS0497	-	E	asparagine synthetase AsnA
C0438	SAG0451	-		bacteriocin transport accessory protein, putative
	SAK0553	-		hypothetical protein
	GBS0498	-		hypothetical protein
C0439	SAG0452	-	L	type II DNA modification methyltransferase, putative
	SAK0554	-	L	methyltransferase, putative
	GBS0499	-	L	hypothetical protein
C0440	SAG0453	-		hypothetical protein
	GBS0500	-		hypothetical protein
C0441	SAG0454	*coa*D	H	phosphopantetheine adenylyltransferase
	SAK0555	*coa*D	H	phosphopantetheine adenylyltransferase
	GBS0501	-	H	phosphopantetheine adenylyltransferase
C0442	SAG0455	-	T	hypothetical protein
	SAK0556	-	T	hypothetical protein
	GBS0502	-	T	hypothetical protein
C0443	SAG0457	-		hypothetical protein
	SAK0558	-		hypothetical protein
	GBS0504	-		hypothetical protein
C0444	SAG0458	-	R	conserved hypothetical protein TIGR00048
	SAK0559	-	R	radical SAM enzyme, Cfr family
	GBS0505	-	R	hypothetical protein
C0445	SAG0459	-	V	VanZF domain protein
	SAK0560	-	V	VanZ family protein
	GBS0506	-	V	hypothetical protein
C0446	SAG0460	-	V	ABC transporter, ATP-binding/permease protein
	SAK0561	-	V	ABC transporter, ATP-binding/permease protein
	GBS0507	-	V	hypothetical protein
C0447	SAG0461	-	V	ABC transporter, ATP-binding/permease protein
	SAK0562	-	V	ABC transporter, ATP-binding/permease protein
	GBS0508	-	V	hypothetical protein
C0448	SAG0462	*trp*G	EH	anthranilate synthase component II
	SAK0563	-	EH	glutamine amidotransferase
	GBS0509	-	EH	hypothetical protein

(Continue on next page)

List of homolog clusters in the 3 GBS reference genomes (Cont'd)

ID	Locus tag	Gene	COG	Annotation
C0449	SAG0463	-	R	BioY family protein
	SAK0565	-	R	BioY family protein
	GBS0510	-	R	hypothetical protein
C0450	SAG0464	*bio*B	H	biotin synthetase
	SAK0566	*bio*B	H	biotin synthetase
	GBS0511	-	H	hypothetical protein
C0451	SAG0465	-		hypothetical protein
	SAK0567	-		hypothetical protein
	GBS0512	-		hypothetical protein
C0452	SAG0466	-	I	thiolase
	SAK0568	-	I	acetyl-CoA acetyltransferase family protein
C0453	SAG0467	-	IQ	AMP-binding enzyme domain protein
	SAK0569	-	IQ	hypothetical protein
	GBS0514	-	IQ	hypothetical protein
C0454	SAG0468	*nth*	L	endonuclease III
	SAK0570	*nth*	L	endonuclease III
	GBS0515	-	L	hypothetical protein
C0455	SAG0469	-		type IV prepilin peptidase-related protein
	SAK0571	-		peptidase, A24A (type 4 prepilin peptidase 1) subfamily
	GBS0516	-		hypothetical protein
C0456	SAG0470	-		hypothetical protein
	SAK0572	-		hypothetical protein
	GBS0517	-		hypothetical protein
C0457	SAG0471	*glk*	KG	glucokinase
	SAK0573	-	KG	glucokinase, putative
	GBS0518	-	KG	hypothetical protein
C0458	SAG0472	-	P	rhodanese-like domain protein
	SAK0574	-	P	rhodanese-like domain protein
	GBS0519	-	P	hypothetical protein
C0459	SAG0473	-	T	elongation factor Tu family protein
	SAK0575	*typ*A	T	GTP-binding protein TypA
	GBS0520	-	T	hypothetical protein
C0460	SAG0474	-		hypothetical protein
	SAK0576	-		hypothetical protein
	GBS0521	-		hypothetical protein
C0461	SAG0475	*mur*D	M	UDP-N-acetylmuramoyl-L-alanyl-D-glutamate synthetase
	SAK0577	*mur*D	M	UDP-N-acetylmuramoyl-L-alanyl-D-glutamate synthetase
	GBS0522	*mur*D	M	UDP-N-acetylmuramoyl-L-alanyl-D-glutamate synthetase
C0462	SAG0476	*mur*G	M	N-acetylglucosaminyl transferase
	SAK0578	*mur*G	M	N-acetylglucosaminyl transferase
	GBS0523	*mur*G	M	N-acetylglucosaminyl transferase
C0463	SAG0477	-	M	cell division protein DivIB, putative
	SAK0579	*div*IB	M	cell division protein DivIB
	GBS0524	-	M	hypothetical protein
C0464	SAG0478	*fts*A	D	cell division protein FtsA
	SAK0580	*fts*A	D	cell division protein FtsA
	GBS0525	*fts*A	D	similar to cell division protein FtsA
C0465	SAG0479	*fts*Z	D	cell division protein FtsZ
	SAK0581	*fts*Z	D	cell division protein FtsZ
	GBS0526	FtsZ	D	cell division protein FtsZ
C0466	SAG0480	-	R	ylmE protein, putative
	SAK0582	-	R	conserved hypothetical protein TIGR00044
	GBS0527	-	R	hypothetical protein
C0467	SAG0481	*ylm*F	S	ylmF protein
	SAK0583	-	S	hypothetical protein
	GBS0528	-	S	hypothetical protein
C0468	SAG0482	-		YGGT family protein
	SAK0584	-		YggT family protein
	GBS0529	-		hypothetical protein
C0469	SAG0483	*ylm*H	S	ylmH protein
	SAK0585	-	S	S4 domain protein
	GBS0530	-	S	hypothetical protein
C0470	SAG0484	-	D	cell division protein DivIVA, putative
	SAK0586	-	D	cell division protein DivIVA, putative
	GBS0531	-	D	hypothetical protein
C0471	SAG0485	*ile*S	J	isoleucyl-tRNA synthetase
	SAK0587	*ile*S	J	isoleucyl-tRNA synthetase
	GBS0532	-	J	isoleucyl-tRNA synthetase
C0472	SAG0486	-		hypothetical protein
	SAK0588	-		hypothetical protein
	GBS0533	-		hypothetical protein

(Continue on next page)

List of homolog clusters in the 3 GBS reference genomes (Cont'd)

ID	Locus tag	Gene	COG	Annotation
C0473	SAG0487	-	LR	MutT/nudix family protein
	SAK0589	-	LR	hydrolase, NUDIX family
	GBS0534	-	LR	hypothetical protein
C0474	SAG0488	-	O	ATP-dependent Clp protease, ATP-binding subunit
	SAK0590	*clp*E	O	ATP-dependent Clp protease, ATP-binding subunit ClpE
	GBS0535	-	O	hypothetical protein
C0475	SAG0489	-		hypothetical protein
C0476	SAG0490	-		hypothetical protein
	SAK0591	-		hypothetical protein
	GBS0536	-		hypothetical protein
C0477	SAG0491	-	E	amino acid ABC transporter, permease protein
	SAK0592	-	E	polar amino acid uptake (PAAT) family ABC transporter, permease protein
	GBS0537	-	E	hypothetical protein
C0478	SAG0492	-	E	amino acid ABC transporter, ATP-binding protein
	SAK0593	-	E	polar amino acid uptake (PAAT) family ABC transporter, ATP-binding protein
	GBS0538	-	E	hypothetical protein
C0479	SAG0493	-	G	phosphoglucomutase/phosphomannomutase family protein
	SAK0594	-	G	phosphoglucomutase/phosphomannomutase family protein
	GBS0539	-	G	hypothetical protein
C0480	SAG0494	*fol*D	H	methylenetetrahydrofolate dehydrogenase/methenyltetrahydrofolate cyclohydrolase
	SAK0595	*fol*D	H	methylenetetrahydrofolate dehydrogenase/methenyltetrahydrofolate cyclohydrolase
	GBS0540	-	H	hypothetical protein
C0481	SAG0495	-	G	hypothetical protein
	SAK0596	-	G	carbohydrate kinase
	GBS0541	-	G	hypothetical protein
C0482	SAG0496	*xse*A	L	exodeoxyribonuclease VII large subunit
	SAK0597	*xse*A	L	exodeoxyribonuclease VII large subunit
	GBS0542	-	L	exodeoxyribonuclease VII large subunit
C0483	SAG0497	*xse*B	L	exodeoxyribonuclease VII small subunit
	SAK0598	*xse*B	L	exodeoxyribonuclease VII small subunit
	GBS0543	*xse*B	L	exodeoxyribonuclease VII small subunit
C0484	SAG0498	-	H	geranyltranstransferase, putative
	SAK0599	-	H	geranyltranstransferase
	GBS0544	-	H	hypothetical protein
C0485	SAG0499	-	J	hemolysin A
	SAK0600	*tly*A	J	hemolysin A
	GBS0545	-	J	hypothetical protein
C0486	SAG0500	-	K	arginine repressor ArgR, putative
	SAK0601	-	K	arginine repressor, putative
	GBS0546	-	K	hypothetical protein
C0487	SAG0501	*rec*N	L	DNA repair protein RecN
	SAK0602	*rec*N	L	DNA repair protein RecN
	GBS0547	-	L	hypothetical protein
C0488	SAG0502	-	S	DegV family protein
	SAK0603	-	S	DegV family protein
	GBS0548	-	S	hypothetical protein
C0489	SAG0503	-	E	lipase/acylhydrolase
	SAK0604	-	E	lipase/acylhydrolase, GDSL family
	GBS0549	-	E	hypothetical protein
C0490	SAG0504	-		hypothetical protein
	SAK0605	-		hypothetical protein
	GBS0550	-		hypothetical protein
C0491	SAG0505	*hup*		DNA-binding protein HU
	SAK0606	*hup*		DNA-binding protein HU
	GBS0551	-		HU like DNA-binding protein
C0492	SAG0506	-		hypothetical protein
	SAK0656	-		hypothetical protein
	GBS0552	-		hypothetical protein
C0493	SAG0507	*pyr*DA	F	dihydroorotate dehydrogenase
	SAK0657	*pyr*D	F	dihydroorotate dehydrogenase
	GBS0553	-	F	dihydroorotate dehydrogenase
C0494	SAG0508	*fib*B	V	beta-lactam resistance factor
	SAK0658	*fib*B	V	beta-lactam resistance factor
	GBS0554	-	V	hypothetical protein
C0495	SAG0509	*fib*A	V	beta-lactam resistance factor
	SAK0659	-	V	FemAB family protein
	GBS0555	-	V	hypothetical protein

(Continue on next page)

List of homolog clusters in the 3 GBS reference genomes (Cont'd)

ID	Locus tag	Gene	COG	Annotation
C0496	SAG0510	-	V	murM protein, putative
	SAK0660	-	V	FemAB family protein
	GBS0556	-	V	hypothetical protein
C0497	SAG0511	-	R	hydrolase, haloacid dehalogenase-like family
	SAK0661	-	R	Cof-like hydrolase
	GBS0557	-	R	hypothetical protein
C0498	SAG0512	-	R	HD domain protein
	SAK0662	-	R	HD domain protein
	GBS0558	-	R	hypothetical protein
C0499	SAG0513	-		hypothetical protein
	SAK0663	-		hypothetical protein
	GBS0559	-		hypothetical protein
C0500	SAG0514	-	P	cation-transporting ATPase, E1-E2 family
	SAK0664	-	P	calcium-transporting ATPase, P-type (transporting), HAD superfamily, subfamily IC
	GBS0560	-	P	hypothetical protein
C0501	SAG0515	-	R	hypothetical protein
	GBS0561	-	R	hypothetical protein
C0502	SAG0516	-		fructose-1,6-bisphosphatase, putative
	SAK0666	*fbp*		fructose-1,6-bisphosphatase
	GBS0562	-		hypothetical protein
C0503	SAG0517	-	C	iron-sulfur cluster-binding protein, putative
	SAK0667	-	C	iron-sulfur cluster-binding protein, putative
	GBS0563	-	C	hypothetical protein
C0504	SAG0519	*fts*E	D	cell division ABC transporter, ATP-binding protein FtsE
	SAK0669	*fts*E	D	cell division ATP binding protein FtsE
	GBS0565	-	D	hypothetical protein
C0505	SAG0520	*fts*X	D	cell division ABC transporter, permease protein FtsX
	SAK0670	-	D	cell division protein FtsX, putative
	GBS0566	-	D	hypothetical protein
C0506	SAG0521	-	R	carboxymethylenebutenolidase-related protein
	SAK0672	-	R	hypothetical protein
	GBS0567	-	R	hypothetical protein
C0507	SAG0522	-	R	metallo-beta-lactamase superfamily protein
	SAK0673	-	R	metallo-beta-lactamase family protein
	GBS0568	-	R	hypothetical protein
C0508	SAG0523	-	IQR	acetoin reductase
	SAK0674	-	IQR	acetoin reductase
	GBS0569	-	IQR	acetoin reductase
C0509	SAG0524	-	L	ATP-dependent DNA helicase
	SAK0675	-	L	ATP-dependent DNA helicase
	GBS0570	-	L	ATP-dependent DNA helicase
C0510	SAG0525	*asp*C	E	aspartate aminotransferase
	SAK0676	-	E	aspartate aminotransferase
	GBS0571	-	E	aspartate aminotransferase
C0511	SAG0526	*asn*S	J	asparaginyl-tRNA synthetase
	SAK0677	*asn*S	J	asparaginyl-tRNA synthetase
	GBS0572	-	J	asparaginyl-tRNA synthetase
C0512	SAG0527	-		hypothetical protein
	SAK0678	-		hypothetical protein
	GBS0573	-		hypothetical protein
C0513	SAG0528	-	F	inosine-uridine preferring nucleoside hydrolase
	SAK0679	-	F	inosine-uridine preferring nucleoside hydrolase family protein
	GBS0574	-	F	hypothetical protein
C0514	SAG0529	-		hypothetical protein
C0515	SAG0530	-	O	OsmC/Ohr family protein
	SAK0680	-	O	organic hydroperoxide resistance protein, putative
	GBS0575	-	O	hypothetical protein
C0516	SAG0531	-	R	hypothetical protein
	SAK0681	-	R	hypothetical protein
	GBS0576	-	R	hypothetical protein
C0517	SAG0532	-		hypothetical protein
	SAK0682	-		hypothetical protein
	GBS0577	-		hypothetical protein
C0518	SAG0533	-	S	hypothetical protein
	SAK0683	-	S	hypothetical protein
	GBS0578	-	S	hypothetical protein
C0519	SAG0534	-		dipeptidase
	SAK0684	*pep*DA		dipeptidase A
	GBS0579	-		hypothetical protein

(Continue on next page)

List of homolog clusters in the 3 GBS reference genomes (Cont'd)

ID	Locus tag	Gene	COG	Annotation
C0520	SAG0535	-	R	zinc ABC transporter, zinc-binding adhesion liprotein
	SAK0685	*adc*A	R	zinc ABC transporter, zinc-binding protein AdcA
	GBS0580	-	P	hypothetical protein
C0521	SAG0536	*rpm*E	J	50S ribosomal protein L31
	SAK0686	*rpm*E	J	50S ribosomal protein L31
	GBS0581	-	J	50S ribosomal protein L31
C0522	SAG0537	-	R	DHH family protein
	SAK0687	-	R	DHH family protein
	GBS0582	-	R	hypothetical protein
C0523	SAG0538	-	F	adenosine deaminase
	SAK0688	*add*	F	adenosine deaminase
	GBS0583	-	F	adenosine deaminase
C0524	SAG0539	-	C	flavodoxin
	SAK0689	-	C	flavodoxin
	GBS0584	-	C	flavodoxin
C0525	SAG0540	-		chorismate mutase, putative
	SAK0690	-		chorismate mutase
	GBS0585	-		hypothetical protein
C0526	SAG0541	-	P	voltage-gated chloride channel family protein
	SAK0691	-	P	voltage-gated chloride channel family protein
	GBS0586	-	P	hypothetical protein
C0527	SAG0544	*rpl*S		50S ribosomal protein L19
	SAK0692	*rpl*S		50S ribosomal protein L19
	GBS0587	-		50S ribosomal protein L19
C0528	SAG0217	-	L	site-specific recombinase, phage integrase family
	SAG0545	-		prophage LambdaSa1, site-specific recombinase, phage integrase family
	SAG2112	-		site-specific recombinase, phage integrase family
	SAK0278	-	L	site-specific recombinase, phage integrase family
	SAK0607	-		prophage LambdaSa03, site-specific recombinase, phage integrase family
	GBS0211	-	L	hypothetical protein
	GBS0237	-	L	hypothetical protein
C0529	SAG0546	-		hypothetical protein
C0530	SAG0547	-	S	hypothetical protein
C0531	SAG0548	-	K	prophage LambdaSa1, repressor protein, putative
	SAG1268	-	K	repressor protein, putative
	GBS1221	-	K	hypothetical protein
C0532	SAG0549	-		hypothetical protein
C0533	SAG0550	-		hypothetical protein
C0534	SAG0551	-		hypothetical protein
C0535	SAG0552	-		hypothetical protein
C0536	SAG0553	-		hypothetical protein
C0537	SAG0554	-	K	prophage LambdaSa1, transcriptional regulator, Cro/CI family
C0538	SAG0555	-	K	prophage LambdaSa1, antirepressor, putative
C0539	SAG0556	-		hypothetical protein
C0540	SAG0557	-		hypothetical protein
C0541	SAG0558	-		hypothetical protein
C0542	SAG0559	-	L	hypothetical protein
C0543	SAG0560	-		hypothetical protein
C0544	SAG0561	-		hypothetical protein
C0545	SAG0562	-		hypothetical protein
C0546	SAG0563	-		hypothetical protein
C0547	SAG0564	-		hypothetical protein
C0548	SAG0565	-		hypothetical protein
C0549	SAG0566	*ssb*	L	prophage LambdaSa1, single-strand binding protein
	SAG1713	*ssb*	L	single-strand DNA-binding protein
	SAG1863	*ssb*	L	prophage LambdaSa2, single-strand binding protein
	SAK1721	*ssb*1	L	single-strand DNA-binding protein
	GBS1758	-	L	single-strand DNA-binding protein
C0550	SAG0567	-	L	prophage LambdaSa1, reverse transcriptase/maturase family protein
C0551	SAG0568	-		hypothetical protein
C0552	SAG0569	-	L	hypothetical protein
C0553	SAG0570	-		hypothetical protein
C0554	SAG0571	-		hypothetical protein
C0555	SAG0572	-		hypothetical protein
C0556	SAG0573	-		hypothetical protein
	SAK0623	-		hypothetical protein
C0557	SAG0574	-		hypothetical protein
C0558	SAG0575	-		hypothetical protein
C0559	SAG0576	-		hypothetical protein

(Continue on next page)

List of homolog clusters in the 3 GBS reference genomes (Cont'd)

ID	Locus tag	Gene	COG	Annotation
C0560	SAG0577	-		hypothetical protein
	SAK0625	-		hypothetical protein
C0561	SAG0578	-		hypothetical protein
	SAK0628	-		hypothetical protein
C0562	SAG0579	-		hypothetical protein
	SAK0629	-		hypothetical protein
C0563	SAG0581	-		hypothetical protein
	SAK0631	-		hypothetical protein
C0564	SAG0582	-		hypothetical protein
	SAK0632	-		hypothetical protein
C0565	SAG0583	-		hypothetical protein
	SAK0633	-		hypothetical protein
C0566	SAG0585	-		hypothetical protein
	SAK0635	-		prophage LambdaSa03, terminase, large subunit, putative
C0567	SAG0586	-		hypothetical protein
	SAK0636	-		hypothetical protein
C0568	SAG0587	-		prophage LambdaSa1, structural protein, putative
	SAK0637	-		prophage LambdaSa03, structural protein, putative
C0569	SAG0588	-		hypothetical protein
	SAK0638	-		hypothetical protein
C0570	SAG0589	-		hypothetical protein
	SAK0639	-		hypothetical protein
C0571	SAG0590	-		hypothetical protein
	SAK0640	-		hypothetical protein
C0572	SAG0591	-		hypothetical protein
C0573	SAG0592	-		hypothetical protein
	SAK0641	-		hypothetical protein
C0574	SAG0593	-		prophage LambdaSa1, structural protein
	SAK0642	-		prophage LambdaSa03, structural protein, putative
C0575	SAG0594	-		hypothetical protein
	SAK0643	-		hypothetical protein
C0576	SAG0595	-		hypothetical protein
	SAK0644	-		hypothetical protein
C0577	SAG0596	-	S	prophage LambdaSa1, pblA protein, internal deletion
	SAK0645	-	S	prophage LambdaSa03, pblA protein, internal deletion
C0578	SAG0597	-		prophage LambdaSa1, minor structural protein, putative
	SAK0646	-		prophage LambdaSa03, tail component, putative
C0579	SAG0598	-	NU	prophage LambdaSa1, N-acetylmuramoyl-L-alanine amidase, family 4
	SAG1837	-		prophage LambdaSa2, lysin, putative
	SAK0647	-	NU	prophage LambdaSa03, minor structural protein, putative
C0580	SAG0599	-		prophage LambdaSa1, minor structural protein, putative
	SAK0648	-		prophage LambdaSa03, minor structural protein, putative
	SAK0759	-		prophage LambdaSa04, minor structural protein, putative
C0581	SAG0600	-		hypothetical protein
	SAK0649	-		hypothetical protein
C0582	SAG0601	-		hypothetical protein
C0583	SAG0602	-		hypothetical protein
C0584	SAG0603	-		hypothetical protein
C0585	SAG0604	-		prophage LambdaSa1, lysin, putative
	SAK0653	-	M	prophage LambdaSa03, peptidoglycan endolysin
C0586	SAG0605	-	V	hypothetical protein
C0587	SAG0606	-		hypothetical protein
	SAG0619	-		hypothetical protein
	SAK0704	-		CsbD family protein
	GBS0600	-		hypothetical protein
C0588	SAG0607	-		hypothetical protein
	SAK0654	-		hypothetical protein
C0589	SAG0608	-		hypothetical protein
	SAK0655	-		hypothetical protein
C0590	SAG0610	-	L	hypothetical protein
	SAK0695	-	L	ISSag6, transposase orfA
	GBS0589	-	L	hypothetical protein
C0591	SAG0612	-		hypothetical protein
	SAK0608	-	S	hypothetical protein
	SAK0697	-		hypothetical protein
	GBS0593	-		unknon
C0592	SAG0613	*vex*1		transmembrane protein Vexp1
	SAK0698	-		ABC transporter, permease protein
	GBS0594	-		hypothetical protein

(Continue on next page)

List of homolog clusters in the 3 GBS reference genomes (Cont'd)

ID	Locus tag	Gene	COG	Annotation
C0593	SAG0614	*vex*2	V	ABC transporter, ATP-binding protein Vexp2
	SAK0699	-	V	ABC transporter, ATP-binding protein
	GBS0595	-	V	hypothetical protein
C0594	GBS0596	-	V	hypothetical protein
C0595	SAG0616	*vnc*R	TK	DNA-binding response regulator VncR
	SAK0189	-	TK	DNA-binding response regulator
	SAK0701	*vnc*R	TK	DNA-binding response regulator VncR
	GBS0597	-	TK	hypothetical protein
C0596	SAG0617	*vnc*S	T	sensor histidine kinase VncS
	SAK0188	-	T	sensor histidine kinase
	SAK0702	-	T	sensor histidine kinase VncS, putative
	GBS0598	-	T	hypothetical protein
C0597	SAG0620	-		hypothetical protein
	SAK0705	-		hypothetical protein
C0598	SAG0621	*rod*A		rod shape-determining protein RodA, putative
	SAK0706	-		cell division protein, FtsW/RodA/SpoVE family
	GBS0601	-		hypothetical protein
C0599	SAG0622	-	R	hydrolase, haloacid dehalogenase-like family
	SAK0707	-	R	HAD-superfamily hydrolase, subfamily IA, variant 1 family protein
	GBS0602	-	R	hypothetical protein
C0600	SAG0623	*gyr*B	L	DNA gyrase subunit B
	SAK0708	*gyr*B	L	DNA gyrase subunit B
	GBS0603	*gyr*B	L	DNA gyrase subunit B
C0601	SAG0624	-	D	septation ring formation regulator EzrA
	SAK0709	*ezr*A	D	septation ring formation regulator EzrA
	GBS0604	-	D	septation ring formation regulator EzrA
C0602	SAG0625	*ser*B	E	phosphoserine phosphatase SerB
	SAK0710	*ser*B	E	phosphoserine phosphatase SerB
	GBS0605	-	E	hypothetical protein
C0603	SAG0626	-	LR	MutT/nudix family protein
	SAK0711	-	LR	hydrolase, NUDIX family
	GBS0606	-	LR	hypothetical protein
C0604	SAG0627	-	S	hypothetical protein
	SAK0712	-	S	hypothetical protein
	GBS0607	-	S	hypothetical protein
C0605	SAG0628	*eno*	G	phosphopyruvate hydratase
	SAK0713	*eno*	G	phosphopyruvate hydratase
	GBS0608	*eno*	G	phosphopyruvate hydratase
C0606	SAG0629	-		hypothetical protein
	SAK0714	-		hypothetical protein
	GBS0153	-		hypothetical protein
	GBS0609	-		hypothetical protein
C0607	SAG0630	*aro*A	E	3-phosphoshikimate 1-carboxyvinyltransferase
	SAK0715	*aro*A	E	3-phosphoshikimate 1-carboxyvinyltransferase
	GBS0610	*aro*A	E	3-phosphoshikimate 1-carboxyvinyltransferase
C0608	SAG0631	*aro*K	E	shikimate kinase
	SAK0716	*aro*K	E	shikimate kinase
	GBS0611	*aro*K	E	shikimate kinase
C0609	SAG0632	-	K	psr protein
	SAK0717	-	K	transcriptional regulator, putative
	GBS0612	-	K	hypothetical protein
C0610	SAG0633	-	J	RNA methyltransferase, TrmA family
	SAK0718	*rum*A	J	23S rRNA (uracil-5-)-methyltransferase RumA
	GBS0613	-	J	hypothetical protein
C0611	SAG0634	-		hypothetical protein
	SAK0767	-		hypothetical protein
	GBS0614	-		hypothetical protein
C0612	SAG0635	-	R	acid phosphatase, class B
	SAK0768	-	R	HAD-superfamily phosphatase, subfamily IIIB
	GBS0615	-	R	hypothetical protein
C0613	SAG0636	-	S	hypothetical protein
	SAG2111	-	S	hypothetical protein
	SAK0719	-	S	hypothetical protein
	SAK0769	-	S	hypothetical protein
	GBS0616	-	S	hypothetical protein

(Continue on next page)

List of homolog clusters in the 3 GBS reference genomes (Cont'd)

ID	Locus tag	Gene	COG	Annotation
C0614	SAG0639	-	L	transposase OrfB, IS3 family
	SAG1068	-	L	IS861, transposase OrfB
	SAG1229	-	L	ISSdy1, transposase OrfB
	SAG1244	-	L	ISSdy1, transposase OrfB
	SAG1527	-	L	IS861, transposase OrfB
	SAK0505	-	L	IS861, transposase orfB
	SAK0703	-	L	transposase OrfB, IS3 family, truncation
	SAK0772	-	L	ISSag5, transposase orfB
	SAK1315	-	L	ISSag4, transposase orfB
	SAK1323	-		ISSag4, transposase orfB
	SAK1550	-	L	IS861, transposase orfB
	GBS1301	-	L	hypothetical protein
C0615	SAG0640	-	L	transposase OrfA, IS3 family
	SAG1241	-	L	transposase OrfA, IS3 family
	SAK0773	-	L	ISSag5, transposase orfA
	GBS0621	-	L	hypothetical protein
C0616	SAG0642	-		hypothetical protein
C0617	SAG0644	-		transcriptional regulator, AraC family
	SAK0775	-		transcriptional regulator, AraC family
	GBS0627	-		hypothetical protein
C0618	SAG0645	-	M	cell wall surface anchor family protein
	SAG1407	-	M	cell wall surface anchor family protein
	SAK0776	-	M	cell wall surface anchor family protein
	GBS0628	-	M	hypothetical protein
	GBS1477	-		hypothetical protein
C0619	SAG0646	-	M	cell wall surface anchor family protein
	SAG1404	-	M	cell wall surface anchor family protein
	SAK0777	-	M	cell wall surface anchor family protein
	GBS0629	-	M	hypothetical protein
	GBS1474	-	M	hypothetical protein
C0620	SAG0647	-	M	sortase family protein
	SAG0650	-	M	sortase family protein
	SAG1406	-	M	sortase family protein
	SAK0778	-	M	sortase family protein
	GBS0630	-	M	hypothetical protein
	GBS1476	-	M	hypothetical protein
C0621	SAG0648	-	M	sortase family protein
	SAG1405	-	M	sortase family protein
	SAK0779	-	M	sortase family protein
	SAK1439	-	M	sortase family protein
	GBS0631	-	M	hypothetical protein
	GBS1475	-	M	hypothetical protein
C0622	SAG0649	-	M	cell wall surface anchor family protein, putative
	SAG1408	-	M	cell wall surface anchor family protein
	SAK0780	-	M	cna B-type domain protein
	GBS0632	-	M	hypothetical protein
	GBS1478	-	M	hypothetical protein
C0623	SAG0651	-	M	hypothetical protein
	SAK0782	-	M	Cna protein B-type domain
	GBS0636	-	M	hypothetical protein
C0624	SAG0654	-		hypothetical protein
C0625	SAG0655	-		hypothetical protein
C0626	SAG0656	-		hypothetical protein
C0627	SAG0657	-		hypothetical protein
	SAK0785	-		hypothetical protein
	GBS0639	-		hypothetical protein
C0628	SAG0658	-	V	lipoprotein, putative
	SAK0786	-	V	beta-lactamase, putative
	GBS0640	-	V	hypothetical protein
C0629	SAG0659	-	R	ABC transporter, ATP-binding protein
	SAK0787	-	R	ABC transporter, ATP-binding protein
	GBS0641	-	R	hypothetical protein
C0630	SAG0660	-	R	hypothetical protein
	SAK0788	-	R	hypothetical protein
	GBS0642	-	R	hypothetical protein
C0631	SAG0661	-	R	hypothetical protein
	SAK0789	-	R	hypothetical protein
	GBS0643	-	R	hypothetical protein
C0632	SAG0662	-		cylX protein
	SAK0790	*cyl*X		cylX protein
	GBS0644	*cyl*X		hypothetical protein

(Continue on next page)

List of homolog clusters in the 3 GBS reference genomes (Cont'd)

ID	Locus tag	Gene	COG	Annotation
C0633	SAG0663	*cyl*D	I	cylD protein
	SAK0791	*cyl*D	I	CylD protein
	GBS0645	*cyl*D	I	hypothetical protein
C0634	SAG0664	*cyl*G	IQR	cylG protein
	SAK0792	*cyl*G	IQR	cylG protein
	GBS0646	*cyl*G	IQR	hypothetical protein
C0635	SAG0665	-	IQ	acyl carrier protein AcpC
	SAK0793	-	IQ	acyl carrier protein
	GBS0647	*acp*C	IQ	hypothetical protein
C0636	SAG0666	-	I	cylZ protein
	SAK0794	*cyl*Z	I	cylZ protein
	GBS0648	-	I	hypothetical protein
C0637	SAG0667	*cyl*A	V	cylA protein
	SAK0795	*cyl*A	V	ABC transporter, ATP-binding protein CylA
	GBS0649	*cyl*A	V	hypothetical protein
C0638	SAG0668	*cyl*B		cylB protein
	SAK0796	*cyl*B		ABC transporter, permease protein CylB
	GBS0650	*cyl*B		hypothetical protein
C0639	SAG0669	*cyl*E		cylE protein
	SAK0797	*cyl*E		CylE protein
	GBS0651	*cyl*E		hypothetical protein
C0640	SAG0670	*cyl*F	E	cylF protein
	SAK0798	*cyl*F	E	cylF protein
	GBS0652	*cyl*F	E	hypothetical protein
C0641	SAG0671	*cyl*I	IQ	cylI protein
	SAK0799	*cyl*I	IQ	cylI protein
	GBS0653	*cyl*I	IQ	hypothetical protein
C0642	SAG0672	*cyl*J	GC	cylJ protein
	SAK0800	*cyl*J	GC	CylJ protein
	GBS0654	*cyl*J	GC	hypothetical protein
C0643	SAG0673	*cyl*K		cylK protein
	SAK0801	*cyl*K		CylK protein
	GBS0655	*cyl*K		hypothetical protein
C0644	SAG0674	-		hypothetical protein
	SAK0802	-		hypothetical protein
C0645	SAG0675	-		secreted protein, putative
	SAK0803	-		hypothetical protein
C0646	SAG0676	-	O	proteinase, putative
	SAG2053	-	O	serine protease, subtilase family
	SAK0804	-	O	peptidase, S8 (subtilisin) family
	SAK1991	*csp*A	O	cell surface serine endopeptidase CspA
	GBS2008	-	O	hypothetical protein
C0647	SAG0677	-		hypothetical protein
	SAK0805	-		hypothetical protein
C0648	SAG0679	-	R	hypothetical protein
	SAK0807	-	R	hypothetical protein
C0649	SAG0680	-	R	hypothetical protein
	SAK0808	-	R	hypothetical protein
C0650	SAG0681	-	R	hypothetical protein
	SAK0809	-	R	hypothetical protein
C0651	SAG0615	*vex*3	V	transmembrane protein Vexp3
	SAG0682	-	V	permease, putative
	SAK0700	-	V	ABC transporter, permease protein Vexp3
	SAK0810	-	V	permease, putative
	GBS0657	-	V	hypothetical protein
C0652	SAG0684	-	V	ABC transporter, ATP-binding protein
	SAK0812	-	V	ABC transporter, ATP-binding protein
	GBS0659	-	V	hypothetical protein
C0653	SAG0685	-	S	hypothetical protein
	SAK0813	-	S	hypothetical protein
	GBS0660	-	S	hypothetical protein
C0654	SAG0686	-		DNA-entry nuclease, putative
	SAK0220	-		hypothetical protein
	SAK0814	-		DNA-entry nuclease, putative
	GBS0382	-		hypothetical protein
	GBS0661	-		hypothetical protein
	GBS0712	-		hypothetical protein
	GBS0997	-		hypothetical protein
C0655	SAG0687	-	S	DedA family protein, putative
	SAK0815	-	S	DedA family protein
	GBS0662	-	S	hypothetical protein

(Continue on next page)

List of homolog clusters in the 3 GBS reference genomes (Cont'd)

ID	Locus tag	Gene	COG	Annotation
C0656	SAG0688	-	R	ABC transporter, ATP-binding protein
	SAK0816	-	R	ABC transporter, ATP-binding protein
	GBS0663	-	R	hypothetical protein
C0657	SAG0689	-	R	hypothetical protein
	SAK0817	-	R	hypothetical protein
	GBS0664	-	R	hypothetical protein
C0658	SAG0690	-		hypothetical protein
	SAK0818	-		hypothetical protein
	GBS0665	-		hypothetical protein
C0659	SAG0691	-	K	transcriptional regulator, LysR family
	SAK0819	-	K	transcriptional regulator, LysR family
	GBS0666	-	K	hypothetical protein
C0660	SAG0692	-	R	regulatory protein, putative
	SAK0820	-	R	hypothetical protein
	GBS0667	-	R	hypothetical protein
C0661	SAG0693	-	L	IS1548 transposase
C0662	SAG0695	*ldh*A	CHR	2-hydroxyacid dehydrogenase
	SAK0821	*ldh*A	CHR	2-hydroxyacid dehydrogenase
	GBS0668	-	CHR	2-hydroxyacid dehydrogenase
C0663	SAG0696	-	G	sugar transporter, putative
	SAK0822	-	G	sugar transporter, putative
	GBS0669	-	G	hypothetical protein
C0664	SAG0697	-	G	2-keto-3-deoxygluconate kinase
	SAG1906	-	G	carbohydrate kinase, PfkB family
	SAK0823	-	G	kinase, PfkB family
	GBS0670	-	G	hypothetical protein
	GBS1893	-	G	hypothetical protein
C0665	SAG0698	-	G	beta-glucuronidase
	SAK0824	-	G	beta-glucuronidase
	GBS0671	-	G	hypothetical protein
C0666	SAG0699	-	K	transcriptional regulator, GntR family
	SAK0825	-	K	transcriptional regulator, GntR family
	GBS0672	-	K	hypothetical protein
C0667	SAG0700	*eda*	G	2-dehydro-3-deoxyphosphogluconate aldolase/4-hydroxy-2-oxoglutarate aldolase
	SAG1907	*eda*	G	keto-hydroxyglutarate-aldolase/keto-deoxy-phosphogluconate aldolase
	SAK0826	*eda*	G	2-dehydro-3-deoxyphosphogluconate aldolase/4-hydroxy-2-oxoglutarate aldolase
	GBS0673	-	G	hypothetical protein
	GBS1894	-	G	keto-hydroxyglutarate-aldolase/keto-deoxy-phosphogluconate aldolase
C0668	SAG0701	*uxa*C	G	uronate isomerase
	SAK0827	*uxa*C	G	uronate isomerase
	GBS0674	-	G	uronate isomerase
C0669	SAG0702	*uxu*A	G	mannonate dehydratase
	SAK0828	*uxu*A	G	mannonate dehydratase
	GBS0675	-	G	mannonate dehydratase
C0670	SAG0703	-	IQR	D-mannonate oxidoreductase
	SAK0829	-	IQR	D-mannonate oxidoreductase
	GBS0676	-	IQR	D-mannonate oxidoreductase
C0671	SAG0704	-	R	hydrolase, haloacid dehalogenase-like family
	SAK0830	-	R	hypothetical protein
	GBS0677	-	R	hypothetical protein
C0672	SAG0705	-		glycosyl hydrolase, family 3
	SAK0831	-		glycosyl hydrolase, family 3
	GBS0678	-		hypothetical protein
C0673	SAG0706	*pep*Q	E	proline dipeptidase
	SAK0832	*pep*Q	E	Xaa-Pro dipeptidase
	GBS0679	-	E	hypothetical protein
	GBS1751	-	E	hypothetical protein
C0674	SAG0707	-	K	transcriptional regulator, RegM family
	SAK0833	*ccp*A	K	catabolite control protein A
	GBS0680	*ccp*A	K	catabolite control protein A
C0675	SAG0708	-		cytoplasmic alpha-amylase
	SAK0834	-		cytoplasmic alpha-amylase
	GBS0681	-		cytoplasmic alpha-amylase
C0676	SAG0709	-	M	glycosyl transferase, group 1 family protein
	SAK0835	-	M	glycosyl transferase, group 1 family protein
	GBS0682	-	M	hypothetical protein
C0677	SAG0710	-	M	glycosyl transferase, group 1 family protein
	SAK0836	-	M	glycosyl transferase, group 1 family protein
	GBS0683	-	M	hypothetical protein

(Continue on next page)

List of homolog clusters in the 3 GBS reference genomes (Cont'd)

ID	Locus tag	Gene	COG	Annotation
C0678	SAG0711	*thr*S	J	threonyl-tRNA synthetase
	SAK0837	*thr*S	J	threonyl-tRNA synthetase
	GBS0684	*thr*S	J	threonyl-tRNA synthetase
C0679	SAG0712	-	TK	DNA-binding response regulator
	SAK0838	-	TK	DNA-binding response regulator
	GBS0685	-	TK	hypothetical protein
C0680	SAG0713	-		hypothetical protein
	SAK0839	-		hypothetical protein
	GBS0686	-		hypothetical protein
C0681	SAG0714	-		hypothetical protein
	SAK0840	-		peptidase propeptide and YPEB domain protein
	GBS0687	-		hypothetical protein
C0682	SAG0715	-	E	amino acid ABC transporter, permease protein
	SAK0841	-	E	polar amino acid uptake (PAAT) family ABC transporter, permease protein
	GBS0688	-	E	hypothetical protein
C0683	SAG0716	-	E	amino acid ABC transporter, permease protein
	SAK0842	-	E	polar amino acid uptake (PAAT) family ABC transporter, permease protein
	GBS0689	-	E	hypothetical protein
C0684	SAG0717	-	ET	amino acid ABC transporter, amino acid-binding protein
	SAK0843	-	ET	polar amino acid uptake (PAAT) family ABC transporter, amino acid-binding protein
	GBS0690	-	ET	hypothetical protein
C0685	SAG0718	-	E	amino acid ABC transporter, ATP-binding protein
	SAK0844	-	E	polar amino acid uptake (PAAT) family ABC transporter, ATP-binding protein
	GBS0691	-	E	hypothetical protein
C0686	SAG0719	-	TK	DNA-binding response regulator
	SAK0845	-	TK	DNA-binding response regulator
	GBS0741	-	TK	hypothetical protein
C0687	SAG0720	-	T	sensory box histidine kinase
	SAK0846	-	T	sensory box histidine kinase
	GBS0742	-	T	hypothetical protein
C0688	SAG0721	-	R	metallo-beta-lactamase superfamily protein
	SAK0847	-	R	metallo-beta-lactamase family protein
	GBS0743	-	R	hypothetical protein
C0689	SAG0722	-	S	hypothetical protein
	SAK0848	-	S	hypothetical protein
	GBS0744	-	S	hypothetical protein
C0690	SAG0723	*rnc*S	K	ribonuclease III
	SAK0849	*rnc*S	K	ribonuclease III
	GBS0745	*rnc*S	K	ribonuclease III
C0691	SAG0724	-	D	chromosome segregation SMC protein
	SAK0850	*smc*	D	chromosome segregation protein SMC
	GBS0746	-	D	hypothetical protein
C0692	SAG0725	-	R	hydrolase, haloacid dehalogenase-like family
	SAK0851	-	R	Cof-like hydrolase family protein
	GBS0747	-	R	hypothetical protein
C0693	SAG0726	-	R	hydrolase, haloacid dehalogenase-like family
	SAK0852	-	R	Cof-like hydrolase
	GBS0748	-	R	hypothetical protein
C0694	SAG0727	*fts*Y	U	signal recognition particle-docking protein FtsY
	SAK0853	*fts*Y	U	signal recognition particle-docking protein FtsY
	GBS0749	-	U	hypothetical protein
C0695	SAG0728	-	S	ABC transporter, substrate-binding protein
	SAK0854	-	S	hypothetical protein
	GBS0750	-	S	hypothetical protein
C0696	SAG0729	-	R	ABC transporter, permease protein, putative
	SAK0855	-	R	permease, putative
	GBS0751	-	R	hypothetical protein
C0697	SAG0730	-		ABC transporter, ATP-binding protein
	SAK0856	-		hypothetical protein
	GBS0752	-		hypothetical protein
C0698	SAG0731	-	C	bacterial luciferase family protein
	SAK0857	-	C	bacterial luciferase family protein
	GBS0753	-	C	hypothetical protein
C0699	SAG0732	-	K	transcriptional accessory protein Tex, putative
	SAK0858	-	K	S1 RNA binding domain protein
	GBS0754	-	K	hypothetical protein
C0700	SAG0733	-		hypothetical protein
	SAK0859	-		hypothetical protein
	GBS0755	-		hypothetical protein

(Continue on next page)

List of homolog clusters in the 3 GBS reference genomes (Cont'd)

ID	Locus tag	Gene	COG	Annotation
C0701	SAG0734	-	KT	phage shock protein C, putative
	SAK0860	-	KT	PspC domain protein
	GBS0756	-	KT	hypothetical protein
C0702	SAG0735	-		hypothetical protein
	SAK0861	-		hypothetical protein
C0703	SAG0736	*hpr*K	T	HPr kinase/phosphorylase
	SAK0862	*hpr*K	T	HPr kinase/phosphorylase
	GBS0757	*pts*K	T	HPr kinase/phosphorylase
C0704	SAG0737	*lgt*		prolipoprotein diacylglyceryl transferase
	SAK0863	*lgt*		prolipoprotein diacylglyceryl transferase
	GBS0758	-		prolipoprotein diacylglyceryl transferase
C0705	SAG0738	-	R	hypothetical protein
	SAK0864	-	R	hypothetical protein
	GBS0759	-	R	hypothetical protein
C0706	SAG0739	-		hypothetical protein
	SAK0865	-		hypothetical protein
	GBS0760	-		hypothetical protein
C0707	SAG0740	-		hypothetical protein
	SAK0866	-		hypothetical protein
	GBS0761	-		hypothetical protein
C0708	SAG0741	-		hypothetical protein
	SAK0867	-		peptidase, U32 (collagenase) family
	GBS0762	-		hypothetical protein
C0709	SAG0742	-		peptidase, U32 family
	SAK0868	-		peptidase, U32 (collagenase) family
	GBS0763	-		hypothetical protein
C0710	SAG0743	-	S	hypothetical protein
	SAK0869	-	S	hypothetical protein
	GBS0764	-	S	hypothetical protein
C0711	SAG0744	-		hypothetical protein
	SAK0870	-		hypothetical protein
	GBS0765	-		hypothetical protein
C0712	SAG0745	-	P	Mn2+/Fe2+ transporter, NRAMP family
	SAG2025	-	P	Mn2+/Fe2+ transporter, NRAMP family
	SAK0871	-	P	Mn2+/Fe2+ transporter, NRAMP family
	GBS0766	-	P	hypothetical protein
C0713	SAG0746	*rib*D	H	riboflavin biosynthesis protein RibD
	SAK0872	*rib*D	H	riboflavin biosynthesis protein RibD
	GBS0767	-	H	hypothetical protein
C0714	SAG0747	*rib*E	H	riboflavin synthase subunit alpha
	SAK0873	*rib*E	H	riboflavin synthase subunit alpha
	GBS0768	-	H	riboflavin synthase subunit alpha
C0715	SAG0748	*rib*A	H	riboflavin biosynthesis protein RibA
	SAK0874	*rib*BA	H	3,4-dihydroxy-2-butanone-4-phosphate synthase/GTP cyclohydrolase II
	GBS0769	-	H	hypothetical protein
C0716	SAG0749	*rib*H		riboflavin synthase, beta subunit
	SAK0875	*rib*E		6,7-dimethyl-8-ribityllumazine synthase
	GBS0770	-		hypothetical protein
C0717	SAG0750	*lys*S	J	lysyl-tRNA synthetase
	SAK0876	*lys*S	J	lysyl-tRNA synthetase
	GBS0771	*lys*S	J	lysyl-tRNA synthetase
C0718	SAG0751	-	R	hydrolase, haloacid dehalogenase-like family
	SAK0877	-	R	HAD-superfamily hydrolase, subfamily IA, variant 1 family protein
	GBS0772	-	R	hypothetical protein
C0719	SAG0752	-	G	phosphoglycerate mutase family protein
	SAK0878	-	G	phosphoglycerate mutase family protein
	GBS0773	-	G	hypothetical protein
C0720	SAG0753	-	S	ebsC family protein, putative
	SAK0879	-	S	YbaK/EbsC family protein
	GBS0774	-	S	hypothetical protein
C0721	SAG0754	-	MG	hypothetical protein
	SAK0880	-	MG	hypothetical protein
	GBS0775	-	MG	hypothetical protein
C0722	SAG0755	-	M	peptidase, U32 family
	SAK0881	-	M	glycosyl hydrolase, family 25
	GBS0776	-	M	hypothetical protein
C0723	SAG0756	-		hypothetical protein
	SAK0882	-		hypothetical protein
	GBS0777	-		hypothetical protein

(Continue on next page)

List of homolog clusters in the 3 GBS reference genomes (Cont'd)

ID	Locus tag	Gene	COG	Annotation
C0724	SAG0757	-		protein of unknown function/lipoprotein, putative
	SAK0883	-		lipoprotein, putative
	GBS0778	-		hypothetical protein
C0725	SAG0758	-	E	oligoendopeptidase F, putative
	SAK0884	*pep*F	E	oligoendopeptidase F
	GBS0779	-	E	hypothetical protein
C0726	SAG0759	*ppc*	C	phosphoenolpyruvate carboxylase
	SAK0885	*ppc*	C	phosphoenolpyruvate carboxylase
	GBS0780	-	C	phosphoenolpyruvate carboxylase
C0727	SAG0760	-	L	IS1548 transposase
C0728	SAG0761	*fts*W		cell division protein, FtsW/RodA/SpoVE family
	SAK0886	-		cell division protein, FtsW/RodA/SpoVE family
	GBS0781	-		hypothetical protein
C0729	SAG0762	*tuf*	J	elongation factor Tu
	SAK0887	*tuf*	J	elongation factor Tu
	GBS0782	*tuf*	J	elongation factor Tu
C0730	SAG0763	*tpi*A	G	triosephosphate isomerase
	SAK0888	*tpi*A	G	triosephosphate isomerase
	GBS0783	*tpi*A	G	triosephosphate isomerase
C0731	SAG0764	-	G	phosphoglyceromutase
	SAK0889	*gpm*A	G	phosphoglyceromutase
	GBS0784	-	G	phosphoglyceromutase
C0732	SAG0765	-	M	penicillin-binding protein 2b
	SAK0890	-	M	penicillin-binding protein 2b
	GBS0785	-	M	hypothetical protein
C0733	SAG0766	*rec*R	L	recombination protein RecR
	SAK0891	*rec*R	L	recombination protein RecR
	GBS0786	*rec*R	L	recombination protein RecR
C0734	SAG0767	*ddl*	M	D-alanylalanine synthetase
	SAK0892	*ddl*	M	D-alanylalanine synthetase
	GBS0787	*ddl*	M	D-alanylalanine synthetase
C0735	SAG0768	*mur*F	M	UDP-N-acetylmuramoylalanyl-D-glutamyl-2,6-diaminopimelate–D-alanyl-D-alanyl ligase
	SAK0893	*mur*F	M	UDP-N-acetylmuramoyl-tripeptide–D-alanyl-D-alanine ligase
	GBS0788	-	M	hypothetical protein
C0736	SAG0769	-		oxalate:formate antiporter
	SAK0894	-		major facilitator family transporter
	GBS0789	-		hypothetical protein
C0737	SAG0770	-	S	hypothetical protein
	SAK0895	-	S	conserved hypothetical integral membrane protein TIGR02206
	GBS0790	-	S	hypothetical protein
C0738	SAG0771	-		cell wall surface anchor family protein
	SAK0896	-		cell wall surface anchor family protein
	GBS0791	-		hypothetical protein
C0739	SAG0772	*prf*C	J	peptide chain release factor 3
	SAK0897	*prf*C	J	peptide chain release factor 3
	GBS0792	-	J	peptide chain release factor 3
C0740	SAG0773	-		hypothetical protein
	SAK0898	-		hypothetical protein
	GBS0793	-		hypothetical protein
C0741	SAG0774	-	P	ABC transporter, ATP-binding protein
	SAK0899	-	P	amino acid ABC transporter, ATP-binding protein, putative
	GBS0794	-	P	hypothetical protein
C0742	SAG0775	-	P	ABC transporter, permease protein
	SAK0900	-	P	amino acid ABC transporter, permease protein, putative
	GBS0795	-	P	hypothetical protein
C0743	SAG0776	-	P	YaeC family protein
	SAK0901	-	P	ABC transporter, substrate-binding protein
	GBS0796	-	P	hypothetical protein
C0744	SAG0777	-	LKJ	ATP-dependent RNA helicase, DEAD/DEAH box family
	SAK0902	-	LKJ	ATP-dependent RNA helicase, DEAD/DEAH box family
	GBS0797	-	LKJ	hypothetical protein
C0745	SAG0778	-	L	hypothetical protein
	SAK0903	-	L	GIY-YIG domain protein
	GBS0798	-	L	hypothetical protein
C0746	SAG0779	-	R	hypothetical protein
	SAK0904	-	R	hypothetical protein
	GBS0799	-	R	hypothetical protein
C0747	SAG0780	-	I	acyltransferase family protein
	SAK0905	-	I	acyltransferase family protein
	GBS0800	-	I	hypothetical protein

(Continue on next page)

List of homolog clusters in the 3 GBS reference genomes (Cont'd)

ID	Locus tag	Gene	COG	Annotation
C0748	SAG0781	*cel*A	L	competence protein CelA
	SAK0906	-	L	ComE operon protein 1, putative
	GBS0801	-	L	hypothetical protein
C0749	SAG0782	-	R	DNA internalization-related competence protein ComEC/Rec2
	SAK0907	-	R	DNA internalization-related competence protein ComEC/Rec2
	GBS0802	-	R	hypothetical protein
C0750	SAG0783	-	R	hydrolase, haloacid dehalogenase-like family
	SAK0908	-	R	Cof-like hydrolase family protein
	GBS0803	-	R	hypothetical protein
C0751	SAG0784	-	K	sugar-binding transcriptional regulator, LacI family
	SAK0909	-	K	sugar-binding transcriptional regulator, LacI family
	GBS0804	-	K	hypothetical protein
C0752	SAG0785	-	I	hypothetical protein
	SAK0910	-	I	GDXG lipolytic enzyme family protein
	GBS0805	-	I	hypothetical protein
C0753	SAG0786	-	QR	hypothetical protein
	SAK0911	-	QR	hypothetical protein
	GBS0806	-	QR	hypothetical protein
C0754	SAG0787	-	L	DNA polymerase III subunit delta
	SAK0912	*hol*A	L	DNA polymerase III subunit delta
	GBS0807	-	L	DNA polymerase III subunit delta
C0755	SAG0788	*sod*A	P	superoxide dismutase, Fe-Mn
	SAK0913	*sod*A	P	superoxide dismutase, Mn
	GBS0808	*sod*	P	manganese-dependent superoxide dismutase
C0756	SAG0789	-	K	transcriptional antiterminator LicT
	SAK0914	-	K	transcriptional antiterminator, BglG family
	GBS0809	-	K	hypothetical protein
	GBS1332	-	K	hypothetical protein
C0757	SAG0790	-	G	PTS system, beta-glucosides-specific IIABC components
	SAK0915	*bgl*F	G	PTS system, beta-glucoside-specific IIABC component
	GBS0810	-	G	hypothetical protein
C0758	SAG0791	*bgl*A		6-phospho-beta-glucosidase
	SAK0916	-		glycosyl hydrolase, family 1
	GBS0811	-		hypothetical protein
	GBS1329	-		hypothetical protein
C0759	SAG0792	-	KT	hypothetical protein
	SAK0917	-	KT	hypothetical protein
	GBS0812	-	KT	hypothetical protein
C0760	SAG0793	*gar*K		glycerate kinase 2
	SAK0918	-		glycerate kinase
	GBS0813	-		hypothetical protein
C0761	SAG0794	-	GE	permease, GntP family
	SAK0919	-	GE	permease, GntP family
	GBS0814	-	GE	hypothetical protein
C0762	SAG0795	-	V	hypothetical protein
	SAK0920	-	V	hypothetical protein
	GBS0815	-	V	hypothetical protein
C0763	SAG0796	-	K	transcriptional regulator, MarR family
	SAK0921	-	K	transcriptional regulator, MarR family
	GBS0816	-	K	hypothetical protein
C0764	SAG0797	*que*A		S-adenosylmethionine:tRNA ribosyltransferase-isomerase
	SAK0922	*que*A		S-adenosylmethionine:tRNA ribosyltransferase-isomerase
	GBS0817	-		S-adenosylmethionine:tRNA ribosyltransferase-isomerase
C0765	SAG0798	-	S	hypothetical protein
	SAK0923	-	S	hypothetical protein
	GBS0818	-	S	hypothetical protein
C0766	SAG0799	*nag*B	G	glucosamine-6-phosphate isomerase
	SAK0924	*nag*B	G	glucosamine-6-phosphate isomerase
	GBS0819	-	G	hypothetical protein
C0767	SAG0800	-	O	glutathione S-transferase family protein
	SAK0925	-	O	glutathione S-transferase domain protein
	GBS0820	-	O	hypothetical protein
C0768	SAG0801	-	J	ribosomal small subunit pseudouridine synthase A, putative
	SAK0926	-	J	RNA pseudouridine synthase family protein
	GBS0821	-	J	hypothetical protein
C0769	SAG0802	-		hypothetical protein
C0770	SAG0803	-		major facilitator family protein
	SAG1892	-		hypothetical protein
	SAK0927	-		major facilitator family protein
	GBS0822	-		hypothetical protein
	GBS1881	-		hypothetical protein

(Continue on next page)

List of homolog clusters in the 3 GBS reference genomes (Cont'd)

ID	Locus tag	Gene	COG	Annotation
C0771	SAG0804	*coi*A	R	competence protein CoiA
	SAK0928	-	R	competence protein CoiA, putative
	GBS0823	-	R	hypothetical protein
C0772	SAG0805	*pep*B	E	oligoendopeptidase B
	SAK0929	*pep*B	E	group B oligopeptidase PepB
	GBS0824	*pep*B	E	group B oligopeptidase PepB
C0773	SAG0806	-	R	hydrolase, haloacid dehalogenase-like family
	SAK0930	-	R	HAD-superfamily hydrolase, subfamily IA, variant 1 family protein
	GBS0825	-	R	hypothetical protein
C0774	SAG0807	-	R	O-methyltransferase family protein
	SAK0931	-	R	O-methyltransferase family protein
	GBS0826	-	R	hypothetical protein
C0775	SAG0808	*prs*A	O	peptidylprolyl isomerase
	SAK0932	*prs*A	O	peptidylprolyl isomerase
	GBS0827	*prs*A	O	peptidylprolyl isomerase
C0776	SAG0809	-	S	hypothetical protein
	SAK0933	-	S	hypothetical protein
	GBS0828	-	S	hypothetical protein
C0777	SAG0810	*ala*S	J	alanyl-tRNA synthetase
	SAK0934	*ala*S	J	alanyl-tRNA synthetase
	GBS0829	*ala*S	J	alanyl-tRNA synthetase
C0778	SAG0811	-	R	hypothetical protein
	SAK0935	-	R	hypothetical protein
	GBS0830	-	R	hypothetical protein
C0779	SAG0812	-	M	glycosyl transferase, family 8
	SAK0936	-	M	glycosyl transferase, family 8
	GBS0831	-	M	hypothetical protein
C0780	SAG0813	-		hypothetical protein
C0781	SAG0814	-		hypothetical protein
	SAK0937	-		hypothetical protein
	GBS0832	-		hypothetical protein
C0782	SAG0815	-	K	transcriptional regulator, Cro/CI family
	SAG1128	-	K	transcriptional regulator, Cro/CI family
	SAK0938	-	K	DNA-binding protein
	GBS0833	-	K	hypothetical protein
	GBS1196	-	K	hypothetical protein
C0783	SAG0816	-		hypothetical protein
	SAK0939	-		hypothetical protein
	GBS0834	-		hypothetical protein
C0784	SAG0817	-	S	hypothetical protein
	SAK0940	-	S	probable proton-coupled thiamine transporter YuaJ
	GBS0835	-	S	hypothetical protein
C0785	SAG0818	*nrd*F-2	F	ribonucleotide-diphosphate reductase beta subunit
	SAK0942	*nrd*F	F	ribonucleotide-diphosphate reductase beta subunit
	GBS0836	-	F	ribonucleotide-diphosphate reductase beta subunit
C0786	SAG0820	*nrd*H	O	ribonucleoside-diphosphate reductase 2, NrdH-redoxin
	SAK0944	-	O	glutaredoxin-like protein NrdH
	GBS0838	-	O	hypothetical protein
C0787	SAG0821	*pts*H	G	phosphocarrier protein HPr
	SAK0945	*pts*H	G	phosphocarrier protein HPr
	GBS0839	*pts*H	G	hypothetical protein
C0788	SAG0822	*pts*I	G	phosphoenolpyruvate-protein phosphotransferase
	SAK0946	*pts*I	G	phosphoenolpyruvate-protein phosphotransferase
	GBS0840	*pts*I	G	phosphoenolpyruvate:sugar phosphotransferase system enzyme I
C0789	SAG0823	*gap*N	C	glyceraldehyde-3-phosphate dehydrogenase, NADP-dependent
	SAK0947	*gap*N	C	glyceraldehyde-3-phosphate dehydrogenase, NADP-dependent
	GBS0841	-	C	hypothetical protein
C0790	SAG0824	-		polysaccharide deacetylase family protein
	SAK0948	-		polysaccharide deacetylase family protein
	GBS0842	-		hypothetical protein
C0791	SAG0825	-	LKJ	ATP-dependent RNA helicase, DEAD/DEAH box family
	SAK0949	-	LKJ	ATP-dependent RNA helicase, DEAD/DEAH box family
	GBS0843	-	LKJ	hypothetical protein
C0792	SAG0826	*udk*	F	uridine kinase
	SAK0950	*udk*	F	uridine kinase
	GBS0844	-	F	uridine kinase
C0793	SAG0827	-	T	hypothetical protein
	SAK0951	-	T	GAF domain protein
	GBS0845	-	T	hypothetical protein

(Continue on next page)

List of homolog clusters in the 3 GBS reference genomes (Cont'd)

ID	Locus tag	Gene	COG	Annotation
C0794	SAG0828	*dna*X	L	DNA polymerase III subunits gamma and tau
	SAK0952	*dna*X	L	DNA polymerase III subunits gamma and tau
	GBS0846	-	L	DNA polymerase III subunits gamma and tau
C0795	SAG0829	-		hypothetical protein
	GBS0847	-		hypothetical protein
C0796	SAG0830	-	K	biotin–protein ligase
	SAK0953	-	H	biotin–protein ligase
	GBS0848	-	K	biotin–protein ligase
C0797	SAG0831	*met*K	H	S-adenosylmethionine synthetase
	SAK0736	*met*K	H	S-adenosylmethionine synthetase
	SAK0954	*met*K	H	S-adenosylmethionine synthetase
	GBS0849	*met*K	H	S-adenosylmethionine synthetase
C0798	SAG0832	-		hypothetical protein
	SAK0955	-		fibrinogen-binding protein
	GBS0850	-		hypothetical protein
C0799	SAG0833	-		hypothetical protein
	SAK0956	-		hypothetical protein
	GBS0851	-		hypothetical protein
C0800	SAG0834	-		hypothetical protein
	SAK0957	-		hypothetical protein
	GBS0852	-		hypothetical protein
C0801	SAG0835	-	S	hypothetical protein
	SAK0958	-	S	hypothetical protein
	GBS0853	-	S	hypothetical protein
C0802	SAG0836	-	S	hypothetical protein
	SAK0959	-	S	hypothetical protein
	GBS0854	-	S	hypothetical protein
C0803	SAG0837	-	P	ABC transporter, ATP-binding protein
	SAK0960	-	P	ABC transporter, ATP-binding protein
	GBS0855	-	P	hypothetical protein
C0804	SAG0838	-	P	hypothetical protein
	SAK0961	-	P	ABC transporter, permease protein
	GBS0856	-	P	hypothetical protein
C0805	SAG0839	-	K	transcriptional regulator, TenA family
	SAK0962	-	K	TENA/THI-4 family protein
	GBS0857	-	K	hypothetical protein
C0806	SAG0840	*thi*D	H	phosphomethylpyrimidine kinase
	SAK0963	*thi*D	H	phosphomethylpyrimidine kinase
	GBS0858	-	H	phosphomethylpyrimidine kinase
C0807	SAG0841	*thi*M	H	hydroxyethylthiazole kinase
	SAK0964	*thi*M	H	hydroxyethylthiazole kinase
	GBS0859	-	H	hydroxyethylthiazole kinase
C0808	SAG0842	*thi*E	H	thiamine-phosphate pyrophosphorylase
	SAK0965	*thi*E	H	thiamine-phosphate pyrophosphorylase
	GBS0860	-	H	hypothetical protein
C0809	SAG0843	*mur*A-1	M	UDP-N-acetylglucosamine 1-carboxyvinyltransferase
	SAK0966	*mur*A	M	UDP-N-acetylglucosamine 1-carboxyvinyltransferase
	GBS0861	-	M	UDP-N-acetylglucosamine 1-carboxyvinyltransferase
C0810	SAG0844	-	J	acetyltransferase, GNAT family
	SAK0967	-	J	acetyltransferase, GNAT family
	GBS0862	-	J	hypothetical protein
C0811	SAG0845	-	K	CBS domain protein
	SAK0968	-	K	DRTGG domain/CBS domain protein
	GBS0863	-	K	hypothetical protein
C0812	SAG0846	*map*	J	methionine aminopeptidase
	SAK0969	*map*	J	methionine aminopeptidase
	GBS0864	-	J	methionine aminopeptidase
C0813	SAG0847	-	S	ribonuclease BN, putative
	SAK0970	-	S	ribonuclease BN, putative
	GBS0865	-	S	hypothetical protein
C0814	SAG0848	-	S	GtrA family protein
	SAK0971	-	S	GtrA family protein
	GBS0866	-	S	hypothetical protein
C0815	SAG0849	-	S	hypothetical protein
	SAK0972	-	S	hypothetical protein
	GBS0867	-	S	hypothetical protein
C0816	SAG0850	*lig*A	L	DNA ligase
	SAK0973	*lig*A	L	DNA ligase
	GBS0868	-	L	DNA ligase

(Continue on next page)

List of homolog clusters in the 3 GBS reference genomes (Cont'd)

ID	Locus tag	Gene	COG	Annotation
C0817	SAG0851	-	IR	bmrU protein, putative
	SAK0974	-	IR	conserved hypothetical protein TIGR00147
	GBS0869	-	IR	hypothetical protein
C0818	SAG0852	-	G	pullulanase, putative
	SAK0975	*pul*A	G	pullulanase, type I
	GBS0870	-	G	hypothetical protein
C0819	SAG0853	*glg*B	G	glycogen branching enzyme
	SAK0976	*glg*B	G	glycogen branching enzyme
	GBS0871	-	G	glycogen branching enzyme
C0820	SAG0854	*glg*C	G	glucose-1-phosphate adenylyltransferase
	SAK0977	*glg*C	G	glucose-1-phosphate adenylyltransferase
	SAK0978	*glg*D	G	glucose-1-phosphate adenylyltransferase, GlgD subunit
	GBS0872	*glg*C	G	glucose-1-phosphate adenylyltransferase
	GBS0873	-	G	hypothetical protein
C0821	SAG0856	*glg*A	G	glycogen synthase
	SAK0979	*glg*A	G	glycogen synthase
	GBS0874	-	G	glycogen synthase
C0822	SAG0857	*atp*E		ATP synthase F0, C subunit
	SAK0980	*atp*E		ATP synthase F0, C subunit
	GBS0875	*atp*E		H+-transporting ATP synthase c chain
C0823	SAG0858	*atp*B		ATP synthase subunit A
	SAK0981	*atp*B		ATP synthase subunit A
	GBS0876	*atp*B		ATP synthase subunit A
C0824	SAG0859	*atp*F	C	ATP synthase subunit B
	SAK0982	*atp*F	C	ATP synthase subunit B
	GBS0877	*atp*F	C	ATP synthase subunit B
C0825	SAG0860	*atp*H	C	ATP synthase subunit D
	SAK0983	*atp*H	C	ATP synthase subunit D
	GBS0878	*atp*H	C	ATP synthase subunit D
C0826	SAG0861	*atp*A	C	ATP synthase subunit A
	SAK0984	*atp*A	C	ATP synthase subunit A
	GBS0879	*atp*A	C	ATP synthase subunit A
C0827	SAG0862	*atp*G	C	ATP synthase subunit C
	SAK0985	*atp*G	C	ATP synthase subunit C
	GBS0880	*atp*G	C	ATP synthase subunit C
C0828	SAG0863	*atp*D	C	ATP synthase subunit B
	SAK0986	*atp*D	C	ATP synthase subunit B
	GBS0881	*atp*D	C	ATP synthase subunit B
C0829	SAG0864	*atp*C		ATP synthase subunit epsilon
	SAK0987	*atp*C		ATP synthase subunit epsilon
	GBS0882	*atp*C		ATP synthase subunit epsilon
C0830	SAG0865	-		hypothetical protein
	SAK0988	-		conserved hypothetical protein TIGR02327
C0831	SAG0866	*mur*A-2	M	UDP-N-acetylglucosamine 1-carboxyvinyltransferase
	SAK0989	*mur*A	M	UDP-N-acetylglucosamine 1-carboxyvinyltransferase
	GBS0883	*mur*A	M	UDP-N-acetylglucosamine 1-carboxyvinyltransferase
C0832	SAG0867	-		hypothetical protein
	SAK0990	-		hypothetical protein
	GBS0884	-		hypothetical protein
C0833	SAG0868	*end*A		DNA-entry nuclease
	SAK0991	*end*A		DNA-entry nuclease
	GBS0885	-		hypothetical protein
C0834	SAG0869	*phe*S	J	phenylalanyl-tRNA synthetase alpha subunit
	SAK0992	*phe*S	J	phenylalanyl-tRNA synthetase alpha subunit
	GBS0886	*phe*S	J	phenylalanyl-tRNA synthetase alpha subunit
C0835	SAG0870	-		acetyltransferase, GNAT family
	SAK0993	-		acetyltransferase, GNAT family
	GBS0887	-		hypothetical protein
C0836	SAG0871	*phe*T	R	phenylalanyl-tRNA synthetase beta subunit
	SAK0994	*phe*T	R	phenylalanyl-tRNA synthetase beta subunit
	GBS0888	*phe*T	J	phenylalanyl-tRNA synthetase beta subunit
C0837	SAG0872	-		hypothetical protein
	SAK0995	-		hypothetical protein
C0838	SAG0873	*rex*B	L	exonuclease RexB
	SAK0996	*rex*B	L	exonuclease RexB
	GBS0890	-	L	hypothetical protein
C0839	SAG0874	*rex*A	L	exonuclease RexA
	SAK0997	*rex*A	L	exonuclease RexA
	GBS0891	-	L	hypothetical protein

(Continue on next page)

List of homolog clusters in the 3 GBS reference genomes (Cont'd)

ID	Locus tag	Gene	COG	Annotation
C0840	SAG0875	-	P	magnesium transporter, CorA family
	SAK0998	-	P	metal ion transporter, CorA family
	GBS0892	-	P	hypothetical protein
C0841	SAG0876	*trm*E	R	tRNA modification GTPase
	SAK0999	*trm*E	R	tRNA modification GTPase
	GBS0893	-	R	tRNA modification GTPase
C0842	SAG0877	-	R	ABC transporter, ATP-binding protein
	SAK1000	-	R	ABC transporter, ATP-binding protein
	GBS0894	-	R	hypothetical protein
C0843	SAG0878	-	C	acetoin dehydrogenase, thymine PPi dependent, E1 component, alpha subunit
	SAK1001	-	C	acetoin dehydrogenase, TPP-dependent, E1 component, alpha subunit, putative
	GBS0895	-	C	hypothetical protein
C0844	SAG0879	-	C	acetoin dehydrogenase, thymine PPi dependent, E1 component, beta subunit
	SAK1002	-	C	acetoin dehydrogenase, TPP-dependent, E1 component, beta subunit, putative
	GBS0896	-	C	hypothetical protein
C0845	SAG0880	-	C	dihydrolipoamide acetyltransferase
	SAK1003	-	C	dihydrolipoamide acetyltransferase
	GBS0897	-	C	dihydrolipoamide acetyltransferase
C0846	SAG0881	-	C	acetoin dehydrogenase, thymine PPi dependent, E3 component, dihydrolipoamide dehydrogenase
	SAK1004	-	C	acetoin dehydrogenase, TPP-dependent, E3 component, dihydrolipoamide dehydrogenase, putative
	GBS0898	-	C	hypothetical protein
C0847	SAG0882	*lpl*A-1	H	lipoate-protein ligase A
	SAK1005	*lpl*A	H	lipoate-protein ligase A
	GBS0899	-	H	hypothetical protein
C0848	SAG0883	-	R	cobyric acid synthase, putative
	SAK1006	-	R	glutamine amidotransferase domain protein
	GBS0900	-	R	hypothetical protein
C0849	SAG0884	-	M	mur ligase family protein
	SAK1007	-	M	mur ligase family protein
	GBS0901	-	M	hypothetical protein
C0850	SAG0885	-		conserved hypothetical protein TIGR00159
	SAK1008	-		conserved hypothetical protein TIGR00159
	GBS0902	-		hypothetical protein
C0851	SAG0886	-	S	hypothetical protein
	SAK1009	-	S	hypothetical protein
	GBS0903	-	S	hypothetical protein
C0852	SAG0887	-	G	phosphoglucomutase/phosphomannomutase family protein
	SAK1010	*glm*M	G	phosphoglucosamine mutase
	GBS0904	-	G	hypothetical protein
C0853	SAG0888	-	S	hypothetical protein
	SAK1011	-	S	hypothetical protein
	GBS0905	-	S	hypothetical protein
C0854	SAG0889	-		hypothetical protein
	SAK1012	-		hypothetical protein
	GBS0906	-		hypothetical protein
C0855	SAG0890	-	H	coproporphyrinogen III oxidase
	SAK1013	-	H	coproporphyrinogen III oxidase
	GBS0907	-	H	coproporphyrinogen III oxidase
C0856	SAG0891	-	I	hypothetical protein
	SAK1014	-	I	thioesterase, Acyl-ACP family
	GBS0908	-	I	hypothetical protein
C0857	SAG0892	-	G	hydrolase, haloacid dehalogenase-like family
	SAK1015	-	G	HAD-superfamily hydrolase, subfamily IIA
	GBS0909	-	G	hypothetical protein
C0858	SAG0893	-		hypothetical protein
	SAG1261	-		hypothetical protein
	SAK1016	-		integral membrane protein TIGR01906
	GBS0910	-		hypothetical protein
C0859	SAG0894	-	S	hypothetical protein
	SAK1017	-	S	CRISPR-associated SAG0894 family protein
	GBS0911	-	S	hypothetical protein
C0860	SAG0895	-	L	lipoyl-binding domain protein
	SAK1018	*cas*1	L	CRISPR-associated protein Cas1
	GBS0912	-	L	hypothetical protein
C0861	SAG0896	-	S	oxidoreductase, putative
	SAK1019	-	S	CRISPR-associated protein Cas2, putative
	GBS0913	-	S	hypothetical protein

(Continue on next page)

List of homolog clusters in the 3 GBS reference genomes (Cont'd)

ID	Locus tag	Gene	COG	Annotation
C0862	SAG0897	-		hypothetical protein
	SAK1020	-		CRISPR-associated SAG0897 family protein
	GBS0914	-		hypothetical protein
C0863	SAG0898	-		hypothetical protein
C0864	SAG0899	-		hypothetical protein
C0865	SAG0900	-		hypothetical protein
C0866	SAG0901	-		hypothetical protein
C0867	SAG0902	-		hypothetical protein
C0868	SAG0903	-		hypothetical protein
C0869	SAG0904	-		hypothetical protein
C0870	SAG0905	*ndk*		nucleoside diphosphate kinase
	SAK1021	*ndk*		nucleoside diphosphate kinase
	GBS0916	*ndk*		nucleoside diphosphate kinase
C0871	SAG0906	*lep*A	M	GTP-binding protein LepA
	SAK1022	*lep*A	M	GTP-binding protein LepA
	GBS0917	-	M	GTP-binding protein LepA
C0872	SAG0907	-	S	protein of unknown function/lipoprotein, putative
	SAK1023	-	S	leucine-rich repeat protein
	GBS0918	-	S	hypothetical protein
C0873	SAG0908	-	R	HD domain protein
	SAK1024	-	R	HD domain protein
	GBS0919	-	R	hypothetical protein
C0874	SAG0909	-		acetyltransferase, GNAT family
	SAK1025	-		acetyltransferase, GNAT family
	GBS0920	-		hypothetical protein
C0875	SAG0910	-		methionine sulfoxide reductase B
	SAK1026	*msr*B		methionine sulfoxide reductase B
	GBS0921	-		methionine sulfoxide reductase B
C0876	SAG0911	-	P	cation-transporting ATPase, E1-E2 family
	SAK1027	-	P	cation-transporting ATPase, P-type, HAD superfamily, subfamily IC
	GBS0922	-	P	hypothetical protein
C0877	SAG0912	-		hypothetical protein
	SAK1028	-		hypothetical protein
	GBS0923	-		hypothetical protein
C0878	SAG0913	*cat*	R	chloramphenicol acetyltransferase
	SAK1029	-	R	transferase, hexapeptide repeat family
	GBS0924	-	R	hypothetical protein
C0879	SAG0914	-	S	hypothetical protein
	SAK1031	-	S	hypothetical protein
	GBS0925	-	S	hypothetical protein
C0880	SAG0915	-	L	Tn916, transposase
	SAG1885	-	L	prophage LambdaSa2, site-specific recombinase, phage integrase family
	SAG1986	-	L	site-specific recombinase, phage integrase family
	SAG1993	-	L	site-specific recombinase, phage integrase family
	SAK1943	-	L	site-specific recombinase, phage integrase family
	SAK2059	-	L	site-specific recombinase, phage integrase family
	SAK2094	-	L	prophage Sa05, site-specific recombinase, phage integrase family
	GBS0482	-		hypothetical protein
	GBS1224	-	L	hypothetical protein
	GBS1969	-		hypothetical protein
	GBS2073	-	L	hypothetical protein
C0881	SAG0916	-		Tn916, excisionase
C0882	SAG0917	-		Tn916, hypothetical protein
C0883	SAG0918	-		Tn916, hypothetical protein
C0884	SAG0919	-	K	Tn916, hypothetical protein
C0885	SAG0920	-		Tn916, hypothetical protein
C0886	SAG0921	-	K	Tn916, transcriptional regulator, putative
C0887	SAG0922	-		Tn916, hypothetical protein
C0888	SAG0923	*tet*M	J	tetracycline resistance protein
	SAG1769	*fus*A	J	elongation factor EF-2
	SAK1791	*fus*A	J	elongation factor EF-2
	GBS1812	*fus*A	J	elongation factor EF-2
C0889	SAG0924	-		Tn916, tetM leader peptide
C0890	SAG0925	-		Tn916, hypothetical protein
C0891	SAG0926	-	M	Tn916, NLP/P60 family protein
	SAG1072	-		hypothetical protein
	SAK1158	-		pneumococcal vaccine antigen A, putative
	GBS1104	-		hypothetical protein
C0892	SAG0927	-		hypothetical protein
C0893	SAG0929	-		Tn916, hypothetical protein

(Continue on next page)

List of homolog clusters in the 3 GBS reference genomes (Cont'd)

ID	Locus tag	Gene	COG	Annotation
C0894	SAG0930	-		Tn916, hypothetical protein
C0895	SAG0931	-		Tn916, hypothetical protein
C0896	SAG0932	-	L	Tn916, transcriptional regulator, putative
	SAG1988	-	L	hypothetical protein
	SAG2114	-	L	hypothetical protein
	SAK1945	-	L	replication initiation factor family protein
C0897	SAG0933	-	D	Tn916, FtsK/SpoIIIE family protein
	SAK2056	-	D	FtsK/SpoIIIE family protein
	GBS1320	-	D	hypothetical protein
	GBS2069	-	D	hypothetical protein
C0898	SAG0934	-		Tn916, hypothetical protein
C0899	SAG0935	-		Tn916, hypothetical protein
C0900	SAG0936	-		Tn916, hypothetical protein
C0901	SAG0938	-	K	transcriptional regulator, GntR family
	SAK1034	-	K	transcriptional regulator, GntR family
	GBS0928	-	K	hypothetical protein
C0902	SAG0939	*dna*E	L	DNA polymerase III subunit alpha
	SAK1035	*dna*E	L	DNA polymerase III subunit alpha
	GBS0929	*dna*E	L	DNA polymerase III subunit alpha
C0903	SAG0940	*pfk*A	G	6-phosphofructokinase
	SAK1036	*pfk*A	G	6-phosphofructokinase
	GBS0930	*pfk*A	G	6-phosphofructokinase
C0904	SAG0941	*pyk*		pyruvate kinase
	SAK1037	*pyk*		pyruvate kinase
	GBS0931	-		pyruvate kinase
C0905	SAG0942	-		signal peptidase I, putative
	SAK1038	*lep*B		signal peptidase I
	GBS0932	-		hypothetical protein
C0906	SAG0943	-		hypothetical protein
	SAK1039	-		hypothetical protein
C0907	SAG0944	*glm*S	M	D-fructose-6-phosphate amidotransferase
	SAK1040	*glm*S	M	D-fructose-6-phosphate amidotransferase
	GBS0933	-	M	D-fructose-6-phosphate amidotransferase
C0908	SAG0945	-	L	IS1548 transposase
C0909	SAG0946	*phn*A	P	phnA protein
	SAK1041	*phn*A	P	PhnA protein
	GBS0934	-	P	hypothetical protein
C0910	SAG0947	-	E	amino acid ABC transporter, permease protein
	SAK1042	-	E	polar amino acid uptake (PAAT) family ABC transporter, permease protein
	GBS0935	-	E	hypothetical protein
C0911	SAG0948	-	E	amino acid ABC transporter, ATP-binding protein
	SAK1043	-	E	polar amino acid uptake (PAAT) family ABC transporter, ATP-binding protein
	GBS0936	-	E	hypothetical protein
C0912	SAG0949	-	ET	amino acid ABC transporter, amino acid-binding protein
	SAK1044	-	ET	polar amino acid uptake (PAAT) family ABC transporter, amino acid-binding protein
	GBS0937	-	ET	hypothetical protein
C0913	SAG0950	*rps*T	J	30S ribosomal protein S20
	SAK1045	*rps*T	J	30S ribosomal protein S20
	GBS0938	*rps*T	J	30S ribosomal protein S20
C0914	SAG0951	*coa*A	H	pantothenate kinase
	SAK1046	*coa*A	H	pantothenate kinase
	GBS0939	-	H	pantothenate kinase
C0915	SAG0952	-	J	hypothetical protein
	SAK1047	-	J	methyltransferase domain protein
	GBS0940	-	J	hypothetical protein
C0916	SAG0953	*cdd*	F	cytidine deaminase
	SAK1048	*cdd*	F	cytidine deaminase
	GBS0941	-	F	cytidine deaminase
C0917	SAG0954	-	R	protein of unknown function/lipoprotein, putative
	SAK1049	-	R	membrane protein, Bmp family
	GBS0942	-	R	hypothetical protein
C0918	SAG0955	-	R	sugar ABC transporter, ATP-binding protein
	SAK1050	-	R	carbohydrate uptake 2 (CUT2) family, carbohydrate-binding protein
	GBS0943	-	R	hypothetical protein
C0919	SAG0956	-	R	sugar ABC transporter, permease protein, putative
	SAK1051	-	R	carbohydrate uptake 2 (CUT2) family, permease protein
	GBS0944	-	R	hypothetical protein

(Continue on next page)

List of homolog clusters in the 3 GBS reference genomes (Cont'd)

ID	Locus tag	Gene	COG	Annotation
C0920	SAG0957	-	R	sugar ABC transporter, permease protein, putative
	SAK1052	-	R	ABC transporter, permease protein, carbohydrate uptake 2 (CUT2) family, putative
	GBS0945	-	R	hypothetical protein
C0921	SAG0958	*nox*	R	NADH oxidase
	SAK1053	*nox*	R	NADH oxidase, water-forming
	GBS0946	-	R	hypothetical protein
C0922	SAG0959	*ldh*	C	L-lactate dehydrogenase
	SAK1054	-	C	L-lactate dehydrogenase
	GBS0947	-	C	L-lactate dehydrogenase
C0923	SAG0960	*gyr*A	L	DNA gyrase subunit A
	SAK1055	*gyr*A	L	DNA gyrase subunit A
	GBS0948	*gyr*A	L	DNA gyrase subunit A
C0924	SAG0961	*srt*A	M	sortase SrtA
	SAK1056	-	M	sortase family protein
	GBS0949	-	M	hypothetical protein
C0925	SAG0962	-	E	glyoxylase family protein
	SAK1057	-	E	glyoxylase family protein
	GBS0950	-	E	hypothetical protein
C0926	SAG0963	-	S	hypothetical protein
	SAK1058	-	S	hypothetical protein
	GBS0951	-	S	hypothetical protein
C0927	SAG0964	-	P	Na+/H+ exchanger family protein
	SAK1059	-	P	Na+/H+ antiporter
	GBS0952	-	P	hypothetical protein
C0928	SAG0967	*gua*A	F	bifunctional GMP synthase/glutamine amidotransferase protein
	SAK1062	*gua*A	F	bifunctional GMP synthase/glutamine amidotransferase protein
	GBS0953	*gua*A	F	bifunctional GMP synthase/glutamine amidotransferase protein
C0929	SAG0968	-	K	transcriptional regulator, GntR family
	SAK1063	-	K	transcriptional regulator, GntR family
	GBS0954	-	K	hypothetical protein
C0930	SAG0969	*gid*	J	glucose-inhibited division protein A
	SAK1064	*gid*	J	glucose-inhibited division protein A
	GBS0955	-	J	glucose-inhibited division protein A
C0931	SAG0970	-		acetyltransferase, GNAT family
	SAK1065	-		acetyltransferase, GNAT family
	GBS0956	-		hypothetical protein
C0932	SAG0971	-	P	protein of unknown function/lipoprotein, putative
	SAK1066	-	P	lipoprotein, NPLA family
	GBS0957	-	P	hypothetical protein
C0933	SAG0973	-	M	nisin-resistance protein, putative
	SAK1068	-	M	nisin resistance protein Nsr, putative
	GBS0959	-	M	hypothetical protein
C0934	SAG0974	-	V	ABC transporter, ATP-binding protein
	SAK1069	-	V	ABC transporter, ATP-binding protein
	GBS0961	-	V	hypothetical protein
C0935	SAG0975	-		ABC transporter, permease protein, putative
	SAK1070	-		ABC transporter, permease protein, putative
	GBS0962	-		hypothetical protein
C0936	SAG0976	-	TK	DNA-binding response regulator
	SAK1071	-	TK	DNA-binding response regulator
	GBS0963	-	TK	hypothetical protein
C0937	SAG0977	-	T	sensor histidine kinase
	SAK1072	-	T	sensor histidine kinase
	GBS0964	-	T	hypothetical protein
C0938	SAG0978	-	L	tyrosine recombinase
	SAK1073	-	L	tyrosine recombinase
	GBS0965	-	L	tyrosine recombinase
C0939	SAG0980	-		hypothetical protein
	SAK1075	-		hypothetical protein
	GBS0967	-		hypothetical protein
C0940	SAG0981	-		satD protein
	SAK1076	*sat*D		satD protein
	GBS0968	-		hypothetical protein
C0941	SAG0982	*ffh*	U	signal recognition particle protein Ffh
	SAK1077	*ffh*	U	signal recognition particle protein
	GBS1017	-	U	hypothetical protein
C0942	SAG0983	-	S	hypothetical protein
	SAK1078	-	S	hypothetical protein
	GBS1018	-	S	hypothetical protein

(Continue on next page)

List of homolog clusters in the 3 GBS reference genomes (Cont'd)

ID	Locus tag	Gene	COG	Annotation
C0943	SAG0984	-	T	sensor histidine kinase CiaH
	SAK1079	*cia*H	T	sensor histidine kinase CiaH
	GBS1019	-	T	hypothetical protein
C0944	SAG0985	*cia*R	TK	DNA-binding response regulator CiaR
	SAK1080	*cia*R	TK	DNA-binding response regulator CiaR
	GBS1020	-	TK	hypothetical protein
C0945	SAG0986	*pep*N	E	aminopeptidase N
	SAK1081	*pep*N	E	aminopeptidase N
	GBS1021	-	E	hypothetical protein
C0946	SAG0987	*pho*U	P	phosphate transport system regulatory protein PhoU
	SAK1082	*pho*U	P	phosphate transport system regulatory protein PhoU
	GBS1022	-	P	hypothetical protein
C0947	SAG0988	-	P	phosphate ABC transporter, ATP-binding protein PstB, putative
	SAK1083	-	P	phosphate ABC transporter, ATP-binding protein PstB, putative
	GBS1023	-	P	hypothetical protein
C0948	SAG0989	-	P	phosphate ABC transporter, ATP-binding protein PstB, putative
	SAK1084	*pst*B	P	phosphate ABC transporter, ATP-binding protein PstB
	GBS1024	-	P	hypothetical protein
C0949	SAG0990	-	P	phosphate ABC transporter, permease protein PstA, putative
	SAK1085	*pst*A	P	phosphate ABC transporter, permease protein PtsA
	GBS1025	-	P	hypothetical protein
C0950	SAG0991	-	P	phosphate ABC transporter, permease protein
	SAK1086	*pst*C	P	phosphate ABC transporter, permease protein PstC
	GBS1026	-	P	hypothetical protein
C0951	SAG0992	-	P	phosphate ABC transporter, phosphate-binding protein
	SAK1087	-	P	phosphate ABC transporter, phosphate-binding protein
	GBS1027	-	P	hypothetical protein
C0952	SAG0993	-	S	NOL1/NOP2/sun family protein
	SAK1088	-	S	NOL1/NOP2/sun family putative RNA methylase
	GBS1028	-	S	hypothetical protein
C0953	SAG0994	-	G	inositol monophosphatase family protein
	SAK1089	-	G	inositol monophosphatase family protein
	GBS1029	-	G	hypothetical protein
C0954	SAG0995	-		hypothetical protein
	SAK1090	-		hypothetical protein
	GBS1030	-		hypothetical protein
C0955	SAG0996	*spx*A	P	transcriptional regulator Spx
	SAK1091	*spx*A	P	transcriptional regulator Spx
	GBS1031	*spx*A	P	transcriptional regulator Spx
C0956	SAG0997	-	H	riboflavin kinase/flavin adenine dinucleotide synthase
	SAK1092	*rib*F	H	riboflavin kinase/flavin adenine dinucleotide synthase
	GBS1032	-	H	riboflavin kinase/flavin adenine dinucleotide synthase
C0957	SAG0998	*tru*B	J	tRNA pseudouridine synthase B
	SAK1093	*tru*B	J	tRNA pseudouridine synthase B
	GBS1033	*tru*B	J	tRNA pseudouridine synthase B
C0958	SAG0999	-		acetyltransferase, GNAT family
	SAK1094	-		acetyltransferase, GNAT family
	GBS1034	-		hypothetical protein
C0959	SAG1000	-	S	hypothetical protein
	SAK1095	-	S	hypothetical protein
	GBS1035	-	S	hypothetical protein
C0960	SAG1001	-	V	hypothetical protein
	SAK1096	-	V	hypothetical protein
	GBS1036	-	V	hypothetical protein
C0961	SAG1002	-		protease, putative
	SAK1097	-		CAAX amino terminal protease family protein
	GBS1037	-		hypothetical protein
C0962	SAG1003	-	V	permease, putative
	SAK1098	-	V	ABC transporter, permease protein, putative
	GBS1038	-	V	hypothetical protein
C0963	SAG1004	-	V	ABC transporter, ATP-binding protein
	SAK1099	-	V	ABC transporter, ATP-binding protein
	GBS1039	-	V	hypothetical protein
C0964	SAG1005	*top*A	L	DNA topoisomerase I
	SAK1100	*top*A	L	DNA topoisomerase I
	GBS0387	-	L	hypothetical protein
	GBS0717	-	L	hypothetical protein
	GBS0992	-	L	hypothetical protein
	GBS1040	-	L	DNA topoisomerase I

(Continue on next page)

List of homolog clusters in the 3 GBS reference genomes (Cont'd)

ID	Locus tag	Gene	COG	Annotation
C0965	SAG1006	*dpr*A	LU	DprA/SMF protein, putative DNA processing factor
	SAK1101	-	LU	DNA processing protein DprA, putative
	GBS1041	-	LU	hypothetical protein
C0966	SAG1007	-	P	iron-compound ABC transporter, iron-compound-binding protein
	SAK1102	-	P	iron chelate uptake ABC transporter, iron chelate-binding protein
	GBS1042	-	P	hypothetical protein
C0967	SAG1008	-	P	iron compound ABC transporter, ATP-binding protein
	SAK1103	-	P	iron chelate uptake ABC transporter, ATP-binding protein
	GBS1043	-	P	hypothetical protein
C0968	SAG1009	-	P	iron compound ABC transporter, permease protein
	SAK1104	-	P	iron chelate uptake ABC transporter, permease protein
	GBS1044	-	P	hypothetical protein
C0969	SAG1010	-	P	iron compound ABC transporter, permease protein
	SAK1105	-	P	iron chelate uptake ABC transporter, permease protein
	GBS1045	-	P	hypothetical protein
C0970	SAG1011	-	R	acetyltransferase, CysE/LacA/LpxA/NodL family
	SAK1106	-	R	maltose O-acetyltransferase
	GBS1046	-	R	hypothetical protein
C0971	SAG1012	*rnh*B	L	ribonuclease HII
	SAK1107	*rnh*B	L	ribonuclease HII
	GBS1047	*rnh*B	L	ribonuclease HII
C0972	SAG1013	-	R	GTP-binding protein
	SAK1108	-	R	GTP-binding protein
	GBS1048	-	R	hypothetical protein
C0973	SAG1014	-	S	hypothetical protein
	SAK1109	-	S	ErfK/YbiS/YcfS/YnhG family protein
	GBS1049	-	S	hypothetical protein
C0974	SAG1015	-	T	carbon starvation protein CstA, putative
	SAK1110	*cst*A	T	carbon starvation protein CstA
	GBS1050	-	T	hypothetical protein
C0975	SAG1016	-	KT	response regulator
	SAK1111	-	KT	DNA-binding response regulator
	GBS1051	-	KT	similar to two-component response regulator LytR
C0976	SAG1017	-	T	sensor histidine kinase, putative
	SAK1112	-	T	sensor histidine kinase
	GBS1052	-	T	hypothetical protein
C0977	SAG1018	-		lipoprotein, putative
C0978	SAG1019	-		hypothetical protein
C0979	SAG1020	-		lipoprotein, putative
	GBS1053	-		hypothetical protein
	GBS1054	-		hypothetical protein
C0980	SAG1021	-		hypothetical protein
	GBS1055	-		hypothetical protein
C0981	SAG1022	-		hypothetical protein
	GBS1056	-		hypothetical protein
C0982	SAG1023	-		hypothetical protein
C0983	SAG1024	-		lipoprotein, putative
	GBS1057	-		hypothetical protein
C0984	SAG1025	-		hypothetical protein
	SAG1033	-	D	FtsK/SpoIIIE family protein
	SAG1034	-		hypothetical protein
	SAK1123	-	D	FtsK/SpoIIIE family protein
	GBS1068	-	D	hypothetical protein
	GBS1069	-		hypothetical protein
C0985	SAG1027	-	S	hypothetical protein
	SAG1698	-	S	hypothetical protein
	SAK1115	-	S	hypothetical protein
	SAK1118	-	S	hypothetical protein
	SAK1708	-	S	hypothetical protein
	GBS1062	-	S	hypothetical protein
C0986	SAG1028	-		hypothetical protein
	GBS1063	-		hypothetical protein
C0987	SAG1029	-		hypothetical protein
	GBS1064	-		hypothetical protein
C0988	SAG1030	-		hypothetical protein
	SAK1120	-		hypothetical protein
	GBS1065	-		hypothetical protein
C0989	SAG1031	-		hypothetical protein
	GBS1066	-		hypothetical protein

(Continue on next page)

List of homolog clusters in the 3 GBS reference genomes (Cont'd)

ID	Locus tag	Gene	COG	Annotation
C0990	SAG1032	-		hypothetical protein
	SAK1122	-		hypothetical protein
	GBS1067	-		hypothetical protein
C0991	SAG1035	-	S	hypothetical protein
	SAK1124	-	S	hypothetical protein
	GBS1070	-	S	hypothetical protein
C0992	SAG1036	-		hypothetical protein
	SAK1125	-		hypothetical protein
	GBS1071	-		hypothetical protein
C0993	SAG1037	-		hypothetical protein
	SAK1126	-		hypothetical protein
	GBS1072	-		hypothetical protein
C0994	SAG1038	-	S	phage infection protein, putative
	SAK1127	-	S	phage infection protein, putative
	GBS1073	-	S	hypothetical protein
C0995	SAG1039	-	S	hypothetical protein
	SAK1128	-	S	hypothetical protein
	SAK1129	-	S	hypothetical protein
	GBS1074	-	S	hypothetical protein
C0996	SAG1040	-	UI	hypothetical protein
	SAK1130	-	UI	hypothetical protein
	GBS1075	-	UI	hypothetical protein
C0997	SAG1041	-		hypothetical protein
	SAK1131	-		hypothetical protein
	GBS1076	-		hypothetical protein
C0998	SAG1042	*car*B	EF	carbamoyl-phosphate synthase large subunit
	SAK1132	*car*B	EF	carbamoyl-phosphate synthase large subunit
	GBS1077	*car*B	EF	carbamoyl-phosphate synthase large subunit
C0999	SAG1043	*car*A-1	EF	carbamoyl-phosphate synthase small subunit
	SAK1133	*car*A	EF	carbamoyl-phosphate synthase small subunit
	GBS1078	-	EF	carbamoyl-phosphate synthase small subunit
C1000	SAG1044	*pyr*B	F	aspartate carbamoyltransferase catalytic subunit
	SAK1134	*pyr*B	F	aspartate carbamoyltransferase catalytic subunit
	GBS1079	*pyr*B	F	aspartate carbamoyltransferase catalytic subunit
C1001	SAG1045	*pyr*C	F	dihydroorotase
	SAK1135	*pyr*C	F	dihydroorotase
	GBS1080	*pyr*C	F	dihydroorotase
C1002	SAG1046	*pyr*E	F	orotate phosphoribosyltransferase
	SAK1136	*pyr*E	F	orotate phosphoribosyltransferase
	GBS1081	*pyr*E	F	orotate phosphoribosyltransferase
C1003	SAG1047	*pyr*F	F	orotidine 5'-phosphate decarboxylase
	SAK1137	*pyr*F	F	orotidine 5'-phosphate decarboxylase
	GBS1082	*pyr*F	F	orotidine 5'-phosphate decarboxylase
C1004	SAG1048	-	S	hypothetical protein
	SAK1138	-	S	hypothetical protein
	GBS1083	-	S	hypothetical protein
C1005	SAG1049	-	R	ABC transporter, ATP-binding protein
	SAK1139	-	R	ABC transporter, ATP-binding protein
	GBS1084	-	R	hypothetical protein
	GBS1742	-	P	hypothetical protein
C1006	SAG1051	*asd*	E	aspartate-semialdehyde dehydrogenase
	SAK1141	*asd*	E	aspartate-semialdehyde dehydrogenase
	GBS1086	-	E	aspartate-semialdehyde dehydrogenase
C1007	SAG1052	-		cell wall surface anchor family protein, putative
	SAK1142	-		fibrinogen-binding protein, putative
	GBS1087	-		hypothetical protein
C1008	SAG1053	-		hypothetical protein
C1009	SAG1054	*cls*	I	cardiolipin synthetase
	SAK1143	-	I	cardiolipin synthase, putative
	GBS1088	-	I	hypothetical protein
C1010	SAG1055	*fhs*	F	formate–tetrahydrofolate ligase
	SAK1144	*fhs*	F	formate–tetrahydrofolate ligase
	GBS1089	-	F	hypothetical protein
C1011	SAG1056	*lpl*A-2	H	lipoate-protein ligase A
	SAK1145	*lpl*A	H	lipoate-protein ligase A
	GBS1090	-	H	hypothetical protein
C1012	SAG1057	-	K	hypothetical protein
	SAK1146	-	K	hypothetical protein
	GBS1091	-	K	hypothetical protein

(Continue on next page)

List of homolog clusters in the 3 GBS reference genomes (Cont'd)

ID	Locus tag	Gene	COG	Annotation
C1013	SAG1058	-		hypothetical protein
	SAK1147	-		hypothetical protein
	GBS1092	-		hypothetical protein
C1014	SAG1059	-	E	glycine cleavage system H protein, putative
	SAK1148	-	E	GcvH family protein
	GBS1093	-	E	hypothetical protein
C1015	SAG1060	-	C	bacterial luciferase family protein
	SAK1149	-	C	bacterial luciferase family protein
	GBS1094	-	C	hypothetical protein
C1016	SAG1061	-	C	oxidoreductase, FMN-binding
	SAK1150	-	C	oxidoreductase, FAD/FMN-binding
	GBS1095	-	C	hypothetical protein
C1017	SAG1062	-	H	hypothetical protein
	SAK1151	-	H	hypothetical protein
	GBS1096	-	H	hypothetical protein
C1018	SAG1063	-	H	hypothetical protein
	SAK1152	*coa*B	H	hypothetical protein
	GBS1097	-	H	hypothetical protein
C1019	SAG1064	-	H	phosphopantothenoylcysteine synthase/decarboxylase
	SAK1153	*coa*C	H	phosphopantothenoylcysteine synthase/decarboxylase
	GBS1098	-	H	phosphopantothenoylcysteine synthase/decarboxylase
C1020	SAG1065	-	S	hypothetical protein
	SAK1154	-	S	hypothetical protein
	GBS1099	-	S	hypothetical protein
C1021	SAG1066	*pgm*	G	phosphoglucomutase
	SAK1155	-	G	phosphoglucomutase/phosphomannomutase family protein
	GBS1100	-	G	hypothetical protein
C1022	SAG1067	-		IS861, transposase OrfA
	SAG1526	-		IS861, transposase OrfA
	SAK0504	-		IS861, transposase orfA
	SAK1549	-		IS861, transposase orfA
C1023	SAG1069	-		hypothetical protein
	GBS1101	-		hypothetical protein
C1024	SAG1070	-	V	ABC transporter, ATP-binding/permease protein
	SAK1156	-	V	ABC transporter, ATP-binding/permease protein
	GBS1102	-	V	hypothetical protein
C1025	SAG1071	-	V	ABC transporter, ATP-binding/permease protein
	SAK1157	-	V	ABC transporter, ATP-binding/permease protein
	GBS1103	-	V	hypothetical protein
C1026	SAG1073	-		hypothetical protein
	SAK1159	-		hypothetical protein
	GBS1105	-		hypothetical protein
C1027	SAG1074	-	E	serine hydroxymethyltransferase
	SAK1160	*gly*A	E	serine hydroxymethyltransferase
	GBS1106	*gly*A	E	serine hydroxymethyltransferase
C1028	SAG1075	-	J	Sua5/YciO/YrdC/YwlC family protein
	SAK1161	-	J	Sua5/YciO/YrdC/YwlC family protein
	GBS1107	-	J	hypothetical protein
C1029	SAG1076	-	J	modification methylase, HemK family
	SAK1162	-	J	modification methylase, HemK family
	GBS1108	-	J	hypothetical protein
C1030	SAG1077	*prf*A	J	peptide chain release factor 1
	SAK0668	*prf*B	J	peptide chain release factor 2
	SAK1163	*prf*A	J	peptide chain release factor 1
	GBS0564	-	J	peptide chain release factor 2
	GBS1109	-	J	peptide chain release factor 1
C1031	SAG1078	*tdk*	F	thymidine kinase
	SAK1164	*tdk*	F	thymidine kinase
	GBS1110	-	F	thymidine kinase
C1032	SAG1079	*xyl*M	R	4-oxalocrotonate tautomerase
	SAK1165	-	R	4-oxalocrotonate tautomerase
	GBS1111	-	R	4-oxalocrotonate tautomerase
C1033	SAG1080	-		hypothetical protein
C1034	SAG1081	-		ApbE family protein
	SAK1166	-		ApbE family protein
	GBS1112	-		hypothetical protein
C1035	SAG1082	-	R	hypothetical protein
	SAK1167	-	R	NADPH-dependent FMN reductase domain protein
	GBS1113	-	R	hypothetical protein

(Continue on next page)

List of homolog clusters in the 3 GBS reference genomes (Cont'd)

ID	Locus tag	Gene	COG	Annotation
C1036	SAG1083	-	R	hypothetical protein
	SAK1168	-	S	NADPH-dependent FMN reductase domain protein
	GBS1114	-	S	hypothetical protein
C1037	SAG1084	-	P	formate/nitrite transporter family protein
	SAK1169	-	P	formate/nitrite transporter family protein
	GBS1115	-	P	hypothetical protein
C1038	SAG1085	*pbu*X	F	xanthine permease
	SAK1170	*pbu*X	F	xanthine permease
	GBS1116	-	F	hypothetical protein
C1039	SAG1086	*xpt*	F	xanthine phosphoribosyltransferase
	SAK1171	*xpt*	F	xanthine phosphoribosyltransferase
	GBS1117	-	F	xanthine phosphoribosyltransferase
C1040	SAG1087	*gua*C	F	guanosine 5'-monophosphate oxidoreductase
	SAK1172	*gua*C	F	guanosine 5'-monophosphate oxidoreductase
	GBS1154	-	F	guanosine 5'-monophosphate oxidoreductase
C1041	SAG1088	-		drug resistance transporter, EmrB/QacA family, putative
	SAK1173	-		drug:H+ antiporter-2 (DHA2) family protein
	GBS1155	-		hypothetical protein
C1042	SAG1089	-		hypothetical protein
	SAK1174	-		hypothetical protein
	GBS1156	-		hypothetical protein
C1043	SAG1090	-	P	potassium uptake protein, putative
	SAK1175	-	P	K+ uptake permease (KUP) family protein
	GBS1157	-	P	hypothetical protein
C1044	SAG1091	-	R	oxidoreductase, short chain dehydrogenase/reductase family
	SAK1176	-	R	oxidoreductase, short chain dehydrogenase/reductase family
	GBS1158	-	R	hypothetical protein
C1045	SAG1092	-	C	phosphate acetyltransferase
	SAK1177	*pta*	C	phosphate acetyltransferase
	GBS1159	*pta*	C	phosphate acetyltransferase
C1046	SAG1093	-	J	ribosomal large subunit pseudouridine synthase, RluD subfamily
	SAK1178	-	J	ribosomal large subunit pseudouridine synthase, RluA family
	GBS1160	-	J	hypothetical protein
C1047	SAG1094	*ppn*K	G	inorganic polyphosphate/ATP-NAD kinase
	SAK1179	*ppn*K	G	inorganic polyphosphate/ATP-NAD kinase
	GBS1161	*ppn*K	G	inorganic polyphosphate/ATP-NAD kinase
C1048	SAG1095	-	S	GTP pyrophosphokinase family protein
	SAK1180	-	S	RelA/SpoT domain protein
	GBS1162	-	S	hypothetical protein
C1049	SAG1096	-	S	hypothetical protein
	SAK1181	-	S	adenylate cyclase, putative
	GBS1163	-	S	hypothetical protein
C1050	SAG1097	*prs*A-2	FE	ribose-phosphate pyrophosphokinase
	SAK1182	*prs*	FE	ribose-phosphate pyrophosphokinase
	GBS1164	-	FE	ribose-phosphate pyrophosphokinase
C1051	SAG1098	*isc*S-1	E	cysteine desulphurase
	SAK1183	-	E	cysteine desulfurase, putative
	GBS1165	-	E	hypothetical protein
C1052	SAG1099	-		hypothetical protein
	SAK1184	-		hypothetical protein
	GBS1166	-		hypothetical protein
C1053	SAG1100	-	R	redox-sensing transcriptional repressor Rex
	SAK1185	-	R	redox-sensing transcriptional repressor Rex
	GBS1167	-	R	redox-sensing transcriptional repressor Rex
C1054	SAG1101	*rad*C	L	DNA repair protein RadC
	SAK1186	*rad*C	L	DNA repair protein RadC
	GBS1168	*rad*C	L	DNA repair protein RadC
C1055	SAG1102	-	R	hypothetical protein
	SAK1187	-	R	hypothetical protein
	GBS1169	-	R	hypothetical protein
C1056	SAG1103	*asc*B		6-phospho-beta-glucosidase
	SAK1188	-		glycosyl hydrolase, family 1
	GBS1170	-		hypothetical protein
C1057	SAG1104	-	E	platelet activating factor, putative
	SAK1189	-	E	lipase/acylhydrolase, GDSL family
	GBS1171	-	E	hypothetical protein
C1058	SAG1105	-	R	hydrolase, haloacid dehalogenase-like family
	SAK1190	-	R	Cof-like hydrolase family protein
	GBS1172	-	R	hypothetical protein

(Continue on next page)

List of homolog clusters in the 3 GBS reference genomes (Cont'd)

ID	Locus tag	Gene	COG	Annotation
C1059	SAG1106	-		transcriptional regulator, AraC family
	SAK0531	-		transcriptional regulator, AraC family
	SAK1191	-		transcriptional regulator, AraC family
	GBS1173	-		hypothetical protein
C1060	SAG1107	-	P	voltage-gated chloride channel family protein
	SAK1192	-	P	chloride channel (ClC) family protein
	GBS1174	-	P	hypothetical protein
C1061	SAG1108	*pot*D	E	spermidine/putrescine ABC transporter, spermidine/putrescine-binding protein
	SAK1193	*pot*D	E	spermidine/putrescine ABC transporter, spermidine/putrescine-binding protein
	GBS1175	-	E	hypothetical protein
C1062	SAG1109	*pot*C	E	spermidine/putrescine ABC transporter, permease protein
	SAK1194	*pot*C	E	spermidine/putrescine ABC transporter, permease protein PotC
	GBS1176	-	E	hypothetical protein
C1063	SAG1110	*pot*B	E	spermidine/putrescine ABC transporter, permease protein
	SAK1195	*pot*B	E	spermidine/putrescine ABC transporter, permease protein PotB
	GBS1177	-	E	hypothetical protein
C1064	SAG1111	*pot*A	E	spermidine/putrescine ABC transporter, ATP-binding protein
	SAK1196	*pot*A	E	spermidine/putrescine ABC transporter, ATP-binding protein
	GBS1178	-	E	hypothetical protein
C1065	SAG1112	*mur*B	M	UDP-N-acetylenolpyruvoylglucosamine reductase
	SAK1197	*mur*B	M	UDP-N-acetylenolpyruvoylglucosamine reductase
	GBS1179	*mur*B	M	UDP-N-acetylenolpyruvoylglucosamine reductase
C1066	SAG1113	*fol*K	H	2-amino-4-hydroxy-6-hydroxymethyldihydropteridine pyrophosphokinase
	SAK1198	*fol*K	H	2-amino-4-hydroxy-6-hydroxymethyldihydropteridine pyrophosphokinase
	GBS1180	-	H	hypothetical protein
C1067	SAG1114	*fol*B	H	dihydroneopterin aldolase
	SAK1199	*fol*B	H	dihydroneopterin aldolase
	GBS1181	-	H	hypothetical protein
C1068	SAG1115	*fol*P	H	dihydropteroate synthase
	SAK1200	*fol*P	H	dihydropteroate synthase
	GBS1182	-	H	hypothetical protein
C1069	SAG1116	*fol*E	H	GTP cyclohydrolase I
	SAK1201	*fol*E	H	GTP cyclohydrolase I
	GBS1183	*fol*E	H	GTP cyclohydrolase I
C1070	SAG1117	*fol*C	H	folylpolyglutamate synthase
	SAK1202	*fol*C	H	folylpolyglutamate synthase
	GBS1184	-	H	hypothetical protein
C1071	SAG1118	*rar*D	R	rarD protein
	SAK1203	*rar*D	R	rarD protein
	GBS1185	-	R	hypothetical protein
C1072	SAG1119	*thr*B	E	homoserine kinase
	SAK1204	*thr*B	E	homoserine kinase
	GBS1186	-	E	homoserine kinase
C1073	SAG1120	*hom*	E	homoserine dehydrogenase
	SAK1205	*hom*	E	homoserine dehydrogenase
	GBS1187	-	E	hypothetical protein
C1074	SAG1121	-		polysaccharide deacetylase family protein
	SAK1206	-		polysaccharide deacetylase family protein
	GBS1188	-		hypothetical protein
C1075	SAG1122	-	M	transporter, BCCT family protein
	SAK1207	-	M	glycine betaine transporter OpuD, interruption-C
	GBS1191	-	M	hypothetical protein
C1076	SAG1123	-		hypothetical protein
C1077	SAG1124	-	C	aldehyde dehydrogenase family protein
	SAK1211	-	C	aldehyde dehydrogenase family protein
	GBS1192	-	C	hypothetical protein
C1078	SAG1125	-		hypothetical protein
	SAK1212	-		hypothetical protein
	GBS1193	-		hypothetical protein
C1079	SAG1126	-		hypothetical protein
	SAK1213	-		hypothetical protein
	GBS1194	-		hypothetical protein
C1080	SAG1127	-		hypothetical protein
	GBS1195	-		hypothetical protein
C1081	SAG1129	-		hypothetical protein
C1082	SAG1130	-		hypothetical protein
	SAK1216	-		hypothetical protein
	GBS1197	-		hypothetical protein
C1083	SAG1131	*tpx*	O	thiol peroxidase
	SAK1217	*tpx*	O	thiol peroxidase
	GBS1198	*tpx*	O	thiol peroxidase

(Continue on next page)

List of homolog clusters in the 3 GBS reference genomes (Cont'd)

ID	Locus tag	Gene	COG	Annotation
C1084	SAG1132	-	L	hypothetical protein
	SAK1218	-	L	hypothetical protein
	GBS1199	-	L	hypothetical protein
C1085	SAG1133	-	R	hypothetical protein
	GBS1200	-	R	hypothetical protein
C1086	SAG1134	-	K	transcriptional regulator, GntR family/potassioum uptake protein, TrkA family
	SAK1220	-	K	transcriptional regulator, GntR family/TrkA domain protein
	GBS1201	-	P	hypothetical protein
C1087	SAG1135	-		gls24 protein, putative
	SAK1221	-		alkaline shock protein 23, putative
	GBS1202	-		hypothetical protein
C1088	SAG1136	-		hypothetical protein
	SAK1222	-		CsbD family protein
	GBS1203	-		hypothetical protein
C1089	SAG1137	-		gls24 protein, putative
	SAK1223	-		alkaline shock protein 23, putative
	GBS1204	-		hypothetical protein
C1090	SAG1138	-		hypothetical protein
	SAK1224	-		hypothetical protein
	GBS1205	-		hypothetical protein
C1091	SAG1139	-		hypothetical protein
	SAK1225	-		hypothetical protein
	GBS1206	-		hypothetical protein
C1092	SAG1140	-	S	hypothetical protein
	SAK1226	-	S	transglycosylase associated protein
	GBS1207	-	S	hypothetical protein
C1093	SAG1141	-	S	hypothetical protein
	SAK1227	-	S	transglycosylase associated protein
	GBS1208	-	S	hypothetical protein
C1094	SAG1142	*pcr*A	L	ATP-dependent DNA helicase PcrA
	SAK1228	*pcr*A	L	ATP-dependent DNA helicase PcrA
	GBS1209	-	L	hypothetical protein
C1095	SAG1143	-	Q	hypothetical protein
	SAK1229	-	Q	thioesterase family protein
	GBS1210	-	Q	hypothetical protein
C1096	SAG1144	*ura*A	F	uracil permease
	SAK1230	*ura*A	F	uracil permease
	GBS1211	-	F	hypothetical protein
C1097	SAG1145	-	E	sodium:alanine symporter family protein
	SAK1231	-	E	amino acid carrier protein
	GBS1212	-	E	hypothetical protein
C1098	SAG1146	-	P	cation efflux family protein
	SAK1232	-	P	cation diffusion facilitator (CDF) family protein
	GBS1213	-	P	hypothetical protein
C1099	SAG1147	-	R	hypothetical protein
	SAK1233	-	R	LrgA family protein
	GBS1214	-	R	hypothetical protein
C1100	SAG1148	-		hypothetical protein
	SAK1234	-		LrgB family protein
	GBS1215	-		hypothetical protein
C1101	SAG1149	-		lipoprotein, putative
	SAK1235	-		hypothetical protein
	GBS1216	-		hypothetical protein
C1102	SAG1150	*rps*A	J	30S ribosomal protein S1
	SAK1237	*rps*A	J	30S ribosomal protein S1
	GBS1225	-	J	30S ribosomal protein S1
C1103	SAG1151	-		hypothetical protein
	SAK1240	-		hypothetical protein
	GBS1226	-		hypothetical protein
C1104	SAG1152	*ilv*E	EH	branched-chain amino acid aminotransferase
	SAK1241	*ilv*E	EH	branched-chain amino acid aminotransferase
	GBS1227	-	EH	branched-chain amino acid aminotransferase
C1105	SAG1153	*par*C	L	DNA topoisomerase IV subunit A
	SAK1242	*par*C	L	DNA topoisomerase IV subunit A
	GBS1228	-	L	DNA topoisomerase IV subunit A
C1106	SAG1154	*par*E	L	DNA topoisomerase IV subunit B
	SAK1243	*par*E	L	DNA topoisomerase IV subunit B
	GBS1229	-	L	DNA topoisomerase IV subunit B
C1107	SAG1155	-	S	hypothetical protein
	SAK1244	-	S	hypothetical protein
	GBS1230	-	S	hypothetical protein

(Continue on next page)

List of homolog clusters in the 3 GBS reference genomes (Cont'd)

ID	Locus tag	Gene	COG	Annotation
C1108	SAG1156	*ung*	L	uracil-DNA glycosylase
	SAK1245	*ung*	L	uracil-DNA glycosylase
	GBS1231	*ung*	L	uracil-DNA glycosylase
C1109	SAG1157	-		hypothetical protein
	SAK1246	-		hypothetical protein
	GBS1232	-		hypothetical protein
C1110	SAG1158	*neu*A	E	CMP-N-acetylneuraminic acid synthetase NeuA
	SAK1247	*neu*A	E	N-acylneuraminate cytidylyltransferase
	GBS1233	*neu*A	E	CMP-N-acetylneuraminic acid synthetase
C1111	SAG1159	*neu*D	R	neuD protein
	SAK1248	*neu*D	R	neuD protein
	GBS1234	*neu*D	R	hypothetical protein
C1112	SAG1160	*neu*C	M	UDP-N-acetylglucosamine-2-epimerase NeuC
	SAK1249	*neu*C	M	UDP-N-acetylglucosamine-2-epimerase NeuC
	GBS1235	*neu*C	M	hypothetical protein
C1113	SAG1161	*neu*B	M	N-acetyl neuramic acid synthetase NeuB
	SAK1250	*neu*B	M	N-acetyl neuramic acid synthetase NeuB
	GBS1236	*neu*B	M	hypothetical protein
C1114	SAG1162	*cps*L	R	polysaccharide biosynthesis protein CpsL
	SAK1251	*cps*L	R	polysaccharide biosynthesis protein CpsL
	GBS1237	*cps*M	R	capsular polysaccharide repeat unit transporter
C1115	SAG1163	*cps*K		polysaccharide biosynthesis protein CpsK(V)
	SAK1252	-		capsular polysaccharide biosynthesis protein CpsK
	GBS1237.1	*cps*L		capsular polysaccharide biosynthesis protein
C1116	SAG1164	*cps*J	M	glycosyl transferase CpsJ(V)
	SAK1253	-	M	capsular polysaccharide biosynthesis protein CpsJ
	GBS1238	CpsIaJ	M	hypothetical protein
C1117	SAG1165	*cps*O	M	glycosyl transferase CpsO(V)
	SAG1166	*cps*N	M	glycosyl transferase CpsN(V)
	SAG1455	-	M	glycosyl transferase, group 2 family protein
	SAK1254	*cps*I	M	capsular polysaccharide biosythesis protein CpsI
	SAK1488	-	M	glycosyl transferase, group 2 family protein
	GBS1239	*cps*J	M	hypothetical protein
	GBS1524	-	M	hypothetical protein
C1118	SAG1167	*cps*M	M	polysaccharide biosynthesis protein CpsM(V)
C1119	SAG1168	*cps*H		polysaccharide biosynthesis protein cpsH(V)
C1120	SAG1169	*cps*G		glycosyl transferase CpsG(V)
	SAK1256	-		polysaccharide biosynthesis protein CpsG
	GBS1241	*cps*G		beta-1,4-galactosyltransferase
C1121	SAG1170	*cps*F	M	polysaccharide biosynthesis protein CpsF
	SAK1257	*cps*F	M	polysaccharide biosynthesis protein CpsF
	GBS1242	*cps*F	M	beta-1,4-galactosyltransferase enhancer
C1122	SAG1171	*cps*E	M	glycosyl transferase CpsE
	SAK1258	*cps*E	M	glycosyl transferase CpsE
	GBS1243	*cps*E	M	hypothetical protein
C1123	SAG1172	*cps*D	D	cpsD protein
	SAK1259	*cps*D	D	tyrosine-protein kinase CpsD
	GBS1244	*cps*D	D	capsular polysaccharide chain length regulator/exporter
C1124	SAG1173	*cps*C	M	cpsC protein
	SAK1260	*cps*C	M	capsular polysaccharide biosynthesis protein cpsC
	GBS1245	*cps*C	M	hypothetical protein
C1125	SAG1174	*cps*B	GM	capsular polysaccharide biosynthesis protein CpsB
	SAK1261	*cps*B	GM	protein-tyrosine phosphatase CpsB
	GBS1246	*cps*B	GM	hypothetical protein
C1126	SAG1175	*cps*A	K	capsular polysaccharide biosynthesis protein CpsA
	SAK1262	-	K	regulatory protein CpsX
	GBS1247	*cps*A	K	hypothetical protein
C1127	SAG1176	-	K	transcriptional regulator, LysR family
	SAK1263	*cps*Y	K	transcriptional regulator CpsY
	GBS1248	*cps*Y	K	hypothetical protein
C1128	SAG1177	-		hypothetical protein
	GBS1250	-		hypothetical protein
C1129	SAG1178	*deo*D-1	F	purine nucleoside phosphorylase
	SAK1265	*deo*D	F	purine nucleoside phosphorylase
	GBS1251	-	F	hypothetical protein
C1130	SAG1179	-	P	voltage-gated chloride channel family protein
	SAK1266	-	P	chloride channel (ClC) family protein
	GBS1252	-	P	hypothetical protein
C1131	SAG1180	*deo*D-2	F	purine nucleoside phosphorylase
	SAK1267	*pun*A	F	purine nucleoside phosphorylase
	GBS1253	-	F	purine nucleoside phosphorylase

(Continue on next page)

List of homolog clusters in the 3 GBS reference genomes (Cont'd)

ID	Locus tag	Gene	COG	Annotation
C1132	SAG1181	*ars*C	P	arsenate reductase
	SAK1268	*ars*C	P	arsenate reductase
	GBS1254	-	P	hypothetical protein
C1133	SAG1182	*deo*B-1	G	phosphopentomutase
	SAG2069	*deo*B-2	G	phosphopentomutase
	SAK1269	*deo*B	G	phosphopentomutase
	SAK2008	*deo*B	G	phosphopentomutase
	GBS1255	-	G	phosphopentomutase
	GBS2023	-	G	phosphopentomutase
C1134	SAG1183	*rpi*A	G	ribose-5-phosphate isomerase A
	SAK1270	*rpi*A	G	ribose-5-phosphate isomerase A
	GBS1256	-	G	ribose-5-phosphate isomerase A
C1135	SAG1184	-	O	hypothetical protein
	SAK1271	-	O	hypothetical protein
	GBS1257	-	O	hypothetical protein
C1136	SAG1185	*est*A	R	tributyrin esterase
	SAK1272	*est*A	R	tributyrin esterase
	GBS1258	-	R	hypothetical protein
C1137	SAG1186	-	R	metallo-beta-lactamase superfamily protein
	SAK1273	-	R	metallo-beta-lactamase family protein
	GBS1259	-	R	hypothetical protein
C1138	SAG1187	-	R	ABC transporter, ATP-binding protein
	SAK1274	-	R	ABC transporter, ATP-binding protein
	GBS1260	-	R	hypothetical protein
C1139	SAG1188	-	R	ABC transporter, permease protein
	SAK1275	-	R	ABC transporter, permease protein
	GBS1261	-	R	hypothetical protein
C1140	SAG1189	-	R	hypothetical protein
	SAK1276	-	R	ABC transporter, substrate-binding protein, putative
	GBS1262	-	R	hypothetical protein
C1141	SAG1190	*pav*A	K	adherence and virulence protein A
	SAK1277	*fbp*A	K	fibronectin/fibrinogen binding protein
	GBS1263	-	K	hypothetical protein
C1142	SAG1191	*bud*A	Q	alpha-acetolactate decarboxylase
	SAK1278	*bud*A	Q	alpha-acetolactate decarboxylase
	GBS1264	-	Q	hypothetical protein
C1143	SAG1192	*ilv*K	EH	alpha-acetolactate synthase
	SAK1279	*bud*B	EH	alpha-acetolactate synthase
	GBS1265	-	EH	alpha-acetolactate synthase
C1144	SAG1193	-		TPR domain protein
	SAK1280	-		hypothetical protein
	GBS1266	-		hypothetical protein
C1145	SAG1194	-	R	hypothetical protein
	SAK1281	-	R	hypothetical protein
	GBS1267	-	R	hypothetical protein
C1146	SAG1195	-	LR	MutT/nudix family protein
	SAK1282	-	LR	hydrolase, NUDIX family
	GBS1268	-	LR	hypothetical protein
C1147	SAG1196	*mut*X	LR	mutator MutT protein
	SAK1283	*mut*X	LR	mutator mutT protein
	GBS1269	-	LR	hypothetical protein
C1148	SAG1197	-		hyaluronidase
	SAK1284	*hyl*B		hyaluronate lyase
	GBS1270	-		hyaluronate lyase
C1149	SAG1198	*rfb*B	M	dTDP-glucose 4,6-dehydratase
	SAK1285	*rfb*B	M	dTDP-glucose 4,6-dehydratase
	GBS1271	-	M	hypothetical protein
C1150	SAG1199	-	M	dTDP-4-dehydrorhamnose 3,5-epimerase
	SAK1286	*rml*C	M	dTDP-4-keto-6-deoxyglucose-3,5-epimerase
	GBS1272	*rml*C	M	dTDP-4-keto-6-deoxyglucose-3,5-epimerase
C1151	SAG1200	*rfb*A	M	glucose-1-phosphate thymidylyltransferase
	SAK1287	*rfb*A	M	glucose-1-phosphate thymidylyltransferase
	GBS1273	*rml*A	M	glucose-1-phosphate thymidyltransferase
C1152	SAG1201	-	E	iminodiacetate oxidase, putative
	SAK1288	-	E	oxidoreductase, FAD-binding
	GBS1274	-	E	hypothetical protein
C1153	SAG1202	-	S	conserved hypothetical protein TIGR00486
	SAK1289	-	S	NIF3 family protein
	GBS1275	-	S	hypothetical protein

(Continue on next page)

List of homolog clusters in the 3 GBS reference genomes (Cont'd)

ID	Locus tag	Gene	COG	Annotation
C1154	SAG1203	-	R	hypothetical protein
	SAK1290	-	R	hypothetical protein
	GBS1276	-	R	hypothetical protein
C1155	SAG1204	-	L	DNA replication protein DnaD, putative
	SAK1291	-	L	DNA replication protein DnaD, putative
	GBS1277	-	L	hypothetical protein
C1156	SAG1205	*apt*	F	adenine phosphoribosyltransferase
	SAK1292	*apt*	F	adenine phosphoribosyltransferase
	GBS1278	-	F	adenine phosphoribosyltransferase
C1157	SAG1206	-		hypothetical protein
	SAK1293	-		Clostridial hydrophobic W repeat protein
	GBS0391	-		hypothetical protein
	GBS0721	-		hypothetical protein
	GBS0988	-		hypothetical protein
	GBS1145	-		hypothetical protein
	GBS1279	-		hypothetical protein
C1158	SAG1207	-		hypothetical protein
C1159	SAG1208	*rec*J	L	single-stranded-DNA-specific exonuclease RecJ
	SAK1294	*rec*J	L	single-stranded-DNA-specific exonuclease RecJ
	GBS1280	-	L	hypothetical protein
C1160	SAG1209	-	R	oxidoreductase, short chain dehydrogenase/reductase family
	SAK1295	-	R	oxidoreductase, short-chain dehydrogenase/reductase family
	GBS1281	-	R	hypothetical protein
C1161	SAG1210	-	R	ribonuclease Z
	SAK1296	*rnz*	R	ribonuclease Z
	GBS1282	-	R	ribonuclease Z
C1162	SAG1211	-		hypothetical protein
	SAK1297	-		hypothetical protein
	GBS1283	-		hypothetical protein
C1163	SAG1212	*hfl*X	R	GTP-binding protein HflX
	SAK1298	-	R	GTP-binding protein
	GBS1284	-	R	hypothetical protein
C1164	SAG1213	*mia*A	J	tRNA delta(2)-isopentenylpyrophosphate transferase
	SAK1299	*mia*A	J	tRNA delta(2)-isopentenylpyrophosphate transferase
	GBS1285	-	J	tRNA delta(2)-isopentenylpyrophosphate transferase
C1165	SAG1214	-		hypothetical protein
C1166	SAG1215	-	P	exfoliative toxin A, putative
	SAK1301	-	P	hypothetical protein
	GBS1287	-	P	hypothetical protein
C1167	SAG1216	-	G	pullulanase, putative
	SAK1302	-	G	pullulanase, extracellular
	GBS1288	-	G	hypothetical protein
C1168	SAG1218	-	L	hypothetical protein
	SAK1304	-	L	uracil DNA glycosylase family protein
	GBS1290	-	L	hypothetical protein
C1169	SAG1219	-	E	dipeptidase
	SAK1305	*pep*V	E	dipeptidase
	GBS1291	-	E	dipeptidase
C1170	SAG1220	-	C	nitroreductase family protein
	SAK1306	-	C	nitroreductase family protein
	GBS1292	-	C	hypothetical protein
C1171	SAG1222	*uvr*C	L	excinuclease ABC subunit C
	SAK1308	*uvr*C	L	excinuclease ABC subunit C
	GBS1294	*uvr*C	L	excinuclease ABC subunit C
C1172	SAG1223	-	S	hypothetical protein
	SAK1309	-	S	hypothetical protein
	GBS1295	-	S	hypothetical protein
C1173	SAG1224	-	V	MATE efflux family protein
	SAK1310	-	V	MATE efflux family protein
	GBS1296	-	V	hypothetical protein
C1174	SAG1225	-	S	hypothetical protein
	SAK1311	-	S	glyoxalase family protein
	GBS1297	-	S	hypothetical protein
C1175	SAG1226	-		hypothetical protein
	SAK1312	-		hypothetical protein
	GBS1298	-		hypothetical protein
C1176	SAG1227	-		hypothetical protein
	SAK1313	-		hypothetical protein
	GBS1299	-		hypothetical protein

(Continue on next page)

List of homolog clusters in the 3 GBS reference genomes (Cont'd)

ID	Locus tag	Gene	COG	Annotation
C1177	SAG1228	-	L	ISSdy1, transposase OrfA
	SAG1243	-	L	ISSdy1, transposase OrfA
	SAK1314	-	L	ISSag4, transposase orfA
	SAK1322	-	L	ISSag4, transposase orfA
	GBS1300	-	L	hypothetical protein
	GBS1310	-	L	hypothetical protein
C1178	SAG1230	-		hypothetical protein
	SAK1316	-		ISSag7, transposase orfA
	GBS1302	-		hypothetical protein
C1179	SAG1233	-		streptococcal histidine triad family protein
	SAK1318	-		streptococcal histidine triad family protein
	SAK1897	-		streptococcal histidine triad family protein
	GBS1306	-		hypothetical protein
	GBS1925	-		hypothetical protein
C1180	SAG1234	*lmb*	P	laminin-binding surface protein
	SAK1319	*lmb*	P	laminin-binding surface protein
	GBS1307	*lmb*	P	laminin-binding surface protein
C1181	SAG1235	-	L	GBSi1, group II intron, maturase
C1182	SAG1237	-		hypothetical protein
	SAK1321	-		hypothetical protein
	GBS0410	-		hypothetical protein
	GBS0740	-		hypothetical protein
	GBS0969	-		hypothetical protein
	GBS1118	-		hypothetical protein
C1183	SAG1238	-		hypothetical protein
C1184	SAG1239	-		hypothetical protein
C1185	SAG1245	-		hypothetical protein
C1186	SAG1246	-		hypothetical protein
	SAK1325	-		hypothetical protein
	GBS1313	-		hypothetical protein
C1187	SAG1247	-	L	site-specific recombinase, phage integrase family
	SAK1326	-	L	site-specific recombinase, phage integrase family
	GBS1314	-	L	hypothetical protein
	GBS1325	-	L	hypothetical protein
C1188	SAG1248	-		hypothetical protein
	GBS1315	-		hypothetical protein
	GBS1326	-		hypothetical protein
C1189	SAG1249	-	K	transcriptional regulator, Cro/CI family
	GBS1327	-	K	hypothetical protein
C1190	SAG1250	-	U	Tn5252, relaxase
	GBS1121	-	U	hypothetical protein
	GBS1338	-	U	hypothetical protein
C1191	SAG1251	-		Tn5252, Orf 9 protein
	GBS1339	-		hypothetical protein
C1192	SAG1252	-		Tn5252, Orf 10 protein
	GBS0622	-		hypothetical protein
	GBS1340	-		hypothetical protein
C1193	SAG1253	-	L	transposase, ISL3 family
C1194	SAG1258	*cad*C	K	cadmium efflux system accessory protein
	SAK2052	*cad*X	K	cadmium resistance accessory protein CadX
	GBS2065	-	K	hypothetical protein
C1195	SAG1259	-		hypothetical protein
C1196	SAG1260	-		hypothetical protein
C1197	SAG1265	-		cadmium resistance transporter, putative
	SAK2051	*cad*D		cadmium resistance protein CadD
	GBS2064	-		hypothetical protein
C1198	SAG1266	-		hypothetical protein
C1199	SAG1267	-		hypothetical protein
C1200	SAG1269	-		hypothetical protein
C1201	SAG1270	-	L	ImpB/MucB/SamB family protein
	SAG1726	*din*P	L	DNA polymerase IV
	SAK1734	*din*B	L	DNA polymerase IV
	GBS1771	-	L	DNA polymerase IV
C1202	SAG1271	-		hypothetical protein
C1203	SAG1272	-		hypothetical protein
C1204	SAG1273	-		hypothetical protein
	GBS1345	-		hypothetical protein
C1205	SAG1274	-		hypothetical protein
	GBS1346	-		hypothetical protein
C1206	SAG1275	-		hypothetical protein
	GBS1347	-		hypothetical protein

(Continue on next page)

List of homolog clusters in the 3 GBS reference genomes (Cont'd)

ID	Locus tag	Gene	COG	Annotation
C1207	SAG1276	-		hypothetical protein
	GBS0386	-		hypothetical protein
	GBS0716	-		hypothetical protein
	GBS0993	-		hypothetical protein
	GBS1126	-		hypothetical protein
	GBS1348	-		hypothetical protein
C1208	SAG1277	-		hypothetical protein
	GBS1132	-		hypothetical protein
	GBS1349	-		hypothetical protein
C1209	SAG1278	-		hypothetical protein
	GBS1350	-		hypothetical protein
C1210	SAG1279	-		hypothetical protein
	GBS1351	-		hypothetical protein
C1211	SAG1280	-	KL	SNF2 family protein
	SAG1618	-	KL	Snf2 family protein
	SAK1633	-	KL	Snf2 family protein
	GBS1352	-	KL	hypothetical protein
	GBS1353	-		hypothetical protein
	GBS1666	-	KL	hypothetical protein
C1212	SAG1281	-		hypothetical protein
	GBS1354	-		hypothetical protein
C1213	SAG1282	-		calcium-binding protein, putative
	GBS1355	-		hypothetical protein
C1214	SAG1283	*ssp*		agglutinin receptor
	GBS1356	-		hypothetical protein
C1215	SAG1284	*abi*GI	K	abortive infection protein AbiGI
	GBS1357	-	K	hypothetical protein
C1216	SAG1285	*abi*GII	S	abortive infection protein AbiGII
	GBS1358	-	S	hypothetical protein
C1217	SAG1286	-	R	Tn5252, Orf28
	SAG1683	-	R	immunogenic secreted protein, putative
	SAK1695	-	R	immunogenic secreted protein, putative
	GBS1133	-	R	hypothetical protein
	GBS1359	-	R	hypothetical protein
	GBS1727	-	R	hypothetical protein
C1218	SAG1287	-	U	Tn5252, Orf26
	GBS1135	-	U	hypothetical protein
	GBS1360	-	U	hypothetical protein
C1219	SAG1289	-		Tn5252, Orf23
	GBS1362	-		hypothetical protein
C1220	SAG1290	-		hypothetical protein
	GBS1363	-		hypothetical protein
C1221	SAG1292	-		hypothetical protein
	GBS1365	-		hypothetical protein
C1222	SAG1294	-		hypothetical protein
	GBS1367	-		hypothetical protein
C1223	SAG1296	-		hypothetical protein
	GBS1369	-		hypothetical protein
C1224	SAG1297	-	L	C-5 cytosine-specific DNA methylase
	SAG1869	-	L	prophage LambdaSa2, type II DNA modification methyltransferase, putative
	SAK0739	-	L	prophage LambdaSa04, methyltransferase, C-5 cytosine-specific family
	GBS1370	-	L	hypothetical protein
C1225	SAG1298	-		hypothetical protein
C1226	SAG1300	-		hypothetical protein
	GBS1373	-		hypothetical protein
C1227	SAG1301	*rpl*L	J	50S ribosomal protein L7/L12
	SAK1334	*rpl*L	J	50S ribosomal protein L7/L12
	GBS1374	*rpl*L	J	50S ribosomal protein L7/L12
C1228	SAG1302	*rpl*J	J	50S ribosomal protein L10
	SAK1335	*rpl*J	J	50S ribosomal protein L10
	GBS1375	*rpl*J	J	50S ribosomal protein L10
C1229	SAG1303	-	O	ATP-dependent Clp protease, ATP-binding subunit
	SAK1336	*clp*L	O	ATP-dependent Clp protease, ATP-binding subunit ClpL
	GBS1376	-	O	hypothetical protein
C1230	SAG1304	-		hypothetical protein
C1231	SAG1305	*mmu*M	E	homocysteine methyltransferase
	SAK1337	*mmu*M	E	homocysteine methyltransferase
	GBS1377	*mmu*M	E	homocysteine methyltransferase
C1232	SAG1306	-	E	amino acid permease
	SAK1338	-	E	amino acid (AAT) family permease protein
	GBS1378	-	E	hypothetical protein

(Continue on next page)

List of homolog clusters in the 3 GBS reference genomes (Cont'd)

ID	Locus tag	Gene	COG	Annotation
C1233	SAG1307	-		hypothetical protein
	SAK1339	-		hypothetical protein
	GBS1379	-		hypothetical protein
C1234	SAG1308	-		hypothetical protein
	SAK1340	-		hypothetical protein
	GBS1380	-		hypothetical protein
C1235	SAG1309	-		hypothetical protein
C1236	SAG1310	-	K	transcriptional regulator, TetR family
	SAK1341	-	K	transcriptional regulator, TetR family
	GBS1381	-	K	hypothetical protein
C1237	SAG1311	-	R	GTP-binding protein
	SAK1342	-	R	GTP-binding protein
	GBS1382	-	R	GTP-binding protein
C1238	SAG1312	*clp*X	O	ATP-dependent protease ATP-binding subunit
	SAK1343	*clp*X	O	ATP-dependent protease ATP-binding subunit
	GBS1383	-	O	ATP-dependent protease ATP-binding subunit
C1239	SAG1313	-		hypothetical protein
C1240	SAG1314	*fol*A	H	dihydrofolate reductase
	SAK1345	*fol*A	H	dihydrofolate reductase
	GBS1384	*dfr*A	H	hypothetical protein
C1241	SAG1315	*thy*A		thymidylate synthase
	SAK1346	*thy*A		thymidylate synthase
	GBS1385	ThyA		thymidylate synthase
C1242	SAG1316	-	I	HMG-CoA synthase
	SAK1347	-	I	hydroxymethylglutaryl-CoA synthase
	GBS1386	-	I	hypothetical protein
C1243	SAG1317	-	I	3-hydroxy-3-methylglutaryl-CoA reductase
	SAK1348	-	I	hydroxymethylglutaryl-CoA reductase, degradative
	GBS1387	-	I	hypothetical protein
C1244	SAG1318	-		hypothetical protein
	SAK1349	-		hypothetical protein
	GBS1388	-		hypothetical protein
C1245	SAG1319	-	R	hemolysin III, putative
	SAK1350	-	R	hemolysin III
	GBS1389	-	R	hypothetical protein
C1246	SAG1320	-	IR	conserved hypothetical protein TIGR00147
	SAK1351	-	IR	conserved hypothetical protein TIGR00147
	GBS1390	-	IR	hypothetical protein
C1247	SAG1321	-	O	hypothetical protein
	SAK1352	-	O	hypothetical protein
	GBS1391	-	O	hypothetical protein
C1248	SAG1322	-		hypothetical protein
	SAK1353	-		hypothetical protein
	GBS1392	-		hypothetical protein
C1249	SAG1323	-	C	isopentenyl pyrophosphate isomerase
	SAK1354	*fni*	C	isopentenyl pyrophosphate isomerase
	GBS1393	-	C	isopentenyl pyrophosphate isomerase
C1250	SAG1324	-	I	phosphomevalonate kinase
	SAK1355	-	I	phosphomevalonate kinase
	GBS1394	-	I	hypothetical protein
C1251	SAG1325	*mva*D	I	diphosphomevalonate decarboxylase
	SAK1356	*mva*D	I	diphosphomevalonate decarboxylase
	GBS1395	-	I	hypothetical protein
C1252	SAG1326	-	I	mevalonate kinase, putative
	SAK1357	*mvk*	I	mevalonate kinase
	GBS1396	-	I	hypothetical protein
C1253	SAG1327	-	T	sensor histidine kinase
	SAK1358	-	T	sensor histidine kinase
	GBS1397	-	T	hypothetical protein
C1254	SAG1328	-	TK	DNA-binding response regulator
	SAK1359	-	TK	DNA-binding response regulator
	GBS1398	-	TK	hypothetical protein
C1255	SAG1329	-	S	GTP pyrophosphokinase family protein
	SAK1360	-	S	RelA/SpoT domain protein
	GBS1399	-	S	hypothetical protein
C1256	SAG1330	-		hypothetical protein
C1257	SAG1331	-		R5 protein
C1258	SAG1332	-	K	transcriptional regulator, MarR family
	SAK1363	-	K	transcriptional regulator, MarR family
	GBS1402	-	K	hypothetical protein

(Continue on next page)

List of homolog clusters in the 3 GBS reference genomes (Cont'd)

ID	Locus tag	Gene	COG	Annotation
C1259	SAG1333	-	F	5'-nucleotidase family protein
	SAK1364	-	F	Ser/Thr protein phosphatase family protein
	GBS1403	-	F	hypothetical protein
C1260	SAG1334	-		peptide deformylase
	SAK1365	*def*		peptide deformylase
	GBS1404	-		peptide deformylase
C1261	SAG1335	*gdh*A	E	glutamate dehydrogenase
	SAK1366	*gdh*A	E	glutamate dehydrogenase
	GBS1405	-	E	glutamate dehydrogenase
C1262	SAG1336	-	S	hypothetical protein
	SAK1367	-	S	hypothetical protein
	GBS1406	-	S	hypothetical protein
C1263	SAG1337	-	V	ABC transporter, ATP-binding/permease protein
	SAK1361	-	V	efflux ABC transporter, permease/ATP-binding protein
	SAK1368	-	V	ABC transporter, ATP-binding/permease protein
	GBS1400	-	V	hypothetical protein
	GBS1407	-	V	hypothetical protein
C1264	SAG1338	-	V	ABC transporter, ATP-binding/permease protein
	SAK1362	-	V	efflux ABC transporter, permease/ATP-binding protein
	SAK1369	-	V	ABC transporter, ATP-binding/permease protein
	GBS1401	-	V	hypothetical protein
	GBS1408	-	V	hypothetical protein
C1265	SAG1339	-		acetyltransferase, GNAT family
	SAK1370	-		acetyltransferase, GNAT family
	GBS1409	-		hypothetical protein
C1266	SAG1340	-	R	ABC transporter, ATP-binding protein
	SAK1371	-	R	ABC transporter, ATP-binding protein
	GBS1410	-	R	hypothetical protein
C1267	SAG1341	-	J	polyA polymerase family protein
	SAK1372	*pap*S	J	polyA polymerase
	GBS1411	-	J	hypothetical protein
C1268	SAG1342	-	S	DegV family protein
	SAK1373	-	S	DegV family protein
	GBS1412	-	S	hypothetical protein
C1269	SAG1343	-		hypothetical protein
	SAK1374	-		hypothetical protein
	GBS1413	-		hypothetical protein
C1270	SAG1344	-		hypothetical protein
	SAK1375	-		hypothetical protein
	GBS1414	-		hypothetical protein
C1271	SAG1345	-	S	hypothetical protein
	SAK1376	-	S	hypothetical protein
	GBS1415	-	S	hypothetical protein
C1272	SAG1346	-	GT	PTS system, fructose specific IIABC components
	SAK1377	-	GT	PTS system, fructose-specific IIABC component
	SAK1759	-	G	PTS system, fructose-specific, IIC component
	SAK1760	-	GT	PTS system, fructose-specific, IIA component
	SAK1761	-		PTS system, fructose-specific, IIB component
	GBS1416	-	G	hypothetical protein
C1273	SAG1347	*fru*K	G	1-phosphofructokinase
	SAK1378	-	G	1-phosphofructokinase, putative
	GBS1417	-	G	hypothetical protein
C1274	SAG1348	*lac*R-1	KG	lactose phosphotransferase system repressor
	SAK1379	-	KG	transcriptional regulator, DeoR family
	GBS1418	-	KG	hypothetical protein
C1275	SAG1349	-	V	beta-lactam resistance factor
	SAK1380	-	V	beta-lactam resistance factor
	GBS1419	-	V	hypothetical protein
C1276	SAG1350	-		surface antigen-related protein
	SAK1381	-		bsp protein
	GBS1420	-		hypothetical protein
C1277	SAG1351	-	H	2-dehydropantoate 2-reductase
	SAK1382	*pan*E	H	2-dehydropantoate 2-reductase
	GBS1421	-	H	2-dehydropantoate 2-reductase
C1278	SAG1352	-	R	regulatory protein, putative
	SAK1383	-	R	hypothetical protein
	GBS1422	-	R	hypothetical protein
C1279	SAG1353	-	O	pyridine nucleotide-disulphide oxidoreductase family protein
	SAK1384	-	O	pyridine nucleotide-disulphide oxidoreductase family protein
	GBS1423	-	O	hypothetical protein

(Continue on next page)

List of homolog clusters in the 3 GBS reference genomes (Cont'd)

ID	Locus tag	Gene	COG	Annotation
C1280	SAG1354	*trm*D		tRNA (guanine-N(1)-)-methyltransferase
	SAK1385	*trm*D		tRNA (guanine-N(1)-)-methyltransferase
	GBS1424	*trm*D		tRNA (guanine-N(1)-)-methyltransferase
C1281	SAG1355	*rim*M	J	16S rRNA-processing protein
	SAK1386	*rim*M	J	16S rRNA-processing protein
	GBS1425	-	J	16S rRNA-processing protein
C1282	SAG1356	-		transcriptional regulator, RofA family
	SAK1389	-		transcriptional regulator, RofA family
	GBS1426	-		hypothetical protein
	GBS1479	-		hypothetical protein
	GBS1530	-		hypothetical protein
C1283	SAG1357	-	R	KH domain protein
	SAK1390	-	R	hypothetical protein
	GBS1427	-	R	hypothetical protein
C1284	SAG1358	*rps*P		30S ribosomal protein S16
	SAK1391	*rps*P		30S ribosomal protein S16
	GBS1428	*rps*P		30S ribosomal protein S16
C1285	SAG1359	-	V	permease, putative
	SAK1392	-	V	ABC transporter, permease protein, putative
	GBS1429	-	V	hypothetical protein
C1286	SAG1360	-	V	ABC transporter, ATP-binding protein
	SAK1393	-	V	ABC transporter, ATP-binding protein
	GBS1430	-	V	hypothetical protein
C1287	SAG1361	-	M	hypothetical protein
	SAK1394	-	M	efflux transporter, RND family, MFP subunit
	GBS1431	-	M	hypothetical protein
C1288	SAG1362	-	EF	carbamoyl-phosphate synthase, large subunit, putative
	SAK1395	-	EF	carbamoyl-phosphate synthase, large subunit, putative
	GBS1432	-	EF	hypothetical protein
C1289	SAG1363	*car*A-2	EF	carbamoyl-phosphate synthase small subunit
	SAK1396	*car*A	EF	carbamoyl-phosphate synthase small subunit
	GBS1433	-	EF	carbamoyl-phosphate synthase small subunit
C1290	SAG1364	*pyr*R	F	pyrimidine regulatory protein PyrR
	SAK1397	*pyr*R	F	pyrimidine regulatory protein PyrR
	GBS1434	-	F	pyrimidine regulatory protein PyrR
C1291	SAG1365	-	J	ribosomal large subunit pseudouridine synthase, RluD subfamily
	SAK1398	-	J	ribosomal large subunit pseudouridine synthase, RluA family
	GBS1435	-	J	hypothetical protein
C1292	SAG1366	*lsp*A	MU	lipoprotein signal peptidase
	SAK1399	*lsp*A	MU	signal peptidase II
	GBS1436	-	MU	hypothetical protein
C1293	SAG1367	-	K	transcriptional regulator, LysR family
	SAK1400	-	K	transcriptional regulator, LysR family
	GBS1437	-	K	hypothetical protein
C1294	SAG1368	*rpm*A		50S ribosomal protein L27
	SAK1401	*rpm*A		50S ribosomal protein L27
	GBS1438	-		50S ribosomal protein L27
C1295	SAG1369	-	J	hypothetical protein
	SAK1402	-	J	hypothetical protein
	GBS1439	-	J	hypothetical protein
C1296	SAG1370	*rpl*U		50S ribosomal protein L21
	SAK1403	*rpl*U		50S ribosomal protein L21
	GBS1440	*rpl*21		50S ribosomal protein L21
C1297	SAG1371	-	M	hypothetical protein
	SAK1404	-	M	capA domain protein
	GBS1441	-	M	hypothetical protein
C1298	SAG1372	*thi*I	H	thiamine biosynthesis protein ThiI
	SAK1405	*thi*I	H	thiamine biosynthesis protein ThiI
	GBS1442	-	H	thiamine biosynthesis protein ThiI
C1299	SAG1373	*isc*S-2	E	cysteine desulphurase
	SAK1406	-	E	aminotransferase, class V
	GBS1443	-	E	hypothetical protein
C1300	SAG1374	-		hypothetical protein
	SAK1407	-		hypothetical protein
	GBS1444	-		hypothetical protein
C1301	SAG1375	*gor*	C	glutathione reductase
	SAK1408	*gor*	C	glutathione reductase
	GBS1445	-	C	glutathione reductase
C1302	SAG1376	-	S	hypothetical protein
	SAK1409	-	S	hypothetical protein
	GBS1446	-	S	hypothetical protein

(Continue on next page)

List of homolog clusters in the 3 GBS reference genomes (Cont'd)

ID	Locus tag	Gene	COG	Annotation
	SAG1377	*aro*C		chorismate synthase
C1303	SAK1410	*aro*C		chorismate synthase
	GBS1447	*aro*C		chorismate synthase
	SAG1378	*aro*B	E	3-dehydroquinate synthase
C1304	SAK1411	*aro*B	E	3-dehydroquinate synthase
	GBS1448	*aro*B	E	3-dehydroquinate synthase
	SAG1379	*aro*D		3-dehydroquinate dehydratase
C1305	SAK1412	*aro*D		3-dehydroquinate dehydratase
	GBS1449	*aro*D		3-dehydroquinate dehydratase
	SAG1380	-	R	hypothetical protein
C1306	SAK1413	-	R	hypothetical protein
	GBS1450	-	R	hypothetical protein
	SAG1381	-	M	sulfatase
C1307	SAK1414	-	M	sulfatase family protein
	GBS1451	-	M	hypothetical protein
	SAG1382	*rpl*T		50S ribosomal protein L20
C1308	SAK1415	*rpl*T		50S ribosomal protein L20
	GBS1452	*rpl*T		50S ribosomal protein L20
	SAG1383	*rpm*I	J	50S ribosomal protein L35
C1309	SAK1416	*rpm*I	J	50S ribosomal protein L35
	GBS1453	*rpm*I	J	50S ribosomal protein L35
	SAG1384	*inf*C	J	translation initiation factor IF-3
C1310	SAK1417	*inf*C	J	translation initiation factor IF-3
	GBS1454	*inf*C	J	translation initiation factor IF-3
	SAG1385	*cmk*	F	cytidylate kinase
C1311	SAK1418	*cmk*	F	cytidylate kinase
	GBS1455	*cmk*	F	cytidylate kinase
	SAG1386	-		hypothetical protein
C1312	SAK1419	-		hypothetical protein
	GBS1456	-		hypothetical protein
	SAG1387	-	C	ferredoxin, 4Fe-4S
C1313	SAK1420	-	C	ferredoxin, putative
	GBS1457	-	C	hypothetical protein
	SAG1388	-		hypothetical protein
C1314	SAK1421	-		hypothetical protein
	GBS1458	-		hypothetical protein
	SAG1389	*pep*T	E	peptidase T
C1315	SAK1422	*pep*T	E	peptidase T
	GBS1459	-	E	peptidase T
	SAG1390	-	R	polysaccharide biosynthesis protein, putative
C1316	SAK1423	-	R	polysaccharide biosynthesis protein
	GBS1460	-	R	hypothetical protein
	SAG1391	*mur*E	M	UDP-N-acetylmuramoylalanyl-D-glutamate–2,6-diaminopimelate ligase
C1317	SAK1424	*mur*E	M	UDP-N-acetylmuramoylalanyl-D-glutamate–2,6-diaminopimelate ligase
	GBS1461	*mur*E	M	UDP-N-acetylmuramoylalanyl-D-glutamate–2,6-diaminopimelate ligase
	SAG1392	-	PH	iron compound ABC transporter, ATP-binding protein
C1318	SAK1425	*fhu*C	PH	ferrichrome ABC transporter, ATP-binding protein
	GBS1462	-	PH	hypothetical protein
	SAG1393	-	P	iron compound ABC transporter, substrate-binding protein
C1319	SAK1426	*fhu*D	P	ferrichrome ABC transporter, ferrichrome-binding protein
	GBS1463	-	P	hypothetical protein
	SAG1394	-	P	iron compound ABC transporter, permease protein
C1320	SAK1427	*fhu*B	P	ferrichrome ABC transporter, permease protein
	GBS1464	-	P	hypothetical protein
	SAG1395	-	P	iron compound ABC transporter, permease protein
C1321	SAK1428	-	P	iron compound ABC transporter, permease protein
	GBS1465	-	P	hypothetical protein
	SAG1396	-		hypothetical protein
C1322	SAK1429	-		hypothetical protein
	GBS1466	-		hypothetical protein
	SAG1397	*ppa*	C	putative manganese-dependent inorganic pyrophosphatase
C1323	SAK1430	*ppa*C	C	putative manganese-dependent inorganic pyrophosphatase
	GBS1467	-	C	putative manganese-dependent inorganic pyrophosphatase
	SAG1398	*pfl*A-2	O	pyruvate formate-lyase-activating enzyme
C1324	SAK1431	*pfl*A	O	pyruvate formate-lyase-activating enzyme
	GBS1468	-	O	hypothetical protein
	SAG1399	-	R	CBS domain protein
C1325	SAK1432	-	R	CBS domain protein
	GBS1469	-	R	hypothetical protein

(Continue on next page)

List of homolog clusters in the 3 GBS reference genomes (Cont'd)

ID	Locus tag	Gene	COG	Annotation
C1326	SAG1400	-		hypothetical protein
	SAK1433	-		hypothetical protein
	GBS1470	-		hypothetical protein
C1327	SAG1401	-	R	conserved hypothetical protein TIGR01212
	SAK1434	-	R	radical SAM protein, TIGR01212 family
	GBS1471	-	R	hypothetical protein
C1328	SAG1402	-	I	PAP2 family protein
	SAK1435	-	I	PAP2 family protein
	GBS1472	-	I	hypothetical protein
C1329	SAG1403	-	S	hypothetical protein
	SAK1436	-	S	hypothetical protein
	GBS1473	-	S	hypothetical protein
C1330	SAG1410	-	M	glycosyl transferase, group 1 family protein
	SAK1445	-	M	glycosyl transferase, group 1 family protein
	GBS1480	-	M	hypothetical protein
C1331	SAG1411	-	R	glycosyl transferase, group 2 family protein
	SAK1446	-	R	glycosyl transferase, group 2 family protein
	GBS1481	-	R	hypothetical protein
C1332	SAG1412	-	R	polysaccharide biosynthesis protein
	SAK1447	-	R	polysaccharide biosynthesis protein
	GBS1482	-	R	hypothetical protein
C1333	SAG1413	-		hypothetical protein
	SAK1448	-		hypothetical protein
	GBS1483	-		hypothetical protein
C1334	SAG1414	-	M	glycosyl transferase, group 2 family protein
	SAK1449	-	M	glycosyl transferase, group 2 family protein
	GBS1484	-	M	hypothetical protein
C1335	SAG1415	-	M	glycosyl transferase, group 2 family protein
	SAK1450	-	M	glycosyl transferase, group 2 family protein
	GBS1485	-	M	hypothetical protein
C1336	SAG1416	-	MG	nucleotide sugar dehydratase, putative
	SAK1451	-	MG	nucleotide sugar dehydratase, putative
	GBS1486	-	MG	hypothetical protein
C1337	SAG1417	-	I	nucleotidyl transferase, putative
	SAK1452	*isp*D	I	2-C-methyl-D-erythritol 4-phosphate cytidylyltransferase
	GBS1487	-	I	hypothetical protein
C1338	SAG1418	-	M	polysaccharide biosynthesis protein, putative
	SAK1453	*lic*D2	M	licD2 protein
	GBS1488	-	M	hypothetical protein
C1339	SAG1419	-		lipoprotein, putative
	SAK1454	-		hypothetical protein
	GBS1489	-		hypothetical protein
C1340	SAG1420	-	S	hypothetical protein
	SAK1455	-	S	hypothetical protein
	GBS1490	-	S	hypothetical protein
C1341	SAG1421	-	M	glycosyl transferase, group 2 family protein
	SAK1456	-	M	glycosyl transferase, group 2 family protein
	GBS1491	-	M	hypothetical protein
C1342	SAG1422	-	M	glycosyl transferase, group 2 family protein
	SAK1457	-	M	glycosyl transferase, group 2 family protein
	GBS1492	-	M	hypothetical protein
C1343	SAG1423	-	M	glycosyl transferase, putative
	SAK1458	*rgp*Ac	M	rgpAc protein
	GBS1493	-	M	hypothetical protein
C1344	SAG1424	*rfb*D	M	dTDP-4-dehydrorhamnose reductase
	SAK1459	*rfb*D	M	dTDP-4-dehydrorhamnose reductase
	GBS1494	*rml*D	M	dTDP-L-rhamnose synthase
C1345	SAG1425	-		hypothetical protein
	SAK1460	-		hypothetical protein
	GBS1495	-		hypothetical protein
C1346	SAG1426	*rpo*D	K	RNA polymerase sigma factor
	SAK1461	*rpo*D	K	RNA polymerase sigma factor
	GBS1496	*rpo*D	K	RNA polymerase sigma factor
C1347	SAG1427	*dna*G	L	DNA primase
	SAK1462	*dna*G	L	DNA primase
	GBS1497	*dna*G	L	DNA primase
C1348	SAG1428	*msc*L		large conductance mechanosensitive channel protein
	SAK1463	*msc*L		large conductance mechanosensitive channel protein
	GBS1498	*msc*L		large conductance mechanosensitive channel protein MscL

(Continue on next page)

List of homolog clusters in the 3 GBS reference genomes (Cont'd)

ID	Locus tag	Gene	COG	Annotation
C1349	SAG1429	*rps*U	J	ribosomal protein S21
	SAK1464	*rps*U	J	ribosomal protein S21
	GBS1499	*rps*U	J	ribosomal protein S21
C1350	SAG1430	-	R	hypothetical protein
	SAK1465	-	R	hypothetical protein
	GBS1500	-	R	hypothetical protein
C1351	SAG1431	-	ET	amino acid ABC transporter, amino acid-binding protein
	SAK1466	-	ET	polar amino acid uptake (PAAT) family ABC transporter, amino acid-binding protein
	GBS1501	-	ET	hypothetical protein
C1352	SAG1432	-		ammonium transporter family protein
	SAK1467	-		ammonium transporter (Amt) family protein
	GBS1502	-		hypothetical protein
C1353	SAG1433	-	O	hypothetical protein
	SAK1468	-	O	hypothetical protein
	GBS1503	-	O	hypothetical protein
C1354	SAG1434	-	R	hypothetical protein
	SAK1469	-	R	hypothetical protein
	GBS1504	-	R	hypothetical protein
C1355	SAG1435	-		hypothetical protein
	SAK1470	-		hypothetical protein
	GBS1505	-		hypothetical protein
C1356	SAG1436	-	G	glycerol-3-phosphate transporter, putative
	SAK1471	-	G	glycerol-3-phosphate transporter, putative
	GBS1506	-	G	hypothetical protein
C1357	SAG1437	-		hypothetical protein
C1358	SAG1438	*glg*P	G	glycogen phosphorylase
	SAK1472	*glg*P	G	glycogen/starch/alpha-glucan phosphorylase
	GBS1507	-	G	hypothetical protein
C1359	SAG1439	*mal*Q		4-alpha-glucanotransferase
	SAK1473	*mal*Q		4-alpha-glucanotransferase
	GBS1508	-		hypothetical protein
C1360	SAG1440	-	K	maltose operon repressor MalR, putative
	SAK1474	-	K	maltose operon transcriptional repressor MalR, putative
	GBS1509	-	K	hypothetical protein
C1361	SAG1441	-	G	maltose/maltodextrin ABC transporter, maltose/maltodextrin-binding protein
	SAK1475	-	G	carbohydrate uptake 1 (CUT1) family, carbohydrate-binding protein
	GBS1510	-	G	hypothetical protein
C1362	SAG1442	-	G	maltose ABC transporter, permease protein
	SAK1476	-	G	cyclodextrin ABC transporter, permease protein
	GBS1511	-	G	hypothetical protein
C1363	SAG1443	-	G	maltose ABC transporter, permease protein
	SAK1477	-	G	cyclodextrin ABC transporter, permease protein
	GBS1512	-	G	hypothetical protein
C1364	SAG1444	-	E	proton/peptide symporter family protein
	SAK1478	-	E	amino acid/peptide transporter
	GBS1513	-	E	hypothetical protein
C1365	SAG1446	-		hypothetical protein
	GBS1515	-		hypothetical protein
C1366	SAG1447	-		hypothetical protein
	SAK1480	-		hypothetical protein
	GBS1516	-		hypothetical protein
C1367	SAG1448	-	M	glycosyl transferase, group 1 family protein
	SAK1481	-	M	glycosyl transferase, group 1 family protein
	GBS1517	-	M	hypothetical protein
C1368	SAG1449	-	U	translocase
	SAK1482	*sec*A	U	translocase
	GBS1518	-	U	translocase
C1369	SAG1450	-		hypothetical protein
	SAK1483	*asp*3		accessory secretory protein Asp3
	GBS1519	-		hypothetical protein
C1370	SAG1451	-		hypothetical protein
	SAK1484	*asp*2		accessory secretory protein Asp2
	GBS1520	-		hypothetical protein
C1371	SAG1452	-		hypothetical protein
	SAK1485	*asp*1		accessory secretory protein Asp1
	GBS1521	-		hypothetical protein
C1372	SAG1453	-	U	preprotein translocase SecY family protein
	SAK1486	-	U	preprotein translocase, SecY subunit, putative
	GBS1522	-	U	hypothetical protein

(Continue on next page)

List of homolog clusters in the 3 GBS reference genomes (Cont'd)

ID	Locus tag	Gene	COG	Annotation
C1373	SAG1454	-		glycosyl transferase, putative
	SAK1487	-		hypothetical protein
	GBS1523	-		hypothetical protein
C1374	SAG1459	-	M	glycosyl transferase, family 8
	SAK1490	-	M	glycosyl transferase, family 8
	GBS1526	-	M	hypothetical protein
C1375	SAG1460	-	M	glycosyl transferase, family 8
	SAG2061	-	M	glycosyl transferase, family 8
	SAK1491	-	M	glycosyl transferase, family 8
	GBS1527	-	M	hypothetical protein
	GBS2016	-	M	hypothetical protein
C1376	SAG1461	-		hypothetical protein
	SAK1492	-		hypothetical protein
	GBS1528	-		hypothetical protein
C1377	SAG1462	-		cell wall surface anchor family protein
	SAK1493	-		cell wall surface anchor family protein
	GBS1529	-		hypothetical protein
C1378	SAG1464	*uvr*B	L	excinuclease ABC subunit B
	SAK1495	*uvr*B	L	excinuclease ABC subunit B
	GBS1531	UvrB	L	excinuclease ABC subunit B
C1379	SAG1465	-		protease, putative
	SAK1496	-		CAAX amino terminal protease family protein
	GBS1532	-		hypothetical protein
C1380	SAG1466	*gln*P	ET	glutamine ABC transporter, glutamine-binding protein/permease protein
	SAK1497	-	ET	polar amino acid uptake (PAAT) family ABC transporter, substrate-binding/permease protein
	GBS1533	*gln*P	ET	glutamine ABC transporter permease and substrate binding protein
C1381	SAG1467	-	E	glutamine ABC transporter, ATP-binding protein, GlnQ putative
	SAK1498	-	E	polar amino acid uptake (PAAT) family ABC transporter, ATP-binding protein
	GBS1534	*gln*Q	E	glutamine ABC transporter ATP-binding protein
C1382	SAG1468	-		hypothetical protein
	GBS1535	-		hypothetical protein
C1383	SAG1469	-		hypothetical protein
	SAK1499	-		hypothetical protein
	GBS1536	-		hypothetical protein
C1384	SAG1470	*obg*	R	GTP-binding protein, GTP1/Obg family
	SAK1500	-	R	GTP-binding protein
	GBS1537	-	R	hypothetical protein
C1385	SAG1471	-		hypothetical protein
	SAK1501	-		hypothetical protein
C1386	SAG1472	*pep*S	E	aminopeptidase PepS
	SAK1502	*pep*S	E	aminopeptidase PepS
	GBS1538	-	E	hypothetical protein
C1387	SAG1473	-		cell wall surface anchor family protein
	SAK1503	-		cell wall surface anchor family protein
	GBS1539	-		hypothetical protein
C1388	SAG1474	-		amidase family protein
	SAK1504	-		amidase family protein
	GBS1540	-		hypothetical protein
C1389	SAG1475	*rsu*A	J	ribosomal small subunit pseudouridine synthase A
	SAK1505	-	J	RNA pseudouridine synthase family protein
	GBS1541	-	J	hypothetical protein
C1390	SAG1476	-	R	oxidoreductase, aldo/keto reductase family
	SAK1506	-	R	oxidoreductase, aldo/keto reductase family
	GBS1542	-	R	hypothetical protein
C1391	SAG1477	-	C	nitroreductase family protein
	SAK1507	-	C	nitroreductase family protein
	GBS1543	-	C	hypothetical protein
C1392	SAG1478	*glo*A	E	lactoylglutathione lyase
	SAK1508	*glo*A	E	lactoylglutathione lyase
	GBS1544	-	E	hypothetical protein
C1393	SAG1479	-	M	glycosyl transferase, group 2 family protein
	SAK1509	-	M	glycosyl transferase, group 2 family protein
	GBS1545	-	M	hypothetical protein
C1394	SAG1480	-	E	amino acid permease
	SAK1510	*cyc*A	E	D-Serine/D-alanine/glycine:H+ symporter
	GBS1546	-	E	hypothetical protein
C1395	SAG1481	*smp*B	O	SsrA-binding protein
	SAK1511	*smp*B	O	SsrA-binding protein
	GBS1547	-	O	SsrA-binding protein

(Continue on next page)

List of homolog clusters in the 3 GBS reference genomes (Cont'd)

ID	Locus tag	Gene	COG	Annotation
C1396	SAG1482	*vac*B	K	exoribonuclease, VacB/Rnb family
	SAK1512	*rnr*	K	ribonuclease R
	GBS1548	-	K	hypothetical protein
C1397	SAG1483	-	U	translocase
	SAK1513	*sec*G	U	translocase
	GBS1549	-	U	translocase
C1398	SAG1484	*rpm*G-1	J	50S ribosomal protein L33
C1399	SAG1485	-		multi-drug resistance protein
	SAK1515	-		drug:H+ antiporter-1 (DHA1) family protein
	GBS1550	-		hypothetical protein
C1400	SAG1486	-		hypothetical protein
	SAK1516	-		hypothetical protein
	GBS1551	-		hypothetical protein
C1401	SAG1487	-	V	ABC transporter, ATP binding protein
	SAK1517	-	V	ABC transporter, ATP binding protein
	GBS1552	-	V	hypothetical protein
C1402	SAG1488	*coa*E	H	dephospho-CoA kinase
	SAK1518	*coa*E	H	dephospho-CoA kinase
	GBS1553	*coa*E	H	dephospho-CoA kinase
C1403	SAG1489	*mut*M	L	formamidopyrimidine-DNA glycosylase
	SAK1519	*mut*M	L	formamidopyrimidine-DNA glycosylase
	GBS1554	-	L	formamidopyrimidine-DNA glycosylase
C1404	SAG1490	-		transcriptional regulator, MutR family
	SAK1520	-		transcriptional activator, Rgg/GadR/MutR family
	GBS1555	-		hypothetical protein
C1405	SAG1491	-	D	hypothetical protein
	SAK1521	-	D	hypothetical protein
	GBS1556	-	D	hypothetical protein
C1406	SAG1492	-		hypothetical protein
C1407	SAG1493	-		hypothetical protein
C1408	SAG1494	-		hypothetical protein
	SAK1522	-		hypothetical protein
C1409	SAG1495	-		CAAX amino terminal protease family protein
	SAK1523	-		CAAX amino terminal protease family protein
	GBS1558	-		hypothetical protein
C1410	SAG1496	-		hypothetical protein
C1411	SAG1497	-		hypothetical protein
C1412	SAG1498	-		hypothetical protein
	SAK1524	-		hypothetical protein
	GBS1559	-		hypothetical protein
C1413	SAG1499	*era*	R	GTP-binding protein Era
	SAK1525	*era*	R	GTP-binding protein Era
	GBS1560	*era*	R	GTP-binding protein Era
C1414	SAG1500	*dgk*A		diacylglycerol kinase
	SAK1526	*dgk*A		diacylglycerol kinase
	GBS1561	-		hypothetical protein
C1415	SAG1501	-	R	hypothetical protein
	SAK1527	-	R	hypothetical protein
	GBS1562	-	R	hypothetical protein
C1416	SAG1502	-	Q	tetracenomycin polyketide synthesis O-methyltransferase TcmP, putative
	SAK1528	-	Q	tetracenomycin polyketide synthesis O-methyltransferase TcmP, putative
	GBS1563	-	Q	hypothetical protein
C1417	SAG1503	-		hypothetical protein
C1418	SAG1504	-		hypothetical protein
C1419	SAG1505	-	LR	MutT/nudix family protein
	SAK1529	-	LR	hydrolase, NUDIX family
	GBS1564	-	LR	hypothetical protein
C1420	SAG1506	-		hypothetical protein
	SAK1530	-		hypothetical protein
	GBS1565	-		hypothetical protein
C1421	SAG1507	-	T	PhoH family protein
	SAK1531	-	T	KH domain/PhoH family protein
	GBS1566	-	T	hypothetical protein
C1422	SAG1508	-	S	67 kDa Myosin-crossreactive streptococcal antigen
	SAK1532	-	S	67 kDa myosin-cross-reactive streptococcal antigen
	GBS1567	-	S	hypothetical protein
C1423	SAG1509	-		hypothetical protein
	SAK1533	-		hypothetical protein
	GBS1568	-		hypothetical protein

(Continue on next page)

List of homolog clusters in the 3 GBS reference genomes (Cont'd)

ID	Locus tag	Gene	COG	Annotation
C1424	SAG1510	*msr*A		methionine sulfoxide reductase A
	SAK1534	*msr*A		methionine sulfoxide reductase A
	GBS1569	-		methionine sulfoxide reductase A
C1425	SAG1511	-	S	hypothetical protein
	SAK1535	-	S	hypothetical protein
	GBS1570	-	S	hypothetical protein
C1426	SAG1512	*frr*		ribosome releasing factor
	SAK1536	*frr*		ribosome releasing factor
	GBS1571	*frr*		ribosome releasing factor
C1427	SAG1513	*pyr*H	F	uridylate kinase
	SAK1537	*pyr*H	F	uridylate kinase
	GBS1572	*pyr*H	F	uridylate kinase
C1428	SAG1514	-	EP	peptide ABC transporter, ATP-binding protein
	SAK1538	*nik*E	EP	nickel ABC transporter, ATP-binding protein
	GBS1573	-	EP	hypothetical protein
C1429	SAG1515	-	EP	peptide ABC transporter, ATP-binding protein
	SAK1539	*nik*D	EP	nickel ABC transporter, ATP-binding protein
	GBS1574	-	EP	hypothetical protein
C1430	SAG1516	-	EP	peptide ABC transporter, permease protein
	SAK1540	*nik*C	EP	nickel ABC transporter, permease protein
	GBS1575	-	EP	hypothetical protein
C1431	SAG1517	-	EP	peptide ABC transporter, permease protein
	SAK1541	*nik*B	EP	nickel ABC transporter, permease protein
	GBS1576	-	EP	hypothetical protein
C1432	SAG1518	-	E	peptide ABC transporter, peptide-binding protein
	SAK1542	*nik*A	E	nickel ABC transporter, nickel-binding protein
	GBS1577	-	E	hypothetical protein
C1433	SAG1519	*rpl*A		50S ribosomal protein L1
	SAK1543	*rpl*A		50S ribosomal protein L1
	GBSP001	*rpl*A		50S ribosomal protein L1
C1434	SAG1520	*rpl*K		50S ribosomal protein L11
	SAK1544	*rpl*K		50S ribosomal protein L11
	GBSP002	*rpl*K		50S ribosomal protein L11
C1435	SAG1521	-	L	transposase, IS30 family, putative
	SAG1795	-	L	transposase, IS30 family, putative
	SAK0164	-	L	ISSag9, transposase
	SAK0184	-	L	ISSag3, transposase
	SAK1545	-	L	ISSag9, transposase
	SAK1592	-	L	ISSag9, transposase
	GBS0208	-	L	hypothetical protein
C1436	SAG1522	-		transporter, major facilitator family
	SAK1546	-		major facilitator family protein
	GBS1580	-		hypothetical protein
C1437	SAG1523	-	R	peptidase, M20/M25/M40 family
	SAK1547	-	R	peptidase, M20D (carboxypeptidase Ss1) subfamily
	GBS1581	-	R	hypothetical protein
C1438	SAG1524	-	K	transcriptional regulator, LysR family
	SAK1548	-	K	transcriptional regulator, LysR family
	GBS1582	-	K	hypothetical protein
C1439	SAG1525	-		hypothetical protein
	GBS1583	-		hypothetical protein
C1440	SAG1528	-	EH	chorismate binding enzyme
	SAK1551	*pab*B	EH	para-aminobenzoate synthase, component I
	GBS1584	-	EH	hypothetical protein
C1441	SAG1529	-	D	FtsK/SpoIIIE family protein
	SAK1552	*fts*K	D	DNA translocase FtsK
	GBS1585	-	D	hypothetical protein
C1442	SAG1530	-	O	peptidyl-prolyl cis-trans isomerase, cyclophilin-type
	SAK1553	-	O	peptidyl-prolyl cis-trans isomerase, cyclophilin-type
	GBS1586	-	O	hypothetical protein
C1443	SAG1531	-	P	manganese ABC transporter, permease protein
	SAK1554	*mts*C	P	metal ABC transporter, permease protein
	GBS1587	-	P	hypothetical protein
C1444	SAG1532	-	P	manganese ABC transporter, ATP-binding protein
	SAK1555	*mts*B	P	metal ABC transporter, ATP-binding protein
	GBS1588	-	P	hypothetical protein
C1445	SAG1533	-	P	manganese ABC transporter, manganese-binding adhesion liprotein
	SAK1556	*mts*A	P	metal ABC transporter, metal-binding lipoprotein
	GBS1589	-	P	hypothetical protein

(Continue on next page)

List of homolog clusters in the 3 GBS reference genomes (Cont'd)

ID	Locus tag	Gene	COG	Annotation
C1446	SAG1534	-	K	iron-dependent transcriptional regulator
	SAK1557	-	K	iron-dependent repressor
	GBS1590	-	K	hypothetical protein
C1447	SAG1535	*pfs*	F	5'-methylthioadenosine/S-adenosylhomocysteine nucleosidase
	SAK1558	*pfs*	F	5'-methylthioadenosine/S-adenosylhomocysteine nucleosidase
	GBS1591	-	F	5'-methylthioadenosine/S-adenosylhomocysteine nucleosidase
C1448	SAG1536	-		hypothetical protein
	SAK1559	-		hypothetical protein
	GBS1592	-		hypothetical protein
C1449	SAG1537	-	LR	MutT/nudix family protein
	SAK1560	-	LR	hydrolase, NUDIX family
	GBS1593	-	LR	hypothetical protein
C1450	SAG1538	*glm*U	M	UDP-N-acetylglucosamine pyrophosphorylase
	SAK1561	*glm*U	M	UDP-N-acetylglucosamine pyrophosphorylase
	GBS1594	-	M	hypothetical protein
C1451	SAG1539	-		hypothetical protein
C1452	SAG1540	-	S	hypothetical protein
	SAK1562	-	S	hypothetical protein
	GBS1595	-	S	hypothetical protein
C1453	SAG1541	-	E	glyoxalase family protein
	SAK1563	-	E	glyoxalase family protein
	GBS1596	-	E	hypothetical protein
C1454	SAG1542	-	R	oxidoreductase, Gfo/Idh/MocA family
	SAK1564	-	R	oxidoreductase, Gfo/Idh/MocA family
	GBS1597	-	R	hypothetical protein
C1455	SAG1544	*fab*G	IQR	3-ketoacyl-(acyl-carrier-protein) reductase
	SAK1566	*fab*G	IQR	3-ketoacyl-(acyl-carrier-protein) reductase
	GBS1600	*fab*G	IQR	3-ketoacyl-(acyl-carrier-protein) reductase
C1456	SAG1545	-		hypothetical protein
	SAK1567	-		hypothetical protein
	GBS1601	-		hypothetical protein
C1457	SAG1546	-		hypothetical protein
	SAK1568	-		hypothetical protein
	GBS1602	-		hypothetical protein
C1458	SAG1547	-		acetyltransferase, GNAT family
	SAK1569	-		acetyltransferase, GNAT family
	GBS1603	-		hypothetical protein
C1459	SAG1548	-	M	glycosyl transferase, group 2 family protein
	SAG1551	-		hypothetical protein
	SAK1570	-	M	glycosyl transferase, group 2 family protein
	GBS1605	-	M	hypothetical protein
C1460	SAG1552	-		hypothetical protein
	SAK1571	-		hypothetical protein
	GBS1606	-		hypothetical protein
C1461	SAG1553	-		hypothetical protein
	SAK1572	-		hypothetical protein
	GBS1607	-		hypothetical protein
C1462	SAG1554	-	S	hypothetical protein
	SAK1573	-	S	hypothetical protein
	GBS1608	-	S	hypothetical protein
C1463	SAG1555	-		hypothetical protein
	SAK1574	-		hypothetical protein
	GBS1609	-		hypothetical protein
C1464	SAG1556	*brn*Q-1	E	branched-chain amino acid transport system II carrier protein
	SAG1636	*brn*Q-2	E	branched-chain amino acid transport system II carrier protein
	SAK1575	*brn*Q	E	branched chain amino acid:H+ symporter
	GBS1610	-	E	hypothetical protein
	GBS1683	-	E	hypothetical protein
C1465	SAG1557	*met*G	J	methionine–tRNA ligase
	SAK1576	*met*G	R	methionine–tRNA ligase
	GBS1611	*met*G	J	methionine–tRNA ligase
C1466	SAG1558	*teh*B	P	tellurite resistance protein TehB
	SAK1577	-	QR	tellurite resistance protein TehB, putative
	GBS1612	-	P	hypothetical protein
C1467	SAG1559	-		hypothetical protein
	SAK1578	-		hypothetical protein
	GBS1613	-		hypothetical protein
C1468	SAG1560	-		hypothetical protein
C1469	SAG1561	-	G	PTS system, IIC component, putative
	SAK1579	-	G	PTS system, putative, lactose/cellobiose family IIC component
	GBS1614	-	G	hypothetical protein

(Continue on next page)

List of homolog clusters in the 3 GBS reference genomes (Cont'd)

ID	Locus tag	Gene	COG	Annotation
C1470	SAG1562	-	R	hypothetical protein
	SAK1580	-	R	hypothetical protein
	GBS1615	-	R	hypothetical protein
C1471	SAG1563	*exo*A	L	exodeoxyribonuclease
	SAK1581	*xth*	L	exodeoxyribonuclease III
	GBS1616	-	L	hypothetical protein
C1472	SAG1564	-	P	hypothetical protein
	SAK1582	-	P	ArsC family protein
	GBS1617	-	P	hypothetical protein
C1473	SAG1565	*ogt*	L	methylated-DNA–protein-cysteine S-methyltransferase
	SAK1583	-	L	methylated-DNA–protein-cysteine S-methyltransferase, putative
	GBS1618	-	L	hypothetical protein
C1474	SAG1566	-	HE	D-isomer specific 2-hydroxyacid dehydrogenase family protein
	SAK1584	-	HE	D-3-phosphoglycerate dehydrogenase, putative
	GBS1619	-	HE	hypothetical protein
C1475	SAG1567	-		acetyltransferase, GNAT family
	SAK1585	-		acetyltransferase, GNAT family
	GBS1620	-		hypothetical protein
C1476	SAG1569	-	P	copper homeostasis protein CutC, putative
	SAK1587	-	P	CutC family protein
	GBS1622	-	P	hypothetical protein
C1477	SAG1570	-		hypothetical protein
C1478	SAG1571	-		hypothetical protein
C1479	SAG1572	-	R	tetrapyrrole methylase family protein
	SAK1588	-	R	tetrapyrrole methylase family protein
	GBS1623	-	R	hypothetical protein
C1480	SAG1573	-	S	hypothetical protein
	SAK1589	-	S	hypothetical protein
	GBS1624	-	S	hypothetical protein
C1481	SAG1574	-	L	DNA polymerase III subunit delta
	SAK1590	*hol*B	L	DNA polymerase III subunit delta
	GBS1625	-	L	DNA polymerase III subunit delta
C1482	SAG1575	*tmk*	F	thymidylate kinase
	SAK1591	*tmk*	F	thymidylate kinase
	GBS1626	-	F	thymidylate kinase
C1483	SAG1577	-	R	AcuB family protein
	SAK1593	-	R	CBS domain protein
	GBS1627	-	R	hypothetical protein
C1484	SAG1578	*liv*F	E	branched-chain amino acid ABC transporter, ATP-binding protein
	SAK1594	*liv*F	E	hydrophobic amino acid uptake (HAAT) family ABC transporter, ATP-binding protein LivF
	GBS1628	-	E	hypothetical protein
C1485	SAG1579	*liv*G	E	branched-chain amino acid ABC transporter, ATP-binding protein
	SAK1595	*liv*G	E	hydrophobic amino acid uptake (HAAT) family ABC transporter, ATP-binding protein LivG
	GBS1629	-	E	hypothetical protein
C1486	SAG1580	-	E	branched-chain amino acid ABC transporter, permease protein
	SAK1596	*liv*M	E	hydrophobic amino acid uptake (HAAT) family ABC transporter, permease protein LivM
	GBS1630	-	E	hypothetical protein
C1487	SAG1581	*liv*H	E	branched-chain amino acid ABC transporter, permease protein
	SAK1597	*liv*H	E	hydrophobic amino acid uptake (HAAT) family ABC transporter, permease protein LivH
	GBS1631	-	E	hypothetical protein
C1488	SAG1582	-	E	branched-chain amino acid ABC transporter, amino acid-binding protein
	GBS1632	-	E	hypothetical protein
C1489	SAG1583	-		hypothetical protein
	SAK1599	-		hypothetical protein
	GBS1633	-		hypothetical protein
C1490	SAG1584	-	L	IS1548 transposase
C1491	SAG1585	*clp*P	OU	ATP-dependent Clp protease proteolytic subunit
	SAK0747	-	OU	prophage LambdaSa04, ClpP endopeptidase (S14) family, non-peptidase homologue, putative
	SAK1600	*clp*P	OU	ATP-dependent Clp protease proteolytic subunit
	GBS1634	*clp*P	OU	ATP-dependent Clp protease proteolytic subunit
C1492	SAG1586	*upp*	F	uracil phosphoribosyltransferase
	SAK1601	*upp*	F	uracil phosphoribosyltransferase
	GBS1635	*upp*	F	uracil phosphoribosyltransferase
C1493	SAG1587	-	E	aminotransferase, class I
	SAK1602	-	E	aminotransferase, classes I and II
	GBS1636	-	E	hypothetical protein

(Continue on next page)

List of homolog clusters in the 3 GBS reference genomes (Cont'd)

ID	Locus tag	Gene	COG	Annotation
	SAG1588	-	J	RNA methyltransferase, TrmH family, group 2
C1494	SAK1603	-	J	RNA methyltransferase, TrmH family
	GBS1637	-	J	hypothetical protein
	SAG1589	-	E	amino acid permease, putative
C1495	SAK1604	-	E	permease, amino acid-polyamine-organocation (APC) family
	GBS1638	-	E	hypothetical protein
	SAG1590	-	P	potassium uptake protein, Trk family
C1496	SAK1605	-	P	K+ transport/nucleotide-binding regulatory domain/protein (KTN) family protein TrkA, putative
	GBS1639	-	P	hypothetical protein
	SAG1591	-	P	cation uptake protein, Trk family
C1497	SAK1606	-	P	K+ transporter (Trk) family protein TrkH, putative
	GBS1640	-	P	hypothetical protein
	SAG1592	-		hypothetical protein
C1498	SAK1607	-		hypothetical protein
	GBS1641	-		hypothetical protein
	SAG1593	*rlu*B	J	ribosomal large subunit pseudouridine synthase B
C1499	SAK1608	*rlu*B	J	ribosomal large subunit pseudouridine synthase B
	GBS1642	-	J	hypothetical protein
	SAG1594	*scp*B		segregation and condensation protein B
C1500	SAK1609	*scp*B		segregation and condensation protein B
	GBS1643	*scp*B		segregation and condensation protein B
	SAG1595	*scp*A	S	segregation and condensation protein A
C1501	SAK1610	*scp*A	S	segregation and condensation protein A
	GBS1644	*scp*A	S	segregation and condensation protein A
	SAG1596	-		tyrosine recombinase
C1502	SAK1611	-		tyrosine recombinase
	GBS1645	-		tyrosine recombinase
	SAG1597	-	R	CBS domain protein
C1503	SAK1612	-	R	CBS domain protein
	GBS1646	-	R	hypothetical protein
	SAG1598	-	R	hypothetical protein
C1504	SAK1613	-	R	phosphoesterase family protein
	GBS1647	-	R	hypothetical protein
	SAG1599	-	F	putative deoxyribonucleotide triphosphate pyrophosphatase/unknown domain fusion protein
C1505				
	SAK1614	*rdg*B	F	putative deoxyribonucleotide triphosphate pyrophosphatase/unknown domain fusion protein
	GBS1648	-	F	putative deoxyribonucleotide triphosphate pyrophosphatase/unknown domain fusion protein
	SAG1600	*mur*I	M	glutamate racemase
C1506	SAK1615	*mur*I	M	glutamate racemase
	GBS1649	-	M	glutamate racemase
	SAG1601	-	S	hypothetical protein
C1507	SAK1616	-	S	hypothetical protein
	GBS1650	-	S	hypothetical protein
	SAG1602	-		hypothetical protein
C1508	SAK1617	-		hypothetical protein
	GBS1651	-		hypothetical protein
	SAG1603	-		transcriptional regulator, biotin repressor family
C1509	SAK1618	-		3H domain protein
	GBS1652	-		hypothetical protein
	SAG1604	-	R	hypothetical protein
C1510	SAK1619	-	R	hypothetical protein
	GBS1653	-	R	hypothetical protein
	SAG1605	-	R	hypothetical protein
C1511	SAK1620	-	R	hypothetical protein
	GBS1654	-	R	hypothetical protein
	SAG1606	-	J	RNA methyltransferase, TrmH family
C1512	SAK1621	-	J	RNA methyltransferase, TrmH family
	GBS1655	-	J	hypothetical protein
	SAG1607	-	C	acylphosphatase
C1513	SAK1622	-	C	acylphosphatase
	GBS1656	-	C	hypothetical protein
	SAG1608	-	U	OxaA-like protein precursor
C1514	SAK1623	-	U	OxaA-like protein precursor
	GBS1657	-	U	OxaA-like protein precursor
	SAG1609	-	E	amino acid ABC transporter, permease protein
C1515	SAK1624	-	E	polar amino acid uptake (PAAT) family ABC transporter, permease protein
	GBS1658	-	E	hypothetical protein

(Continue on next page)

List of homolog clusters in the 3 GBS reference genomes (Cont'd)

ID	Locus tag	Gene	COG	Annotation
C1516	SAG1610	-	ET	amino acid ABC transporter, substrate-binding protein
	SAK1625	-	ET	polar amino acid uptake (PAAT) family ABC transporter, polar amino acid-binding protein
	GBS1659	-	ET	hypothetical protein
C1517	SAG1611	-		amidase
	SAK1626	-		amidase
	GBS1660	-		amidase
C1518	SAG1612	*gre*A	K	transcription elongation factor GreA
	SAK1627	*gre*A	K	transcription elongation factor GreA
	GBS1661	*gre*A	K	transcription elongation factor GreA
C1519	SAG1613	-		hypothetical protein
	SAK1628	-		conserved hypothetical protein TIGR00247
	GBS1662	-		hypothetical protein
C1520	SAG1614	-		acetyltransferase, GNAT family
	SAK1629	-		acetyltransferase, GNAT family
	GBS1663	-		hypothetical protein
C1521	SAG1615	*mur*C	M	UDP-N-acetylmuramate–L-alanine ligase
	SAK1630	*mur*C	M	UDP-N-acetylmuramate–L-alanine ligase
	GBS1664	*mur*C	M	UDP-N-acetylmuramate–L-alanine ligase
C1522	SAG1616	-		hypothetical protein
	SAK1631	-		hypothetical protein
	GBS1665	-		hypothetical protein
C1523	SAG1617	-		hypothetical protein
	SAK1632	-		hypothetical protein
C1524	SAG1619	-	L	IS1548 transposase
C1525	SAG1620	-	R	GTP-binding protein EngA
	SAK1634	-	R	GTP-binding protein EngA
	GBS1667	-	R	GTP-binding protein EngA
C1526	SAG1621	*dna*I	L	primosomal protein DnaI
	SAK1635	*dna*I	L	primosomal protein DnaI
	GBS1668	*dna*I	L	primosomal protein DnaI
C1527	SAG1622	-	L	hypothetical protein
	SAK1636	-	L	replication initiation and membrane attachment protein, putative
	GBS1669	-	L	hypothetical protein
C1528	SAG1623	-		hypothetical protein
	SAK1637	-		hypothetical protein
	GBS1670	-		hypothetical protein
C1529	SAG1624	*csr*S	T	sensor histidine kinase CsrS
	SAK1638	-	T	sensor histidine kinase CsrS, putative
	GBS1671	-	T	hypothetical protein
C1530	SAG1625	*csr*R	TK	DNA-binding response regulator CsrR
	SAK1639	*csr*R	TK	DNA-binding response regulator CsrR
	GBS1672	-	TK	hypothetical protein
C1531	SAG1626	-	R	hypothetical protein
	SAK1640	-	R	hypothetical protein
	GBS1673	-	R	hypothetical protein
C1532	SAG1627	*htp*X	O	heat shock protein HtpX
	SAK1641	*htp*X	O	heat shock protein HtpX
	GBS1674	-	O	heat shock protein HtpX
C1533	SAG1628	*lem*A		lemA protein
	SAK1642	-		LemA family protein
	GBS1675	-		hypothetical protein
C1534	SAG1629	*gid*B	M	glucose-inhibited division protein B
	SAK1643	*gid*B	M	glucose-inhibited division protein B
	GBS1676	*gid*B	M	glucose-inhibited division protein B
C1535	SAG1630	-	P	sodium transport family protein
	SAK1644	-	P	K+ transporter (Trk) family protein
	GBS1677	-	P	hypothetical protein
C1536	SAG1631	-	P	potassium uptake protein, Trk family, putative
	SAK1645	-	P	potassium uptake protein, K+ transporter (Trk) family
	GBS1678	-	P	hypothetical protein
C1537	SAG1632	-	P	cobalt transport family protein
	SAK1646	-	P	nickel/cobalt uptake transporter (NiCoT) family ABC transporter, permease protein
	GBS1679	-	P	hypothetical protein
C1538	SAG1633	-	P	ABC transporter, ATP-binding protein
	SAK1647	-	P	ABC transporter, ATP-binding protein
	GBS1680	-	P	hypothetical protein
C1539	SAG1634	-	S	hypothetical protein
	SAK1648	-	S	hypothetical protein
	GBS1681	-	S	hypothetical protein

(Continue on next page)

List of homolog clusters in the 3 GBS reference genomes (Cont'd)

ID	Locus tag	Gene	COG	Annotation
C1540	SAG1635	-	E	sodium:dicarboxylate symporter family protein
	SAK1649	*ygj*U	E	dicarboxylate/amino acid,cation (Na+ or H+) symporter (DAACS) family protein YgjU
	GBS1682	-	E	hypothetical protein
C1541	SAG1638	-	P	ABC transporter, permease protein
	SAK1652	*met*I	P	D-methionine ABC transporter, permease protein
	GBS1685	-	P	hypothetical protein
C1542	SAG1639	-	P	ABC transporter, ATP-binding protein
	SAK1653	-	P	amino acid ABC transporter, ATP-binding protein, putative
	GBS1686	-	P	hypothetical protein
C1543	SAG1640	-	E	hypothetical protein
	SAK1654	-	E	hypothetical protein
	GBS1687	-	E	hypothetical protein
C1544	SAG1641	-	P	YaeC family protein
	SAK1655	-	P	lipoprotein, NLPA family
	GBS1688	-	P	hypothetical protein
C1545	SAG1642	-	ET	ABC transporter, substrate-binding protein
	SAK1656	-	ET	amino acid ABC transporter, amino acid-binding protein, putative
	GBS1689	-	ET	hypothetical protein
C1546	SAG1643	-	R	glutamine amidotransferase, class I
	SAK1657	-	R	glutamine amidotransferase class I domain protein
	GBS1690	-	R	hypothetical protein
C1547	SAG1644	-		hypothetical protein
C1548	SAG1645	-		hypothetical protein
	SAK1658	-		hypothetical protein
	GBS1691	-		hypothetical protein
C1549	SAG1646	-		hypothetical protein
C1550	SAG1647	-	G	dihydroxyacetone kinase family protein
	SAK1659	-	G	dihydroxyacetone kinase DAK1 domain protein
	GBS1692	-	G	hypothetical protein
C1551	SAG1648	-	K	transcriptional regulator, TetR family, putative
	SAK1660	-	K	transcriptional regulator, putative
	GBS1693	-	K	hypothetical protein
C1552	SAG1649	-		hypothetical protein
	SAK1661	-		hypothetical protein
C1553	SAG1650	-	G	dihydroxyacetone kinase family protein
	SAK1662	-	G	dihydroxyacetone kinase DAK1 domain protein
	GBS1694	-	G	hypothetical protein
C1554	SAG1651	-	G	dihydroxyacetone kinase family protein
	SAK1663	-	G	dihydroxyacetone kinase DAK2 domain protein
	GBS1695	-	G	hypothetical protein
C1555	SAG1652	-	S	hypothetical protein
	SAK1664	-	S	hypothetical protein
	GBS1696	-	S	hypothetical protein
C1556	SAG1653	*glp*F-2		glycerol uptake facilitator protein
	SAK1665	*glp*F		glycerol uptake facilitator protein
	GBS1697	-		hypothetical protein
C1557	SAG1654	-	G	hypothetical protein
	SAK1666	-	G	hypothetical protein
	GBS1698	-	G	hypothetical protein
C1558	SAG1655	-	K	transcriptional regulator, MerR family
	SAK1667	-	K	transcriptional regulator, MerR family
	GBS1699	-	K	hypothetical protein
C1559	SAG1656	-	R	hypothetical protein
	SAK1668	-	R	hypothetical protein
	GBS1700	-	R	hypothetical protein
C1560	SAG1657	-		hypothetical protein
	SAK1669	-		hypothetical protein
	GBS1701	-		hypothetical protein
C1561	SAG1658	-	QR	hypothetical protein
	SAK1670	-	QR	hypothetical protein
	GBS1702	-	QR	hypothetical protein
C1562	SAG1659	-		iojap-related protein
	SAK1671	-		iojap-related protein
	GBS1703	-		hypothetical protein
C1563	SAG1660	-	Q	isochorismatase family protein
	SAK1672	-	Q	isochorismatase family protein
	GBS1704	-	Q	hypothetical protein
C1564	SAG1661	-	H	conserved hypothetical protein TIGR00488
	SAK1673	-	H	HD domain protein
	GBS1705	-	H	hypothetical protein

(Continue on next page)

List of homolog clusters in the 3 GBS reference genomes (Cont'd)

ID	Locus tag	Gene	COG	Annotation
	SAG1662	*nad*D	H	nicotinic acid mononucleotide adenyltransferase
C1565	SAK1674	*nad*D	H	nicotinic acid mononucleotide adenyltransferase
	GBS1706	*nad*D	H	nicotinic acid mononucleotide adenyltransferase
	SAG1663	-		conserved hypothetical protein TIGR00253
C1566	SAK1675	-		conserved hypothetical protein TIGR00253
	GBS1707	-		hypothetical protein
	SAG1664	-	R	GTP-binding protein
C1567	SAK1676	-	R	GTP-binding protein
	GBS1708	-	R	hypothetical protein
	SAG1665	-	R	hydrolase, haloacid dehalogenase-like family
C1568	SAK1677	-	R	hydrolase, HAD subfamily IIIA
	GBS1709	-	R	hypothetical protein
	SAG1666	-	GER	hypothetical protein
C1569	SAK1678	-	GER	integral membrane protein
	GBS1710	-	GER	hypothetical protein
	SAG1667	*gat*B	J	aspartyl/glutamyl-tRNA amidotransferase subunit B
C1570	SAK1679	*gat*B	J	aspartyl/glutamyl-tRNA amidotransferase subunit B
	GBS1711	-	J	aspartyl/glutamyl-tRNA amidotransferase subunit B
	SAG1668	*gat*A		glutamyl-tRNA amidotransferase subunit A
C1571	SAK1680	*gat*A		glutamyl-tRNA amidotransferase subunit A
	GBS1712	-		glutamyl-tRNA amidotransferase subunit A
	SAG1669	*gat*C	J	aspartyl/glutamyl-tRNA amidotransferase subunit C
C1572	SAK1681	*gat*C	J	aspartyl/glutamyl-tRNA amidotransferase subunit C
	GBS1713	-	J	aspartyl/glutamyl-tRNA amidotransferase subunit C
	SAG1670	*ppd*K	G	pyruvate phosphate dikinase
C1573	SAK1682	*ppd*K	G	pyruvate phosphate dikinase
	GBS1714	-	G	pyruvate phosphate dikinase
	SAG1671	-		hypothetical protein
C1574	SAK1683	-		hypothetical protein
	GBS1715	-		hypothetical protein
	SAG1672	-	R	CBS domain protein
C1575	SAK1684	-	R	CBS domain protein
	GBS1716	-	R	hypothetical protein
	SAG1673	-	I	3-hydroxybutyryl-CoA dehydrogenase
C1576	SAK1685	-	I	3-hydroxybutyryl-CoA dehydrogenase
	GBS1717	-	I	3-hydroxybutyryl-CoA dehydrogenase
	SAG1674	-	Q	isochorismatase family protein
C1577	SAK1686	-	Q	isochorismatase family protein
	GBS1718	-	Q	hypothetical protein
	SAG1675	-		transcriptional repressor CodY
C1578	SAK1687	*cod*Y		transcriptional repressor CodY
	GBS1719	-		transcriptional repressor CodY
	SAG1676	-	E	aspartate aminotransferase
C1579	SAK1688	-	E	aspartate aminotransferase
	GBS1720	-	E	aspartate aminotransferase
	SAG1677	-	T	hypothetical protein
C1580	SAK1689	-	T	universal stress family protein
	GBS1721	-	T	hypothetical protein
	SAG1678	-	S	hydrolase, haloacid dehalogenase-like family
C1581	SAK1690	-	S	Cof-like hydrolase family protein
	GBS1722	-	S	hypothetical protein
	SAG1679	-		asparaginase family protein
C1582	SAK1691	-		asparaginase family protein
	GBS1723	-		hypothetical protein
	SAG1680	*aro*E	E	shikimate 5-dehydrogenase
C1583	SAK1692	*aro*E	E	shikimate 5-dehydrogenase
	GBS1724	-	E	hypothetical protein
	SAG1681	-	R	oxidoreductase, aldo/keto reductase family
C1584	SAK1693	-	R	oxidoreductase, aldo/keto reductase family
	GBS1725	-	R	hypothetical protein
	SAG1682	*rec*G	LK	ATP-dependent DNA helicase RecG
C1585	SAK1694	*rec*G	LK	ATP-dependent DNA helicase RecG
	GBS1726	*rec*G	LK	hypothetical protein
	SAG1684	*alr*	M	alanine racemase
C1586	SAK1696	*alr*	M	alanine racemase
	GBS1728	*alr*	M	alanine racemase
	SAG1685	*acp*S	I	4'-phosphopantetheinyl transferase
C1587	SAK1697	*acp*S	I	4'-phosphopantetheinyl transferase
	GBS1729	*acp*S	I	4'-phosphopantetheinyl transferase

(Continue on next page)

List of homolog clusters in the 3 GBS reference genomes (Cont'd)

ID	Locus tag	Gene	COG	Annotation
C1588	SAG1686	-	E	3-deoxy-7-phosphoheptulonate synthase
	SAK1698	-	E	3-deoxy-7-phosphoheptulonate synthase
	GBS1730	-	E	3-deoxy-7-phosphoheptulonate synthase
C1589	SAG1687	*sec*A	U	translocase
	SAK1699	*sec*A	U	translocase
	GBS1731	-	U	translocase
C1590	SAG1688	*man*A	G	mannose-6-phosphate isomerase, class I
	SAK1700	*man*A	G	mannose-6-phosphate isomerase, class I
	GBS1732	-	G	hypothetical protein
C1591	SAG1689	*scr*K	KG	fructokinase
	SAK1701	*scr*K	KG	fructokinase
	GBS1733	-	KG	hypothetical protein
C1592	SAG1690	-	G	PTS system, IIABC components
	SAK1702	-	G	PTS system, sucrose-specific IIABC component
	GBS1734	-	G	hypothetical protein
C1593	SAG1691	*scr*B		sucrose-6-phosphate hydrolase
	SAK1703	*scr*B		sucrose-6-phosphate hydrolase
	GBS1735	-		hypothetical protein
C1594	SAG1692	*scr*R	K	sucrose operon repressor ScrR
	SAK1704	*scr*R	K	sucrose operon repressor ScrR
	GBS1736	-	K	hypothetical protein
C1595	SAG1693	*nus*B	K	transcription antitermination protein NusB
	SAK1705	*nus*B	K	transcription antitermination protein NusB
	GBS1737	*nus*B	K	transcription antitermination protein NusB
C1596	SAG1694	-		hypothetical protein
	SAK1706	-		hypothetical protein
	GBS1738	-		hypothetical protein
C1597	SAG1695	*efp*	J	elongation factor P
	SAK1707	*efp*	J	elongation factor P
	GBS1739	-	J	elongation factor P
C1598	SAG1696	-		hypothetical protein
C1599	SAG1697	-		hypothetical protein
C1600	SAG1699	-		hypothetical protein
C1601	SAG1700	-		hypothetical protein
	SAK1116	-		hypothetical protein
	SAK1709	-		hypothetical protein
C1602	SAG1701	-		hypothetical protein
	SAK1710	-		hypothetical protein
C1603	SAG1702	-		hypothetical protein
	SAK1711	-		hypothetical protein
C1604	SAG1703	-		hypothetical protein
	SAK1712	-		hypothetical protein
C1605	SAG1704	-	F	cytidine/deoxycytidylate deaminase family protein
	SAK1713	-	F	cytidine/deoxycytidylate deaminase family protein
	GBS1750	-	F	hypothetical protein
C1606	SAG1706	-		hypothetical protein
	SAK1715	-		hypothetical protein
	GBS1752	-		hypothetical protein
C1607	SAG1707	-		drug resistance transporter, EmrB/QacA family
	SAK1716	-		drug:H+ antiporter-2 (DHA2) family protein
	GBS1753	-		hypothetical protein
C1608	SAG1708	-		hypothetical protein
C1609	SAG1709	*uvr*A	L	excinuclease ABC subunit A
	SAK1717	*uvr*A	L	excinuclease ABC subunit A
	GBS1754	*uvr*A	L	excinuclease ABC subunit A
C1610	SAG1710	-		hypothetical protein
	SAK1718	-		hypothetical protein
	GBS1755	-		hypothetical protein
C1611	SAG1711	-	P	magnesium transporter, CorA family
	SAK1719	-	P	CorA metal ion transporter (MIT) family protein
	GBS1756	-	P	hypothetical protein
C1612	SAG1712	*rps*R		30S ribosomal protein S18
	SAK1720	*rps*R		30S ribosomal protein S18
	GBS1757	*rps*R		30S ribosomal protein S18
C1613	SAG1714	*rps*F	J	30S ribosomal protein S6
	SAK1722	*rps*F	J	30S ribosomal protein S6
	GBS1759	*rps*F	J	30S ribosomal protein S6
C1614	SAG1715	*mut*Y	L	A/G-specific adenine glycosylase
	SAK1723	*mut*Y	L	A/G-specific adenine glycosylase
	GBS1760	-	L	hypothetical protein

(Continue on next page)

List of homolog clusters in the 3 GBS reference genomes (Cont'd)

ID	Locus tag	Gene	COG	Annotation
C1615	SAG1716	-	K	transcriptional regulator, Cro/CI family
	SAK1724	-	K	DNA-binding protein
	GBS1761	-	K	hypothetical protein
C1616	SAG1717	*trx*	OC	thioredoxin
	SAK1725	*trx*	OC	thioredoxin
	GBS1762	-	OC	hypothetical protein
C1617	SAG1718	-		PAP2 family protein
	GBS1763	-		hypothetical protein
C1618	SAG1719	-	L	MutS2 family protein
	SAK1727	-	L	MutS2 family protein
	GBS1764	-	L	hypothetical protein
C1619	SAG1720	-		hypothetical protein
	SAK1728	-		CvpA family protein
	GBS1765	-		hypothetical protein
C1620	SAG1721	-		hypothetical protein
	SAK1729	-		hypothetical protein
	GBS1766	-		hypothetical protein
C1621	SAG1722	*rnh*C	L	ribonuclease HIII
	SAK1730	*rnh*C	L	ribonuclease HIII
	GBS1767	-	L	ribonuclease HIII
C1622	SAG1723	-	U	signal peptidase I
	SAK1443	*lep*B	U	signal peptidase I
	SAK1731	*lep*B	U	signal peptidase I
	GBS1768	-	U	hypothetical protein
C1623	SAG1724	-	L	helicase, putative
	SAK1732	-	L	helicase, RecD/TraA family
	GBS1769	-	L	hypothetical protein
C1624	SAG1725	-		hypothetical protein
	SAK1733	-		hypothetical protein
	GBS1770	-		hypothetical protein
C1625	SAG1727	*pfl*D-2	C	formate acetyltransferase
	SAK1735	*pfl*B	C	formate acetyltransferase 1
	GBS1772	-	C	hypothetical protein
C1626	SAG1728	-		FMN-binding protein
	SAK1736	-		FMN-binding protein
	GBS1773	-		hypothetical protein
C1627	SAG1729	-	V	hypothetical protein
	SAK1737	-	V	beta-lactamase, putative
	GBS1774	-	V	hypothetical protein
C1628	SAG1730	-	E	hypothetical protein
	SAK1738	-	E	hypothetical protein
	GBS1775	-		hypothetical protein
C1629	SAG1731	-		hypothetical protein
	SAK1739	-		hypothetical protein
	GBS1776	-		hypothetical protein
C1630	SAG1732	-		glycerol uptake facilitator protein, putative
	SAK1740	-		glycerol facilitator/aquaporin
	GBS1777	-		hypothetical protein
C1631	SAG1733	-	T	universal stress protein family
	SAK1741	-	T	universal stress family protein
	GBS1778	-	T	hypothetical protein
C1632	SAG1734	-		transporter, putative
	SAK1742	-		major facilitator family protein
	GBS1779	-		hypothetical protein
C1633	SAG1735	-	T	transcriptional regulator, Crp/Fnr family
	SAK1743	-	T	transcriptional regulator, putative
	GBS1780	-	T	hypothetical protein
C1634	SAG1736	*pep*X	R	x-prolyl-dipeptidyl aminopeptidase
	SAK1744	*pep*X	R	x-prolyl-dipeptidyl aminopeptidase
	GBS1781	-	R	x-prolyl-dipeptidyl aminopeptidase
C1635	SAG1737	-		hypothetical protein
	SAK1745	-		hypothetical protein
	GBS1782	-		hypothetical protein
C1636	SAG1738	-	H	polyprenyl synthetase family protein
	SAK1746	-	H	polyprenyl synthetase family protein
	GBS1783	-	H	hypothetical protein
C1637	SAG1739	*cyd*C	CO	ABC transporter, ATP-binding protein CydC
	SAK1747	*cyd*C	CO	ABC transporter, ATP-binding protein CydC
	GBS1784	-	CO	hypothetical protein

(Continue on next page)

List of homolog clusters in the 3 GBS reference genomes (Cont'd)

ID	Locus tag	Gene	COG	Annotation
C1638	SAG1740	-	CO	ABC transporter, ATP-binding protein CydD
	SAK1748	*cyd*D	CO	ABC transporter, ATP-binding protein CydD
	GBS1741	-	V	hypothetical protein
	GBS1785	-	CO	hypothetical protein
C1639	SAG1741	*cyd*B	C	cytochrome d ubiquinol oxidase, subunit II
	SAK1749	*cyd*B	C	cytochrome d ubiquinol oxidase, subunit II
	GBS1786	-	C	hypothetical protein
C1640	SAG1742	*cyd*A	C	cytochrome d oxidase, subunit I
	SAK1750	*cyd*A	C	cytochrome d ubiquinol oxidase, subunit II
	GBS1787	-	C	hypothetical protein
C1641	SAG1743	-	C	pyridine nucleotide-disulphide oxidoreductase family protein
	SAK1751	-	C	pyridine nucleotide-disulphide oxidoreductase family protein
	GBS1788	-	C	hypothetical protein
C1642	SAG1744	-	H	1,4-dihydroxy-2-naphthoate octaprenyltransferase
	SAK1752	-	H	1,4-dihydroxy-2-naphthoate octaprenyltransferase,
	GBS1789	-	H	1,4-dihydroxy-2-naphthoate octaprenyltransferase
C1643	SAG1745	-		hypothetical protein
	SAK1753	-		hypothetical protein
	GBS1790	-		hypothetical protein
C1644	SAG1746	-		hypothetical protein
C1645	SAG1747	-	S	hypothetical protein
	SAK1770	-	S	hypothetical protein
	GBS1791	-	S	hypothetical protein
C1646	SAG1748	*cfa*	M	cyclopropane-fatty-acyl-phospholipid synthase
	SAK1771	*cfa*	M	cyclopropane-fatty-acyl-phospholipid synthase
	GBS1792	-	M	hypothetical protein
C1647	SAG1749	-	K	transcriptional regulator, MerR family
	SAK1772	-	K	transcriptional regulator, MerR family
	GBS1793	-	K	hypothetical protein
C1648	SAG1750	-	L	DNA polymerase III subunit epsilon
	SAK1773	-	L	DNA polymerase III subunit epsilon
	GBS1794	-	L	DNA polymerase III subunit epsilon
C1649	SAG1751	-		hypothetical protein
	SAK1774	-		hypothetical protein
	GBS1795	-		hypothetical protein
C1650	SAG1752	-	R	conserved hypothetical protein TIGR00275
	SAK1775	-	R	conserved hypothetical protein TIGR00275
	GBS1796	-	R	hypothetical protein
C1651	SAG1753	-	C	hypothetical protein
	SAK1776	-	C	hypothetical protein
	GBS1797	-	C	hypothetical protein
C1652	SAG1754	*rps*N	J	ribosomal protein S14
	SAK1777	*rps*N	J	ribosomal protein S14
	GBS1798	-	J	30S ribosomal protein S14
C1653	SAG1755	-		hypothetical protein
C1654	SAG1756	-	E	hypothetical protein
	SAK1778	*lta*E	E	low specificity L-threonine aldolase
	GBS1799	-	E	hypothetical protein
C1655	SAG1757	-	O	O-sialoglycoprotein endopeptidase family protein
	SAK1779	-	O	metalloendopeptidase, putative, glycoprotease family
	GBS1800	-	O	hypothetical protein
C1656	SAG1758	-	R	ribosomal-protein-alanine acetyltransferase, putative
	SAK1780	*rim*I	R	ribosomal-protein-alanine acetyltransferase
	GBS1801	-	R	hypothetical protein
C1657	SAG1759	-	O	hypothetical protein
	SAK1781	-	O	peptidase, putative, M22 (O-sialoglycoprotein endopeptidase) family
	GBS1802	-	O	hypothetical protein
C1658	SAG1760	-		hypothetical protein
	SAK1782	-		hypothetical protein
	GBS1803	-		hypothetical protein
C1659	SAG1761	-	R	metallo-beta-lactamase superfamily protein
	SAK1783	-	R	metallo-beta-lactamase family protein
	GBS1804	-	R	hypothetical protein
C1660	SAG1762	-	R	hypothetical protein
	SAK1784	-	R	CHAP domain protein
	GBS1805	-	R	hypothetical protein
C1661	SAG1763	*gln*A		glutamine synthetase, type I
	SAK1785	*gln*A		glutamine synthetase, type I
	GBS1806	*gln*A		glutamine synthetase

(Continue on next page)

List of homolog clusters in the 3 GBS reference genomes (Cont'd)

ID	Locus tag	Gene	COG	Annotation
C1662	SAG1764	*gln*R	K	transcriptional regulator GlnR
	SAK1786	*gln*R	K	transcriptional regulator GlnR
	GBS1807	-	K	hypothetical protein
C1663	SAG1765	-	S	hypothetical protein
	SAK1787	-	S	hypothetical protein
	GBS1808	-	S	hypothetical protein
C1664	SAG1766	*pgk*		phosphoglycerate kinase
	SAK1788	*pgk*		phosphoglycerate kinase
	GBS1809	-		phosphoglycerate kinase
C1665	SAG1767	-	R	acid phosphatase
	SAK1789	-	R	5'-nucleotidase, lipoprotein e(P4) family
	GBS1810	-	R	hypothetical protein
C1666	SAG1768	*gap*	G	glyceraldehyde-3-phosphate dehydrogenase
	SAK1790	*gap*	G	glyceraldehyde-3-phosphate dehydrogenase
	GBS1811	-	G	glyceraldehyde-3-phosphate dehydrogenase
C1667	SAG1770	*rps*G	J	30S ribosomal protein S7
	SAK1792	*rps*G	J	30S ribosomal protein S7
	GBS1813	*rps*G	J	30S ribosomal protein S7
C1668	SAG1771	*rps*L		30S ribosomal protein S12
	SAK1793	*rps*L		30S ribosomal protein S12
	GBS1814	*rps*L		30S ribosomal protein S12
C1669	SAG1772	*pur*R	F	purine operon repressor
	SAK1794	*pur*R	F	purine operon repressor
	GBS1815	-	F	purine operon repressor
C1670	SAG1773	-	R	HD domain protein
	SAK1795	-	R	HD domain protein
	GBS1816	-	R	hypothetical protein
C1671	SAG1774	-	S	hypothetical protein
	SAK1796	-	S	RmuC domain protein
	GBS1817	-	S	hypothetical protein
C1672	SAG1775	-	H	hypothetical protein
	SAK1797	-	H	thiamine pyrophosphokinase
	GBS1818	-	H	hypothetical protein
C1673	SAG1776	*rpe*	G	ribulose-phosphate 3-epimerase
	SAK1758	-	G	D-allulose-6-phosphate 3-epimerase
	SAK1798	*rpe*	G	ribulose-phosphate 3-epimerase
	GBS1819	-	G	ribulose-phosphate 3-epimerase
C1674	SAG1777	-	R	ribosome-associated GTPase
	SAK1799	-	R	ribosome-associated GTPase
	GBS1820	-	R	ribosome-associated GTPase
C1675	SAG1778	-		rRNA (guanine-N1-)-methyltransferase, putative
	SAK1800	-		rRNA (guanine-N1-) methyltransferase (Mycinamicin-resistance protein)
	GBS1821	-		hypothetical protein
C1676	SAG1779	*ksg*A	J	dimethyladenosine transferase
	SAK1801	*ksg*A	J	dimethyladenosine transferase
	GBS1822	*ksg*A	J	dimethyladenosine transferase
C1677	SAG1780	-		hypothetical protein
C1678	SAG1781	-	L	primase-related protein
	SAK1803	-	L	primase-related protein
	GBS1824	-	L	hypothetical protein
C1679	SAG1782	-	L	deoxyribonuclease, TatD family
	SAK1804	-	L	hydrolasease, TatD family
	GBS1825	-	L	hypothetical protein
C1680	SAG1783	-		hypothetical protein
	SAK1805	-		hypothetical protein
	GBS1826	-		hypothetical protein
	GBS1965	-		hypothetical protein
C1681	SAG1784	-		hypothetical protein
	SAK1806	-		hypothetical protein
	GBS1827	-		hypothetical protein
C1682	SAG1785	-		hypothetical protein
	SAK1807	-		hypothetical protein
	GBS1828	-		hypothetical protein
C1683	SAG1786	-		hypothetical protein
	SAK1808	-		hypothetical protein
	SAK1952	-		hypothetical protein
	SAK1957	-		hypothetical protein
	SAK1959	-		hypothetical protein
	GBS1829	-		hypothetical protein
	GBS1980	-		hypothetical protein

(Continue on next page)

List of homolog clusters in the 3 GBS reference genomes (Cont'd)

ID	Locus tag	Gene	COG	Annotation
C1684	SAG1787	*dlt*D	M	dltD protein
	SAK1809	*dlt*D	M	dltD protein
	GBS1830	-	M	hypothetical protein
C1685	SAG1788	*dlt*C	IQ	D-alanine–poly(phosphoribitol) ligase subunit 2
	SAK1810	*dlt*C	IQ	D-alanine–poly(phosphoribitol) ligase subunit 2
	GBS1831	-	IQ	D-alanine–poly(phosphoribitol) ligase subunit 2
C1686	SAG1789	*dlt*B	M	dltB protein
	SAK1811	*dlt*B	M	dltB protein
	GBS1832	-	M	hypothetical protein
C1687	SAG1790	*dlt*A	Q	D-alanine–D-alanyl carrier protein ligase
	SAK1812	*dlt*A	Q	D-alanine–D-alanyl carrier protein ligase
	GBS1833	-	Q	D-alanine–D-alanyl carrier protein ligase
C1688	SAG1791	-	T	sensor histidine kinase
	SAK1813	*dlt*S	T	sensor histidine kinase DltS
	GBS1834	-	T	hypothetical protein
C1689	SAG1792	-	TK	DNA-binding response regulator
	SAK1814	*dltr*	TK	DNA-binding response regulator DltR
	GBS1835	-	TK	hypothetical protein
C1690	SAG1793	*rpm*H		50S ribosomal protein L34
	SAK1815	*rpm*H		50S ribosomal protein L34
	GBS1836	*rpm*H		50S ribosomal protein L34
C1691	SAG1794	-	S	hypothetical protein
	SAK1816	-	S	hypothetical protein
	GBS1837	-	S	hypothetical protein
C1692	SAG1796	-	E	amino acid ABC transporter, permease protein
	SAK1817	*pro*WX	E	glycine betaine/proline ABC transporter, permease/substrate-binding protein
	GBS1838	-	E	hypothetical protein
C1693	SAG1797	-	E	amino acid ABC transporter, ATP-binding protein
	SAK1818	*pro*V	E	glycine betaine/proline ABC transporter, ATP-binding protein
	GBS1839	-	E	hypothetical protein
C1694	SAG1798	-		hypothetical protein
C1695	SAG1799	*xfp*	G	putative phosphoketolase
	SAK1819	*xpk*A	G	putative phosphoketolase
	GBS1840	-	G	putative phosphoketolase
C1696	SAG1800	-	R	hypothetical protein
	SAK1820	-	R	hypothetical protein
	GBS1841	-	R	hypothetical protein
C1697	SAG1801	-	K	transcriptional antiterminator, BglG family
	SAK1762	-	K	PRD domain/PTS system IIA domain protein
	SAK1821	-	K	PRD domain protein
	GBS1842	-	K	hypothetical protein
C1698	SAG1802	-		hypothetical protein
	SAK1822	-		hypothetical protein
	GBS1843	-		hypothetical protein
C1699	SAG1803	-	G	carbohydrate kinase, FGGY family
	SAK1823	-	G	carbohydrate kinase, FGGY family
	GBS1844	-	G	hypothetical protein
C1700	SAG1804	-	R	hypothetical protein
	SAK1824	-	R	amidohydrolase family protein
	GBS1845	-	R	hypothetical protein
C1701	SAG1805	-	G	PTS system, IIC component, putative
	SAK0529	-	G	PTS system, galactitol-specific IIC component
	SAK1825	-	G	PTS system, IIC component, putative
	GBS1846	-	G	hypothetical protein
C1702	SAG1806	-	CHR	glyoxylate reductase, NADH-dependent
	SAK1826	-	CHR	D-isomer specific 2-hydroxyacid dehydrogenase family protein
	GBS1847	-	CHR	hypothetical protein
C1703	SAG1807	-		hypothetical protein
	SAK1827	-		hypothetical protein
	GBS1848	-		hypothetical protein
C1704	SAG1808	-	K	sugar-binding transcriptional regulator, LacI family
	SAK1828	-	K	sugar-binding transcriptional regulator, LacI family
	GBS1849	-	K	hypothetical protein
C1705	SAG1809	-		transaldolase
	SAK1757	-		transaldolase
	SAK1829	-		transaldolase
	GBS1850	-		transaldolase
C1706	SAG1810	-	G	L-ribulose-5-phosphate 4-epimerase
	SAK1830	*ara*D	G	L-ribulose-5-phosphate 4-epimerase
	GBS1851	-	G	L-ribulose-5-phosphate 4-epimerase

(Continue on next page)

List of homolog clusters in the 3 GBS reference genomes (Cont'd)

ID	Locus tag	Gene	COG	Annotation
C1707	SAG1811	-	G	hexulose-6-phosphate isomerase, putative
	SAK1831	-	G	hexulose-6-phosphate isomerase, putative
	GBS1852	-	G	hypothetical protein
C1708	SAG1812	-	G	hexulose-6-phosphate synthase, putative
	SAK1832	-	G	hexulose-6-phosphate synthase, putative
	GBS1853	-	G	hypothetical protein
C1709	SAG1813	-	GT	PTS system, IIA component
	SAK1833	-	GT	PTS system, IIA component
	GBS1854	-	GT	hypothetical protein
C1710	SAG1814	-	G	PTS system, IIB component
	SAK1834	-	G	PTS system, IIB component, lactose/cellobiose family
	GBS1855	-	G	hypothetical protein
C1711	SAG1815	*ula*A		ascorbate-specific PTS system enzyme IIC
	SAK1835	*ula*A		ascorbate-specific PTS system enzyme IIC
	GBS1856	*ula*A		ascorbate-specific PTS system enzyme IIC
C1712	SAG1816	-		hypothetical protein
	SAK1836	-		hypothetical protein
	GBS1857	-		hypothetical protein
C1713	SAG1817	-		hypothetical protein
	SAK1837	-		hypothetical protein
	GBS1858	-		hypothetical protein
C1714	SAG1818	*pur*A		adenylosuccinate synthetase
	SAK1838	*pur*A		adenylosuccinate synthetase
	GBS1859	*pur*A		adenylosuccinate synthetase
C1715	SAG1819	*pfo*R	G	perfringolysin O regulator protein
	SAK1839	*pfo*R	G	pfoR protein
	GBS1860	-	G	hypothetical protein
C1716	SAG1820	-	S	hypothetical protein
	SAK1840	-	S	hypothetical protein
	GBS1861	-	S	hypothetical protein
C1717	SAG1821	-	H	putative glutamate–cysteine ligase
	SAK1841	-	H	putative glutamate–cysteine ligase
	GBS1862	-	M	putative glutamate–cysteine ligase
C1718	SAG1822	-		hypothetical protein
	SAK1842	-		hypothetical protein
	GBS1863	-		hypothetical protein
C1719	SAG1823	-	P	hypothetical protein
	SAK1843	-	P	tellurite resistance protein, putative
	GBS1864	-	P	hypothetical protein
C1720	SAG1824	*hsl*O		Hsp33-like chaperonin
	SAK0774	-		chaperone protein HslO, putative
	SAK1844	*hsl*O		Hsp33-like chaperonin
	GBS0625	-		hypothetical protein
	GBS1865	*hsl*O		Hsp33-like chaperonin
C1721	SAG1825	-	J	NifR3/Smm1 family protein
	SAK1845	-	J	tRNA-dihydrouridine synthase family protein
	GBS1866	-	J	hypothetical protein
C1722	SAG1826	-	F	deoxynucleoside kinase family protein
	SAK1846	-	F	deoxynucleoside kinase family protein
	GBS1867	-	F	hypothetical protein
C1723	SAG1827	*pat*	M	phosphinothricin N-acetyltransferase
	SAK1847	-	M	phosphinothricin N-acetyltransferase, putative
	GBS1868	-	M	hypothetical protein
C1724	SAG1828	-	O	ATP-dependent Clp protease, ATP-binding subunit
	SAK1848	*clp*C	O	ATP-dependent Clp protease, ATP-binding subunit ClpC
	GBS0388	-	O	hypothetical protein
	GBS0718	-	O	hypothetical protein
	GBS0991	-	O	hypothetical protein
	GBS1869	-	O	hypothetical protein
C1725	SAG1829	*cts*R		transcriptional regulator CtsR
	SAK1849	*cts*R		transcriptional regulator CtsR
	GBS1870	*cts*R		hypothetical protein
C1726	SAG1830	-		hypothetical protein
	SAK1850	-		hypothetical protein
	GBS1871	-		hypothetical protein
C1727	SAG1831	*tsf*		elongation factor Ts
	SAK1851	*tsf*		elongation factor Ts
	GBS1872	*tsf*		elongation factor Ts
C1728	SAG1832	*rps*B		30S ribosomal protein S2
	SAK1852	*rps*B		30S ribosomal protein S2
	GBS1873	*rps*B		30S ribosomal protein S2

(Continue on next page)

List of homolog clusters in the 3 GBS reference genomes (Cont'd)

ID	Locus tag	Gene	COG	Annotation
C1729	SAG1833	*ahp*C	O	alkyl hydroperoxide reductase, subunit C
	SAK1853	*ahp*C	O	alkyl hydroperoxide reductase, subunit C
	GBS1874	-	O	hypothetical protein
C1730	SAG1834	*ahp*F	O	alkyl hydroperoxide reductase, subunit F
	SAK1854	*ahp*F	O	NADH dehydrogenase
	GBS1875	-	O	hypothetical protein
C1731	SAG1835	-	S	hypothetical protein
C1732	SAG1836	-	N	hypothetical protein
C1733	SAG1838	-		prophage LambdaSa2, holin, putative
C1734	SAG1839	-		hypothetical protein
C1735	SAG1840	-		hypothetical protein
C1736	SAG1841	-		hypothetical protein
C1737	SAG1842	-	S	prophage LambdaSa2, PblB, putative
	SAK0758	-	S	prophage LambdaSa04, minor structural protein
C1738	SAG1843	-		hypothetical protein
	SAK0757	-	S	prophage LambdaSa04, tail protein, putative
C1739	SAG1844	-	S	hypothetical protein
	SAK0756	-	S	prophage LambdaSa04, tail tape measure protein, TP901 family
C1740	SAG1845	-		hypothetical protein
C1741	SAG1846	-		hypothetical protein
C1742	SAG1847	-		hypothetical protein
C1743	SAG1848	-		hypothetical protein
	SAK0751	-		hypothetical protein
C1744	SAG1849	-		hypothetical protein
C1745	SAG1850	-		hypothetical protein
C1746	SAG1851	-		hypothetical protein
C1747	SAG1852	-		hypothetical protein
C1748	SAG1853	-	R	prophage LambdaSa2, protease, putative
C1749	SAG1854	-		hypothetical protein
C1750	SAG1855	-	R	prophage LambdaSa2, terminase large subunit, putative
	SAK0742	-	R	prophage LambdaSa04, terminase, large subunit
C1751	SAG1856	-		hypothetical protein
C1752	SAG1857	-		prophage LambdaSa2, HNH endonuclease family protein
C1753	SAG1858	-		hypothetical protein
C1754	SAG1859	-	L	prophage LambdaSa2, site-specific recombinase, phage integrase family
C1755	SAG1860	-		hypothetical protein
C1756	SAG1861	-	K	prophage LambdaSa2, transcriptional regulator, Cro/CI family
C1757	SAG1862	-		hypothetical protein
C1758	SAG1864	-		hypothetical protein
C1759	SAG1865	-		hypothetical protein
C1760	SAG1866	-		hypothetical protein
C1761	SAG1867	-		hypothetical protein
C1762	SAG1868	-		hypothetical protein
C1763	SAG1870	-	L	prophage LambdaSa2, DNA replication protein DnaC, putative
C1764	SAG1872	-		hypothetical protein
C1765	SAG1873	*dna*C-1	L	prophage LambdaSa2, replicative DNA helicase
	SAG2139	*dna*C-2	L	replicative DNA helicase
	SAK2097	*dna*C	L	replicative DNA helicase
	GBS2098	*dna*C	L	replicative DNA helicase
C1766	SAG1874	-		hypothetical protein
C1767	SAG1875	-		hypothetical protein
C1768	SAG1876	-		prophage LambdaSa2, HNH endonuclease family protein
C1769	SAG1877	-	K	prophage LambdaSa2, antirepressor protein, putative
	SAK2090	-	K	prophage Sa05, BRO domain protein
C1770	SAG1878	-		hypothetical protein
C1771	SAG1879	-		hypothetical protein
C1772	SAG1880	-		hypothetical protein
C1773	SAG1881	-		hypothetical protein
C1774	SAG1882	-	K	prophage LambdaSa2, repressor protein, putative
C1775	SAG1883	-		hypothetical protein
	SAK0609	-		hypothetical protein
C1776	SAG1884	-		hypothetical protein
C1777	SAG1886	-		hypothetical protein
C1778	SAG1887	-	P	Na+/H+ exchanger family protein
	SAK1856	-	P	monovalent cation:proton antiporter-1 (CPA1) family protein
	GBS1876	-	P	hypothetical protein
C1779	SAG1888	-		hypothetical protein
	SAK1857	-		hypothetical protein
	GBS1877	-		hypothetical protein

(Continue on next page)

List of homolog clusters in the 3 GBS reference genomes (Cont'd)

ID	Locus tag	Gene	COG	Annotation
C1780	SAG1889	-	V	microcin immunity protein MccF, putative
	SAK1858	-	V	peptidase, U61 (muramoyl-tetrapeptide carboxypeptidase) family
	GBS1878	-	V	hypothetical protein
C1781	SAG1890	*pep*O	O	endopeptidase O
	SAK1859	*pep*O	O	endopeptidase O
	GBS1879	-	O	hypothetical protein
C1782	SAG1891	-	R	oxidoreductase, Gfo/Idh/MocA family
	SAK1860	-	R	oxidoreductase, NAD-binding
	GBS1880	-	R	hypothetical protein
C1783	SAG1893	-		hypothetical protein
C1784	SAG1894	-	T	cyclic nucleotide-binding domain protein
	SAK1862	-	T	cyclic nucleotide-binding domain protein
	GBS1882	-	T	hypothetical protein
C1785	SAG1895	*def*		peptide deformylase
	SAK1863	*def*		peptide deformylase
	GBS1883	*def*		peptide deformylase
C1786	SAG1896	*reg*R	K	sugar-binding transcriptional regulator RegR
	SAK1864	-	K	transcriptional regulator, putative
	GBS1884	-	K	hypothetical protein
C1787	SAG1897	-		hypothetical protein
	SAK1865	-		conserved hypothetical protein, truncation
	GBS1885	-		hypothetical protein
C1788	SAG1899	-		PTS system, IIC component
	SAG1949	-		PTS system, IIC component
	SAK1909	-		PTS system, IIC component, mannose/fructose/sorbose family
	GBS1887	-		hypothetical protein
	GBS1937	-		hypothetical protein
C1789	SAG1901	-	R	glucuronyl hydrolase
	GBS1889	-	R	hypothetical protein
C1790	SAG1903	-		hypothetical protein
C1791	SAG1905	-	G	hypothetical protein
	GBS1892	-	G	hypothetical protein
C1792	SAG1908	-		hypothetical protein
	SAK1866	-		hypothetical protein
	GBS1895	-		hypothetical protein
C1793	SAG1909	-	R	nitroreductase family protein
	SAK1867	-	R	nitroreductase family protein
	GBS1896	-	R	hypothetical protein
C1794	SAG1910	-	K	transcriptional regulator, MarR family
	SAK1868	-	K	transcriptional regulator, MarR family
	GBS1897	-	K	hypothetical protein
C1795	SAG1911	-	L	DNA polymerase III subunit alpha
	SAK1324	-	L	exonuclease, DNA polymerase III, epsilon subunit family
	SAK1869	*pol*C	L	DNA polymerase III subunit alpha
	GBS1312	-	L	hypothetical protein
	GBS1898	*pol*C	L	DNA polymerase III subunit alpha
C1796	SAG1912	-	NU	N-acetylmuramoyl-L-alanine amidase, family 4 protein
	SAK0761	-	NU	prophage LambdaSa04, mannosyl-glycoprotein endo-beta-N-acetylglucosamidase family protein
	SAK1870	-	NU	mannosyl-glycoprotein endo-beta-N-acetylglucosamidase, putative
	GBS1899	-	NU	hypothetical protein
C1797	SAG1913	*pro*S	J	prolyl-tRNA synthetase
	SAK1871	*pro*S	J	prolyl-tRNA synthetase
	GBS1900	*pro*S	J	prolyl-tRNA synthetase
C1798	SAG1914	-	M	membrane-associated zinc metalloprotease, putative
	SAK1872	-	M	peptidase, M50A (S2P peptidase) subfamily
	GBS1901	-	M	hypothetical protein
C1799	SAG1915	*cds*A	I	phosphatidate cytidylyltransferase
	SAK1873	*cds*A	I	phosphatidate cytidylyltransferase
	GBS1902	-	I	hypothetical protein
C1800	SAG1916	*upp*S	I	undecaprenyl diphosphate synthase
	SAK1874	*upp*S	I	undecaprenyl diphosphate synthase
	GBS1903	-	I	hypothetical protein
C1801	SAG1917	*yaj*C	U	preprotein translocase subunit YajC
	SAK1875	*yaj*C	U	preprotein translocase subunit YajC
	GBS1904	*yaj*C	U	preprotein translocase subunit YajC
C1802	SAG1918	-		bacteriocin transport accessory protein, putative
	SAK1876	-		hypothetical protein
	GBS1905	-		hypothetical protein

(Continue on next page)

List of homolog clusters in the 3 GBS reference genomes (Cont'd)

ID	Locus tag	Gene	COG	Annotation
C1803	SAG1919	-	C	malate oxidoreductase
	SAK1878	-	C	malate dehydrogenase (decarboxylating)
	GBS1906	-	C	hypothetical protein
C1804	SAG1920	-		citrate carrier protein, CCS family
	SAK1879	-		citrate, cation symporter (CCS) family protein
	GBS1907	-		hypothetical protein
C1805	SAG1921	-	T	sensor histidine kinase
	SAK1880	-	T	sensor histidine kinase, putative
	GBS1908	-	T	hypothetical protein
C1806	SAG1922	-	KT	response regulator
	SAK1881	-	KT	response regulator
	GBS1909	-	KT	hypothetical protein
C1807	SAG1923	*gal*E	M	UDP-glucose 4-epimerase
	SAK0538	*gal*E	M	UDP-glucose 4-epimerase
	SAK1882	*gal*E	M	UDP-glucose 4-epimerase
	GBS1910	-	M	hypothetical protein
C1808	SAG1924	*dex*B		glucan 1,6-alpha-glucosidase
	SAK1883	*dex*B		glucan 1,6-alpha-glucosidase
	GBS1911	-		hypothetical protein
C1809	SAG1925	-	G	sugar ABC transporter, ATP-binding protein
	SAK1884	*msm*K	G	sugar ABC transporter, ATP-binding protein
	GBS1912	-	G	hypothetical protein
C1810	SAG1926	-	TQ	helix-turn-helix domain protein, fis-type
	SAK1885	-	TQ	DNA-binding protein, Fis family
	GBS1913	-	TQ	hypothetical protein
C1811	SAG1927	-	G	lacX protein
	SAK1886	-	G	lactose operon protein LacX
	GBS1328	-	G	hypothetical protein
	GBS1914	-	G	hypothetical protein
C1812	SAG1928	*lac*D	G	tagatose 1,6-diphosphate aldolase
	SAK1887	*lac*D	G	tagatose 1,6-diphosphate aldolase
	GBS1333	-	G	tagatose 1,6-diphosphate aldolase
	GBS1915	-	G	tagatose 1,6-diphosphate aldolase
C1813	SAG1929	*lac*C	G	tagatose-6-phosphate kinase
	SAK1888	*lac*C	G	tagatose-6-phosphate kinase
	GBS1334	-	G	hypothetical protein
	GBS1916	-	G	hypothetical protein
C1814	SAG1930	*lac*B	G	galactose-6-phosphate isomerase
	SAK1889	*lac*B	G	galactose-6-phosphate isomerase
	GBS1335	-	G	galactose-6-phosphate isomerase
	GBS1917	-	G	galactose-6-phosphate isomerase
C1815	SAG1931	*lac*A	G	galactose-6-phosphate isomerase
	SAK1890	*lac*A	G	galactose-6-phosphate isomerase
	GBS1336	-	G	galactose-6-phosphate isomerase
	GBS1918	-	G	galactose-6-phosphate isomerase
C1816	SAG1932	-	G	neuraminidase-related protein
	SAK1891	-	G	sialidase domain protein
	SAK1892	-		hypothetical protein
	GBS1919	-	G	hypothetical protein
C1817	SAG1933	-	G	PTS system, IIC component, putative
	SAK0526	-	G	PTS system, galactitol-specific IIC component, putative
	SAK1893	-	G	PTS system, IIC component, putative
	GBS1920	-	G	hypothetical protein
C1818	SAG1934	-	G	PTS system, IIB component, putative
	SAK1894	-	G	PTS system, IIB component, putative
	GBS1921	-	G	hypothetical protein
C1819	SAG1935	-	GT	PTS system, IIA component, putative
	SAK0524	-	GT	PTS system, galactitol-specific IIA component, putative
	SAK0528	-	GT	PTS system, galactitol-specific IIA component, putative
	SAK1895	-	GT	PTS system, IIA component, putative
	GBS1922	-	GT	hypothetical protein
C1820	SAG1936	*lac*R-2	KG	lactose phosphotransferase system repressor
	SAK1896	-	KG	lactose phosphotransferase system repressor, putative
	GBS1337	-	KG	hypothetical protein
	GBS1923	-	KG	hypothetical protein
C1821	SAG1938	-	P	adhesion lipoprotein
	SAK1898	-	P	laminin-binding surface protein
	GBS1926	-	P	hypothetical protein
C1822	SAG1939	-		D-tyrosyl-tRNA deacylase
	SAK1899	*dtd*		D-tyrosyl-tRNA deacylase
	GBS1927	-		D-tyrosyl-tRNA deacylase

(Continue on next page)

List of homolog clusters in the 3 GBS reference genomes (Cont'd)

ID	Locus tag	Gene	COG	Annotation
C1823	SAG1940	-	TK	GTP pyrophosphokinase family protein
	SAK1900	*rel*A	TK	GTP pyrophosphokinase
	GBS1928	*rel*A	TK	hypothetical protein
C1824	SAG1941	*cpd*B	F	2',3'-cyclic nucleotide 2'-phosphodiesterase/3'-nucleotidase bifunctional periplasmic precursor protein
	SAK1901	*cpd*B	F	2',3'-cyclic nucleotide 2'-phosphodiesterase/3'-nucleotidase bifunctional periplasmic precursor protein
	GBS1929	-	F	2',3'-cyclic nucleotide 2'-phosphodiesterase/3'-nucleotidase bifunctional periplasmic precursor protein
C1825	SAG1942	*nrd*I-2	F	hypothetical protein
	SAK1902	*nrd*I	F	hypothetical protein
	GBS1930	-	F	hypothetical protein
C1826	SAG1943	-	G	hypothetical protein
	SAK1903	-	G	peptidase, M42 (glutamyl aminopeptidase) family
	GBS1931	-	G	hypothetical protein
C1827	SAG1944	-	F	hypothetical protein
	SAK1904	-	F	hypothetical protein
	GBS1932	-		hypothetical protein
C1828	SAG1945	-	P	iron ABC transporter, iron-binding protein
	SAK1905	-	P	ABC transporter, substrate-binding protein
	GBS1933	-	P	hypothetical protein
C1829	SAG1946	-	T	DNA-binding response regulator
	SAK1906	-	T	DNA-binding response regulator, AraC family
	GBS1934	-	T	hypothetical protein
C1830	SAG1947	-	T	hypothetical protein
	SAK1907	-	T	sensor histidine kinase, putative
	GBS1935	-	T	hypothetical protein
C1831	SAG1948	-		PTS system, IID component
	SAK1908	-		PTS system, IID component, mannose/fructose/sorbose family
	GBS1936	-		hypothetical protein
C1832	SAG1950	-		PTS system, IIB component
	SAK1910	-		PTS system, IIB component, mannose/fructose/sorbose family
	GBS1938	-		hypothetical protein
C1833	SAG1951	-	G	PTS system, IIA component, putative
	SAK1911	-	G	PTS system, IIA component, mannose/fructose/sorbose family
	GBS1939	-	G	hypothetical protein
C1834	SAG1952	-		hypothetical protein
	SAK1912	-		hypothetical protein
	GBS1940	-		hypothetical protein
C1835	SAG1953	-		hypothetical protein
C1836	SAG1954	-		hypothetical protein
	SAK1913	-		hypothetical protein
	SAK1914	-		hypothetical protein
	GBS1941	-		hypothetical protein
C1837	SAG1955	-	V	ABC transporter, ATP-binding protein
	SAG1980	-	V	ABC transporter, ATP-binding protein
	SAG2035	-	V	ABC transporter, ATP-binding protein
	SAK1033	-	V	ABC transporter, ATP-binding protein
	SAK1975	-	V	ABC transporter, ATP-binding protein
	GBS0927	-	V	hypothetical protein
	GBS1942	-	V	hypothetical protein
	GBS1993	-	V	hypothetical protein
C1838	SAG1957	-	KT	response regulator
	SAK1918	-	KT	response regulator
	GBS1944	-	KT	hypothetical protein
C1839	SAG1958	-	R	hypothetical protein
	SAK1919	*rgf*B	R	RgfB protein
	GBS1945	-	R	hypothetical protein
C1840	SAG1959	-	G	PTS system, IIABC components
	SAK1920	-	G	PTS system, glucose-specific IIABC component, putative
	GBS1946	-	G	hypothetical protein
C1841	SAG1960	-	T	sensor histidine kinase
	SAK1921	-	T	sensor histidine kinase
	GBS1947	-	T	hypothetical protein
C1842	SAG1961	*pho*B	TK	phosphate regulon response regulator PhoB
	SAK1922	-	TK	phosphate regulon transcriptinal regulatory protein PhoB, putative
	GBS1948	-	TK	hypothetical protein
C1843	SAG1962	-	P	phosphate transport system regulatory protein PhoU, putative
	SAK1923	*pho*U	P	phosphate transport system regulatory protein PhoU
	GBS1949	-	P	hypothetical protein

(Continue on next page)

List of homolog clusters in the 3 GBS reference genomes (Cont'd)

ID	Locus tag	Gene	COG	Annotation
C1844	SAG1963	-	P	phosphate ABC transporter, ATP-binding protein
	SAK1924	*pst*B	P	phosphate ABC transporter, ATP-binding protein
	GBS1950	-	P	hypothetical protein
C1845	SAG1964	-	P	phosphate ABC transporter, permease protein
	SAK1925	*pst*A	P	phosphate ABC transporter, permease protein PtsA
	GBS1951	-	P	hypothetical protein
C1846	SAG1965	-	P	phosphate ABC transporter, permease protein
	SAK1926	*pst*C	P	phosphate ABC transporter, permease protein PstC
	GBS1952	-	P	hypothetical protein
C1847	SAG1966	-	P	hemolysin precursor, putative
	SAK1927	-	P	phosphate ABC transporter, phosphate-binding protein, putative
	GBS1953	-	P	hypothetical protein
C1848	SAG1967	-		hypothetical protein
	SAK1928	-	F	hypothetical protein
	GBS1954	-		hypothetical protein
C1849	SAG1968	-	S	conserved hypothetical protein TIGR00046
	SAK1929	-	S	conserved hypothetical protein TIGR00046
	GBS1955	-	S	hypothetical protein
C1850	SAG1969	*prm*A	J	ribosomal protein L11 methyltransferase
	SAK1930	*prm*A	J	ribosomal protein L11 methyltransferase
	GBS1956	-	J	ribosomal protein L11 methyltransferase
C1851	SAG1970	-	S	hypothetical protein
	SAK1931	-	S	hypothetical protein
	GBS1957	-	S	hypothetical protein
C1852	SAG1971	-		hypothetical protein
C1853	SAG1972	-	K	transcriptional regulator, MerR family
	SAK1932	-	K	transcriptional regulator, MerR family
	GBS1958	-	K	hypothetical protein
C1854	SAG1973	-		acetyltransferase, GNAT family
	SAK1933	-		acetyltransferase, GNAT family
	GBS1959	-		hypothetical protein
C1855	SAG1974	-	LR	MutT/nudix family protein
	SAK1934	-	LR	hydrolase, NUDIX family
	GBS1960	-	LR	hypothetical protein
C1856	SAG1975	-		hypothetical protein
	SAK1935	-	S	hypothetical protein
	GBS1961	-		hypothetical protein
C1857	SAG1976	-		hypothetical protein
	SAK1936	-		hypothetical protein
	GBS1962	-		hypothetical protein
C1858	SAG1977	-		acetyltransferase, GNAT family
	SAK1937	-		acetyltransferase, GNAT family
	GBS1963	-		hypothetical protein
C1859	SAG1978	-	L	ATPase, AAA family
	SAK1938	-	L	ATPase, AAA family
	GBS1964	-	L	hypothetical protein
C1860	SAG1979	-		hypothetical protein
	SAG2034	-		hypothetical protein
	SAK1974	-		hypothetical protein
	GBS1992	-		hypothetical protein
C1861	SAG1981	-		hypothetical protein
C1862	SAG1982	-	K	transcriptional regulator, Cro/CI family
	SAG2037	-	K	transcriptional regulator, Cro/CI family
	SAK1977	-	K	DNA-binding protein
	GBS1994	-	K	hypothetical protein
C1863	SAG1983	-	R	hypothetical protein
	SAG2039	-	R	hypothetical protein
	SAK1979	-	R	MagZ family protein
	GBS1996	-	R	hypothetical protein
C1864	SAG1984	-	R	conserved hypothetical protein TIGR00730
	SAG2040	-	R	conserved hypothetical protein TIGR00730
	SAK1980	-	R	decarboxylase family protein
	GBS1997	-	R	hypothetical protein
C1865	SAG1985	-		hypothetical protein
	SAK1942	-		hypothetical protein
C1866	SAG1987	-		hypothetical protein
	SAK1944	-		hypothetical protein
C1867	SAG1989	-		hypothetical protein
	SAG2115	-		hypothetical protein
	SAK1946	-		hypothetical protein

(Continue on next page)

List of homolog clusters in the 3 GBS reference genomes (Cont'd)

ID	Locus tag	Gene	COG	Annotation
C1868	SAG1990	-		hypothetical protein
	SAK1947	-		hypothetical protein
	GBS1971	-		hypothetical protein
C1869	SAG1991	-	K	transcriptional regulator, Cro/CI family
C1870	SAG1992	-		hypothetical protein
C1871	SAG1994	-		hypothetical protein
	GBS0481	-		hypothetical protein
C1872	SAG1995	-		hypothetical protein
C1873	SAG1996	-		cell wall surface anchor family protein, putative
	GBS0392	-		hypothetical protein
	GBS0479	-		hypothetical protein
	GBS0722	-		hypothetical protein
	GBS0987	-		hypothetical protein
	GBS1144	-		hypothetical protein
C1874	SAG1997	-		hypothetical protein
	GBS0478	-		hypothetical protein
C1875	SAG1998	-		hypothetical protein
	GBS0404	-	NU	hypothetical protein
	GBS0477	-		hypothetical protein
	GBS0734	-	NU	hypothetical protein
	GBS0975	-	NU	hypothetical protein
C1876	SAG1999	-		hypothetical protein
C1877	SAG2000	-		hypothetical protein
	GBS0476	-		hypothetical protein
C1878	SAG2001	-		conjugal transfer protein, interruption-C
C1879	SAG2005	-		hypothetical protein
C1880	SAG2006	-		hypothetical protein
C1881	SAG2007	-		hypothetical protein
C1882	SAG2008	-	S	hypothetical protein
C1883	SAG2009	-	D	hypothetical protein
C1884	SAG2010	-		hypothetical protein
C1885	SAG2011	-		hypothetical protein
C1886	SAG2012	-		hypothetical protein
C1887	SAG2013	-		hypothetical protein
C1888	SAG2014	-	R	hypothetical protein
C1889	SAG2015	-		transcriptional regulator, Cro/CI family
C1890	SAG2016	-		hypothetical protein
C1891	SAG2017	-	L	transcriptional regulator, Cro/CI family
C1892	SAG2018	-	D	FtsK/SpoIIIE family protein
	GBS0241	-	D	hypothetical protein
C1893	SAG2019	-		hypothetical protein
C1894	SAG2020	-		hypothetical protein
C1895	SAG2021	-	M	cell wall surface anchor family protein
	GBS0393	-		hypothetical protein
	GBS0723	-		hypothetical protein
	GBS0986	-		hypothetical protein
	GBS1143	-		hypothetical protein
C1896	SAG2022	-	L	transposase, ISL3 family
C1897	SAG2026	-		hypothetical protein
	SAK1965	-		hypothetical protein
	GBS1985	-		hypothetical protein
C1898	SAG2027	-	V	ABC transporter, ATP-binding protein
	SAK1966	-	R	ABC transporter, ATP-binding protein
	GBS1986	-	V	hypothetical protein
C1899	SAG2028	-		hypothetical protein
C1900	SAG2029	-		streptomycin resistance protein
	SAK1967	-		aminoglycoside 6-adenylyltransferase, putative
	GBS1987	-		hypothetical protein
C1901	SAG2030	-		hypothetical protein
	SAK1968	-		hypothetical protein
	GBS1988	-		hypothetical protein
C1902	SAG2031	-		hypothetical protein
	SAK1971	-		hypothetical protein
	GBS1989	-		hypothetical protein
C1903	SAG2032	-	K	hypothetical protein
	SAK1972	-	K	transcriptional regulator, PadR family
	GBS1990	-	K	hypothetical protein
C1904	SAG2033	-		acetyltransferase, GNAT family
	SAK1973	-		acetyltransferase, GNAT family
	GBS1991	-		hypothetical protein

(Continue on next page)

List of homolog clusters in the 3 GBS reference genomes (Cont'd)

ID	Locus tag	Gene	COG	Annotation
C1905	SAG2036	-		hypothetical protein
	SAK1976	-		hypothetical protein
C1906	SAG2038	-		PAP2 family protein
	SAK1978	-		PAP2 family protein
	GBS1995	-		hypothetical protein
C1907	SAG2041	-	R	protease, putative
	SAK1981	-		CAAX amino terminal protease family protein
	GBS1998	-	R	hypothetical protein
C1908	SAG2042	-	P	rhodanese-like domain protein
	SAK1982	-	P	rhodanese-like domain protein
	GBS1999	-	P	hypothetical protein
C1909	SAG2043	*cfb*		cAMP factor
	SAK1983	*cfb*		CAMP factor
	GBS2000	-		CAMP factor
C1910	SAG2044	-		hypothetical protein
C1911	SAG2045	-	F	topology modulation protein
	SAK1984	-	F	topology modulation protein
	GBS2001	-	F	topology modulation protein
C1912	SAG2046	-	C	glycerol dehydrogenase, putative
	SAK1985	-	C	glycerol dehydrogenase, putative
	GBS2002	-	C	hypothetical protein
C1913	SAG2047	-	S	hypothetical protein
	SAK1986	-	S	hypothetical protein
	GBS2003	-	S	hypothetical protein
C1914	SAG2048	-	E	bifunctional homocysteine S-methyltransferase/5,10-methylenetetrahydrofolate reductase protein
	SAK1987	-	E	bifunctional homocysteine S-methyltransferase/5,10-methylenetetrahydrofolate reductase protein
	GBS2004	-	E	bifunctional homocysteine S-methyltransferase/5,10-methylenetetrahydrofolate reductase protein
C1915	SAG2049	*met*E	E	5-methyltetrahydropteroyltriglutamate–homocysteine methyltransferase
	SAK1988	*met*E	E	5-methyltetrahydropteroyltriglutamate–homocysteine methyltransferase
	GBS2005	-	E	5-methyltetrahydropteroyltriglutamate–homocysteine methyltransferase
C1916	SAG2050	-	S	hypothetical protein
	SAK1989	-	S	azaleucine resistance protein AzlD, putative
	GBS2006	-	S	hypothetical protein
C1917	SAG2051	-	E	branched-chain amino acid transport protein AzlC, putative
	SAK1990	-	E	azaleucine resistance protein AzlC, putative
	GBS2007	-	E	hypothetical protein
C1918	SAG2052	-		hypothetical protein
C1919	SAG2054	-	TK	DNA-binding response regulator
	SAK1992	-	TK	DNA-binding response regulator
	GBS2009	-	TK	similar to two-component response regulator
C1920	SAG2055	-	T	sensor histidine kinase
	SAK1993	-	T	sensor histidine kinase
	GBS2010	-	T	hypothetical protein
C1921	SAG2056	-		chromosome assembly-related protein
	SAK1994	-		hypothetical protein
	GBS2011	-		hypothetical protein
C1922	SAG2057	*leu*S	J	leucyl-tRNA synthetase
	SAK1995	*leu*S	J	leucyl-tRNA synthetase
	GBS2012	*leu*S	J	leucyl-tRNA synthetase
C1923	SAG2058	-		major facilitator family protein
	SAK1996	-		major facilitator family protein
	GBS2013	-		hypothetical protein
C1924	SAG2059	-	R	hypothetical protein
	SAK1997	-	R	patatin-like phospholipase family protein
	GBS2014	-	R	hypothetical protein
C1925	SAG2060	-	M	glycosyl transferase, family 8
	SAK1489	-	M	glycosyl transferase, family 8
	GBS1525	-	M	hypothetical protein
	GBS2015	-	M	hypothetical protein
C1926	SAG2062	*nus*G	K	transcription antitermination protein NusG
	SAK1998	*nus*G	K	transcription antitermination protein NusG
	GBS2017	*nus*G	K	transcription antitermination protein NusG
C1927	SAG2063	-		pathogenicity protein, putative
	SAK2002	-		pathogenicity protein, putative, interuption N-terminus
	GBS2018	-		putative peptidoglycan linked protein
C1928	SAG2064	-	U	translocase
	SAK2003	*sec*E	U	translocase
	GBS2019	-	U	translocase

(Continue on next page)

List of homolog clusters in the 3 GBS reference genomes (Cont'd)

ID	Locus tag	Gene	COG	Annotation
C1929	SAG2065	*rpm*G	J	50S ribosomal protein L33
C1930	SAG2066	*pbp*2A	M	penicillin-binding protein 2A
	SAK2005	*pbp*2A	M	penicillin-binding protein 2A
	GBS2020	-	M	hypothetical protein
C1931	SAG2067	-	J	ribosomal large subunit pseudouridine synthase, RluD subfamily
	SAK2006	-	J	ribosomal large subunit pseudouridine synthase, RluA family
	GBS2021	-	J	hypothetical protein
C1932	SAG2068	-	S	hypothetical protein
	SAK2007	-	S	hypothetical protein
	GBS2022	-	S	hypothetical protein
C1933	SAG2070	*deo*C	F	deoxyribose-phosphate aldolase
	SAK2009	*deo*C	F	deoxyribose-phosphate aldolase
	GBS2024	-	F	hypothetical protein
C1934	SAG2071	-		Na+ dependent nucleoside transporter
	SAK2010	-		concentrative nucleoside transporter (CNT) family protein
	GBS2025	-		hypothetical protein
C1935	SAG2072	*udp*	F	uridine phosphorylase
	SAK2011	*udp*	F	uridine phosphorylase
	GBS2026	-	F	hypothetical protein
C1936	SAG2073	-	K	transcriptional regulator, GntR family
	SAK2012	-	K	transcriptional regulator, GntR family
	GBS2027	-	K	hypothetical protein
C1937	SAG2074	*gro*EL	O	chaperonin GroEL
	SAK2013	*gro*EL	O	chaperonin GroEL
	GBS2029	*gro*EL	O	chaperonin GroEL
C1938	SAG2075	*gro*ES	O	co-chaperonin GroES
	SAK2014	*gro*ES	O	co-chaperonin GroES
	GBS2030	*gro*ES	O	co-chaperonin GroES
C1939	SAG2076	-	R	ABC transporter, ATP-binding protein
	SAK2015	-	R	ABC transporter, ATP-binding protein
	GBS2031	-	R	hypothetical protein
C1940	SAG2077	-	R	ABC transporter, permease protein
	SAK2016	-	R	ABC transporter, permease protein
	GBS2032	-	R	hypothetical protein
C1941	SAG2078	-	R	protein of unknown function/lipoprotein, putative
	SAK2017	-	R	ABC transporter, substrate-binding protein
	GBS2033	-	R	hypothetical protein
C1942	SAG2079	-	R	hydrolase, haloacid dehalogenase-like family
	SAK2018	-	R	Cof-like hydrolase family protein
	GBS2034	-	R	hypothetical protein
C1943	SAG2080	-	R	glyoxalase family protein
	SAK2019	-	R	glyoxalase family protein
	GBS2035	-	R	hypothetical protein
C1944	SAG2081	-	S	hypothetical protein
	SAK2020	-	S	hypothetical protein
	GBS2036	-	S	hypothetical protein
C1945	SAG2082	*nrd*G	O	anaerobic ribonucleoside-triphosphate reductase activating protein
	SAK2021	*nrd*G	O	anaerobic ribonucleoside-triphosphate reductase activating protein
	GBS2037	-	O	hypothetical protein
C1946	SAG2083	-	R	acetyltransferase, GNAT family
	SAK2022	-	R	acetyltransferase, GNAT family
	GBS2038	-	R	hypothetical protein
C1947	SAG2084	-	R	virulence factor MviM, putative
	SAK2023	-	R	oxidoreductase, Gfo/Idh/MocA family
	GBS2039	-	R	hypothetical protein
C1948	SAG2085	-		hypothetical protein
	SAK2024	-		hypothetical protein
	GBS2040	-		hypothetical protein
C1949	SAG2086	*nrd*D	F	anaerobic ribonucleoside triphosphate reductase
	SAK1140	-	F	ATP cone domain protein
	SAK2025	*nrd*D	F	anaerobic ribonucleoside triphosphate reductase
	GBS1085	-	F	hypothetical protein
	GBS2041	-	F	anaerobic ribonucleoside triphosphate reductase
C1950	SAG2087	-		hypothetical protein
	SAK2026	-		hypothetical protein
	GBS2042	-		hypothetical protein
C1951	SAG2088	-		hypothetical protein
	SAK2027	-		hypothetical protein
C1952	SAG2089	-	S	hypothetical protein
	SAK2028	-	S	hypothetical protein
	GBS2043	-	S	hypothetical protein

(Continue on next page)

List of homolog clusters in the 3 GBS reference genomes (Cont'd)

ID	Locus tag	Gene	COG	Annotation
C1953	SAG2090	-		Holliday junction resolvase-like protein
	SAK2029	-		Holliday junction resolvase-like protein
	GBS2044	-		Holliday junction resolvase-like protein
C1954	SAG2091	-		hypothetical protein
	SAK2030	-		hypothetical protein
	GBS2045	-		hypothetical protein
C1955	SAG2092	*spx*A	P	transcriptional regulator Spx
	SAK2031	*spx*A	P	transcriptional regulator Spx
	GBS2046	*spx*A	P	transcriptional regulator Spx
C1956	SAG2093	*rec*A	L	recombinase A
	SAK2032	*rec*A	L	recombinase A
	GBS2047	*rec*A	L	recombinase A
C1957	SAG2095	*tag*		DNA-3-methyladenine glycosylase I
	SAK2034	*tag*		DNA-3-methyladenine glycosylase I
	GBS2049	-		hypothetical protein
C1958	SAG2096	*ruv*A	L	Holliday junction DNA helicase motor protein
	SAK2035	*ruv*A	L	Holliday junction DNA helicase motor protein
	GBS2050	*ruv*A	L	Holliday junction DNA helicase motor protein
C1959	SAG2097	-		transporter, putative
	SAK2036	-		major facilitator family transporter
	GBS2051	-		hypothetical protein
C1960	SAG2098	*hex*B	L	DNA mismatch repair protein
	SAK2037	*hex*B	L	DNA mismatch repair protein
	GBS2052	*mut*L	L	DNA mismatch repair protein
C1961	SAG2099	-		hypothetical protein
C1962	SAG2100	-		cold shock protein, CSD family
	SAK2039	-		cold shock protein
	GBS2053	-		hypothetical protein
C1963	SAG2101	*hex*A	L	DNA mismatch repair protein
	SAK2040	*hex*A	L	DNA mismatch repair protein
	GBS2054	*mut*S	L	DNA mismatch repair protein
C1964	SAG2102	-	K	arginine repressor ArgR, putative
	SAK2041	*arg*R	K	arginine repressor
	GBS2055	-	K	hypothetical protein
C1965	SAG2103	*arg*S	J	arginyl-tRNA synthetase
	SAK2042	*arg*S	J	arginyl-tRNA synthetase
	GBS2056	*arg*S	J	arginyl-tRNA synthetase
C1966	SAG2104	-		hypothetical protein
	SAK2043	-		hypothetical protein
	GBS2057	-		hypothetical protein
C1967	SAG2105	-	S	hypothetical protein
	SAK2044	-	S	hypothetical protein
	GBS2058	-	S	hypothetical protein
C1968	SAG2106	-	S	hypothetical protein
	SAK2045	-	S	hypothetical protein
	GBS2059	-	S	hypothetical protein
C1969	SAG2107	*asp*S	J	aspartyl-tRNA synthetase
	SAK2046	*asp*S	J	aspartyl-tRNA synthetase
	GBS2060	*asp*S	J	aspartyl-tRNA synthetase
C1970	SAG2108	*his*S	J	histidyl-tRNA synthetase
	SAK2047	*his*S	J	histidyl-tRNA synthetase
	GBS2061	*his*S	J	histidyl-tRNA synthetase
C1971	SAG2109	*rpm*F	J	50S ribosomal protein L32
	SAK2048	*rpm*F	J	50S ribosomal protein L32
	GBS2062	*rpm*F	J	50S ribosomal protein L32
C1972	SAG2110	*rpm*G-3	J	50S ribosomal protein L33
	SAK2049	*rpm*G3	J	50S ribosomal protein L33
	GBS2063	*rpm*G	J	50S ribosomal protein L33
C1973	SAG2113	-		hypothetical protein
C1974	SAG2116	-		hypothetical protein
C1975	SAG2117	-		hypothetical protein
C1976	SAG2118	-	K	transcriptional regulator, Cro/CI family
C1977	SAG2119	-	S	hypothetical protein
C1978	SAG2120	-		hypothetical protein
C1979	SAG2121	-	S	hypothetical protein
	SAK2060	-	S	peptidase propeptide and YPEB domain protein
	GBS2080	-	S	hypothetical protein
C1980	SAG2122	-	TK	DNA-binding response regulator
	SAK2061	-	TK	DNA-binding response regulator
	GBS2081	-	TK	hypothetical protein

(Continue on next page)

List of homolog clusters in the 3 GBS reference genomes (Cont'd)

ID	Locus tag	Gene	COG	Annotation
C1981	SAG2123	-	T	sensor histidine kinase
	SAK2062	-	T	sensor histidine kinase
	GBS2082	-	T	hypothetical protein
C1982	SAG2124	-	S	hypothetical protein
	SAK2063	-	S	hypothetical protein
	GBS2083	-	S	hypothetical protein
C1983	SAG2125	*arc*C-1	E	carbamate kinase
	SAK2064	*arc*C	E	carbamate kinase
	GBS2084	-	E	carbamate kinase
C1984	SAG2126	*arg*F-1	E	ornithine carbamoyltransferase
	SAK2065	*arg*F	E	ornithine carbamoyltransferase
	GBS2085	-	E	ornithine carbamoyltransferase
C1985	SAG2127	-	T	sensor histidine kinase
	SAK2066	-	T	sensor histidine kinase, putative
	GBS2086	-	T	hypothetical protein
C1986	SAG2128	-	T	response regulator
	SAK2067	-	T	response regulator
	GBS2087	-	T	hypothetical protein
C1987	SAG2129	-	E	amino acid ABC transporter, ATP-binding protein
	SAK2068	-	E	glycine betaine/carnitine/choline ABC transporter, ATP-binding protein
	GBS2088	-	E	hypothetical protein
C1988	SAG2130	-	M	amino acid ABC transporter, amino acid-binding protein/permease protein
	SAK2069	-	M	glycine betaine/carnitine/choline ABC transporter, permease/substrate-binding protein
	GBS2089	-	M	hypothetical protein
C1989	SAG2131	-	S	hypothetical protein
	SAK2070	-	S	FmtC protein, putative
	GBS2090	-	S	hypothetical protein
C1990	SAG2132	-	R	hypothetical protein
	SAK2071	-	R	hypothetical protein
	GBS2091	-	R	hypothetical protein
C1991	SAG2133	-	S	hypothetical protein
	SAK2072	-	S	hypothetical protein
	GBS2092	-	S	hypothetical protein
C1992	SAG2134	-	S	hypothetical protein
	SAK2073	-	S	prophage Sa05, membrane protein, putative
	GBS2093	-	S	hypothetical protein
C1993	SAG2135	-	K	transcriptional regulator, TetR family, putative
	SAK2074	-	K	prophage Sa05, transcriptional regulator, putative
	GBS2094	-	K	hypothetical protein
C1994	SAG2136	-		hypothetical protein
	SAK2075	-		hypothetical protein
	GBS2095	-		hypothetical protein
C1995	SAG2137	*rps*D	J	30S ribosomal protein S4
	SAK2095	*rps*D	J	30S ribosomal protein S4
	GBS2096	*rps*D	J	30S ribosomal protein S4
C1996	SAG2138	-		hypothetical protein
	SAK2096	-		hypothetical protein
	GBS2097	-		hypothetical protein
C1997	SAG2140	*rpl*I	J	50S ribosomal protein L9
	SAK2098	*rpl*I	J	50S ribosomal protein L9
	GBS2099	*rpl*I	J	50S ribosomal protein L9
C1998	SAG2141	-	T	DHH family protein
	SAK2099	-	T	DHH family protein
	GBS2100	-	T	hypothetical protein
C1999	SAG2142	*gid*A	D	glucose-inhibited division protein A
	SAK2100	*gid*A	D	glucose-inhibited division protein A
	GBS2101	-	D	glucose-inhibited division protein A
C2000	SAG2143	-	U	hypothetical protein
	SAK2101	-	U	membrane protein, MarC family
	GBS2102	-	U	hypothetical protein
C2001	SAG2144	*trm*U	J	tRNA (5-methylaminomethyl-2-thiouridylate)-methyltransferase
	SAK2102	*trm*U	J	tRNA (5-methylaminomethyl-2-thiouridylate)-methyltransferase
	GBS2103	-	J	tRNA (5-methylaminomethyl-2-thiouridylate)-methyltransferase
C2002	SAG2145	*sdh*B	E	L-serine dehydratase, iron-sulfur-dependent, beta subunit
	SAK2103	*sdh*B	E	L-serine dehydratase, iron-sulfur-dependent, beta subunit
	GBS2104	-	E	hypothetical protein
C2003	SAG2146	*sdh*A	E	L-serine dehydratase, iron-sulfur-dependent, alpha subunit
	SAK2104	*sdh*A	E	L-serine dehydratase, iron-sulfur-dependent, alpha subunit
	GBS2105	-	E	hypothetical protein

(Continue on next page)

List of homolog clusters in the 3 GBS reference genomes (Cont'd)

ID	Locus tag	Gene	COG	Annotation
C2004	SAG2147	-		protein of unknown function/lipoprotein, putative
	SAK2105	-		transglycosylase-like domain protein
	GBS2106	-		hypothetical protein
C2005	SAG2148	-		LysM domain protein
	SAK0762	-	M	prophage LambdaSa04, LysM domain protein
	SAK2106	-		LysM domain protein
	GBS2107	-		hypothetical protein
C2006	SAG2149	-	P	cobalt transport family protein
	SAK2107	-	P	cobalt ABC transporter, permease protein
	GBS2108	-	P	hypothetical protein
C2007	SAG2150	-	P	ABC transporter, ATP-binding protein
	SAK2108	-	P	cobalt ATP transporter, putative, ATP-binding protein
	GBS2109	-	P	hypothetical protein
C2008	SAG2151	-	P	ABC transporter, ATP-binding protein
	SAK2109	-	P	cobalt ABC transporter, putative, ATP-binding protein
	GBS2110	-	P	hypothetical protein
C2009	SAG2152	*pgs*A	I	CDP-diacylglycerol–glycerol-3-phosphate 3-phosphatidyltransferase
	SAK2110	*pgs*A	I	CDP-diacylglycerol–glycerol-3-phosphate 3-phosphatidyltransferase
	GBS2111	*pgs*A	I	hypothetical protein
C2010	SAG2153	-	R	peptidase, M16 family
	SAK2111	-	R	peptidase, M16C (eupitrilysin) subfamily
	GBS2112	-	R	hypothetical protein
C2011	SAG2154	-	R	hypothetical protein
	SAK2112	-	R	peptidase M16 inactive domain protein
	GBS2113	-	R	hypothetical protein
C2012	SAG2155	-	S	hypothetical protein
	SAK2113	-	S	hypothetical protein
	GBS2114	-	S	hypothetical protein
C2013	SAG2156	*rec*F	L	recombination protein F
	SAK2114	*rec*F	L	recombination protein F
	GBS2115	*rec*F	L	recombination protein F
C2014	SAG2157	-	G	transporter, putative
	SAK2115	-	G	glucose uptake protein
	GBS2116	-	G	hypothetical protein
C2015	SAG2158	-		transcriptional regulator, Cro/CI family
	SAK2116	-		DNA-binding protein
	GBS2117	-		hypothetical protein
C2016	SAG2159	*gua*B	F	inositol-5-monophosphate dehydrogenase
	SAK2117	*gua*B	R	inositol-5-monophosphate dehydrogenase
	GBS2118	-	F	inositol-5-monophosphate dehydrogenase
C2017	SAG2160	-	K	transcriptional regulator, ArgR family
	SAK2118	-	K	arginine repressor, putative
	GBS2119	-	K	hypothetical protein
C2018	SAG2161	-	T	transcriptional regulator, Crp/Fnr family
	SAK2119	-	T	transcriptional regulator, Crp/Fnr family
	GBS2120	-	T	hypothetical protein
C2019	SAG2162	-	S	hypothetical protein
	SAK2120	-	S	B3/4 domain protein
	GBS2121	-	S	hypothetical protein
C2020	SAG2163	*arc*A	E	arginine deiminase
	SAK2121	*arc*A	E	arginine deiminase
	GBS2122	-	E	arginine deiminase
C2021	SAG2164	-		acetyltransferase, GNAT family
	SAK2122	-		acetyltransferase, GNAT family
	GBS2123	-		hypothetical protein
C2022	SAG2165	*arg*F-2	E	ornithine carbamoyltransferase
	SAK2123	*arg*F	E	ornithine carbamoyltransferase
	GBS2124	-	E	ornithine carbamoyltransferase
C2023	SAG2166	*arc*D	E	arginine/ornithine antiporter
	SAK2124	-	E	arginine/ornithine antiporter
	GBS2125	-	E	hypothetical protein
C2024	SAG2167	*arc*C-2	E	carbamate kinase
	SAK2125	*arc*C	E	carbamate kinase
	GBS2126	-	E	carbamate kinase
C2025	SAG2168	*trp*S	J	tryptophanyl-tRNA synthetase
	SAK2126	*trp*S	J	tryptophanyl-tRNA synthetase
	GBS2127	*trp*S	J	tryptophanyl-tRNA synthetase
C2026	SAG2169	-		hypothetical protein
	SAK2127	-		hypothetical protein
	GBS2128	-		hypothetical protein

(Continue on next page)

List of homolog clusters in the 3 GBS reference genomes (Cont'd)

ID	Locus tag	Gene	COG	Annotation
C2027	SAG2170	-	S	hypothetical protein
	SAK2128	-	S	conserved hypothetical protein FRAMESHIFT
	GBS2129	-	S	hypothetical protein
C2028	SAG2171	-	R	ABC transporter, ATP-binding protein
	SAK2129	-	R	ABC transporter, ATP-binding protein
	GBS2130	-	R	hypothetical protein
C2029	SAG2172	-		ABC transporter, permease protein, putative
	SAK2130	-		hypothetical protein
	GBS2131	-		hypothetical protein
C2030	SAG2173	-	S	hypothetical protein
	SAK2134	-	S	hypothetical protein
	GBS2132	-	S	hypothetical protein
C2031	SAG2174	-	O	serine protease
	SAK2135	*htr*A	O	serine peptidase HtrA
	GBS2133	-	O	hypothetical protein
C2032	SAG2175	-	K	partitioning protein, ParB family
	SAK2136	-	K	chromosome partition protein, ParB family
	GBS2134	-	K	hypothetical protein
C2033	SAK0185	-		hypothetical protein
C2034	SAK0228	-	U	competence protein, putative
	GBS0161	-	U	hypothetical protein
C2035	SAK0229	-		competence protein, putative
	GBS0162	-	NU	hypothetical protein
C2036	SAK0230	-		hypothetical protein
	GBS0163	-	NU	hypothetical protein
C2037	SAK0275	-		hypothetical protein
C2038	SAK0279	-		DNA-binding protein
	GBS0212	-		hypothetical protein
C2039	SAK0320	-	L	ISSag8, transposase
C2040	SAK0520	-	S	hypothetical protein
	GBS0484	-	S	hypothetical protein
C2041	SAK0521	-	S	hypothetical protein
	GBS0485	-	S	hypothetical protein
C2042	SAK0522	-	QR	hypothetical protein
	GBS0486	-	QR	hypothetical protein
C2043	SAK0525	-	G	PTS system, galactitol-specific IIB component, putative
C2044	SAK0527	*rha*D	G	rhamnulose-1-phosphate aldolase
C2045	SAK0530	-	G	PTS system, galactitol-specific IIB component, putative
C2046	SAK0535	-	G	alpha-galactosidase, putative
C2047	SAK0537	*gal*T	G	galactose-1-phosphate uridylyltransferase
C2048	SAK0539	-	G	aldose 1-epimerase, interruption-N
C2049	SAK0542	-	G	aldose 1-epimerase, interruption-C
C2050	SAK0546	-		hypothetical protein
C2051	SAK0548	-		hypothetical protein
	GBS0493	-		hypothetical protein
C2052	SAK0557	-	F	Ser/Thr protein phosphatase family protein
	GBS0503	-	F	hypothetical protein
C2053	SAK0564	-	L	ISSag8, transposase
C2054	SAK0610	-		prophage LambdaSa03, transcriptional regulator, Cro/CI family
C2055	SAK0611	-	K	prophage LambdaSa03, transcriptional regulator, Cro/CI family
C2056	SAK0612	-		hypothetical protein
C2057	SAK0613	-		hypothetical protein
C2058	SAK0614	-		hypothetical protein
C2059	SAK0615	-		hypothetical protein
C2060	SAK0616	-		conserved hypothetical protein TIGR01618
C2061	SAK0617	-	KL	prophage LambdaSa03, helicase, putative
C2062	SAK0618	-		hypothetical protein
C2063	SAK0619	-		conserved hypothetical protein/bacteriophage resistance protein
C2064	SAK0620	-		hypothetical protein
C2065	SAK0621	-		hypothetical protein
C2066	SAK0622	-		hypothetical protein
C2067	SAK0624	-		hypothetical protein
C2068	SAK0626	-		hypothetical protein
C2069	SAK0627	-		hypothetical protein
C2070	SAK0630	-	V	prophage LambdaSa03, HNH endonuclease family protein
C2071	SAK0634	-		hypothetical protein
C2072	SAK0650	-		hypothetical protein
C2073	SAK0651	-		hypothetical protein
C2074	SAK0652	-		prophage LambdaSa03, holin, phi LC3 family
C2075	SAK0671	-	L	ISSag8, transposase

(Continue on next page)

List of homolog clusters in the 3 GBS reference genomes (Cont'd)

ID	Locus tag	Gene	COG	Annotation
C2076	SAK0720	-		hypothetical protein
C2077	SAK0723	-		hypothetical protein
C2078	SAK0724	-		hypothetical protein
C2079	SAK0725	-	L	prophage LambdaSa04, DNA polymerase
C2080	SAK0726	-		hypothetical protein
C2081	SAK0727	-		hypothetical protein
C2082	SAK0728	-		hypothetical protein
C2083	SAK0729	-		hypothetical protein
C2084	SAK0730	-	R	prophage LambdaSa04, DNA primase, P4 family
C2085	SAK0731	-		hypothetical protein
C2086	SAK0732	-		hypothetical protein
C2087	SAK0733	-	KL	prophage LambdaSa04, helicase, SNF2 family
C2088	SAK0734	-		hypothetical protein
C2089	SAK0737	-	V	prophage LambdaSa04, HNH endonuclease family protein
C2090	SAK0738	-	K	prophage LambdaSa04, DNA methylase
	GBS1120	-	K	hypothetical protein
C2091	SAK0740	-		hypothetical protein
C2092	SAK0741	-	L	prophage LambdaSa04, terminase, small subunit, P27 family
C2093	SAK0744	-	R	prophage LambdaSa04, RelE/ParE family protein
C2094	SAK0745	-		hypothetical protein
C2095	SAK0746	-	S	prophage LambdaSa04, portal protein, HK97 family
C2096	SAK0748	-	R	prophage LambdaSa04, major capsid protein, HK97 family
C2097	SAK0749	-		conserved hypothetical protein TIGR01560
C2098	SAK0750	-	R	prophage LambdaSa04, head-tail adaptor, putative
C2099	SAK0752	-		hypothetical protein
C2100	SAK0753	-		prophage LambdaSa04, major tail protein, phi13 family
C2101	SAK0754	-		hypothetical protein
C2102	SAK0755	-		hypothetical protein
C2103	SAK0760	-		prophage LambdaSa04, holin
C2104	SAK0763	-	L	prophage LambdaSa04, site-specific recombinase, resolvase family
C2105	SAK0764	-	L	prophage LambdaSa04, site-specific recombinase, resolvase family
C2106	SAK0765	-		hypothetical protein
C2107	SAK0770	-		hypothetical protein
	GBS0617	-		hypothetical protein
C2108	SAK0941	-	L	ISSag8, transposase
C2109	SAK1032	-		hypothetical protein
	GBS0926	-		hypothetical protein
C2110	SAK1067	-		hypothetical protein
	GBS0958	-		hypothetical protein
C2111	SAK1113	-		hypothetical protein
C2112	SAK1114	-		hypothetical protein
C2113	SAK1117	-		hypothetical protein
C2114	SAK1119	-		hypothetical protein
C2115	SAK1121	-		hypothetical protein
C2116	SAK1255	*cps*H		capsular polysaccharide synthesis protein CpsH
	GBS1240	*cps*I		capsular polysaccharide repeating-unit polymerase
C2117	SAK1300	-		hypothetical protein
C2118	SAK1303	-	ER	hypothetical protein
	GBS1289	-	ER	hypothetical protein
C2119	SAK1307	-	C	glycerophosphoryl diester phosphodiesterase family protein
	GBS1293	-	C	hypothetical protein
C2120	SAK1327	-		plasmid replication protein
	GBS1316	-		hypothetical protein
C2121	GBS1321	-		hypothetical protein
C2122	SAK1331	-		hypothetical protein
	GBS1322	-		hypothetical protein
C2123	SAK1332	-		hypothetical protein
	GBS1323	-		hypothetical protein
C2124	SAK1333	-		type IIG restriction enzyme and methyltransferase
	GBS1324	-		hypothetical protein
C2125	SAK1344	-		hypothetical protein
C2126	SAK1437	-	M	sortase family protein, putative
C2127	SAK1438	-		hypothetical protein
C2128	SAK1440	-		surface protein Spb1
C2129	SAK1441	-	M	cna B-type domain protein
C2130	SAK1442	-		hypothetical protein
C2131	SAK1444	-		hypothetical protein
C2132	SAK1479	-	LR	hydrolase, NUDIX family
	GBS1514	-	LR	hypothetical protein

(Continue on next page)

List of homolog clusters in the 3 GBS reference genomes (Cont'd)

ID	Locus tag	Gene	COG	Annotation
C2133	SAK1754	*hsd*M	V	type I restriction-modification system, M subunit
C2134	SAK1755	-		hypothetical protein
C2135	SAK1766	-		hypothetical protein
C2136	SAK1767	-	R	phenazine biosynthesis protein, PhzF family
C2137	SAK1768	-	J	acetyltransferase, GNAT family, FRAMESHIFT
C2138	SAK1769	-		hypothetical protein
C2139	SAK1802	-	FGR	HIT family protein
	GBS1823	-	FGR	hypothetical protein
C2140	SAK1877	-	L	ISSag8, transposase
C2141	SAK1916	-	V	ABC transporter, ATP-binding protein, truncation
C2142	SAK1917	*rgf*C	T	histidine kinase
	GBS1943	-	T	hypothetical protein
C2143	SAK1940	-		hypothetical protein
C2144	SAK1941	-		hypothetical protein
C2145	SAK1948	-	K	transcriptional regulator, putative
	GBS1972	-	K	hypothetical protein
C2146	SAK1949	-	K	hypothetical protein
	GBS1975	-	K	hypothetical protein
C2147	SAK1950	-	R	DJ-1/PfpI family protein
C2148	SAK1954	-		hypothetical protein
	SAK1963	-		hypothetical protein
	GBS1968	-		hypothetical protein
	GBS1976	-		hypothetical protein
	GBS1983	-		hypothetical protein
C2149	SAK1955	-		hypothetical protein
	GBS1978	-		hypothetical protein
C2150	SAK1958	-		hypothetical protein
C2151	SAK1960	-		hypothetical protein
C2152	SAK1961	-		hypothetical protein
	SAK1962	-		hypothetical protein
	GBS1981	-		hypothetical protein
C2153	SAK1964	-		hypothetical protein
	GBS1984	-		hypothetical protein
C2154	SAK2033	*cin*A	R	competence damage-inducible protein A
	GBS2048	-	R	competence damage-inducible protein A
C2155	SAK2038	-		hypothetical protein
C2156	SAK2050	-		IS10R, transposase
C2157	SAK2053	-		hypothetical protein
	GBS2066	-		hypothetical protein
C2158	SAK2054	-		hypothetical protein
	GBS2067	-		hypothetical protein
C2159	SAK1330	-		hypothetical protein
	SAK2055	-		hypothetical protein
	GBS2068	-		hypothetical protein
C2160	SAK2057	-		hypothetical protein
	GBS2070	-		hypothetical protein
C2161	SAK2058	-		plasmid replication protein
	GBS2071	-		hypothetical protein
C2162	SAK2076	-		hypothetical protein
C2163	SAK2077	-	S	hypothetical protein
C2164	SAK2078	-		hypothetical protein
C2165	SAK2079	-		prophage Sa05, transcriptional regulator, ArpU family
C2166	SAK2080	-		hypothetical protein
C2167	SAK2081	-		hypothetical protein
C2168	SAK2082	-		hypothetical protein
C2169	SAK2083	-	R	prophage Sa05, DNA primase, P4 family
C2170	SAK2084	-		prophage Sa05, DNA replication protein, putative
C2171	SAK2085	-		hypothetical protein
C2172	SAK2086	-		hypothetical protein
C2173	SAK2087	-		hypothetical protein
C2174	SAK2088	-		prophage Sa05, transcriptional regulator, CopG family
C2175	SAK2089	-	S	prophage Sa05, BRO domain protein
C2176	SAK2091	-		prophage Sa05, DNA-binding protein
C2177	SAK2092	-		hypothetical protein
C2178	SAK2093	-		hypothetical protein
C2179	GBS0045	-		hypothetical protein
C2180	GBS0046	-		hypothetical protein
C2181	GBS0207	-	K	hypothetical protein
C2182	GBS0236	-		hypothetical protein
C2183	GBS0238	-		hypothetical protein

(Continue on next page)

List of homolog clusters in the 3 GBS reference genomes (Cont'd)

ID	Locus tag	Gene	COG	Annotation
C2184	GBS0239	-		hypothetical protein
C2185	GBS0240	-		hypothetical protein
C2186	GBS0242	-		hypothetical protein
C2187	GBS0243	-		hypothetical protein
C2188	GBS0244	-		hypothetical protein
C2189	GBS0361	-		hypothetical protein
C2190	GBS0362	-		hypothetical protein
C2191	GBS0363	-		hypothetical protein
C2192	GBS0364	-		hypothetical protein
C2193	GBS0365	-		hypothetical protein
C2194	GBS0366	-		hypothetical protein
C2195	GBS0367	-		hypothetical protein
C2196	GBS0369	-		hypothetical protein
C2197	GBS0370	-		hypothetical protein
C2198	GBS0371	-		hypothetical protein
C2199	GBS0372	-		hypothetical protein
C2200	GBS0373	-		hypothetical protein
C2201	GBS0374	-		hypothetical protein
C2202	GBS0375	-		hypothetical protein
C2203	GBS0376	-		hypothetical protein
C2204	GBS0377	-		hypothetical protein
C2205	GBS0378	-		hypothetical protein
C2206	GBS0379	-		hypothetical protein
C2207	GBS0380	-		hypothetical protein
C2208	GBS0381	-		hypothetical protein
C2209	GBS0383	-		hypothetical protein
C2210	GBS0384	-		hypothetical protein
C2211	GBS0385	-		hypothetical protein
C2212	GBS0389	-		hypothetical protein
C2213	GBS0390	-		hypothetical protein
C2214	GBS0394	-	L	hypothetical protein
C2215	GBS0395	-		hypothetical protein
C2216	GBS0396	-	U	hypothetical protein
C2217	GBS0397	-		hypothetical protein
C2218	GBS0398	-		hypothetical protein
C2219	GBS0399	-	L	hypothetical protein
C2220	GBS0400	-		hypothetical protein
C2221	GBS0401	-		hypothetical protein
C2222	GBS0402	-		hypothetical protein
C2223	GBS0403	-		hypothetical protein
C2224	GBS0405	-		hypothetical protein
C2225	GBS0406	-	D	hypothetical protein
C2226	GBS0407	-		hypothetical protein
C2227	GBS0409	-		hypothetical protein
C2228	GBS0474	-		hypothetical protein
C2229	GBS0480	-		hypothetical protein
C2230	GBS0638	-		hypothetical protein
C2231	GBS0658	-		hypothetical protein
C2232	GBS0692	-		hypothetical protein
C2233	GBS0693	-		hypothetical protein
C2234	GBS0694	-		hypothetical protein
C2235	GBS0695	-		hypothetical protein
C2236	GBS0696	-		hypothetical protein
C2237	GBS0697	-		hypothetical protein
C2238	GBS0698	-		hypothetical protein
C2239	GBS0699	-		hypothetical protein
C2240	GBS0700	-		hypothetical protein
C2241	GBS0701	-		hypothetical protein
C2242	GBS0702	-		hypothetical protein
C2243	GBS0703	-		hypothetical protein
C2244	GBS0704	-		hypothetical protein
C2245	GBS0705	-		hypothetical protein
C2246	GBS0706	-		hypothetical protein
C2247	GBS0707	-		hypothetical protein
C2248	GBS0708	-		hypothetical protein
C2249	GBS0709	-		hypothetical protein
C2250	GBS0710	-		hypothetical protein
C2251	GBS0711	-		hypothetical protein
C2252	GBS0713	-		hypothetical protein

(Continue on next page)

List of homolog clusters in the 3 GBS reference genomes (Cont'd)

ID	Locus tag	Gene	COG	Annotation
C2253	GBS0714	-		hypothetical protein
C2254	GBS0715	-		hypothetical protein
C2255	GBS0719	-		hypothetical protein
C2256	GBS0720	-		hypothetical protein
C2257	GBS0724	-	L	hypothetical protein
C2258	GBS0725	-		hypothetical protein
C2259	GBS0726	-	U	hypothetical protein
C2260	GBS0727	-		hypothetical protein
C2261	GBS0728	-		hypothetical protein
C2262	GBS0729	-	L	hypothetical protein
C2263	GBS0730	-		hypothetical protein
C2264	GBS0731	-		hypothetical protein
C2265	GBS0732	-		hypothetical protein
C2266	GBS0733	-		hypothetical protein
C2267	GBS0735	-		hypothetical protein
C2268	GBS0736	-	D	hypothetical protein
C2269	GBS0737	-		hypothetical protein
C2270	GBS0739	-		hypothetical protein
C2271	GBS0915	-		hypothetical protein
C2272	GBS0970	-		hypothetical protein
C2273	GBS0972	-		hypothetical protein
C2274	GBS0973	-	D	hypothetical protein
C2275	GBS0974	-		hypothetical protein
C2276	GBS0976	-		hypothetical protein
C2277	GBS0977	-		hypothetical protein
C2278	GBS0978	-		hypothetical protein
C2279	GBS0979	-		hypothetical protein
C2280	GBS0980	-	L	hypothetical protein
C2281	GBS0981	-		hypothetical protein
C2282	GBS0982	-		hypothetical protein
C2283	GBS0983	-	U	hypothetical protein
C2284	GBS0984	-		hypothetical protein
C2285	GBS0985	-	L	hypothetical protein
C2286	GBS0989	-		hypothetical protein
C2287	GBS0990	-		hypothetical protein
C2288	GBS0994	-		hypothetical protein
C2289	GBS0995	-		hypothetical protein
C2290	GBS0996	-		hypothetical protein
C2291	GBS0998	-		hypothetical protein
C2292	GBS0999	-		hypothetical protein
C2293	GBS1000	-		hypothetical protein
C2294	GBS1001	-		hypothetical protein
C2295	GBS1002	-		hypothetical protein
C2296	GBS1003	-		hypothetical protein
C2297	GBS1004	-		hypothetical protein
C2298	GBS1005	-		hypothetical protein
C2299	GBS1006	-		hypothetical protein
C2300	GBS1007	-		hypothetical protein
C2301	GBS1008	-		hypothetical protein
C2302	GBS1009	-		hypothetical protein
C2303	GBS0368	-		hypothetical protein
C2304	GBS1010	-		hypothetical protein
C2305	GBS1011	-		hypothetical protein
C2306	GBS1012	-		hypothetical protein
C2307	GBS1013	-		hypothetical protein
C2308	GBS1014	-		hypothetical protein
C2309	GBS1015	-		hypothetical protein
C2310	GBS1016	-		hypothetical protein
C2311	GBS1061	-		hypothetical protein
C2312	GBS1119	-		hypothetical protein
C2313	GBS1122	-		hypothetical protein
C2314	GBS1123	-		hypothetical protein
C2315	GBS1124	-		hypothetical protein
C2316	GBS1125	-		hypothetical protein
C2317	GBS1127	-		hypothetical protein
C2318	GBS1128	-	U	hypothetical protein
C2319	GBS1129	-		hypothetical protein
C2320	GBS1130	-		hypothetical protein
C2321	GBS1131	-		hypothetical protein

(Continue on next page)

List of homolog clusters in the 3 GBS reference genomes (Cont'd)

ID	Locus tag	Gene	COG	Annotation
C2322	GBS1134	-		hypothetical protein
C2323	GBS1136	-		hypothetical protein
C2324	GBS1137	-		hypothetical protein
C2325	GBS1138	-		hypothetical protein
C2326	GBS1139	-		hypothetical protein
C2327	GBS1140	-		hypothetical protein
C2328	GBS1141	-		hypothetical protein
C2329	GBS1142	-		hypothetical protein
C2330	GBS1146	-		hypothetical protein
C2331	GBS1147	-	K	hypothetical protein
C2332	GBS1148	-		hypothetical protein
C2333	GBS1150	-		hypothetical protein
C2334	GBS1151	-		hypothetical protein
C2335	GBS1152	-		hypothetical protein
C2336	GBS1153	-		hypothetical protein
C2337	GBS1217	-		hypothetical protein
C2338	GBS1218	-		hypothetical protein
C2339	GBS1219	-		hypothetical protein
C2340	GBS1220	-		hypothetical protein
C2341	GBS1222	-		hypothetical protein
C2342	GBS1223	-	V	hypothetical protein
C2343	GBS1286	-		hypothetical protein
C2344	GBS1318	-		hypothetical protein
C2345	GBS1341	-	L	hypothetical protein
C2346	GBS1342	-	L	hypothetical protein
C2347	GBS1343	-		hypothetical protein
C2348	GBS1344	-	K	hypothetical protein
C2349	GBS1361	-		hypothetical protein
C2350	GBS1364	-	U	hypothetical protein
C2351	GBS1371	-		hypothetical protein
C2352	GBS1598	-		hypothetical protein
C2353	GBS1599	-		hypothetical protein
C2354	GBS1621	*ser*C	HE	phosphoserine aminotransferase
C2355	GBS1740	-	V	hypothetical protein
C2356	GBS1743	-	P	hypothetical protein
C2357	GBS1744	-		hypothetical protein
C2358	GBS1745	-		hypothetical protein
C2359	GBS1746	-		hypothetical protein
C2360	GBS1748	-	Q	hypothetical protein
C2361	GBS1749	-	K	hypothetical protein
C2362	GBS1970	-		hypothetical protein
C2363	GBS1973	-		hypothetical protein
C2364	GBS1974	-		hypothetical protein
C2365	GBS1977	-		hypothetical protein
C2366	GBS2028	-		hypothetical protein
C2367	GBS2072	-		hypothetical protein
C2368	GBS2074	-		hypothetical protein
C2369	GBS2076	-		hypothetical protein
C2370	GBS2077	-		hypothetical protein

(End of Table)
