# Supplementary material for: Computational Bacterial Genome-Wide Analysis of Phylogenetic Profiles Reveals Potential Virulence Genes of Streptococcus agalactiae
Source: PLoS One. 2011 Apr 4;6(4):e17964. doi: 10.1371/journal.pone.0017964 (PMC3070697; doi:10.1371/journal.pone.0017964)

**Supporting Information - Text S1: Prioritization of candidate virulence genes in the GBS genomes by using all known virulence factors as training set**

In addition to the fifteen individual analyses by functional categories, identical prioritization method was applied to rank all 6134 genes in three GBS genomes using all 134 virulence genes as the training set. We observed that the highly-prioritized genes were generally skewed towards training set with higher number of training genes (Table S2.1). Out of the top-30 genes, the list of highly-ranked genes were skewed towards *cyl* (6 genes out of 30 were found in the *cyl*-only training set), *cps* (4), and *neu* (4) gene clusters, all of which contains have more genes represented in the training set compared to others (*cyl*: 36 training genes, *cps*: 37 genes, *neu:* 12 genes, see Table 1).

One exception to this trend was the *lmb* gene rank, where 4 out of top-30 genes appeared in the individual rank of *lmb* gene which contained only one ortholog. Within the top-30, there were 7 genes with this aggregated training set that did not appear in individual gene ranks, including genes encoding cytoplasmic alpha-amylase (C0675), CAAX amino terminal protease family protein (C1907), *lac*X (C1811), a putative phosphoketolase (C1695) and several hypothetical proteins (C1463, C1176, C1557).

We did not include the results in our primary discussion, not only because the results were likely to be skewed towards more frequently represented functions in the training set, but also because the selection of these genes did not meet our criteria of the definition of a functional category. While it is acknowledged that some of these genes may contribute to GBS virulence, the heterogeneity of genes implies an inconsistency in their molecular functional roles, a disparity that is discordant to our original postulate such that their phylogenetic profiles may not have a high degree of similarity, which are likely to affect the robustness of the results.

**Table Text-S2.1. Top-30 genes prioritised by inductive CGP using all known GBS virulence factors as training set**

| Rank | Cluster | Score | Gene product / Annotation | Gene was also ranked within the top-10 of individual functional rank(s) |
| --- | --- | --- | --- | --- |
| 1 | C1367/M | 0.9865 | glycosyltransferase, group 1 family protein | C-antigens, *cps, neu* |
| 2 | C0359 | 0.9798 | transcriptional regulator, putative | *cps* |
| 3 | C0675 | 0.9769 | cytoplasmic alpha-amylase | (none) |
| 4 | C0342 | 0.9750 | [*fab*Z] (3R)-hydroxymyristoyl ACP dehydratase | *cyl* |
| 5 | C1907 | 0.9728 | CAAX amino terminal protease family protein | (none) |
| 6 | C1985/T | 0.9711 | sensor histidine kinase, putative | *neu* |
| 7 | C0156/M | 0.9682 | penicillin-binding protein 1B, putative | *pbp*1A |
| 8 | C1330/M | 0.9677 | glycosyl transferase, group 1 family protein | *cps, neu* |
| 9 | C0520/RP | 0.9652 | [*adc*A] ABC transporter, Zn-binding adhesion lipoprotein | *lmb* |
| 10 | C1445/P | 0.9639 | [*mts*A] manganese ABC transporter, manganese-binding adhesion liprotein | *lmb* |
| 11 | C1463 | 0.9635 | hypothetical protein (SAG1555, GBS1609, SAK1574) | (none) |
| 12 | C0340/IQ | 0.9631 | [*fab*F] 3-oxoacyl-(acyl carrier protein) synthase | *cyl* |
| 13 | C1260 | 0.9631 | peptide deformylase | *scp*B |
| 14 | C1211/KL | 0.9628 | SNF2 family protein | *scp*B, pilli, *csp*A |
| 15 | C0339/IQR | 0.9620 | [*fab*G] 3-ketoacyl-(acyl-carrier-protein) reductase | *cyl* |
| 16 | C0508/IQR | 0.9618 | acetoin reductase | *cyl* |
| 17 | C1821/P | 0.9617 | laminin-binding surface protein | *lmb* |
| 18 | C1811/G | 0.9604 | lactose operon protein LacX | (none) |
| 19 | C1443/P | 0.9582 | [*mts*C] manganese ABC transporter, permease protein | *lmb* |
| 20 | C1860 | 0.9567 | hypothetical protein | *cyl* |
| 21 | C1816/G | 0.9558 | neuraminidase-related protein | *hyl*B |
| 22 | C1231/E | 0.9555 | [*mmu*M] homocysteine methyltransferase | *cps* |
| 23 | C1927 | 0.9543 | pathogenicity protein, putative | *fbs*B |
| 24 | C1176 | 0.9535 | hypothetical protein (SAG1227, GBS1299, SAK1313) | (none) |
| 25 | C1194/K | 0.9505 | [*cad*C] cadmium resistance accessory protein CadX | *pav*A, *scp*B, *csp*A |
| 26 | C1695 | 0.9502 | putative phosphoketolase | (none) |
| 27 | C2124 | 0.9500 | type IIG restriction enzyme and methyltransferase | *fbs*A, *neu* |
| 28 | C0222/D | 0.9493 | [*pre*] plasmid recombination enzyme | *fbs*B |
| 29 | C1044/R | 0.9492 | oxidoreductase, short chain dehydrogenase family | *cyl* |
| 30 | C1557/G | 0.9490 | hypothetical protein (SAG1654, GBS1698, SAK_1666) | (none) |

Number of genes occurred in the individual functional ranks: *cyl* gene cluster (6), *cps* gene cluster (4), *neu* gene cluster (4), *lmb* gene (4), *scp*B gene (3), *csp*A gene (2), GBS minor pilin cluster (1), *hyl*B gene (1), *fbs*A gene (1), *fbs*B gene (1), *pav*A gene (1), *pbp*1A gene(1), C-antigens (1), *cfb*(0), and spb1(0).


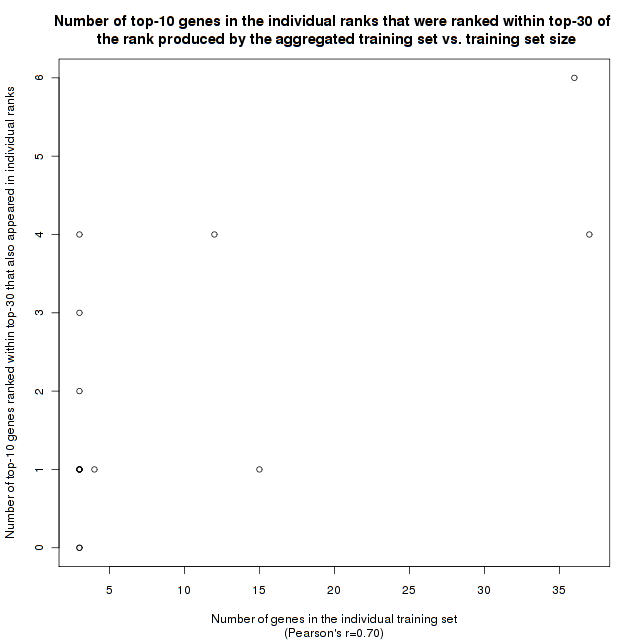

Supplement: Text S1 — Prioritization of candidate virulence genes in the GBS genomes by using all known virulence factors as training set. (DOC) [file pone.0017964.s004.doc]
