# Supplementary material for: Computational Bacterial Genome-Wide Analysis of Phylogenetic Profiles Reveals Potential Virulence Genes of Streptococcus agalactiae
Source: PLoS One. 2011 Apr 4;6(4):e17964. doi: 10.1371/journal.pone.0017964 (PMC3070697; doi:10.1371/journal.pone.0017964)
Supplement: Text S2 — Correlations between prioritized gene lists produced by different machine learning algorithms. (DOC) [file pone.0017964.s005.doc]

**Supporting Information - Text S2. Correlations between prioritized gene lists produced by different machine learning algorithms**

**Purpose**

While the observation of a good AUC in the rediscovery experiments supported the use phylogenetic profiles to rank virulence genes, it remained unknown how well the gene ranks would agree with each other. To address this question, we compared the overall virulence gene rank produced by four machine learning algorithms.

**Methods**

We analysed the correlation between 4 machine learning algorithms by using the *de novo* gene lists from all 6214 genes in three GBS genomes. Pairwise comparisons of the rank fractions (position of the gene in the genome, 0 = top of the rank, 1 = bottom) were performed on each gene for each algorithm combinations. The correlations between the ranks were measured by Pearson's correlation coefficient *r*.

**Results**

The correlations between different gene ranks produced by individual algorithms were medium (0.44-0.65), with the support vector machines achieved the highest *r* (0.65). The results are shown in Figure S1.


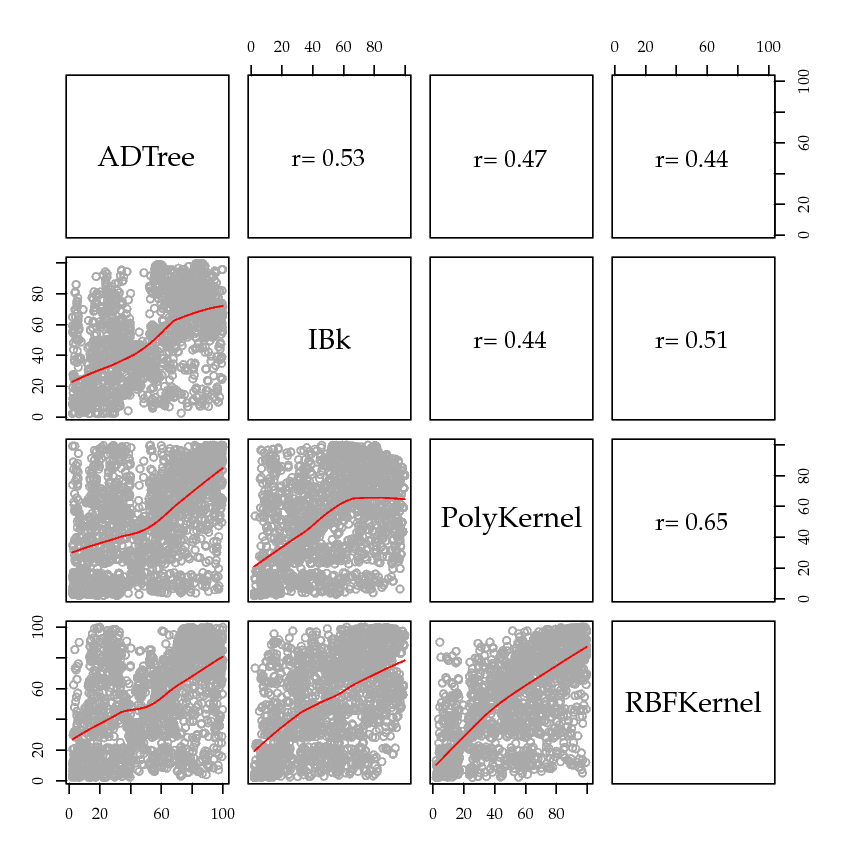


**Figure S3.1.** Correlation between individual ranks produced by ADTree, IBk, SVM/Poly, and SVM/RBF. The numbers on both axes indicate the rank fraction as percentiles from the top of the rank. Genes in the training sets are excluded from the analysis.

**Interpretations**

While individual performance of classifiers were comparable in the rediscovery experiment of virulence using the rank produced by all virulence genes, the agreements between individual ranks are much poorer. In effect, the results in rediscovery experiment 2 showed that the true virulence genes were ranked very highly by individual algorithms in rediscovery experiments, but the order of their appearance in the individual lists were less consistent. Given the overall AUC is good as demonstrated by rediscovery experiment 2, the "correct" virulence genes are expected to be ranked much higher than non-virulence genes by most algorithms. Combining individual ranks produced by each algorithm is therefore a justified approach to enhance the gene ranks.
