# Supplementary material for: Computational Bacterial Genome-Wide Analysis of Phylogenetic Profiles Reveals Potential Virulence Genes of Streptococcus agalactiae
Source: PLoS One. 2011 Apr 4;6(4):e17964. doi: 10.1371/journal.pone.0017964 (PMC3070697; doi:10.1371/journal.pone.0017964)
Supplement: Text S3 — Additional materials and methods. (DOC) [file pone.0017964.s006.doc]

# Supporting Information – Text S3: Additional material and methods

**Selection of virulence category**

For each of the known virulence GBS factors, literature review was performed and the location of each gene were identified and labeled in the reference genomes. The criteria for grouping of the known virulence factors into 15 functional categories was based on being discriminable by BLAST at the critical E-value, and their contribution to a distinguishable biological mechanism in GBS pathogenesis. Many GBS virulence genes are currently known, including genes encoding adhesin proteins (*fbs*A and *fbs*B: fibrinogen binding proteins [S1-5], *pav*A, fibrotic-binding protein [S6]; *scp*B, streptococcal C5a peptidase which is also capable of binding to fibronectin [S7,8]; *lmb,* a laminin-binding protein [S9-11]; and genes encoding a pilus gene cluster [S12-14]); invasins (*bca*, *rib*, and *alp*1-5: α-like family proteins [S15-19]; *cyl* cluster: β-haemolysins/cytolysins which may also trigger aberrant immune responses [S20-26]; *cfb*: streptococcal CAMP factor [S27]; *hyl*B: hyaluronate lyase [S28-30]; and *spb*1 [S27,31]), as well as many genes encoding proteins that contribute to the ability to evade host immune system (*bac*: C-β protein/surface antigen [S32-34]; *cps* and *neu* gene clusters, responsible for biosynthesis of terminally-sialylated polysaccharide capsule [S35-41]; *csp*A: a serine protease [S42]; and *pbp*1A, penicillin binding protein 1A [S43-45]).

## Rediscovery of the training genes

For each functional GBS gene category containing *n* virulence genes, a leave-one-out (*n*-fold) cross-validation was performed, with the remaining candidate genes assigned a negative class. Rediscovery performance was measured by AUC for each combination of algorithm and gene category. All genes in NEM6 genome were used as candidate genes for the first rediscovery experiment, and all genes from the 3 reference genomes were applied in the second experiment.

## Sub-sampling of negative examples in the *de novo* discovery of GBS virulence genes

In the rediscovery experiments, all remaining genes were labeled as negative by assuming they are not related to GBS virulence. In contrast, the selection of negative examples is less trivial in the *de novo* discovery of virulence genes. Specifically, some of the genes labeled negative in the rediscovery experiment may be genuinely related to GBS virulence and are thus interesting candidates for discovery. Since it was impossible to determine *a priori* which of these genes are not virulence-related, we are likely to construct a highly-biased training set that is strongly skewed towards the negative group, if the machine learning models are trained with a large proportion of true virulence genes that are falsely labeled as negative.

One approach to ameliorate such bias is to perform multiple classifications with smaller sets of negative examples that are randomly sampled. The aim of random sub-sampling is to create some training sets consisting of smaller proportion of false negative genes. While keeping the positive genes (the known virulence genes) constant in the training set, the candidate virulence genes are thus more likely to be revealed in some training sets containing few false-negatives. Such genes are expected to be discovered more readily instead of being erroneously classified as negative with a biased aggregated training set. In supporting the use of the sub-sampling method, Terabe *et al.* previously demonstrated that the use of sub-sampling does not produce inferior performance when compared with an aggregated sample set [S46].

The reduction of this bias by using the sub-sampling approach can also be proven:

*Proof.* Let *A =* the number of positive gene examples in the training set (*A* > 0), *B =* the number of unknown gene examples in the test set (*B* > 0), *p =* the proportion of positive genes in B falsely labeled negative, and *k =* the proportion of B sub-sampled. Define *f*(*k*) *= kBp* / (*A+kB*) *=* the proportion of unknown genes mislabeled as negative in the training set. It can be shown that:


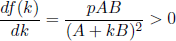


for all values of 0<*k*<1, indicating that *f*(*k*) decreases with a decreasing value of *k*. That is, the proportion of mislabeled training set is smaller with smaller values of *k*. QED.

Each inductive CGP model was trained only with a subset of the unknown genes as negatives. For each gene or gene group, all virulence genes in the group were labeled as positive gene examples. The remaining 3/4 of candidate genes were randomly sampled without replacement and were assigned a negative class (*k*=0.75). This value of *k* was selected because while, in theory, the proportion of mislabeled gene is smallest when *k* approaches 0, the machine learning algorithms still require sufficient number negative examples to achieve sufficient discriminatory power of virulence genes. Predictions were made on the remaining one-quarter of the unknown genes and scores from each run were obtained for each gene to be predicted. The above procedure was repeated for 1000 runs for each of the 15 gene categories to improve coverage. Scores from each run were averaged by arithmetic means which formed the basis of ranking.

## Combining the ranks from multiple models

While the AUC from each of the ranks were very high in both rediscovery experiments, the agreements between different algorithms are poor. It is, however, not possible to distinguish which model performs better, as each algorithm provides a different "view" of the phylogenetic profile. Nevertheless, it is expected that a relevant co-occurring virulence gene should be ranked highly regardless the algorithms chosen. To increase the likelihood of identifying the true virulence genes, we aggregated the ranks produced by 4 machine learning algorithms into a final rank by using the following voting function such that:


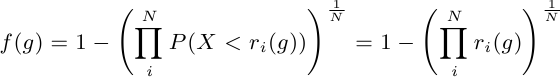


where *g* is the candidate gene, *f*(*g*) is the final aggregated score of gene *g*, X is an uniform random variable, and *r_i_* is the rank fraction (position of the rank, start from 1, divided by the total number of genes in the entire list) of rank *i*.

Compared with an additive model, using a multiplicative model is considered more desirable because genes that were ranked very highly in individual prioritized lists will appear more favorably in the overall aggregated rank.

## Clustering of homologous genes

Because more than one GBS genome was used as candidate genes, homologous (including both orthologs and closely-related paralogs) genes would appear multiple times in close proximity in a prioritized rank due to high degrees of sequence similarities. To allow better visualization of results, the prioritized results were collated by grouping the genes into homolog clusters, with the primary intention of aggregating orthologs, based on the reciprocal best BLAST hit method described by Hirsh *et al.* [S47]. The complete list of clusters is listed in Supporting Table S2.

**References**

S1. Pietrocola G, et al. (2006) Multiple interactions of FbsA, a surface protein from *Streptococcus agalactiae*, with fibrinogen: affinity, stoichiometry, and structural characterization. *Biochem* 45: 12840–52.

S2. Pierno M, Maravigna L, Piazza R, Visai L, Speziale P (2006) FbsA-driven fibrinogen polymerization: a bacterial "deceiving strategy". *Phys Rev Lett* 96: 028108.

S3. Jonsson IM, Pietrocola G, Speziale P, Verdrengh M, Tarkowski A (2005) Role of fibrinogen-binding adhesin expression in septic arthritis and septicemia caused by *Streptococcus agalactiae*. *J Infect Dis* 192: 1456–64.

S4. Gutekunst H, Eikmanns BJ, Reinscheid DJ (2004) The novel fibrinogen-binding protein FbsB promotes *Streptococcus agalactiae* invasion into epithelial cells. *Infect Immun* 72: 3495–504.

S5. Rosenau A, et al. (2007) Evaluation of the ability of *Streptococcus agalactiae* strains isolated from genital and neonatal specimens to bind to human fibrinogen and correlation with characteristics of the fbsA and fbsB genes. *Infect Immun* 75: 1310–7.

S6. Pracht D, et al. (2005) PavA of *Streptococcus pneumoniae* modulates adherence, invasion, and meningeal inflammation. *Infect Immun* 73: 2680–9.

S7. Beckmann C, Waggoner JD, Harris TO, Tamura GS, Rubens CE (2002) Identification of novel adhesins from Group B streptococci by use of phage display reveals that C5a peptidase mediates fibronectin binding. *Infect Immun* 70: 2869–76.

S8. Tamura GS, Hull JR, Oberg MD, Castner DG (2006) High-affinity interaction between fibronectin and the group B streptococcal C5a peptidase is unaffected by a naturally occurring four-amino-acid deletion that eliminates peptidase activity. *Infect Immun* 74: 5739–46.

S9. Spellerberg B, et al. (1999) Lmb, a protein with similarities to the LraI adhesin family, mediates attachment of *Streptococcus agalactiae* to human laminin. *Infect Immun* 67: 871–8.

S10. Elsner A, et al. (2002) Involvement of Lsp, a member of the LraI-lipoprotein family in *Streptococcus pyogenes*, in eukaryotic cell adhesion and internalization. *Infect Immun* 70: 4859–69.

S11. Tenenbaum T, et al. (2007) *Streptococcus agalactiae* invasion of human brain microvascular endothelial cells is promoted by the laminin-binding protein Lmb. *Microbes Infect* 9: 714–20.

S12. Lauer P, et al. (2005) Genome analysis reveals pili in Group B Streptococcus. *Science* 309: 105.

S13. Maione D, et al. (2005) Identification of a universal Group B streptococcus vaccine by multiple genome screen. *Science* 309: 148–50.

S14. Krishnan V, et al. (2007) An IgG-like domain in the minor pilin GBS52 of *Streptococcus agalactiae* mediates lung epithelial cell adhesion. *Structure* 15: 893–903.

S15. Gravekamp C, Rosner B, Madoff LC (1998) Deletion of repeats in the alpha C protein enhances the pathogenicity of group B streptococci in immune mice. *Infect Immun* 66: 4347–54.

S16. Larsson C, Stålhammar-Carlemalm M, Lindahl G (1999) Protection against experimental infection with group B streptococcus by immunization with a bivalent protein vaccine. *Vaccine* 17: 454–8.

S17. Larsson C, Stålhammar-Carlemalm M, Lindahl G (1996) Experimental vaccination against group B streptococcus, an encapsulated bacterium, with highly purified preparations of cell surface proteins Rib and alpha. Infect Immun 64: 3518–23.

S18. Baron MJ, Bolduc GR, Goldberg MB, Aupérin TC, Madoff LC (2004) Alpha C protein of group B Streptococcus binds host cell surface glycosaminoglycan and enters cells by an actin-dependent mechanism. *J Biol Chem* 279: 214–23.

S19. Bolduc GR, Baron MJ, Gravekamp C, Lachenauer CS, Madoff LC (2002) The alpha C protein mediates internalization of group B Streptococcus within human cervical epithelial cells. *Cell Microbiol* 4: 751–8.

S20. Pritzlaff CA, et al. (2001) Genetic basis for the beta-haemolytic/cytolytic activity of group B Streptococcus. *Mol Microbiol* 39: 236.

S21. Liu GY, et al. (2004) Sword and shield: linked group B streptococcal beta-hemolysin/cytolysin and carotenoid pigment function to subvert host phagocyte defense. *Proc Natl Acad Sci USA* 101: 14491–6.

S22. Ring A, et al. (2002) Synergistic action of nitric oxide release from murine macrophages caused by group B streptococcal cell wall and beta-hemolysin/cytolysin. *J Infect Dis* 186: 1518–21.

S23. Doran KS, Liu GY, Nizet V (2003) Group B streptococcal beta-hemolysin/cytolysin activates neutrophil signaling pathways in brain endothelium and contributes to development of meningitis. *J Clin Invest* 112: 736–44.

S24. Nizet V, et al. (1996) Group B streptococcal beta-hemolysin expression is associated with injury of lung epithelial cells. *Infect Immun* 64: 3818–26.

S25. Gibson RL, Nizet V, Rubens CE (1999) Group B streptococcal beta-hemolysin promotes injury of lung microvascular endothelial cells. *Pediatr Res* 45: 626–34.

S26. Doran KS, Chang JCW, Benoit VM, Eckmann L, Nizet V (2002) Group B streptococcal beta-hemolysin/cytolysin promotes invasion of human lung epithelial cells and the release of interleukin-8. *J Infect Dis* 185: 196–203.

S27. Lang S, Palmer M (2003) Characterization of *Streptococcus agalactiae* CAMP factor as a pore-forming toxin. *J Biol Chem* 278: 38167–73.

S28. Li S, Jedrzejas MJ (2001) Hyaluronan binding and degradation by *Streptococcus agalactiae* hyaluronate lyase. *J Biol Chem* 276: 41407–16.

S29. Pritchard DG, et al. (2000) Characterization of the active site of group B streptococcal hyaluronan lyase. *Proteins* 40: 126–34.

S30. Mello LV, De Groot BL, Li S, Jedrzejas MJ (2002) Structure and flexibility of *Streptococcus agalactiae* hyaluronate lyase complex with its substrate. Insights into the mechanism of processive degradation of hyaluronan. *J Biol Chem* 277: 36678–88.

S31. Adderson EE, et al. (2003) Subtractive hybridization identifies a novel predicted protein mediating epithelial cell invasion by virulent serotype III group B *Streptococcus agalactiae*. *Infect Immun* 71: 6857–63.

S32. Jerlström PG, Talay SR, Valentin-Weigand P, Timmis KN, Chhatwal GS (1996) Identification of an immunoglobulin A binding motif located in the beta-antigen of the c protein complex of group B streptococci. *Infect Immun* 64: 2787–93.

S33. Areschoug T, Stålhammar-Carlemalm M, Karlsson I, Lindahl G (2002) Streptococcal beta protein has separate binding sites for human factor H and IgA-Fc. *J Biol Chem* 277: 12642–8.

S34. Jarva H, Jokiranta TS, Würzner R, Meri S (2003) Complement resistance mechanisms of streptococci. *Mol Immunol* 40: 95–107.

S35. Cieslewicz MJ, et al. (2005) Structural and genetic diversity of group B streptococcus capsular polysaccharides. *Infect Immun* 73: 3096–103.

S36. Dore N, Bennett D, Kaliszer M, Cafferkey M, Smyth CJ (2003) Molecular epidemiology of group B streptococci in Ireland: associations between serotype, invasive status and presence of genes encoding putative virulence factors. *Epidemiol Infect* 131: 823–33.

S37. Rubens CE, Wessels MR, Heggen LM, Kasper DL (1987) Transposon mutagenesis of type III group B Streptococcus: correlation of capsule expression with virulence. *Proc Natl Acad Sci USA* 84: 7208–12.

S38. Lewis AL, Hensler ME, Varki A, Nizet V (2006) The group B streptococcal sialic acid O-acetyltransferase is encoded by *neu*D, a conserved component of bacterial sialic acid biosynthetic gene clusters. *J Biol Chem* 281: 11186–92.

S39. Unkmeir A, et al. (2002) Lipooligosaccharide and polysaccharide capsule: virulence factors of *Neisseria meningitidis* that determine meningococcal interaction with human dendritic cells. *Infect Immun* 70: 2454–62.

S40. Edwards MS, Kasper DL, Jennings HJ, Baker CJ, Nicholson-Weller A (1982) Capsular sialic acid prevents activation of the alternative complement pathway by type III, group B streptococci. *J Immunol* 128: 1278–83.

S41. Marques MB, Kasper DL, Pangburn MK, Wessels MR (1992) Prevention of C3 deposition by capsular polysaccharide is a virulence mechanism of type III group B streptococci. *Infect Immun* 60: 3986–93.

S42. Harris TO, Shelver DW, Bohnsack JF, Rubens CE (2003) A novel streptococcal surface protease promotes virulence, resistance to opsonophagocytosis, and cleavage of human fibrinogen. *J Clin Invest* 111: 61–70.

S43. Jones AL, Needham RHV, Clancy A, Knoll KM, Rubens CE (2003) Penicillin-binding proteins in *Streptococcus agalactiae*: a novel mechanism for evasion of immune clearance. *Mol Microbiol*  47: 247–56.

S44. Hamilton A, et al. (2006) Penicillin-binding protein 1a promotes resistance of group B streptococcus to antimicrobial peptides. *Infect Immun* 74: 6179–87.

S45. Jones AL, Mertz RH, Carl DJ, Rubens CE (2007) A streptococcal penicillin-binding protein is critical for resisting innate airway defenses in the neonatal lung. *J Immunol* 179: 96–202.

S46. Terabe M, Washio T, Motoda H (2001) S3Bagging: Fast Classifier Induction Method with Subsampling and Bagging. In: Hoffmann F, Hand DJ, Adams N, Fisher D, Guimaraes G, editors. *Advances in Intelligent Data Analysis*. London, UK: Springer-Verlag. pp. 177-86

S47. Hirsh AE, Fraser HB (2001) Protein dispensability and rate of evolution. *Nature* 411: 1046-9.
